# Development of the Community-based complex Interventions to sustain Independence in Older People (CII-OP) typology: a qualitative synthesis of interventions in randomised controlled trials

**DOI:** 10.1093/ageing/afae102

**Published:** 2024-05-25

**Authors:** Thomas Frederick Crocker, Magda Jordão, Natalie Lam, Ridha Ramiz, Lubena Mirza, Ismail Patel, Alison Ellwood, Eleftheria Patetsini, Joie Ensor, Anne Forster, Andrew Clegg, John Gladman, The HTA complex interventions review team, Deirdre Andre, Ram Bajpai, Matthew Bond, John Green, Jessica Morgan, Richard D Riley, Rebecca Walford

**Affiliations:** Academic Unit for Ageing and Stroke Research (University of Leeds), Bradford Institute for Health Research, Bradford Teaching Hospitals NHS Foundation Trust, Bradford, UK; Academic Unit for Ageing and Stroke Research (University of Leeds), Bradford Institute for Health Research, Bradford Teaching Hospitals NHS Foundation Trust, Bradford, UK; Academic Unit for Ageing and Stroke Research (University of Leeds), Bradford Institute for Health Research, Bradford Teaching Hospitals NHS Foundation Trust, Bradford, UK; Academic Unit for Ageing and Stroke Research (University of Leeds), Bradford Institute for Health Research, Bradford Teaching Hospitals NHS Foundation Trust, Bradford, UK; Academic Unit for Ageing and Stroke Research (University of Leeds), Bradford Institute for Health Research, Bradford Teaching Hospitals NHS Foundation Trust, Bradford, UK; Academic Unit for Ageing and Stroke Research (University of Leeds), Bradford Institute for Health Research, Bradford Teaching Hospitals NHS Foundation Trust, Bradford, UK; Academic Unit for Ageing and Stroke Research (University of Leeds), Bradford Institute for Health Research, Bradford Teaching Hospitals NHS Foundation Trust, Bradford, UK; Academic Unit for Ageing and Stroke Research (University of Leeds), Bradford Institute for Health Research, Bradford Teaching Hospitals NHS Foundation Trust, Bradford, UK; Institute of Applied Health Research, College of Medical and Dental Sciences, University of Birmingham, Birmingham, UK; Centre for Prognosis Research, Keele School of Medicine, Keele University, Keele, UK; Academic Unit for Ageing and Stroke Research (University of Leeds), Bradford Institute for Health Research, Bradford Teaching Hospitals NHS Foundation Trust, Bradford, UK; Academic Unit for Ageing and Stroke Research (University of Leeds), Bradford Institute for Health Research, Bradford Teaching Hospitals NHS Foundation Trust, Bradford, UK; Centre for Rehabilitation and Ageing Research, Academic Unit of Injury, Inflammation and Recovery Sciences, University of Nottingham; Health Care of Older People, Nottingham University Hospitals NHS Trust, Nottingham, UK

**Keywords:** taxonomy, classification, primary health care practice, rehabilitation therapy, frail, older people

## Abstract

**Introduction:**

Community-based services to sustain independence for older people have varying configurations. A typology of these interventions would improve service provision and research by providing conceptual clarity and enabling the identification of effective configurations. We aimed to produce such a typology.

**Method:**

We developed our typology by qualitatively synthesising community-based complex interventions to sustain independence in older people, evaluated in randomised controlled trials (RCTs), in four stages: (i) systematically identifying relevant RCTs; (ii) extracting descriptions of interventions (including control) using the Template for Intervention Description and Replication; (iii) generating categories of key intervention features and (iv) grouping the interventions based on these categories. PROSPERO registration: CRD42019162195.

**Results:**

Our search identified 129 RCTs involving 266 intervention arms. The Community-based complex Interventions to sustain Independence in Older People (CII-OP) typology comprises 14 action components and 5 tailoring components. Action components include procedures for treating patients or otherwise intended to directly improve their outcomes; regular examples include formal homecare; physical exercise; health education; activities of daily living training; providing aids and adaptations and nutritional support. Tailoring components involve a process that may result in care planning, with multiple action components being planned, recommended or prescribed. Multifactorial action from care planning was the most common tailoring component. It involves individualised, multidomain assessment and management, as in comprehensive geriatric assessment. Sixty-three different intervention types (combinations) were identified.

**Conclusions:**

Our typology provides an empirical basis for service planning and evidence synthesis. We recommend better reporting about organisational aspects of interventions and usual care.

## Key Points

- There is a lack of conceptual clarity about community-based health and social care interventions for older people.
- We developed a typology from the literature, comprising 14 action components and 5 tailoring components.
- A tailoring component, multifactorial action from care planning, was most commonly studied.
- Formal homecare, physical exercise and health education were the most common action components studied.
- This typology is valuable for synthesising evidence and enabling dialogue with policy makers, service commissioners and providers.

## Background

The threats to independence in old age typically arise from the combination of frailty, multi-morbidity and disability rather than a single health condition [1–3]. In response, interventions to sustain independence are often complex, involving multiple, tailored intervention components [4, 5]. Previous systematic reviews of literature evaluating community-based complex interventions to sustain independence in older people have broadly indicated that they may be beneficial [5–8]. However, these interventions have variable components and configurations. These reviews have not illuminated which components or configurations are most effective—crucial information for policymakers, commissioners and providers. A barrier to this understanding is the lack of an agreed typology for grouping these interventions [9].

Experimental interventions are often labelled in terms of the professional providing them (e.g. nurse-led), the location (e.g. geriatric clinic) or a brief but inconsistently defined phrase (e.g. case management) [3]. Furthermore, control group interventions are often just described as ‘usual care’ despite huge variations in this across time, location, population and setting [10, 11]. The Template for Intervention Description and Replication (TIDieR) reporting guide was developed to improve reporting in clinical trials, particularly for complex interventions [12]. TIDieR comprises 12 features for each intervention: a brief name, why, what materials, what procedures, who provided, how, where, when and how much, tailoring, possible modifications and how well planned and executed [12]. While it was developed in collaboration with the Consolidated Standards of Reporting Trials (CONSORT) steering group [12, 13] and is recommended for application in systematic reviews [14], it is a template for describing interventions rather than a typology.

Existing classification systems are also unsuitable for supporting evidence synthesis. Some require a high level of detail unavailable from published trials and unlikely to group interventions (e.g. International Classification of Healthcare Interventions (ICHI) [15]); other approaches use inconsistent features of interventions, such as aims, means or location (e.g. National Institute for Health and Care Excellence (NICE) multimorbidity guideline and Cochrane Effective Practice and Organisation of Care (EPOC) taxonomy) [3, 16], which are unlikely to produce mutually exclusive groups. Therefore, we planned to produce a typology capable of systematically grouping interventions based on trial reports, as part of a broader evidence synthesis project [17–19].

## Methods

We conducted a qualitative synthesis of community-based complex interventions for sustaining the independence of older people that were evaluated in randomised controlled trials (RCTs). This involved four stages: (i) systematic identification of relevant RCTs and related publications, (ii) the extraction of descriptions of the interventions (including control and comparator interventions) using TIDieR, (iii) a qualitative analysis of the data generating categories of key intervention features and (iv) grouping the interventions based on these categories.

### Systematic identification of studies

The search strategy was part of our systematic review and network meta-analysis (NMA) of community-based complex interventions to sustain independence in older people [17–19], registered on PROSPERO (CRD42019162195) and detailed in Appendix 1.

#### Study selection

Eligible studies were RCTs or cluster-RCTs (cRCTs). Eligible participant populations were older people living at home (mean age 65 years or older). Populations, including participants living in residential/nursing homes, were excluded. An eligible intervention:

- was both initiated and mainly provided in the community;
- included two or more interacting components (intervention practices, structural elements and contextual factors);
- was targeted at the individual person, with the provision of appropriate specialist care; and
- focused on sustaining (maintaining or improving) the person’s independence.

Eligible comparators were usual care, placebo, attention control or a different complex intervention that met our criteria. We excluded condition-specific interventions, focusing on those suitable for an ageing population in general. We also excluded fall-prevention interventions, which have been synthesised in NMAs [20, 21].

### Data extraction

For all eligible interventions and comparators in the included studies, we extracted data from primary and related/secondary publications in the form of codes within a coding structure based on TIDieR (Appendix 2) and descriptive text organised in a table for each TIDieR item.

One reviewer (MJ, NL, RR, LM, IP or EP) coded and summarised each intervention, and one other reviewer (TC) assessed this, with any disagreements resolved by consensus discussion. To begin, a reviewer familiarised themselves with the intervention descriptions in a study. They used NVivo 12 Plus (QSR International Pty Ltd, Hawthorn East, Victoria, Australia) to conduct open coding within the TIDieR coding structure, creating new codes as required. The review team was guided by a shared code book (Appendix 3). Coding and summarising of each intervention were checked by TC and subsequently revised in agreement with the original coder; revisions were disseminated across the team to further promote consistency. Guidance was sought from the project management group (PMG) if necessary.

### Qualitative analysis

An iterative approach to analysis was taken, consolidating the coding as we progressed and collapsing the first-stage codes into categories and subcategories. To support our aim of developing exclusive, distinct intervention groups, we sought to develop exclusive categories within each TIDieR domain (e.g. 06. How) or subdomain (e.g. 06.1. Individual-group (size)). We also refined our coding scheme, seeking more detailed or less detailed coding within a domain in response to reflections about their usefulness for grouping the interventions (see stage 4). Theory and practice were considered by contrasting the codes with those in existing classification systems [3, 16, 22] and by involving advisory groups. This included comparing and contrasting the information extracted, as well as discussions among reviewers, within the wider research team (including the PMG), and with the Implementation Advisory Group (IAG). The IAG members were part of a special interest group on frailty, including clinicians, policy makers, commissioners and leaders of delivery organisations. Key categories were presented, followed by an open discussion and further written feedback, as relevant. The categories were revised until the research team reached an agreement.

### Intervention grouping

A similar iterative approach was followed to group the interventions. This approach served to establish general principles for the typology, specifically which of the categories would contribute to the intervention grouping and how they would be combined. Proposals were made and discussed by members of the research team on the basis of their reflections on working with the intervention descriptions and coding. Provisional approaches were then discussed with the IAG and our Patient and Public Involvement (PPI) group to assure the meaningfulness and relevance of the developed groups and to prioritise areas for clarification and development. The PPI group included three older people who were part of the experienced Frailty Oversight Group (FOG) and have provided lay representation for multiple older people’s health research studies [23]. Written lay language materials were sent to all PPI members, followed by a discussion of relevance and clarity. The changes suggested were integrated into the public-facing names and plain language descriptions for the categories that contributed to the grouping, intended to assist with dissemination and conversations with patients about the evidence.

## Results

### Typology

Briefly, the Community-based complex Interventions to sustain Independence in Older People (CII-OP) typology that we developed enables the classification of interventions based on two kinds of core components: action components and tailoring components. Action components include procedures for treating patients or otherwise intended to directly improve their outcomes, and tailoring components involve a process that may result in care planning, with multiple action components being planned, recommended or prescribed. There are 14 action components and 5 tailoring components in total in the CII-OP typology. Collectively, these 19 components identified every kind of active procedure that was intended to be delivered to all participants in the study arms.

The following sections provide detailed results from the stages of our typology development and application, including definitions, examples and justification of our final typology (CII-OP).

### Systematic search

We screened 40,291 records and included 129 studies consisting of 496 reports. There were 266 eligible intervention arms (122 two-arm studies, 6 three-arm studies and 1 four-arm study). Appendix 4 details the PRISMA flow diagram [24] and which studies and reports were included.

### Data extraction

All 266 eligible trial intervention arms were coded, and TIDieR descriptions were produced. Appendix 2 contains the original coding structure, coding after 29 studies had been extracted when we began grouping/collapsing first-stage codes, and the final coding structure. During coding, we identified that important organisational features, such as a multidisciplinary team or care coordination role, were not captured within the TIDieR domains, and so we added domain 6b, ‘How organised’ to our coding structure. We were able to populate 71.7% of TIDieR cells in total. Completion was far higher among experimental interventions (87.2%) compared with control interventions (including ‘usual care’, 54.6%). Completion varied by TIDieR domain, with nearly all experimental interventions described to some extent for the first nine TIDieR domains (1365/1390 = 98.2%), while the final three TIDieR items were only partially populated (‘Modifications’, ‘How well (planned)’, and ‘How well (actual)’: 211/417 = 50.6%). For control interventions, only ‘Brief name’, ‘What (procedures)’, ‘Where’ and ‘When and how much’ were routinely described (500/508 = 98.4%; total for other TIDieR items: 902/1651 = 54.6%). Appendix 5 illustrates an example of a description for one intervention.

### Qualitative analysis

For consistency, we defined key terms for the analysis: ‘intervention’ to denote the totality of care, ‘action’ to denote directly therapeutic or preventive procedures undertaken with individuals receiving the intervention and ‘component’ to denote separable procedural elements within the whole package. Box 1 justifies and elaborates on these definitions and the two types of component that became the dimensions of our typology.

**Table:** 

**Box 1 Descriptions of key terms used in the data analysis**	
**Key term**	**Description**
Intervention	The whole package of care that a population is randomly assigned to receive in a treatment or comparison arm in an RCT/cRCT, including any usual care. In keeping with TIDieR, the term does not refer to smaller units, such as the procedures specific to the experimental arm alone or a single component [12].
Action	Procedures for treating participants or otherwise intended to directly improve their outcomes (called ‘intervention’ in some models of the clinical process [25, 26]). An intervention (as defined in this article) often includes multiple actions. Actions are distinguished from other procedures, including assessment, care planning and non-clinical (i.e. organisational) implementation and management.
Component	Procedures that are similarly located in time and space are closely interrelated and could be implemented independently of others [27]. For example, a physical exercise programme may involve social interaction based on the relationship with a therapist/instructor. However, the social interaction is closely interrelated with the delivery of the exercise itself, and the delivery would need to be changed to split out or remove this aspect. Therefore, the social interaction involved in the physical exercise programme is not considered a separate component. Similarly, an instructor may assess the performance of a participant and tailor or grade the exercise programme, but these procedures are performed to inform the exercise, so they are also part of the same component.
Action component	An action component includes action procedures and may include other procedures related to the action, as in the example above.
Tailoring component	A tailoring component includes a process that may result in care planning, with multiple action components being planned, recommended or prescribed. A tailoring component does not itself include action procedures.

#### Action components

Some action components provided to the participants were relatively easy to categorise, such as physical exercise, which has a widely-used definition and was confidently identified. These participant-level components usually included inter-related procedures (see Box 1), but we considered that these were provided to enable or enhance the actions, and therefore it was the actions that defined the component. We recognised that within each category of action component there were differences in the details between different cases, which could be clinically important. For example, physical exercise could be focused on strength training or cardiorespiratory fitness, and training regimes could differ according to intensity and duration. However, given the variety of such details in the data, we did not split the component of physical exercise into several components, as doing so would produce too many components for any useful effectiveness synthesis to be performed. Similar reasoning was applied to other components.

Other action components were more difficult to define, such as when there was potential for overlap between provisional categories. An example was a component described as ‘health education’, which seemed to overlap with descriptions of ‘support for self-management’, ‘health promotion’ and even ‘motivational interviewing’. Box 2 describes the iterative process from coding, content analysis and expert input that led us to define the ‘health education’ component. Similar approaches were taken with other components.

Box 2 Summary of the iterative process, including coding (stage 2), qualitative analysis (stage 3) and expert input in the development of the ‘health education’ component

(1) Reviewers open-coded the interventions, creating unique codes for each intervention within the action procedures subdomain, such as ‘Self-management skills module’ and ‘Health promotion education’.
(2) Based on comparing and contrasting, two education action components were created: ‘Educating and training in health maintenance and self-care’ and ‘Educating and training in self-management skills, including problem solving’. The first focused more on information provision, and the second on self-management.
(3) The components were discussed with members of the PMG, including: Should information provision be enough to consider there is an education process at play, or is self-management necessary? Should individual and group education be separated? What is the difference between individual education and providing health-related advice as part of clinical practice?
(4) To better address the questions that emerged from the previous discussion, reviewers recoded education and self-management aspects separately for a wider pool of studies. A matrix of studies with and without education and self-management to analyse (non-)overlap and new contrasting examples of individual provision of information were also developed.
(5) The new coding and analysis were presented in an expert discussion with members of the PMG, and consensus was reached regarding the previous questions. The consensus redefined the health education category as the provision of information individually or in groups, independent of the presence of self-management techniques. Additionally, to distinguish health education from the health advice that routinely occurs in clinical practice, this definition required the existence of a pre-determined set of educational topics to be delivered to participants, although it allowed the individual selection to be tailored to the participant.

#### Distinguishing core components

Across all interventions, different components were delivered to the population in varying degrees. For example, some interventions included action components delivered to all participants, such as physical exercise and nutritional support. In other interventions, an assessment led to some action components being delivered to low percentages of participants. Looking ahead to our planned grouping of interventions, this posed a problem related to the importance of our components and regarding consistency between studies reported with differing levels of detail.

‘Core components’ of complex interventions are often defined as the essential elements containing the active ingredients through which an intervention is theorised to work in a population [28]. Since the complete programme theory for an intervention was rarely explicit, we decided to consider action components intended for all participants to be types of ‘core components’. By contrast, if action components were selected in different configurations for different participants, we considered these to be additional components. We collectively termed these configurations ‘multifactorial action’, which we recognised as the consequence of a tailoring process, including care planning. Such a process may also include identification and assessment, and where any elements of that process were intended for all participants, we considered the tailoring process to be a core component.

#### Core components

Using the concepts and processes described above, we identified 19 core components among the 266 interventions, which are described in Box 3. As above, these components applied to a population (they were intended for all participants) rather than at participant-level only. Fourteen of the nineteen core components were action components. Five further core components were identified, where multifactorial action could result from a tailoring process.

Box 3 The 19 core components identified in the 266 interventions: 14 action components and 5 tailoring components

**Action components** 

(1) Formal homecare (identified in 41 interventions). Homecare involves frequent visits at home by health or care practitioners to provide services, including support with household tasks, self-care and nursing care.
(2) Physical exercise (30 interventions). Support to carry out physical exercise (physical activity aimed at improving physical fitness), through training sessions or specialised advice and activities. This is more specific and detailed than what may be provided in health education.
(3) Health education (26 interventions). Providing information about a set of health topics in group sessions or one-on-one according to a pre-specified plan or protocol. Advice provided as part of clinical practice and written-only information are not sufficient.
(4) Activities of daily living (ADL) training (13 interventions). Providing support and advice to practice ADL and conduct it independently with more success and/or safety.
(5) Providing aid and adaptations (12 interventions). Providing equipment or technology to assist with independence in ADL (excluding communication and social engagement, see below). Examples include kitchen aids, grab rails or a system of sensors to turn on lights.
(6) Nutritional support (10 interventions). Providing nutrition-related support (including information about theory or practice). May include activities, such as completing a food diary, weight monitoring or the provision of supplements.
(7) Psychological (mood) therapy (4 interventions). Providing activities aimed at maintaining or supporting helpful psychological processes to deal with a variety of areas (e.g. anxiety). Includes education and training about psychological principles and techniques with this aim.
(8) Technology for communication and engagement (4 interventions). Providing devices/systems that support communication with friends, family, neighbours or the community (e.g. tablet and social media applications) and usually support to use them.
(9) Cognitive training (3 interventions). Providing training in cognitive activities (e.g. memory), including practical exercises and information provision about cognitive strategies.
(10) Engagement in meaningful activities (3 interventions). Providing support to identify and participate in activities that the participant finds meaningful, for example, leisure, craft or volunteering.
(11) Care voucher provision (2 interventions). Providing a voucher that can be spent on health and personal care services. Advice and administrative support on how the voucher can be spent are also provided.
(12) Alternative medicine, such as homeopathy and naturopathy (1 intervention). Providing naturopathic and/or homeopathic consultation and treatment.
(13) Social skills training (1 intervention). Providing information and training on social skills (e.g. assertiveness).
(14) Welfare rights advice (1 intervention). Providing tailored advice and support to access available welfare services and benefits.

**Tailoring components:** 

(15) Multifactorial action from care planning (117 interventions), with or without medication review and self-management

In many interventions, there was an assessment and care-planning process for all participants, which was used to select which actions would be delivered to whom. Here, an individualised care plan incorporating selected actions is an expected output for participants. Across the population, these actions are to include more than any one of the action components above, and the selection of components is to be varied between participants.

This component is broad and warrants further distinction based on particular aspects. Two relevant aspects were identified: the presence or absence of a medication review and self-management strategies (these aspects were not mutually exclusive).

A medication review was provided with the multifactorial-action component in 53 interventions. In these cases, medication changes are one of the possible recommended actions, selected based on assessment and planning.

Self-management strategies were integrated into the assessment and care-planning process in 26 such interventions. These are specific psychological strategies to support behaviour change and the self-management of health conditions or risks identified in the assessment. The strategies include, for example, motivational interviewing, problem-solving and goal setting; we did not recognise self-management strategies based only on general comments about encouragement of behaviour change or self-management.

(16) Routine review following multifactorial action from care planning (82 interventions)

Routine review is a process of scheduled, regular follow-ups for all participants subsequent to multifactorial action from care planning, with or without medication review and self-management. This does not include additional contacts that are *ad hoc* or determined by need, which are present in almost all examples of multifactorial action from care planning.

(17) Medication review (4 interventions)

In addition to being an aspect that we found to be relevant as part of multifactorial action from care planning, medication review was provided on its own in some interventions. In this component, medication-related changes follow from an assessment focused on the medication regimen, for example, by identifying problems with polypharmacy.

(18) Routine risk-screening (7 interventions)

Routine risk screening involves the process of identifying members of a population as a preliminary step. Only those identified as having possible health problems or being at increased risk of adverse outcomes receive subsequent multifactorial action from care planning. A standardised tool, such as a questionnaire or analysis of health records, is used in the identification process.

(19) Monitoring (2 interventions)

Monitoring also involves a process to identify those with possible changes in day-to-day health for further multifactorial action from care planning. In this component, the identification process is based on regular (e.g. daily) measurement of bodily functions, such as blood pressure and heart rate, which trigger multifactorial action from care planning if thresholds are met.

#### Plain language descriptions

The public-facing names and plain language descriptions for each component and aspect we developed with our PPI group are provided in Appendix 6. The components are also organised into topics here, which were the suggestions of PPI members.

### Intervention grouping

#### A typology focused on actions

The development of an intervention typology required us to decide which attributes of an intervention should be prioritised in how interventions were grouped. Following discussions in the PMG, which considered several alternatives (e.g. the roles and professions of those delivering the intervention), we decided to focus primarily on the actions. Actions were considered the most likely agents of change in the interventions and thus they identified critical components. Tailoring components were only considered where they resulted in multifactorial action—sets of action components that vary between participants. An intervention could include both core action and tailoring components, in which some action components were intended for all participants and others were tailored.

#### Unpicking usual care

Many clinical studies compared an ‘active’ intervention against ‘usual care’. Usual care was often not well described and would be expected to differ between countries and time periods. Importantly, in the context of community interventions, usual care does not mean ‘no care’ and can include components as used in ‘active’ intervention arms, either for some participants or the whole study population. The approach we took was to treat usual care or standard intervention arms consistently with experimental arms and identify any core components that were included. Then, we labelled usual care, where there was no particular component intended to be delivered to all participants, as ‘available care’. This acknowledged the availability of a wide range of primary, secondary, tertiary and wider community services that would be accessed by some, but not all, participants without specifying their nature. We also included here interventions that only consisted of actions that were not intended or anticipated to affect an individual’s independence, such as attention control, placebo and other minor actions, such as giving a leaflet. With this definition, some of the control, comparison or usual care interventions were classified simply as ‘available care’, while for other such interventions, components additional to ‘available care’ were clearly classifiable; most commonly, this was when all of the control group participants received homecare or multifactorial action from care planning.

#### Application of the intervention typology

We classified the 266 interventions according to the 19 core components and aspects: 14 action components, five tailoring components, and, in the case of multifactorial action, whether or not the component included medication review, self-management strategies or both. This gave us 63 different combinations (or intervention types), which formed our groups, as detailed in Table 1. Given their multiplicity, we did not seek to further disaggregate the interventions based on other attributes. Forty-seven interventions were a combination of action components only; sixty-six were defined by tailoring components only, while 55 included both tailoring and action components. However, 98 interventions had no core components, all of which were control arms that formed one group (the largest in our sample) termed ‘available care’ as above. The other groups, including more than 10 interventions, were multifactorial action and review with medication review (24 interventions), multifactorial action and review (15 interventions) and homecare (12 interventions). Eight groups contained two interventions, while 37 groups contained only one intervention. Fourteen of the sixty-three intervention types featured only one core component: an action component in nine types and a tailoring component in five types (including three types of multifactorial action from care planning). The greatest combination of components was six in the type ‘ADL, aids, education, exercise, multifactorial action and review with medication review and self-management’. TIDieR descriptions of groups containing more than one intervention are presented in Appendix 7.

**Table 1:** Interventions classified according to the typology

**Intervention group label**		**Core action components**															**Core tailoring components and aspects**						
	**Interventions**	**hmcr**	**ADL**	**aids**	**cgn**	**comm**	**educ**	**eng**	**exrc**	**hmnt**	**ntr**	**psyc**	**sst**	**vchr**	**wlfr**		**mfa**	**mfar**	**w/med** ^**a**^	**w/slfm** ^**a**^	**med**	**mntr**	**rsk**
ADL	2		●																				
ADL and exercise	1		●						●														
ADL, aids and exercise	1		●	●					●														
ADL, aids, education, exercise, multifactorial-action and review with medication review and self-management	2		●	●			●		●								●	●	●	●			
ADL, medication-review, nutrition and social-skills	1		●								●		●								●		
ADL, nutrition and exercise	1		●						●		●												
Aids	2			●																			
Aids, cognitive training, telecoms, and monitoring	1			●	●	●																●	
Aids, education and telecoms	1			●		●	●																
Aids, multifactorial-action and review	1			●													●	●					
Available care	98																						
Care voucher	1													●									
Care voucher, education, multifactorial-action and review with medication review and self-management	1						●							●			●	●	●	●			
Cognitive training, medication-review, nutrition and exercise	1				●				●		●										●		
Cognitive training, nutrition and exercise	1				●				●		●												
Education	3						●																
Education and multifactorial-action	1						●										●						
Education and risk-screening	1						●																●
Education, exercise, multifactorial-action and review with medication review and self-management	2						●		●								●	●	●	●			
Education, exercise, multifactorial-action and review with self-management	1						●		●								●	●		●			
Education, multifactorial-action and review	1						●										●	●					
Education, multifactorial-action and review with medication review	3						●										●	●	●				
Education, multifactorial-action and review with medication review and self-management	5						●										●	●	●	●			
Education, multifactorial-action and review with self-management	2						●										●	●		●			
Exercise	7								●														
Exercise and multifactorial-action with medication review	1								●								●		●				
Exercise and multifactorial-action with medication review and self-management	1								●								●		●	●			
Exercise and psychology	3								●			●											
Exercise, multifactorial-action and review	1								●								●	●					
Exercise, multifactorial-action and review with medication review	1								●								●	●	●				
Exercise, multifactorial-action and review with medication review and self-management	1								●								●	●	●	●			
Homecare	12	●																					
Homecare and aids	1	●		●																			
Homecare and medication-review	1	●																			●		
Homecare and multifactorial-action	5	●															●						
Homecare and nutrition	1	●									●												
Homecare, ADL, aids and multifactorial-action	1	●	●	●													●						
Homecare, ADL, aids and multifactorial-action with self-management	1	●	●	●													●			●			
Homecare, ADL, multifactorial-action and review with self-management	3	●	●														●	●		●			
Homecare, aids and telecoms	1	●		●		●																	
Homecare, alternative-medicine and exercise	1	●							●	●													
Homecare, education, multifactorial-action and review	1	●					●										●	●					
Homecare, multifactorial-action and review	6	●															●	●					
Homecare, multifactorial-action and review with medication review	3	●															●	●	●				
Homecare, multifactorial-action and review with medication review and self-management	1	●															●	●	●	●			
Homecare, multifactorial-action and review with self-management	2	●															●	●		●			
Homecare, nutrition, multifactorial-action and review	1	●									●						●	●					
Meaningful-activities and education	2						●	●															
Meaningful-activities and multifactorial-action with self-management	1							●									●			●			
Medication-review, nutrition and exercise	1								●		●										●		
Monitoring	1																					●	
Multifactorial-action	9																●						
Multifactorial-action and review	15																●	●					
Multifactorial-action and review with medication review	24																●	●	●				
Multifactorial-action and review with medication review and self-management	3																●	●	●	●			
Multifactorial-action and review with self-management	2																●	●		●			
Multifactorial-action with medication review	5																●		●				
Multifactorial-action with medication review and self-management	1																●		●	●			
Nutrition and exercise	3								●		●												
Psychology	1											●											
Risk-screening	6																						●
Telecoms	1					●																	
Welfare-advice	1														●								

^a^Not a component but an aspect of multifactorial action from care planning.

ac, available care; ADL, activities of daily living training; aids, provision of aids and adaptions; cgn, cognitive training; comm, technology for communication and engagement; educ, education; eng, engagement in meaningful activities; exrc, exercise; hmcr, homecare; hmnt, alternative medicine; med, medication review; mfa, multifactorial action; mfar, multifactorial action and follow-on routine review; mntr-mfa, monitoring, which may trigger multifactorial action; ntr, nutritional support; psyc, psychological therapy; rsk-mfa, risk screening, which may trigger multifactorial action; sst, social skills training; vchr, care voucher provision; wlfr, welfare rights advice; w/med, with medication review; w/slfm, with self-management.

## Discussion

We identified 19 core components of community-based complex interventions for older people to sustain independence, along with two aspects of multifactorial action from care planning. We used these to classify 266 published interventions into 63 different types/groups. The largest groups and hence most suitable for pooling in syntheses of intervention effectiveness were ‘multifactorial action and review with medication review’ and ‘multifactorial action and review’, and comparator groups ‘available care’ and ‘homecare’. Thirty-seven groups were formed of one intervention only, limiting the scope for aggregative evidence syntheses. Overall, the intervention types can be divided into those defined by tailoring the population’s care (multifactorial, 41 groups), providing only a standard set of action components (multicomponent, 21 groups), and providing no core components (available care).

This work was underpinned by exhaustive and systematic searching of the RCT literature, so our results are likely to be comprehensive. Additionally, we systematically extracted and analysed published data, meaning our typology was based upon empirical evidence of substantial differences in active intervention content rather than relying on the label applied by the study authors or the prior assumptions of the research team. The typology was also the subject of discussions with external reference groups (IAG and PPI), providing additional reassurance of clinical relevance. The systematic data extraction and reflection also allowed us to complement the set of TIDieR domains, which do not specify an item for organisational features such as team structures, roles and responsibilities. We extracted these additional features wherever possible, which enriched the intervention descriptions and highlighted an important addition to future intervention descriptions.

We acknowledge limitations to our typology. Each of our 19 core components was broad, and there are sub-categories that we did not distinguish in view of our purpose of grouping interventions for synthesis. Our analysis was limited by the variable level of detail in the intervention descriptions provided. The intervention group ‘available care’, representing no core components (for all) but the multiple potential intervention components that may be accessed by an individual, is heterogeneous and variable between countries and times. We did not further divide available care because of limitations of description and because it would further divide all of the intervention groups, as the same additional services are typically available to participants in all arms of a trial. While this does not invalidate our typology based on core intervention components, it limits the value of evidence syntheses that depend on the assumption that ‘available care’ interventions are homogenous.

Related to the above, additional components, not intended for all participants, are not detailed in our typology. Therefore, further components relevant to sustaining independence may still be identified. Our typology does not account for differences in the trial populations to which interventions are delivered (e.g. people living with frailty versus an unselected older population). This is important in interventions that tailor actions according to assessment and care planning and therefore vary what is delivered according to the characteristics of the population. Nevertheless, we believe that our intervention components and typology are sufficiently valid for use in synthesising and interpreting the evidence base.

Our findings provide an evidence-based and more sophisticated alternative to the *ad hoc* grouping typically used elsewhere. For example, an earlier review named five loosely defined intervention groups (geriatric assessment of older people, geriatric assessment of older people assessed as frail, community-based care after hospital discharge, fall prevention and group education and counselling) [5]. While a limited number of categories is intuitively appealing, it can be unclear how they were chosen or why an intervention was placed in one and not another. Having loosely defined intervention groupings is problematic for policymakers, commissioners and service planners when attempting to translate the available evidence into suitable operating models for service delivery.

Although the uneven distribution of interventions in groups may appear to be a limitation of the typology, this merely reflects the varied extent to which the interventions have been investigated in trials and that the typology was developed through a focus on the content of the interventions rather than achieving some preconceived notion of what a good division of interventions would look like.

Our component ‘multifactorial action from care planning’ encompasses the notion of ‘(comprehensive) geriatric assessment’ (CGA), which is widely used, such as in the Prevention of Falls Network Europe (ProFaNE) taxonomy for falls prevention [22], the EPOC taxonomy used by the Cochrane Collaboration for health systems [16] and the earlier review of community-based complex interventions [5]. We found it a more suitable term than CGA because it more precisely described the active intervention (the term CGA does not refer to care planning or action in response to assessment), because multifactorial action from care planning may or may not have taken place whether or not ‘CGA’ was mentioned, and because it avoided having to define whether an assessment was ‘comprehensive’.

Although we excluded falls prevention interventions in this study, our list of action components has a similar level and content as the ProFaNE (falls prevention) taxonomy’s ‘descriptor’ subdomains (e.g. ProFaNE lists ‘exercises’, ‘psychological’, ‘fluid or nutrition therapy’ and ‘environment/assistive technology’, as intervention descriptors) [22]. These similarities support the likelihood that our typology will have some utility beyond the data set from which it was derived and that our approach to conceptualising interventions resonates with others.

Our typology enabled us to progress our NMA of these interventions, which usually estimated low heterogeneity within the intervention groups where this was measurable and could be used by others performing similar syntheses [18]. For example, our findings could be a starting point for a recent Cochrane review call for a taxonomy of integrated care components [9]. Our approach to identifying intervention types could be adopted by others seeking to conceptualise or synthesise other complex interventions. Those wishing to do so may choose to adopt the principles that emerged through this work and allowed us to manage the complexity of the interventions and proceed within the common limitations of intervention descriptions (see Box 4).

Box 4 Principles for trial-intervention typology development that emerged through this work

Principle 1. Intervention types should primarily be defined by actions and procedures that tailor the selection of actions, which can be conceptualised in terms of core components.

Principle 2. To be considered a core component, a procedure must have been intended to be provided to all or almost all the participants.

Principle 3. Trial procedures (e.g. screening for trial eligibility, outcome assessment) generally need not be considered part of the intervention.

Principle 4. Actions that can reasonably be presumed to have a minimal impact on outcomes of interest need not be considered in/contribute to the typology.

Principle 5. Tailoring procedures that tailor which kinds of actions are received should be considered more important than those that only tailor the details of one kind of action.

Principle 6. Comparator interventions should be analysed and grouped using the same approach as experimental interventions.

The typology is of merit in its own right, not least to help commissioners, providers and practitioners have a terminology that is free of ambiguous terms (e.g. CGA, case management) but that focuses on key, active components. While this typology does not indicate which of these components and combinations are most effective, it illustrates the large number of different potential ways in which the independence of older people could be sustained. This could encourage commissioners, providers and clinicians to take a multifactorial approach to interventions rather than a narrow focus on a single component. The plain language descriptions may also facilitate co-designing services with members of the public. Future research could seek to co-develop a future iteration of the typology in order to suit the specific needs of commissioners, providers and clinicians.

Our typology poses challenges to researchers. The potential number of combinations of our 19 intervention components is huge. Very few of these combinations have been subject to research, and those that have been studied are often in only one study. It will not be feasible to provide a complete evidence base of multiple robust trials exploring every intervention combination. Yet we are confident that the typology is not based on micro-components and an unnecessary degree of division; each component is substantive and distinct. Our typology could help researchers to consider carefully, which combinations are the most critically important to evaluate. Our experience reinforces the need for better reporting of interventions, in particular control interventions and organisational aspects. It is vital that trialists report the health interventions intended for all participants in an arm, and we also suggest reporting the proportion of participants receiving each component of care, which may help better characterise multifactorial action, risk screening and available care interventions.

## Conclusions

We derived the CII-OP typology of 19 intervention components from trials of community-based complex interventions, which have been tested in 63 different combinations. Future guidelines on intervention reporting should explicitly include organisational aspects, which we found lacking. This typology contributes to shared conceptual clarity and can be used for intervention effectiveness syntheses, the interpretation of published literature and service planning and commissioning.

## Supplementary Material

aa-23-1134-File002_afae102

## Data Availability

The published articles that support the findings of this study are available from the relevant publishers. The coding structure, plain language descriptions and intervention group summaries are among the materials provided in the appendices. All other data requests should be submitted to the corresponding author for consideration.
