# Supplementary material for: Development of the Community-based complex Interventions to sustain Independence in Older People (CII-OP) typology: a qualitative synthesis of interventions in randomised controlled trials
Source: Age Ageing. 2024 May 25;53(5):afae102. doi: 10.1093/ageing/afae102 (PMC11127769; doi:10.1093/ageing/afae102)
Supplement: aa-23-1134-File002_afae102 [file aa-23-1134-file002_afae102.pdf]

Development of a typology of community-based complex interventions to sustain independence in older people (CII-OP): a qualitative synthesis of interventions in randomised controlled trials

APPENDICES

Contents

Appendix 1: Information sources, study selection process and electronic search strategies 3
Information sources 3
Study selection process 3
Electronic search strategies 3
Cochrane Central Register of Controlled Trials (CENTRAL) 3
MEDLINE 6
Embase 9
APA Psycinfo 12
CINAHL 15
Trial registers 21
Appendix 2: Coding structures 22
Original coding structure 22
Interim 29 studies 23
Final coding structure 34
Appendix 3: Codebook 43
Appendix 4: PRISMA flow diagram, included studies and reports 47
Summary characteristics of 129 included studies[1-129] 49
References to included studies 54
Appendix 5. Example intervention description for each of the TIDieR items 87
References (appendix 5) 89
Appendix 6: Plain language descriptions of the components and aspects of components used to determine intervention groups, organised by topic 90
Appendix 7: Intervention group summaries where group contains more than one intervention .. 95
Group: ADL 95
Group: ADL, aids, education, exercise, multifactorial-action and review with medication review and self-management 96
Group: Aids 98
Group: Available care 100

Group: Education ... 103
Group: Education, exercise, multifactorial-action and review with medication review and self-management strategies ... 104
Group: Education, multifactorial-action and review with medication review ... 106
Group: Education, multifactorial-action and review with medication review and self-management strategies ... 108
Group: Education, multifactorial-action and review with self-management strategies ... 110
Group: Exercise ... 111
Group: Exercise and psychology ... 113
Group: Homecare ... 115
Group: Homecare and multifactorial-action ... 116
Group: Home care, ADL, multifactorial-action from care-planning and review with self-management strategies ... 118
Group: Homecare, multifactorial-action and review ... 119
Group: Homecare, multifactorial-action from care-planning and review with medication review ... 121
Group: Homecare, multifactorial-action and review with self-management strategies ... 123
Group: Meaningful activities and education ... 125
Group: Multifactorial-action ... 126
Group: Multifactorial-action and review ... 129
Group: Multifactorial-action and review with medication review ... 131
Group: Multifactorial-action and review with medication review and self-management strategies ... 134
Group: Multifactorial-action and review with self-management strategies ... 136
Group: Multifactorial-action with medication review ... 138
Group: Nutrition and exercise ... 139
Group: Risk-screening ... 141
References (appendix 7) ... 143

Appendix 1: Information sources, study selection process and electronic search strategies

Information sources

We searched the following databases and trial registers from inception between the 9th and 11th of August 2021:

- Cochrane Central Register of Controlled Trials (CENTRAL) Wiley (1992-);
- MEDLINE Ovid (1946-);
- Embase and Embase Classic Ovid (1947-);
- CINAHL EBSCO (1972-);
- APA PsycINFO Ovid (1806-);
- US National Institutes of Health Ongoing Trials Register ClinicalTrials.gov (www.clinicaltrials.gov);
- World Health Organization International Clinical Trials Registry Platform (https://trialsearch.who.int).

We scanned the reference lists of included studies (backward citation searching) to identify additional eligible studies.

Study selection process

Two reviewers independently screened records (title and abstract). We retrieved reports (full-text) of studies identified as potentially eligible based on their record. Two reviewers, and then independently assessed eligibility and included all eligible studies and all of their reports. Disagreements were resolved by consensus between the reviewers, with guidance from the project management group if necessary.

Electronic search strategies

Cochrane Central Register of Controlled Trials (CENTRAL)

Cochrane Central Register of Controlled Trials (CENTRAL) via Wiley interface was searched. The database coverage was 1992 to present and the database was searched on the 11th of August 2021

#1 ((frail* or prefrailty)):ti,ab,kw (Word variations have been searched) 4037

#2 MeSH descriptor: [Aged] explode all trees 213642

#3 MeSH descriptor: [Geriatrics] this term only 207

#4 (elderly or old* next people* or old* next person* or old* next wom?n* or old* next m?n* or old* next male* or old* next female* or old* next adult* or old* next age* or aging or geriatric* or senior next citizen* or seniors or pensioner* or veteran* or sexagenarian* or septuagenarian* or octogenarian* or nonagenarian* or centenarian*):ti,ab,kw 92534

#5 ((over Near/2 ("60" or "61" or "62" or "63" or "64" or "65" or "66" or "67" or "68" or "69" or "70" or "71" or "72" or "73" or "74" or "75" or "76" or "77" or "78" or "79" or "80" or "81" or "82" or "83" or "84" or "85" or "86" or "87" or "88" or "89" or "90" or "91" or "92" or "93" or "94" or "95" or "96" or "97" or "98" or "99" or "100") Near years)):ti,ab,kw (Word variations have been searched) 3277

#6 {or #1-#5} 283983

#7 MeSH descriptor: [Independent Living] this term only 544

#8 MeSH descriptor: [Community Health Nursing] explode all trees 345

#9 ("Community support services"):ti,ab,kw (Word variations have been searched) 23

#10 MeSH descriptor: [Managed Care Programs] explode all trees 502

#11 ("health maintenance organization*" or "health maintenance organisation*"):ti,ab,kw (Word variations have been searched) 627

#12 (HMO*):ti,ab,kw (Word variations have been searched) 494

#13 MeSH descriptor: [Social Work] this term only 184

#14 (social Near/3 services):ti,ab,kw (Word variations have been searched) 1417

#15 ("Voluntary services"):ti,ab,kw (Word variations have been searched) 14

#16 MeSH descriptor: [Home Nursing] this term only 282

#17 ("house call*"):ti,ab,kw (Word variations have been searched) 583

#18 (home near/5 visit*):ti,ab,kw (Word variations have been searched) 5140

#19 ((("general practice" or "primary care" or nurse* or group or "ambulatory clinic" or "geriatric clinic") near/3 visit*)):ti,ab,kw (Word variations have been searched) 4731

#20 MeSH descriptor: [Geriatric Assessment] this term only 1509

#21 (pharmac* near/2 visit):ti,ab,kw (Word variations have been searched) 278

#22 ((home or house) near/2 appointment*):ti,ab,kw (Word variations have been searched) 24

#23 ("Home Care Services"):ti,ab,kw (Word variations have been searched) 2257

#24 MeSH descriptor: [Home Care Services] this term only 1883

#25 MeSH descriptor: [Health Services for the Aged] this term only 456

#26 MeSH descriptor: [Home Health Nursing] explode all trees 7

#27 ("district nursing"):ti,ab,kw (Word variations have been searched) 115

#28 ("health visit*"):ti,ab,kw (Word variations have been searched) 186

#29 ("community matron"):ti,ab,kw (Word variations have been searched) 4

#30 (home Near/3 (intervention* or support* or assessment*)):ti,ab,kw (Word variations have been searched) 4926

#31 MeSH descriptor: [Home Health Nursing] this term only 7

#32 (((preventive* or preventative*) near/5 medicine)):ti,ab,kw (Word variations have been searched) 781

#33 MeSH descriptor: [Preventive Medicine] this term only 121

#34 ((preventive* or preventative*) near/3 (program* or intervent* or support* or care or service* or approach* or "case management" or measure* or OT or "occupational therapy" or assess*)):ti,ab,kw (Word variations have been searched) 6275

#35 {or #7-#34} 25735

#36 MeSH descriptor: [Geriatric Nursing] this term only 178

#37 ("geriatric nursing"):ti,ab,kw (Word variations have been searched) 274

#38 {or #36-#37} 274

#39 MeSH descriptor: [Community Health Services] this term only 1061

#40 (community):ti,ab,kw 46478

#41 MeSH descriptor: [Community Health Nursing] explode all trees 345

#42 MeSH descriptor: [Community Pharmacy Services] this term only 271

#43 MeSH descriptor: [Home Care Services] this term only 1883

#44 MeSH descriptor: [Aftercare] this term only 661

#45 MeSH descriptor: [Primary Health Care] this term only 4388

#46 (domiciliary or ("social support" and home*) or ((homecare or medical) near/2 home) or (home and package*) or (outreach and home) or "(alternative setting" and home) or "home visit*" or "home manag*" or homecare or "home care" or "home therap*" or (model* adj1 home*) or "home program*" or "home monitor*"):ti,ab,kw (Word variations have been searched) 12652

#47 ("home-based" or homebased or homebound):ti,ab,kw (Word variations have been searched) 7510

#48 ((live or living or lived or dwell*) near/5 ("at home" or "own home" or "in home" or alone or independent*)):ti,ab,kw (Word variations have been searched)3855

#49 ("Home care" or "primary care" or "primary healthcare" or "primary health care" or "community dwelling"):ti,ab,kw (Word variations have been searched) 31085

#50 {or #39-#49} 80654

#51 #38 AND #50 103

#52 #35 or #51 25779

#53 #6 and #52 8010

#54 (coronary heart disease or CHD or chronic obstructive pulmonary disease or COPD or kidney failure or CKD or Heart failure or diabetes or asthma or cancer or schizophrenia or severe mental illness*):ti 210929

#55 #53 NOT #54 7003

MEDLINE

MEDLINE(R) ALL was searched via OvidSP. The database coverage was 1946 to present and the database was searched on 9th of August 2021

1 randomized controlled trial.pt. (539556)

2 controlled clinical trial.pt. (94320)

3 randomized.ab. (529280)

4 placebo.ab. (220248)

5 clinical trials as topic.sh. (196870)

6 randomly.ab. (363058)

7 trial.ti. (244962)

8 or/1-7 (1384889)

9 exp animals/ not humans.sh. (4870600)

10 8 not 9 [Cochrane Highly Sensitive Search Strategy for identifying randomized trials in MEDLINE: sensitivity- and precision-maximizing version (2008 revision] (1274483)

11 Clinical Trial, Phase III/ (18797)

12 ("phase 3" or "phase3" or "phase III" or "P3" or "PIII").ti,ab,kw. (73139)

13 11 or 12 [search filter for phase three trials to supplement Cochrane HSSS, Cooper 2019] (79735)

14 10 or 13 [final RCT filter] (1318490)

15 (frail* or prefrailty).tw. (25865)

16 exp aged/ (3283911)

17 geriatrics/ (30590)

18 (elder* or older or old people* or old person* or old wom#n*1 or old m#n*1 or old male*1 or old female*1 or old adult*1 or old age* or aging or ageing or geriatric* or senior citizen* or seniors or pensioner* or veteran* or sexagenarian* or septuagenarian* or octogenarian* or nonagenarian* or centenarian*).tw,kf. (1385083)

19 (over adj2 ("60" or "61" or "62" or "63" or "64" or "65" or "66" or "67" or "68" or "69" or "70" or "71" or "72" or "73" or "74" or "75" or "76" or "77" or "78" or "79" or "80" or "81" or "82" or "83" or "84" or "85" or "86" or "87" or "88" or "89" or "90" or "91" or "92" or "93" or "94" or "95" or "96" or "97" or "98" or "99" or "100") adj years).tw. (21451)

20 or/15-19 [older or frail people] (4132930)

21 independent living/ (8001)

22 community health services/ (32391)

23 community health nursing/ (19684)

24 Community support services.tw. (173)

25 exp managed care programs/ (40081)

26 (health maintenance organi?ation* or HMO*).tw. (13817)

27 (Social adj3 services).tw. (10694)

28 Voluntary services.tw. (99)

29 *home nursing/ (5361)

30 House Calls/ (3846)

31 house call*.tw. (656)

32 (home adj5 visit*).tw. (12399)

33 ((general practice or primary care or nurse* or group or ambulatory clinic or geriatric clinic) adj3 visit*).tw. (9527)

34 *geriatric assessment/ (13906)

35 (pharmac* adj2 visit).tw. (212)

36 ((home or house) adj2 appointment*).tw. (52)

37 Home Care Services/ (34738)

38 Home care service*.tw. (1913)

39 *health services for the aged/ (14001)

40 home health nursing/ (364)

41 district nursing.tw. (667)

42 health visit*.ti. or health visit*.ab. /freq=2 (2285)

43 community matron*.ti. or community matron*.ab. /freq=2 (83)

44 (home adj3 (intervention* or support* or assessment*)).tw. (8887)

45 preventive health services/ (14024)

46 ((preventive* or preventative*) adj5 medicine).tw. (7306)

47 preventative medicine/ (11938)

48 ((preventive* or preventative*) adj3 (program* or intervent* or support* or care or service* or approach* or case management or measure* or OT or occupational therapy or assess*)).tw. (66507)

49 or/21-48 (283985)

50 geriatric nursing/ (13707)

51 geriatric nurs*.tw,kf. (1164)

52 or/50-51 [geriatric nursing] (14118)

53 community.ti,ab,kf. (539116)

54 community health services/ or community health nursing/ or community mental health services/ or community pharmacy services/ (74241)

55 "domiciliary care"/ (34738)

56 aftercare/ (10404)

57 primary health care/ (83064)

58 (domiciliary or (social support and home*) or ((homecare or medical) adj2 home) or (home and package*) or (outreach and home) or (alternative setting and home) or home visit* or home manag* or homecare or home care or home therap* or (model* adj1 home*) or home program* or home monitor*).tw. (58982)

59 ((live or living or lived or dwell*) adj5 ("at home" or "own home" or "in home" or alone or independent*)).tw. (17479)

60 (home-based or homebased or homebound).tw. (12811)

61 (Home care or primary care or primary health care or primary healthcare).tw. (163820)

62 or/53-61 [interventions in a community or home setting] (808717)

63 52 and 62 [geriatric nursing and interventions in a community or home setting] (2015)

64 49 or 63 [all interventions] (284694)

65 (coronary heart disease or CHD or chronic obstructive pulmonary disease or COPD or kidney failure or CKD or Heart failure or diabetes or asthma or cancer or schizophrenia or severe mental illness*).ti. (1592860)

66 64 not 65 [all interventions excluding specific diseases in title] (267883)

67 14 and 20 and 66 [RCTS and older people and interventions] (7005)

Embase

Embase and Embase Classic via OvidSP was searched. The database coverage was 1947 to present and the database was search on the 9th of August 2021

1 randomized controlled trial/ (672319)

2 controlled clinical study/ (463974)

3 1 or 2 (860531)

4 random*.tw. (1703521)

5 randomization/ (91766)

6 intermethod comparison/ (273924)

7 placebo.tw. (332206)

8 (compare or compared or comparison).ti. (574408)

9 ((evaluated or evaluate or evaluating or assessed or assess) and (compare or compared)).ab. (2067060)

10 (open adj label).ti,ab. (89661)

11 ((double or single or doubly or singly) adj blind).tw. (227285)

12 parallel group$1.tw. (27916)

13 double blind procedure/ (188870)

14 (crossover or cross over).tw. (113362)

15 ((assign* or match or matched or allocation) adj5 (alternate or group$1 or intervention$1 or patient$1 or subject$1 or participant$1)).tw. (362240)

16 (assigned or allocated).tw. (427304)

17 (controlled adj7 (study or design or trial)).tw. (388612)

18 (volunteer or volunteers).tw. (265628)

19 human experiment/ (551078)

20 trial.ti. (343846)

21 or/4-20 (5189451)

22 21 or 3 (5347546)

23 (random* adj sampl* adj7 ("cross section*" or questionnaire$1 or survey* or database$1)).tw. not (comparative study/ or controlled study/ or randomi?ed controlled.tw. or randomly assigned.tw.) (8774)

24 Cross-sectional study/ not (randomized controlled trial/ or controlled clinical study/ or controlled study/ or randomi?ed controlled.tw. or control group$1.tw.) (277846)

25 (((case adj control*) and random*) not randomi?ed controlled).tw. (18755)

26 (Systematic review not (trial or study)).ti. (182362)

27 (nonrandom* not random*).tw. (17268)

28 "Random field*".tw. (2544)

29 (random cluster adj3 sampl*).tw. (1374)

30 (review.ab. and review.pt.) not trial.ti. (913087)

31 "we searched".ab. and (review.ti. or review.pt.) (37761)

32 "update review".ab. (119)

33 (databases adj4 searched).ab. (44421)

34 (rat or rats or mouse or mice or swine or porcine or murine or sheep or lambs or pigs or piglets or rabbit or rabbits or cat or cats or dog or dogs or cattle or bovine or monkey or monkeys or trout or marmoset$1).ti. and animal experiment/ (1116446)

35 Animal experiment/ not (human experiment/ or human/) (2346095)

36 or/23-35 (3759577)

37 22 not 36 [Cochrane Highly Sensitive Search Strategy for identifying controlled trials in Embase: (2018 revision); Ovid format (Glanville *et al.*, 2019b)] (4755948)

38 (frail* or prefrailty).tw. (39809)

39 aged/ (3370037)

40 very elderly/ (236950)

41 frail elderly/ (10922)

42 geriatrics/ (39915)

43 (elder* or older or old pele*ople* or old person* or old wom#n*1 or old m#n*1 or old ma1 or old female*1 or old adult*1 or old age* or aging or ageing or geriatric* or senior citizen* or seniors or pensioner* or veteran* or sexagenarian* or septuagenarian* or octogenarian* or nonagenarian* or centenarian*).tw,kw. (1838159)

44 (over adj2 ("60" or "61" or "62" or "63" or "64" or "65" or "66" or "67" or "68" or "69" or "70" or "71" or "72" or "73" or "74" or "75" or "76" or "77" or "78" or "79" or "80" or "81" or "82" or "83" or "84" or "85" or "86" or "87" or "88" or "89" or "90" or "91" or "92" or "93" or "94" or "95" or "96" or "97" or "98" or "99" or "100") adj years).tw. (33789)

45 or/38-44 [frail or elderly people] (4516350)

46 independent living/ (5523)

47 community care/ (61677)

48 community health nursing/ (26723)

49 Community support services.tw. (239)

50 (health maintenance organi?ation* or HMO*).tw. (16728)

51 (Social adj3 services).tw. (14016)

52 Voluntary services.tw. (148)

53 home visit/ (3712)

54 house call*.tw. (852)

55 (home adj5 visit*).tw. (17275)

56 ((general practice or primary care or nurse* or group or ambulatory clinic or geriatric clinic) adj3 visit*).tw. (14158)

57 *geriatric assessment/ (6239)

58 (pharmac* adj2 visit).tw. (504)

59 ((home or house) adj2 appointment*).tw. (107)

60 Home Care/ (66345)

61 Home care service*.tw. (2345)

62 *elderly care/ (21267)

63 district nursing.tw. (664)

64 health visit*.ti. or health visit*.ab. /freq=2 (2402)

65 community matron*.ti. or community matron*.ab. /freq=2 (82)

66 (home adj3 (intervention* or support* or assessment*)).tw. (12351)

67 preventive health service/ (30244)

68 ((preventive* or preventative*) adj5 medicine).tw. (12282)

69 preventive medicine/ (29022)

70 ((preventive* or preventative*) adj3 (program* or intervent* or support* or care or service* or approach* or case management or measure* or OT or occupational therapy or assess*)).tw. (88643)

71 or/46-70 [specific interventions] (376111)

72 geriatric nursing/ (12986)

73 geriatric nurs*.tw,kw. (1405)

74 or/72-73 [geriatric nursing] (13603)

75 community.tw,kw. (686753)

76 community health services/ or community health nursing/ or mental health service/ or "pharmacy (shop)"/ (144914)

77 aftercare/ (8598)

78 primary health care/ (70765)

79 (domiciliary or (social support and home*) or ((homecare or medical) adj2 home) or (home and package*) or (outreach and home) or (alternative setting and home) or home visit* or home manag* or homecare or home care or home therap* or (model* adj1 home*) or home program* or home monitor*).tw. (78824)

80 ((live or living or lived or dwell*) adj5 ("at home" or "own home" or "in home" or alone or independent*)).tw. (24399)

81 (home-based or homebased or homebound).tw. (17773)

82 (Home care or primary care or primary healthcare or primary health care).tw. (217034)

83 or/75-82 [home or community setting] (1060004)

84 74 and 83 [geriatric nursing and home or community setting] (1864)

85 71 or 84 [all interventions] (376832)

86 (coronary heart disease or CHD or chronic obstructive pulmonary disease or COPD or kidney failure or CKD or Heart failure or diabetes or asthma or cancer or schizophrenia or severe mental illness*).ti. (2270105)

87 85 not 86 [all interventions except those mentioning specific diseases] (350036)

88 37 and 45 and 87 [RCT and elderly and Interventions] (17333)

APA Psycinfo

APA Psycinfo via OvidSP was searched. The database coverage was 1806 to present and the database was searched on the 9th of August 2021

1 (control: or random:).tw. or exp treatment/ [sensitive rct psycinfo search strategy Eady *et al.*, 2009] (1743140)

2 (frail* or prefrailty).tw. (5244)

3 exp aging/ (79898)

4 geriatric patients/ (13753)

5 geriatrics/ (11969)

6 (elder* or older or old people* or old person* or old wom#n*1 or old m#n*1 or old male*1 or old female*1 or old adult*1 or old age* or aging or geriatric* or senior citizen* or seniors or pensioner* or veteran* or sexagenarian* or septuagenarian* or octogenarian* or nonagenarian* or centenarian*).tw. (355903)

7 (over adj2 ("60" or "61" or "62" or "63" or "64" or "65" or "66" or "67" or "68" or "69" or "70" or "71" or "72" or "73" or "74" or "75" or "76" or "77" or "78" or "79" or "80" or "81" or "82" or "83" or "84" or "85" or "86" or "87" or "88" or "89" or "90" or "91" or "92" or "93" or "94" or "95" or "96" or "97" or "98" or "99" or "100") adj years).tw. (2391)

8 or/2-7 [frail or elderly people] (371975)

9 Self-Care Skills/ (4756)

10 community health/ (3653)

11 community services/ (17234)

12 social services/ (9557)

13 Community support services.tw. (219)

14 exp managed care/ (4567)

15 (health maintenance organi?ation* or HMO*).tw. (2449)

16 (Social adj3 services).tw. (11772)

17 Voluntary services.tw. (71)

18 home visiting programs/ (1861)

19 home care/ (6905)

20 house call*.tw. (106)

21 (home adj5 visit*).tw. (5619)

22 ((general practice or primary care or nurse* or group or ambulatory clinic or geriatric clinic) adj3 visit*).tw. (2716)

23 (pharmac* adj2 visit).tw. (23)

24 ((home or house) adj2 appointment*).tw. (12)

25 Independent Living Programs/ (408)

26 Home care service*.tw. (706)

27 district nursing.tw. (64)

28 health visit*.ti. or health visit*.ab. /freq=2 (342)

29 community matron*.ti. or community matron*.ab. /freq=2 (14)

30 (home adj3 (intervention* or support* or assessment*)).tw. (5172)

31 ((preventive* or preventative*) adj5 medicine).tw. (1085)

32 preventive medicine/ (2464)

33 ((preventive* or preventative*) adj3 (program* or intervent* or support* or care or service* or approach* or case management or measure* or OT or occupational therapy or assess*)).tw. (17107)

34 or/9-33 [interventions] (83270)

35 geriatric nursing.tw. (252)

36 (geriatrics/ or geriatric patients/) and nursing/ (639)

37 or/35-36 [geriatric nursing] (833)

38 community.tw. (275605)

39 community services/ or community health/ or community mental health services/ or pharmacy/ (28713)

40 (community healthcare or community health care).tw. (588)

41 home care/ (6905)

42 aftercare/ (1121)

43 primary health care/ (19284)

44 Public Health Service Nurses/ (658)

45 (domiciliary or (social support and home*) or ((homecare or medical) adj2 home) or (home and package*) or (outreach and home) or (alternative setting and home) or home visit* or home manag* or homecare or home care or home therap* or (model* adj1 home*) or home program* or home monitor*).tw. (19096)

46 ((live or living or lived or dwell*) adj5 ("at home" or "own home" or "in home" or alone or independent*)).tw. (10597)

47 (home-based or homebased or homebound).tw. (5823)

48 (Home care or primary care or primary health care or primary healthcare).tw. (44178)

49 or/38-48 [community or home based] (340141)

50 37 and 49 [geriatric nursing and community or home based] (174)

51 34 or 50 [all interventions] (83378)

52 (coronary heart disease or CHD or chronic obstructive pulmonary disease or COPD or kidney failure or CKD or Heart failure or diabetes or asthma or cancer or schizophrenia or severe mental illness*).ti. (116600)

53 51 not 52 [all interventions except specific diseases in title] (79888)

54 1 and 8 and 53 [RCT filter and elderly and all interventions except specific diseases in title] (7917)

CINAHL

CINAHL via EBSCO interface was searched. The database coverage was 1972 to present and the database was searched on the 9th of August 2021

#	Query	Limiters/Expanders	Last Run Via	Results
S46	S10 AND S18 and S45	Search modes - Boolean/Phrase	Interface - EBSCOhost Research Databases Search Screen - Advanced Search Database - CINAHL	10,636
S45	S43 NOT S44	Search modes - Boolean/Phrase	Interface - EBSCOhost Research Databases Search Screen - Advanced Search Database - CINAHL	106,016
S44	TI ("coronary heart disease" or CHD or "chronic obstructive pulmonary disease" or COPD or "kidney failure" or CKD or "Heart failure" or diabetes or asthma or cancer or schizophrenia or "severe mental illness*")	Search modes - Boolean/Phrase	Interface - EBSCOhost Research Databases Search Screen - Advanced Search Database - CINAHL	501,973
S43	S29 or S42	Search modes - Boolean/Phrase	Interface - EBSCOhost Research Databases Search Screen -	113,278

			Advanced Search Database - CINAHL	
S42	S30 and S41	Search modes - Boolean/Phrase	Interface - EBSCOhost Research Databases Search Screen - Advanced Search Database - CINAHL	2,217
S41	S31 OR S32 OR S33 OR S34 OR S35 OR S36 OR S37 OR S38 OR S39 OR S40	Search modes - Boolean/Phrase	Interface - EBSCOhost Research Databases Search Screen - Advanced Search Database - CINAHL	687,262
S40	TX "Home care" or "primary care" or "primary health care" or "primary healthcare"	Search modes - Boolean/Phrase	Interface - EBSCOhost Research Databases Search Screen - Advanced Search Database - CINAHL	198,977
S39	TX "home-based" or homebased or homebound	Search modes - Boolean/Phrase	Interface - EBSCOhost Research Databases Search Screen - Advanced Search Database - CINAHL	9,035
S38	(MH "Community Health Services")	Search modes - Boolean/Phrase	Interface - EBSCOhost Research Databases Search Screen - Advanced Search Database - CINAHL	22,541
S37	TX ((live or living or lived or dwell*) N5 ("at home" or "own home" or "in home" or community or alone or independent*)	Search modes - Boolean/Phrase	Interface - EBSCOhost Research Databases Search Screen - Advanced Search Database - CINAHL	57,710
S36	TX domiciliary or ("social support" and home*) or ((homecare or medical) N2 home) or (home and package*) or (outreach and home) or (alternative setting and home) or home visit* or home manag* or homecare or "home care" or "home therap*" or (model* N1 home*) or "home program*" or "home monitor*")	Search modes - Boolean/Phrase	Interface - EBSCOhost Research Databases Search Screen - Advanced Search Database - CINAHL	68,720
S35	(MH "Primary Health Care")	Search modes - Boolean/Phrase	Interface - EBSCOhost Research Databases Search Screen -	67,490

			Advanced Search Database - CINAHL	
S34	(MH "After Care")	Search modes - Boolean/Phrase	Interface - EBSCOhost Research Databases Search Screen - Advanced Search Database - CINAHL	16,366
S33	(MH "Community Health Nursing")	Search modes - Boolean/Phrase	Interface - EBSCOhost Research Databases Search Screen - Advanced Search Database - CINAHL	28,024
S32	(MH "Community Mental Health Services")	Search modes - Boolean/Phrase	Interface - EBSCOhost Research Databases Search Screen - Advanced Search Database - CINAHL	9,964
S31	TX community	Search modes - Boolean/Phrase	Interface - EBSCOhost Research Databases Search Screen - Advanced Search Database - CINAHL	479,269
S30	(MH "Gerontologic Nursing")	Search modes - Boolean/Phrase	Interface - EBSCOhost Research Databases Search Screen - Advanced Search Database - CINAHL	13,362
S29	S19 OR S20 OR S21 OR S22 OR S23 OR S24 OR S25 OR S26 OR S27 OR S28	Search modes - Boolean/Phrase	Interface - EBSCOhost Research Databases Search Screen - Advanced Search Database - CINAHL	111,533
S28	TX ((preventive* or preventative*) N3 (program* or intervent* or support* or care or service* or approach* or case management or measure* or OT or "occupational therapy" or assess*))	Search modes - Boolean/Phrase	Interface - EBSCOhost Research Databases Search Screen - Advanced Search Database - CINAHL	47,492
S27	(MH "Preventive Health Care")	Search modes - Boolean/Phrase	Interface - EBSCOhost Research Databases Search Screen - Advanced Search Database - CINAHL	21,369

S26	TX (home N3 (intervention* or support* or assessment*))	Search modes - Boolean/Phrase	Interface - EBSCOhost Research Databases Search Screen - Advanced Search Database - CINAHL	10,880
S25	TX "community matron*"	Search modes - Boolean/Phrase	Interface - EBSCOhost Research Databases Search Screen - Advanced Search Database - CINAHL	283
S24	TX "health visit*"	Search modes - Boolean/Phrase	Interface - EBSCOhost Research Databases Search Screen - Advanced Search Database - CINAHL	8,986
S23	TX "district nursing"	Search modes - Boolean/Phrase	Interface - EBSCOhost Research Databases Search Screen - Advanced Search Database - CINAHL	2,176
S22	MM "Home Health Care"	Search modes - Boolean/Phrase	Interface - EBSCOhost Research Databases Search Screen - Advanced Search Database - CINAHL	17,073
S21	(MH "Health Services for the Aged")	Search modes - Boolean/Phrase	Interface - EBSCOhost Research Databases Search Screen - Advanced Search Database - CINAHL	6,819
S20	(MH "Home Visits") or (MH "Community Living")	Search modes - Boolean/Phrase	Interface - EBSCOhost Research Databases Search Screen - Advanced Search Database - CINAHL	23,215
S19	TX "Community support services"	Search modes - Boolean/Phrase	Interface - EBSCOhost Research Databases Search Screen - Advanced Search Database - CINAHL	155
S18	S11 OR S12 OR S13 OR S14 OR S15 OR S16 OR S17	Search modes - Boolean/Phrase	Interface - EBSCOhost Research Databases Search Screen - Advanced Search Database - CINAHL	1,209,432
S17	TX (over N2 ("60" or "61" or "62" or "63" or "64" or "65" or "66" or "67" or "68" or "69" or "70" or	Search modes - Boolean/Phrase	Interface - EBSCOhost Research Databases Search Screen -	7,999

	"71" or "72" or "73" or "74" or "75" or "76" or "77" or "78" or "79" or "80" or "81" or "82" or "83" or "84" or "85" or "86" or "87" or "88" or "89" or "90" or "91" or "92" or "93" or "94" or "95" or "96" or "97" or "98" or "99" or "100") N1 years)		Advanced Search Database - CINAHL	
S16	TX (aging or ageing or geriatric* or gerontologic* or elderly or "senior citizen*" or seniors or pensioner* or veteran* or sexagenarian* or septuagenarian* or octogenarian* or nonagenarian* or centenarian*)	Search modes - Boolean/Phrase	Interface - EBSCOhost Research Databases Search Screen - Advanced Search Database - CINAHL	523,198
S15	TX ((older or elder*) N2 (person or people or adult* or patient* or m?n* or wom?n* or female* or male*))	Search modes - Boolean/Phrase	Interface - EBSCOhost Research Databases Search Screen - Advanced Search Database - CINAHL	194,690
S14	(MH "Aged+")	Search modes - Boolean/Phrase	Interface - EBSCOhost Research Databases Search Screen - Advanced Search Database - CINAHL	878,186
S13	(MH "Geriatrics")	Search modes - Boolean/Phrase	Interface - EBSCOhost Research Databases Search Screen - Advanced Search Database - CINAHL	5,708
S12	TX (frail*)	Search modes - Boolean/Phrase	Interface - EBSCOhost Research Databases Search Screen - Advanced Search Database - CINAHL	18,976
S11	TX (prefrailty)	Search modes - Boolean/Phrase	Interface - EBSCOhost Research Databases Search Screen - Advanced Search Database - CINAHL	160
S10	S1 OR S2 OR S3 OR S4 OR S5 OR S6 OR S7 OR S8 OR S9	Search modes - Boolean/Phrase	Interface - EBSCOhost Research Databases Search Screen -	1,157,497

			Advanced Search<br>Database - CINAHL	
S9	AB group or AB groups	Search modes - Boolean/Phrase	Interface - EBSCOhost Research Databases<br>Search Screen - Advanced Search<br>Database - CINAHL	795,542
S8	AB trial or AB Trials	Search modes - Boolean/Phrase	Interface - EBSCOhost Research Databases<br>Search Screen - Advanced Search<br>Database - CINAHL	299,002
S7	AB randomly	Search modes - Boolean/Phrase	Interface - EBSCOhost Research Databases<br>Search Screen - Advanced Search<br>Database - CINAHL	96,217
S6	AB (randomised or randomized)	Search modes - Boolean/Phrase	Interface - EBSCOhost Research Databases<br>Search Screen - Advanced Search<br>Database - CINAHL	221,475
S5	TX "randomised controlled trial*"	Search modes - Boolean/Phrase	Interface - EBSCOhost Research Databases<br>Search Screen - Advanced Search<br>Database - CINAHL	26,900
S4	TX "controlled clinical trial*"	Search modes - Boolean/Phrase	Interface - EBSCOhost Research Databases<br>Search Screen - Advanced Search<br>Database - CINAHL	10,403
S3	(MH "Clinical Trials")	Search modes - Boolean/Phrase	Interface - EBSCOhost Research Databases<br>Search Screen - Advanced Search<br>Database - CINAHL	177,904
S2	(MH "Randomized Controlled Trials")	Search modes - Boolean/Phrase	Interface - EBSCOhost Research Databases<br>Search Screen - Advanced Search<br>Database - CINAHL	117,892
S1	TX "randomized controlled trial*"	Search modes - Boolean/Phrase	Interface - EBSCOhost Research Databases<br>Search Screen - Advanced Search<br>Database - CINAHL	

Trial registers

To search Clinicaltrials.gov we used the advanced search interface, and searched the Conditions or Disease field using the following search terms: Frail Elderly Syndrome, frailty syndrome, Age-Related Atrophy, Frailty, Old Age, Debility. The search yielded 861 records.

For the International Clinical Trials Registry Platform (ICTRP) we used the advanced search interface, and used the search syntax older or elderly or frail in Title field and community or complex or independent or independence in Intervention field (with synonyms, all recruitment status).The search resulted in 425 records.

Appendix 2: Coding structures

Original coding structure

Name	Files	References
01. Brief name	0	0
02. Why	0	0
Goal	0	0
Rationale or theory	0	0
03. What_ Materials	0	0
a. training and implementation materials	0	0
b. provided materials	0	0
c. materials used as part of delivery (not provided to participants)	0	0
d. other materials	0	0
04. What_ Procedures	0	0
a. implementing	0	0
b. providing- assessing and planning	0	0
c. providing- intervening	0	0
d. resulting	0	0
e. supporting	0	0
05. Who provided	0	0
05.1 Number of intervention providers	0	0
5.1.1. Unidisciplinary	0	0
5.1.2. Multidisciplinary	0	0
05.2 Disciplinary background	0	0
Care coordinator	0	0
Dietitian	0	0
Doctor (GP)	0	0
Doctor (secondary or tertirary)	0	0
Health visitor	0	0
Nurse	0	0
Occupational therapist	0	0
Personal care assistant	0	0
Pharmacist	0	0
Physiotherapist	0	0
Podiatrist	0	0
Support staff	0	0
zOther	0	0
05.3 Other details	0	0
06. How	0	0
06.1. Individual-Group (size)	0	0
06.2. Medium	0	0

06.2.1. face to face	0	0
06.2.2. distance (synchronous)	0	0
06.2.3. asynchronous	0	0
06.3. Interactivity	0	0
07. Where	0	0
07.1. Intervention location	0	0
07.1.1. Home	0	0
07.1.2. Other	0	0
07.2. Equipment+facilities	0	0
07.3. Country+infrastructure	0	0
08. When and how much	0	0
08.1. When started	0	0
08.2. Duration of sessions	0	0
08.3. Number of sessions	0	0
08.4. Session's schedule	0	0
08.4.1. Fixed	0	0
08.4.2. Varied	0	0
08.5. Intensity or dose (other)	0	0
09. Tailoring	0	0
10. Modifications	0	0
11. How well (planned)	0	0
12. How well (actual)	0	0

Interim 29 studies

Name	Files	References
01. Brief name	42	127
02. Why	47	278
Goal	45	129
Rationale or theory	39	143
03. What_ Materials	35	167
a. training and implementation materials	11	18
b. provided materials	24	65
Dietary supplements	1	1
Equipment to aid ADLs	1	2
Info on available services and support	2	5
Newsletter	1	1
Participant's record of compliance	1	2
c. materials used as part of delivery (not provided to participants)	26	65
d. other materials	12	19
04. What_ Procedures	52	585
a. implementing (enabling)	26	66
Adapting the intervention to suit the target participants	1	7

Arranging funding structure	2	3
arranging provision of sessions and workshops by other organisations	2	6
Arranging sharing of medical information	1	1
Chaperoning	1	1
deliverer recruitment	6	9
Recruiting healthcare professionals of other disciplinaries	1	2
Staff recruitment	1	1
deliverer training	17	22
deliverer training	2	2
Health service provides training to intervention-delivery staff	1	1
including the training of nurses	1	1
staff training	1	1
Staff training (2)	1	2
training	1	1
Training physiotherapist to deliver intervention	2	3
Training the staff	4	5
Developing community partnerships	3	8
Developing nutritional information	1	1
Developing processes and guidelines within multidisciplinary team	2	3
Translating intervention materials	2	2
a.b. screening	2	2
Referral to physiotherapist (after screening)	2	2
b. providing- assessing and planning	37	161
Environmental-functional (OT-aids-adaptations) assessment and planning	6	10
Home telecare assessment and evaluation	1	1
Occupational therapy assessment and planning	1	1
OT need assessment	2	5
Functional activity assessment and planning	3	8
Care goal discussion	2	3
Deciding the daily activities to train jointly (physio and older person)	1	1
Functional clinical assessment	2	3
Testing daily functioning with diagnostic tools	1	1
ICT needs assessment and planning	1	3
Conducting ICT needs assessment and orientation session	1	3
Medication assessment and planning	7	13
Medication Management reviewing	3	4
Medication Reviewing	1	5
Multidomain assessment without planning and arranging	1	1
Multidomain assessment, planning and arranging	23	113
Arranging services (single central needs assessor)	2	3
Assessing needs (single central needs assessor)	2	3
Assessing patient through review of medical record and home visit	3	4
Case management (referring, coordinating)	1	8

Comprehensive assessment, planning and arranging	1	12
Comprehensive Initial Assessment	2	3
Conducting initial structured telephone interview assessment	1	6
Developing and managing goal-directed care plan	3	10
Developing care plan, liaising with care services and coordinating them	3	6
Discussion for Referrals (Nurse with participant and thier families)	1	1
Individualized Care Planning	4	6
Multimodal assessment, planning and arranging	1	1
Providing comprehensive geriatric assessment at clinic (selected cohort)	1	8
Undertaking comprehensive geriatric assessment (single local service coordinator)	3	8
Multidomain assessment, planning and arranging (selective)	1	1
Nutritional assessment and planning	1	2
Monitoring compliance with diet	1	1
Nutritional assessment based on food diary	1	1
Physical assessment and planning	1	2
Conducting maximum strength test	1	2
Social work assessment and planning	1	1
b.b. providing- reviewing and adjusting	24	57
Encouraging intervention participation including reviewing goals	6	7
encouragement contact	1	1
Encouraging participant to continue lifestyle changes	1	2
Regular mental health assessment and arranging crisis intervention	2	3
regularly reviewing and adjusting environmental support plan	1	3
regularly reviewing and adjusting multidomain care plan	12	36
Case management	1	12
CGA every 6 months	1	1
ongoing care management	2	3
ongoing telephone case management	2	9
regularly screening functional status	1	1
Reviewing care plan and discharging (one-off)	3	5
Case conference	2	2
Reviewing care plan and discharging to family care	1	3
c. providing- actioning	32	146
cognitive-behavioral therapy	3	8
Educating and training in health maintenance and self care	4	13
Educating and training in self-management skills including problem solving	6	9
Motivational interviewing	1	5
nutritional or dietary education or advice	3	7
Nutritional Education	1	2
Providing dietary advice (dietitian consultation)	1	3
Providing nutrition workshop	1	1
physical training (primarily)	17	44

Baduanjin training	1	4
Muscle Strength, balance and co-ordination exercise	0	0
Physical Fitness Training including mobility practice	1	3
Providing physical therapy and advice	1	1
Providing supervised progressive functional task exercise programme	3	12
Supervised progressive resistance exercise training focused on leg muscles	1	3
Supervised repetitive functional ADL training	3	4
Walking - exercise	0	0
Prepare for transition back to family care	1	1
Providing aids and adaptations (simple or mechanical)	1	4
Providing community OT support	1	4
Providing information (written) about caring for own health	1	1
Providing information (written) about nutrition or diet	2	2
Providing information about available services	1	1
Providing information and advice to facilitate independent living including trying equipment (ILC visit)	1	6
Providing juices regularly (freshly prepared fruit or vegetable)	1	1
Providing naturopathic and or homeopathic treatment	1	4
Providing personal care assistance	1	1
Providing protein-rich products	1	1
Providing technology for social participation	2	4
Providing telecare (safety monitoring and alerting)	1	4
Providing telecare (supporting everyday activities)	1	4
Providing telehealth (health monitoring and communication)	1	1
Social skills empowerment training	1	2
Training and support for technology-telehealth-telecare	2	7
Training for aids and adaptations (simple or mechanical)	1	1
c.o. providing- actioning selective	11	26
Depression-related support	2	2
Educating and training in health maintenance and self care	3	3
Encouraging access to alcoholism support	1	1
Providing aids and adaptations (simple or mechanical)	3	5
Providing alcoholism related info	1	1
Providing personal care assistance	1	1
Providing psychosocial support	1	1
Providing rehabilitation	1	1
Providing telecare (safety monitoring and alerting)	1	2
Providing telehealth (health monitoring and communication)	1	1
Substance cessation programs (including smoking and alcohol)	2	5
Teaching about medication use	2	2
prescribing medication	0	0
Training and support in technology-telehealth-telecare	1	1
d. resulting	15	34

Calling with queries and progress updates	1	1
completing 3-day food diary	1	1
Completing record of compliance	1	1
Conducting additional exercises at home and in further sessions	1	1
Following dietary recommendations including supplementation	1	2
Life style changing elements	1	1
Participant fitting equipments	1	1
Participating in care decisions	2	3
participating in peer support	1	1
Patients meeting care management goals	1	1
Patient's self-care and disease management	1	1
Performing regular physical activities	2	3
Personal coping strategies	1	1
practising exercise routinely	1	2
Pts recording in diary	1	2
Sharing experiences and learning with peers	1	1
Taking action based on assessment	1	1
Using assistive services and becoming proficient (implied)	1	4
working towards short-term goals on own	1	2
e. supporting	20	34
Arranging medium for peer support (setting up facebook group)	1	1
Caregiver support	1	1
case conference	0	0
Coordinators managing the support workers	2	3
Discussing problems in team meetings	2	2
Engaging interpreters in activities provision	1	1
geriatrician supervising case manager	1	1
Intervention delivery supervision	1	1
Keepig deliverers informed about progress	1	1
Multidisciplinary team communicating about patient's care	3	4
Nurse practitioners consulting and liaising with GPs	1	1
Providing health status info to staff	1	1
Refresh staff training	1	1
researchers supporting deliverers - debrief and planning	1	2
Staff sharing experiences - peer support	1	2
Supervision	1	3
Support from research team	1	2
Telephone helpline for participants to call about intervention related queries	1	1
Training case managers	1	2
Training for nurse practitioners (Work Shop)	1	1
transporting participants to a centre	1	1
f. available usual care	19	47
Accessing conventional care	2	3

Arranging care	1	2
Audiology	1	1
Geriatric medicine	1	1
Geriatric psychiatry	1	1
Geriatric urology	1	1
Home care	1	1
Home-based primary care	1	1
Information communication technology workshop	1	1
Mental health	1	1
Neuropsychology	1	1
Paid caregivers providing home care	1	4
primary health care	1	3
Prosthetics	1	1
Providing geriatric services	1	1
Receiving community services	3	3
Rehabilitation services	1	1
Social work	1	1
Urology	1	1
usual commmunity services	1	1
Vaccinations	1	3
g. attention control	2	3
Comprehensive assessment (control)	1	2
Paper-based fake sensors	1	1
Social questions	0	0
zOther	0	0
05. Who provided	40	203
05.1 Number of intervention providers	11	18
5.1.1. Unidisciplinary	3	4
01	7	11
02 per participant	1	1
1	1	2
5.1.2. Multidisciplinary	7	12
01+	1	2
01-02 (implied)	1	1
02	2	4
02+	2	4
03	5	7
03+	2	8
04	3	5
04 per participant - implied min number	2	3
04+	1	5
04+1	2	2
05	1	1

05+	3	13
1 or more (implied)	1	1
3 minimum	1	2
Variable	3	8
05.2 Disciplinary background	37	160
Care coordinator	4	6
Community health workers	1	1
Dietitian	5	5
Doctor (GP)	12	28
Doctor (secondary or tertirary)	3	5
Exercise trainers	1	1
Health visitor	0	0
Nurse	21	47
Occupational therapist	9	10
Personal care assistant	1	1
Pharmacist	4	6
Physiotherapist	12	12
Podiatrist	0	0
Support staff	5	5
zOther	21	33
dental hygienists	1	1
Homeopatic study physicians	1	1
Naturopath	1	1
Nurse OR community nurse	0	0
Nurse practitioner	2	2
OR Social worker	1	4
Paramedic	1	1
Social Worker	1	2
specialists	1	1
Sport therapist	1	1
05.3 Other details	20	25
06. How	40	262
06.1. Individual-Group (size)	33	77
01 (Individual)	26	44
02 (patient-carer dyad)	1	3
02-05	2	3
04-06 (group of older adults)	1	1
04-07 (group of older adults)	2	2
06-08 (group of older adults)	1	4
08-10 (group of older adults)	1	1
10-15	1	1
11 max (group of older adults)	1	1
Group (size not specified)	3	7

06.2. Medium	32	146
06.2.1. face to face	31	100
consultation [implied]	1	1
group [implied]	6	12
home visit [explicitly face-to-face]	2	4
home visit [implied]	15	22
interview [explicitly face-to-face]	1	1
patient visits centre [implied]	5	11
physical therapist treatment [implied]	1	1
06.2.2. distance (synchronous)	17	34
Telemedicine videolink with peripheral monitoring devices (optional)	1	1
Telephone	16	29
06.2.3. asynchronous	6	12
Community Facebook group	1	1
email	1	2
Instructional video	1	2
Leaflet	1	1
Newsletter	1	1
Notications to assistive technology devices (alerts to tablets)	1	3
Paper-based manual	1	1
Schedule of activities	1	1
06.3. Interactivity	10	38
participant-professional	8	15
peer-to-peer	4	5
06.4. Other features	1	1
06b. How Organised	35	417
analyt	17	62
Case manager	12	31
case manager maybe	3	4
fragmented	1	1
integrated	5	6
MDT	4	4
MDT or nurse + physician	2	6
Nurse + physician	5	8
team other	1	2
deliverer inter-relations (team, liaison, referral, collaboration, communication, etc)	30	100
name or short description of the organisational system	7	10
organisations, geography and finance	20	54
finances	4	8
local-global	5	12
ownership	2	3
other	1	1
responsibilities of individual deliverers	22	59

responsibility for actioning	2	2
responsibility for care coordination	10	27
responsibility for planning	4	4
07. Where	50	236
07.1. Intervention location	41	98
07.1.1. Home	30	45
07.1.2. Other	21	38
Community-based Independent Living Centre	1	1
Environment around pt's home	1	1
In bed as bedbound	1	1
Local sports clubs, gyms, centre	2	2
Physician Practice	1	4
Supported living	1	4
Varied according to therapist's opinion	1	1
07.2. Equipment+facilities	9	18
07.3. Country+infrastructure	41	116
Germany	1	2
08. When and how much	48	325
08.1. When started	39	119
08.1.a. event - status - assessed category [tool or criteria] (timing)	37	96
Assessed as low mood and mild to moderate disability, and enrolled in community-based organization or clinic for immigrants or minorities	2	5
Assessed as pre-frail [Fried]	1	4
Assessed as pre-frail or frail [Fried] or inactive	1	3
Assessed as pre-frail or frail [GFI] (10 months after)	1	3
Assessed as vulnerable [ED visit, multimorbidity, frequent practice visits and polypharmacy]	3	5
Assessed at high risk [4 of 10 on GPSS]	1	7
Assessed at high risk for hospitalization and functional decline	1	6
Assessed at high risk of functional decline [SPQ]	1	2
Assessed at high risk of future health service use and receiving home care services	1	3
Assessed at risk of functional decline [ISCOPE]	2	4
Assessed at risk of functional decline [SPQ]	1	7
Assessed at risk of functional decline or institutionalization [CNQ]	1	7
Event - discharged from hospital (2-4 weeks after)	0	0
Event - referred for community OT, assessed as low priority (2 weeks after)	1	2
Event - referred for community OT, assessed as low priority (3-8 months after)	1	2
Event - referred for disability support services, assessed at high risk of institutionalisation	3	8
Event - referred for social work or occupational therapy for community maintenance	1	1
Multiple- Event - referral for in-home services, or Event - discharged from in-patient rehabilitation (past year), or Status - receiving home healthcare	2	4
No particular trigger - responded to general invitation	1	3

Status - 1+ chronic condition, enrolled in local health maintenance organization	2	3
Status - certified as requiring support, enrolled on public long-term care insurance	1	1
Status - enrolled in a senior center	1	2
Status - housebound	1	2
Status - member of district community centre	0	0
Status - not dependent in ADL and foreign born	2	3
Status - receiving home care services	2	2
Status - receiving informal home care but not formal	1	1
Status - resident of apartment-sharing with integrated nursing care	1	2
Status - resident of Independent Living Unit	1	2
Status - resident of Klong Toey slum	1	2
08.2. Duration of sessions	17	32
0.5 - 02 hours	1	1
01 - 01.5 hours (visits)	1	1
01 hour sessions (PM) SB unclear	1	1
01.5 hours	1	1
02 - 03 hours	2	2
02 h max	1	1
02h	1	1
15 - 90 minutes	1	3
15 mins to 2.5 hrs	1	3
30 minutes	1	1
30-60 min	2	2
60 min	1	1
60 minutes	2	2
90 - 120 minutes	1	1
Varied	1	2
08.3. Number of sessions	29	70
01 [only]	1	1
01 + [at least 1 session]	2	4
02 to 30+	1	3
03	1	4
04 +	1	1
04 +1	1	1
05	1	1
05 + (3.03 Home Visit + 1.17 Telephone call)	1	2
06	2	2
09	1	1
12 +	1	3
12 minimum	1	1
12 minimum +1 installation + 4 training	1	2
12+	1	1

13	1	3
13 +	1	5
14	2	4
18 +	1	1
18 maximum	2	3
33 max (1 POM, 24 Physical Fitness, 5 Social Skills, 3 Nutrition)	1	5
46 (10 PM + 36 SB)	1	2
54 max (25 set + 29 voluntary)	2	6
82	1	3
Varied	3	4
08.4. Session's schedule	35	91
08.4.1. Fixed	25	52
02 - 4 - 16 wks	1	1
1 off-session	2	3
1 weekly for a month + 1-off	1	2
23 weeks	1	1
2-3 weeks after group session (one-off)	1	1
24 weeks (12 weeks intensive, 12 weeks moderate)	1	2
2-monthly	2	3
2-weekly	4	5
3 monthly	2	3
3-monthly after 1 mth	1	2
3-weekly	1	1
3x per month for 3 months	1	2
3x per week	3	3
3x-4x per week	1	1
Monthly	2	4
Weekly	4	6
Weekly calls 1st mth - Fortnightly visits 0-3 mths - Monthly visits 3-6 mths (6 mths total)	1	2
Weekly x 7	1	2
08.4.2. Varied	17	29
15 month average (12-18 months)	2	3
1x per day - 2x per month	1	1
As and when needed	1	1
As needed	1	1
As required	1	1
Every 6 mths (CGA) & 2 weeks + upto 4x per day (contact)	2	3
Home visits varied (Minimum of 1 and maximum of 7)	1	2
Informal communication	1	2
PHC visit	1	1
Telephone calls from the nurse	1	1
Twice monthly	1	1

Unspecified	2	2
Upto 18 sessions in 3 months (12 weeks)	1	1
08.5. Intensity or dose (other)	4	9
09. Tailoring	40	247
action component tailoring	18	47
algorithmic	5	6
Patient preference incorporated	10	24
unclear or other	9	17
screening tailoring	1	1
unclear	1	2
Whole care plan tailoring	17	64
algorithmic	3	10
Patient preference incorporated	10	25
unclear or other	11	29
10. Modifications	3	6
11. How well (planned)	14	27
12. How well (actual)	20	60

Final coding structure

Name	Files	References
01. Brief name	237	647
02. Why	337	1653
Goal	280	655
Rationale or theory	275	992
03. What_ Materials	216	771
04. What_ Procedures	352	2964
b. providing- assessing and planning	233	1078
Medication assessment and planning	15	34
Monitoring with referral for multimodal assessment, planning and arranging	6	17
Multidomain assessment and planning leading to multifactorial action	202	932
Medication review	0	0
no	48	75
yes	75	119
Multidomain assessment and planning not shared or enacted	3	7
Multidomain assessment without planning and arranging	6	9
Multidomain assessment, planning and arranging (selective)	9	23
Routine screening with referral for multimodal assessment, planning and arranging	10	24
b.b. providing- reviewing and adjusting	134	289
routine follow-up	122	259
c. providing- actioning	150	563
01. Health Education	42	84
02. Exercises	52	139

03. ADL - Functional training	19	65
04. Aids - appliances – adaptations	18	66
06. Psychological procedures	6	14
08. Diets – nutrition	15	30
09. Homeopathy - naturopathy	1	4
10. Training and provision of technology for communication and engagement	5	22
13. Formal home care	35	84
Cognitive training	3	8
Engagement in meaningful activities	5	19
Health and social care voucher	2	3
Social skills empowerment training	1	2
Welfare rights advice with follow-up	4	11
c.m. providing- actioning (minor)	54	82
c.o. providing- actioning (selective)	77	205
f. available usual care	191	498
05. Who provided	232	1010
05.2 Disciplinary background	170	514
Care coordinator	14	21
Community health workers	2	2
Coordinator	1	1
Dietitian	12	15
Doctor (GP)	51	94
Doctor (secondary or tertirary)	9	12
Exercise trainers	4	5
Geriatrician	7	11
Health visitor	3	3
Home support staff	3	4
Nurse	99	161
Occupational therapist	33	46
Personal care assistant	5	7
Pharmacist	9	11
Physician	1	1
Physiotherapist	40	43
Podiatrist	1	2
Support staff	9	13
zOther	40	62
Acupuncturist	1	1
Audiologist	1	1
case manager (background unspecified)	2	2
clinical	1	1
Clinical assistant	1	1
Coaches	1	1
computer engineer	1	1

dental hygienists	2	3
Dentist	0	0
Formal and Informal caregiver	1	1
Geriatrician	6	7
Handy man	1	1
Handyman	2	2
Health care worker	1	3
health counselor	2	3
Homeopatic study physicians	1	1
lawyer	1	1
Medical student	1	2
municipality home care staff	2	2
municipality social care staff	1	1
Naturopath	1	1
neuropsychologist	1	1
no healthcare training staff	1	1
non-medical staff	1	1
Nurse OR community nurse	0	0
Nurse practitioner	2	2
nursing scientist	1	1
OR Social worker	2	5
Others - unspecified	4	5
Pain specialist	1	1
Paramedic	1	1
Physician's assistant	1	2
Police	1	1
psychiatrist	1	1
psychologist	2	2
Psychologist Trainer	1	1
Qualified Trainer (physical exercise)	1	1
Reablement care team leader (social care manager)	1	1
Reablement worker (social care worker)	1	1
Rehabilitation specialist	2	2
research assistant	2	2
researchers	2	5
Social educators (Social workers)	1	1
Social Worker	8	13
sociologist	1	1
specialists	2	2
Speech and language pathologist	1	1
Speech and Language Therapist	1	1
speech therapist	1	1
Sport therapist	2	2

Student helper	2	2
Trained health educator	1	1
Unskilled volunteers	1	5
Volunteer	1	1
Volunteers	1	3
voucher specialist	2	2
Welfare rights advisor	2	3
05.3 Other details	137	285
06. How	217	910
06.1. Individual-Group (size)	162	276
01 (Individual)	130	180
01 +	1	2
01 implied	0	0
01 or 02 (patient-carer)	9	12
02 (patient-carer dyad)	10	12
02 +	2	4
02+	1	1
02-05	2	3
04-06 (group of older adults)	6	6
04-07 (group of older adults)	2	2
06-08 (group of older adults)	1	4
08-10 (group of older adults)	3	3
10-15	1	1
11 max (group of older adults)	1	1
2+ (Individual + caregiver)	0	0
Group	14	21
Group (size not specified)	3	7
06.2. Medium	173	447
06.2.1. face to face	150	284
06.2.2. distance (synchronous)	79	114
06.2.3. asynchronous	33	48
06.4. Other features	55	120
Self-management psy techniques	46	103
06b. How Organised	253	1168
care coordination	111	195
no	51	85
unsure	12	20
yes	46	82
care planning decision-making	133	226
multidisciplinary	60	100
outsourced to GP	2	6
probably multidisciplinary	5	15
unidisciplinary	68	99

unsure	4	5
07. Where	248	737
07.1. Intervention location	160	293
07.1.1. Home	122	177
07.1.2. Other	57	96
07.2. Equipment+facilities	10	21
07.3. Country+infrastructure	214	417
08. When and how much	333	1558
08.1. When started	278	766
...	1	3
08.1.a. event - status - assessed category [tool or criteria] (timing)	35	84
Assessed as low mood and mild to moderate disability, and enrolled in community-based organization or clinic for immigrants or minorities	2	5
Assessed as pre-frail [Fried]	1	4
Assessed as pre-frail or frail [Fried] or inactive	1	3
Assessed as pre-frail or frail [GFI] (10 months after)	1	3
Assessed as vulnerable [ED visit, multimorbidity, frequent practice visits and polypharmacy]	3	4
Assessed at high risk [4 of 10 on GPSS]	1	6
Assessed at high risk for hospitalization and functional decline	1	5
Assessed at high risk of functional decline [SPQ]	1	2
Assessed at high risk of future health service use and receiving home care services	0	0
Assessed at risk of functional decline [ISCOPE]	2	4
Assessed at risk of functional decline [SPQ]	1	4
Assessed at risk of functional decline or institutionalization [CNQ]	1	7
Event - discharged from hospital (2-4 weeks after)	0	0
Event - referred for community OT, assessed as low priority (2 weeks after)	1	2
Event - referred for community OT, assessed as low priority (3-8 months after)	1	2
Event - referred for disability support services, assessed at high risk of institutionalisation	3	8
Event - referred for social work or occupational therapy for community maintenance	1	1
Multiple- Event - referral for in-home services, or Event - discharged from in-patient rehabilitation (past year), or Status - receiving home healthcare	2	4
No particular trigger - responded to general invitation	1	3
Status - 1+ chronic condition, enrolled in local health maintenance organization	2	3
Status - certified as requiring support, enrolled on public long-term care insurance	1	1
Status - enrolled in a senior center	1	2
Status - housebound	1	2
Status - member of district community centre	0	0
Status - not dependent (not receiving home help or nursing services)	1	1
Status - not dependent in ADL and foreign born	0	0
Status - receiving home care services	2	2
Status - receiving informal home care but not formal	1	1
Status - resident of apartment-sharing with integrated nursing care	1	1

Status - resident of Independent Living Unit	1	2
Status - resident of Klong Toey slum	1	2
Assessed - no need for BADLS assistance [Sent PRA] & Status - enrolled in a GP practice and 65 years or older	10	20
Assessed as 1 or + frailty criteria [FRAIL and modified Fried] & Status - 65 years old	3	13
Assessed as ambulatory frail [able to walk, no cognitive disorders, with IADL difficulty] by national standardized system and Status - not receiving long-term care services in last 3 mths	3	7
Assessed as frail (0.25 or more Frailty index) and no medical restrictions to exercise & Status 65 years old or older	2	8
Assessed as frail [3 or more Fried criteria] & Event - discharged from the hospital rehab and aged care service	6	14
Assessed as frail [GFI 5+] & 70+	5	13
Assessed as frail elderly [+75, +3 chronic diagnose, 6+ drugs, 3+ hospital stays in 6mths]	2	4
Assessed as functionally vulnerable (2 IADL or 1or+ ADL, HRCA Vulnerability Index 1or+) & 70 or older	3	5
Assessed as having complex problems by postal questionnaire (ISCOPE) sent by GP	4	7
Assessed as having difficulty on ADL or IADL due to chronic health conditions & Status - 60 years old or more, living alone, interested in computers	1	4
Assessed as impaired in at least 3 ADL & Status 80+ years old, German level of care 1-	4	9
Assessed as mildly frail [CFS]	2	5
Assessed as needing assistance, walking independently & going outdoors less than 3x week	1	4
Assessed as physically frail [rapid gait and single chair stand]	4	20
Assessed as self-perceived poor health not receiving or waiting for nursing home or home regular services	3	11
Assessed as undernourished or at risk of undernutrition AND Status - receiving home care services and 65+ years old	1	4
Assessed as vulnerable [any of - concern about community living, recent bereavement, hospitalization, acute illness, frequent physician contact, multiple medical problems, polypharmacy, adverse drug events, functional impairment, diagnostic uncertainty]	1	1
Assessed at risk of functional decline [ISAR-PC]	4	18
Assessed at risk of functional decline [VES]	2	5
Assessed multimorbidity and-or polypharmacy and-or care gap and 60 years or older	3	5
At least partial disable with chronic disease and difficult contact with practice	1	1
Event - discharged from rehabilitation hospital & Status - 50 years old of more	2	10
Event - following visit to ED & Assessed at-risk of functional decline (SF12-MCS less than 55 after 1 mth of ED)	2	11
Event - people applying for or referred to home-based care services	1	1
Event - post discharge, over 75 & Assessed as not needing district nurse	2	8
Event - referral to home care services	1	2
Event - referral to home care services & Assessed - 1+ ADL no complex needs	2	6
Event - referral to personal homecare services & Status 75+ years old	3	9
Event - referred by GP & Status - limitations in function, cognition or mental well being	5	8
Event- after admission to home health care & Assessed - need to improve at least 1 ADL	1	4

Identified as Frail by Postal Questionnaires	1	1
Identified minority and low income people through clinical site placement and outreach	0	0
New referral for home based support services	3	3
No particular trigger - responded to general invitation	3	11
Not dependent in ADLs	3	4
Older foreign-born people not dependent in ADLs	2	3
Referral to social services for assessment for substantial levels of care and consideration for residential or nursing home placement	1	4
Screened - fall concerns and activity avoidance postal questionnaire	3	5
Screened as moderate or severely impaired in ADLs	1	4
Screening positive in a casefinding questionnaire	2	5
Status - 60+ & Assessed - at least mild frail (CFAI-plus) or feel frail	3	10
Status - 60+ low-income enrolling 1st time health prevention	1	3
Status - 60+ mobile residing in a low-cost government subsidized flat & without uncontrolled health conditions or recent physical problems	2	4
Status - 60+ or older & Assessed - frail or pre-frail (Fried et al. phenotype and FinD)	4	9
Status - 60+ with one or more chronic illnesses & Event - post-discharge from a rehabilitative hospital	1	4
Status - 60+ years old with multiple chronic conditions, able to use telemonitoring & Assessed as at risk 15+ in ERA index	3	11
Status - 65+ & 5+ drugs & Assessed as frail (3 or + in PRIMSA-7)	1	7
Status - 65+ & Assessed risk factors to losing independence	3	9
Status - 65+ able to walk 2 or more min without person help & Assessed - SPPB = or +4 & sedentary and or not enough activity	4	5
Status - 65+ no acute health needs or major medical events in the last 6 months	1	4
Status - 65+ not completely ADL dependent with ISDN or ADSL services & Assessed - mild or moderately frail (EFS)	3	7
Status - 65+ not receiving home visits & Assessed - dependent IADLs independent ADLs	1	4
Status - 65+ older & multiple physical, social, functional problems or frail [unspecified criteria]	2	5
Status - 65+ receiving home care no serious cognitive or psychological problems of bedbound	3	6
Status - 65+ registered in GP practice	1	2
Status - 65+ years enrolled in primary care center & Assessed as frail based on Barber, Fried modified	2	8
Status - 68-79 years old and not in need of care	1	4
Status - 70 yr+ screened as pre-frail + Event - consulting in primary care	2	5
Status - 70+ or 50+ if indigenous or aboriginal or younger but has documented chronic or complex agerelated conditions & multiple morbidities or a social situation that requires the attention of multiple healthcare providers or facilities	3	6
Status - 70+ veteran eligible but not enrolled in VA health care	1	2
Status - 70+ veterans in geriatric or primary care without significant unresolved issues & not enganging in regular exercise	3	9
Status - 70+ years old not receiving rehab & Assessed as pre-frail [1or2 CHS criteria]	2	6
Status - 70+ years old, selected from the 2 GP practices age-sex register	1	3
Status - 70-78 years + no cardiovascular diseases or dementia + enrolled with participating GPs	5	9

Status - 75 & 65 if Maori, returned replay card, enrolled in GP	2	4
Status - 75 years old or more & enrolled in GP	1	1
Status - 75 years-old contacted by post with support of health care center	1	4
Status - 75 years-old or more	1	2
Status - 75+ and signed up in a GP practice which agreed to participate	2	9
Status - 75+ years patients of participating primary care practices	1	7
Status - 75-84 NOT using home care	3	6
Status - 80+ years old invited through various healthcare and community services and initiation letter	2	4
Status - Able to move outdoors independently + sedentary + cognitively intact	2	6
Status - At risk of disability and inactive	2	11
Status - At risk of malnutrition + Caregiver-dependent + participating in Home Care Program	2	6
Status - awaiting home-based services +low income & Assessed with difficulty in at least 1 ADL or 2 IADLs	1	3
Status - enrolled in a cohort study and 85 year old	3	5
Status - enrolled in a community center, 60+ years old	2	5
Status - enrolled in GP practices offering home visits, good rehabilitation, and GPs who could provide preventive care, and being 75 or 80 years old	7	15
Status - enrolled in health insurance program for +1 year and at high risk (by age or has chronic condition)	1	8
Status - enrolled in LUCAS cohort study and 60 years or older & Assessed as frail (LUCAS questionnaire)	2	11
Status - enrolled in Medicare A & B & Assessed - limitation 2+ADL or 3+IADL & Event - hospitalized, at nursing home or receiving home healthcare in last 12 mths or 2+ER last 6mths	3	6
Status - enrolled in the veteran affairs service, 70 years or older & Assessed as able to exercise (walk 30ft without help and no relevant conditions) and less than 150 min a week exercise	4	8
Status - enrolled in usual care health insurance; 75 years-old or more	2	4
Status - ethnically diverse +60 enrolled in senior center or senior housing residence	4	12
Status - in socioeconomically disadvantaged area recruited from GP	3	10
Status - Insured with CalPERS Longterm care program, 1+ chronic diagnosis, less than 2 ADL deficiencies	3	9
Status - living in federally subsidized apartments & from diverse cultures	4	10
Status - low income & Assessed with difficulty in at least 1 ADL or 2 IADLs	5	11
Status - low income elders referred to social services by health services & Assessed at medium risk based on uniform statewide assessment	1	6
Status - low income, enrolled in local health maintenance organization	4	11
Status - not dependent in ADL and foreign born	2	5
Status - participating in a longitudinal study & Assessed - not very physical and functionally active	2	5
Status - receiving home care & Assessed - level of care	1	3
Status - receiving home care services	5	12
Status - receiving home care services & diziness, hypotension previous fall or 6 or + medicine	2	7
Status- 65+ yrs Prefrail and frail (according to 5 Cardiovascular Health Study frailty phenotype)	1	2
When in low care need	2	2

08.2. Duration of sessions	96	147
08.3. Number of sessions	146	296
08.4. Session's schedule	170	325
08.4.1. Fixed	62	111
08.4.2. Varied	38	57
08.5. Intensity or dose (other)	9	17
09. Tailoring	233	696
10. Modifications	13	21
11. How well (planned)	155	407
12. How well (actual)	151	538

Appendix 3: Codebook

Name	Description
01. Brief name	Provide the name or a phrase that describes the intervention
02. Why	Describe any rationale, theory, or goal of the elements essential to the intervention
Goal	Goal of the elements essential to the intervention. AKA the aims. Note, this should only be the goal of the intervention, not the study.
Rationale or theory	Rationale or theory of the elements essential to the intervention. Includes mechanisms, active ingredients, causal paths, theoretical basis.
03. What_ Materials	Describe any physical or informational materials used in the intervention, including those provided to participants or used in intervention delivery or in training of intervention providers. Provide information on where the materials can be accessed (such as online appendix, URL)
a. training and implementation materials	Including those used in training of intervention providers and implementation of the intervention.
b. provided materials	materials provided to participants
c. materials used as part of delivery (not provided to participants)	Describe any physical or informational materials used in intervention delivery
d. other materials	materials not fitting the above categories.
04. What_ Procedures	Describe each of the procedures, activities, and/or processes used in the intervention, including any enabling or support activities
a. implementing (enabling)	procedures such as recruiting, training, establishing referral pathways, necessary to implement the intervention.
b. providing- assessing and planning	procedures mainly related to assessing a participant or planning their care. Add subcodes to this.
c. providing-intervening	Procedures mainly involving treating or otherwise intervening with patients. Create subcodes under this node.
d. resulting	Procedures, actions or behaviours that are supposed to be carried out by the patient, carer, etc. as a result of the intervening.

e. supporting	i.e, not setup (implementing), but not patient-specific, such as meetings, audits, ongoing training.
f. available usual care	care that is available to the population if needed but is not actively provided to most/all of the study population. Differentiate from standard care which is provided to the participants. Because of this it will depend on the population, but might be GP services, home care services… However, in another study, participants may be receiving home care services (for example) as part of a standard intervention (i.e. not available usual care) in comparison with an enhanced home care service or alternative.
05. Who provided	For each category of intervention provider (such as psychologist, nursing assistant), describe their expertise, background, and any specific training given
05.1 Number of intervention providers	Interpret this as per recipient of intervention (not total number of staff). Separate unidisciplinary from multidisciplinary and code with number of people
5.1.1. Unidisciplinary	
5.1.2. Multidisciplinary	
05.2 Disciplinary background	Add an in vivo code for each disciplinary background
Care coordinator	
Dietitian	
Doctor (GP)	General practitioner, AKA family physician, primary care doctor.
Doctor (secondary or tertirary)	Specialist doctor, e.g. geriatrician.
Health visitor	
Nurse	Including practice nurses, community nurses, nurse practitioners, palliative care and other specialist nurses
Occupational therapist	
Personal care assistant	
Pharmacist	

Physiotherapist	
Podiatrist	
Support staff	recptionists, secretarial and clerical staff
zOther	
05.3 Other details	Including: o Pre-existing skills reqd o Additional training o Delivery assessment (i.e. competence and monitoring) o Role (existing or specially recruited) o Incentives+reimbursement
06. How	Describe the modes of delivery (such as face to face or by some other mechanism, such as internet or telephone) of the intervention and whether it was provided individually or in a group
06.1. Individual-Group (size)	Code first with size then anything relevant to the relation between the participants / nature of the group, e.g. 2 (patient-carer dyad). Code size ranges with a dash between.
06.2. Medium	
06.2.1. face to face	in person, in the same room
06.2.2. distance (synchronous)	telecommunication such as telephone or live voice or video call
06.2.3. asynchronous	Communication where sending and receiving is not necessarily simultaneous or concurrent in time. i.e. received at a different time than when sent such as email, letter, webpage, text message, recorded video, app including videos
06.3. Interactivity	code anything related to interactivity (or passivity) under this code, such as that it involved questions and answers or that participants were encouraged to interact with each other.
07. Where	Describe the type(s) of location(s) where the intervention occurred, including any necessary infrastructure or relevant features
07.1. Intervention location	
07.1.1. Home	
07.1.2. Other	
07.2. Equipment+facilities	distinguish from materials, which are consumed/given

07.3. Country+infrastructure	
08. When and how much	Describe the number of times the intervention was delivered and over what period of time including the number of sessions, their schedule, and their duration, intensity, or dose
08.1. When started	timing of the intervention in relation to relevant events
08.2. Duration of sessions	
08.3. Number of sessions	in total, or as a range
08.4. Session's schedule	
08.4.1. Fixed	
08.4.2. Varied	
08.5. Intensity or dose (other)	
09. Tailoring	If the intervention was planned to be personalised, titrated or adapted, then describe what, why, when, and how Code at this (only) anything relating to tailoring.
10. Modifications	If the intervention was modified during the course of the study, describe the changes (what, why, when, and how) Code at this (only) anything relating to modifications.
11. How well (planned)	If intervention adherence or fidelity was assessed, describe how and by whom, and if any strategies were used to maintain or improve fidelity, describe them
12. How well (actual)	If intervention adherence or fidelity was assessed, describe the extent to which the intervention was delivered as planned

Appendix 4: PRISMA flow diagram, included studies and reports

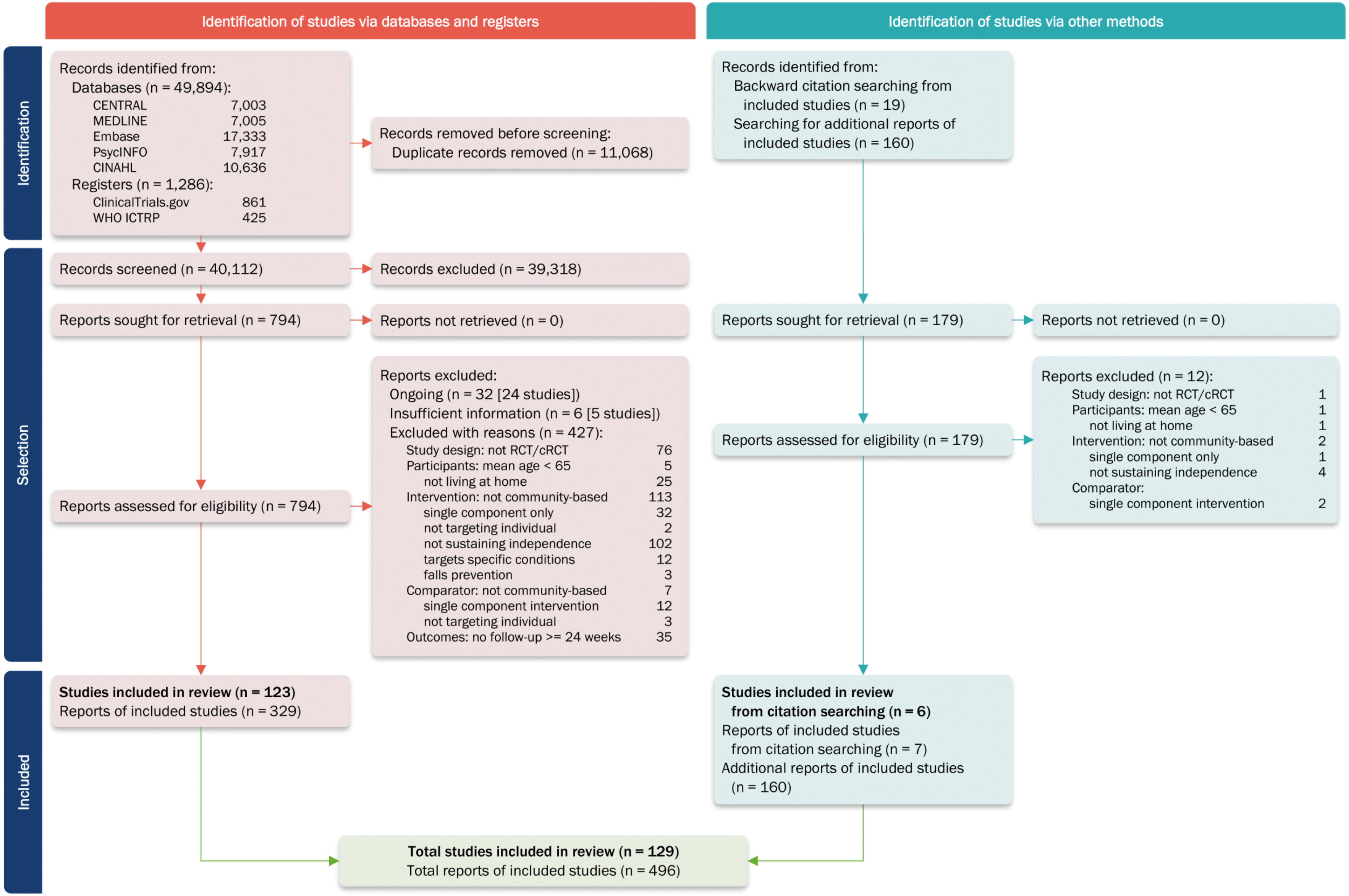

Figure 1. PRISMA flow diagram showing identification, selection and inclusion of studies from databases, registers and other sources.

Summary characteristics of 129 included studies[1-129]

Study	Design	Enrolment began	Country	Population frailty	Enrolled	Experimental interventions	Control interventions	Funding
Alegria 2019[1, 130, 131]	RCT	2015	USA	P	307	exrc & psyc	ac	NC
Arthanat 2019[2, 132, 133]	RCT	> 2005	USA	U	97	comm	ac	NC
Auvinen 2020[3, 134-136]	RCT	2015	FIN	F	512	hmcr & med	hmcr	NC
Balaban 1988[4]	RCT	1981	USA	F	198	mfa-(w/med)	ac	NC
Barenfeld 2018[5, 137-140]	RCT	2012	SWE	all	131	educ	ac	NC
Bernabei 1998[6, 141, 142]	RCT	1995	ITA	F	200	hmcr & mfar(w/med)	hmcr	NC
Bleijenberg 2016[7, 143-151]	cRCT	2010	NLD	P,F	m:39; 3092	rsk-mfa-;<br>rsk-mfa-	ac	NC
Blom 2016[8, 150, 152, 153]	cRCT	2009	NLD	all	m:59; 1379	mfa-(w/med+slfm)	ac	NC
Borrows 2013[9]	RCT	2008	GBR	U	36	aids	mfa-	NC
Botjes 2013[10, 154, 155]	RCT	2011	NLD	U	218	mfa-	ac	NC
Bouman 2008[11, 156-160]	RCT	2002	NLD	P,F	330	mfar(w/med)	ac	NC
Brettschneider 2015[12, 161-164]	RCT	2007	DEU	F	336	mfar(w/med)	ac	NC
Cameron 2013[13, 165-174]	RCT	2008	AUS	F	241	exrc & mfar(w/med+slfm)	ac	NC
Carpenter 1990[14]	RCT	< 2006	GBR	all	539	rsk-m+fa-	ac	NC
Cesari 2014[15, 175-180]	RCT	> 2005	FRA	U	?	mfar(w/med)	ac	NC
Challis 2004[16, 181]	RCT	1998	GBR	F	256	mfar(w/med)	mfar	NC
Clark 1997[17, 182-186]	RCT	1994	USA	R,P	361	eng & educ	ac	Mx
Clark 2012[18, 187-192]	RCT	2004	USA	U	460	eng & educ	ac	NC
Coleman 1999[19]	cRCT	< 2006	USA	F	m:9; 169	educ & mfar(w/med+slfm)	ac	NC
Counsell 2007[20, 193-197]	cRCT	2002	USA	U	m:164; 951	educ & mfar(w/med+slfm)	ac	NC
Cutchin 2009[21, 198]	RCT	2008	USA	U	110	mfar	ac	NC
Dalby 2000[22, 199]	RCT	< 2006	CAN	F	142	mfar(w/med)	ac	NC
de Craen 2006[23, 200-202]	RCT	2000	NLD	all	402	mfa-	ac	NC
Dorresteijn 2016[24, 203-206]	RCT	2009	NLD	U	389	ADL	ac	NC
Dupuy 2017[25, 207]	RCT	> 2005	FRA	P,F	32	hmcr & aids & comm	hmcr	NC
Fabacher 1994[26]	RCT	< 2006	USA	all	254	mfar(w/med)	ac	NC
Fairhall 2015[27 208, 209]	RCT	2013	AUS	P	230	mfar(w/med)	ac	NC
Faul 2009[28, 210]	RCT	?	USA	R,P	81	educ & exrc & mfar(w/med+slfm);<br>exrc & mfa-(w/med+slfm)		NC
Fernandez-Barres 2017[29, 211, 212]	RCT	2010	ESP	F	173	hmcr & ntr	hmcr	NC
Fischer 2009[30, 213]	RCT	2004	DEU	all	4224	eng & mfa-(w/slfm)	ac	?
Ford 1971[31, 214]	RCT	1963	USA	P,F	300	mfar(w/med)	ac	NC
Fox 1997[32]	RCT	1994	USA	all	237	mfar(w/med+slfm)	mfar(w/med)	NC
Fristedt 2019[33, 215]	RCT	2015	SWE	F	62	hmcr & mfar(w/med)	hmcr	NC
Gene Huguet 2018[34]	RCT	2016	ESP	P	200	med & ntr & exrc	ac	NC

Study	Design	Enrolment began	Country	Population frailty	Enrolled	Experimental interventions	Control interventions	Funding
Gill 2002[35, 216-219]	RCT	< 2006	USA	P,F	188	ADL & exrc	ac	NC
Giné-Garriga 2020[36, 220-230]	RCT	2016	EEE	R	1360	exrc	ac	NC
Gitlin 2006[37, 231-241]	RCT	2003	USA	P,F	319	ADL & aids & exrc	ac	NC
Grimmer 2013[38, 242]	RCT	2014	AUS	U	?	mfa-	ac	?
Gustafson 2021[39, 243, 244]	RCT	2013	USA	all	390	aids & educ & comm	ac	Mx
Gustafsson 2013[40, 137, 245-252]	RCT	2007	SWE	all	491	educ & mfa-;<br>educ	ac	NC
Hall 1992[41]	RCT	1986	CAN	F	167	hmcr & mfar(w/slfm)	hmcr & mfar	?
Harari 2008[42 253-267]	RCT	2000	GBR	all	2503	mfar(w/med)	ac	NC
Hattori 2019[43, 268]	RCT	2018	JPN	P,F	375	educ & mfar(w/slfm)	mfar	NC
Hay 1998[44, 269]	RCT	< 2006	CAN	U	619	mfa-	ac;<br>ac	NC
Hebert 2001[45]	RCT	< 2006	CAN	P,F	503	mfar(w/med)	ac	NC
Henderson 2005[46, 270]	cRCT	2002	AUS	R	m:16; 167	mfar	ac	NC
Hendriksen 1984[47, 271-273]	RCT	1980	DNK	all	600	mfar	ac	NC
Hogg 2009[48, 274-278]	RCT	2004	CAN	U	241	mfar(w/med)	ac	NC
Holland 2005[49, 279, 280]	RCT	2001	USA	U	504	educ & exrc & mfar(w/slfm)	ac	NC
Howel 2019[50, 281-283]	RCT	2011	GBR	all	755	wlfr	ac	NC
Imhof 2012[51, 151]	RCT	2008	CHE	all	461	mfar	ac	NC
Jing 2018[52]	RCT	2016	CHN	F	80	psyc;<br>exrc & psyc		?
Jitapunkul 1998[53]	RCT	1993	THA	U	160	rsk-mfa-	ac	NC
Kerse 2014[54, 284-288]	cRCT	2008	NZL	P,F	m:60; 3893	rsk-mfa-	ac	NC
King 2012[55, 289-291]	cRCT	2006	NZL	P,F	m:21; 186	hmcr & ADL & mfar(w/slfm)	hmcr	NC
Kono 2016[56, 292, 293]	RCT	2011	JPN	P	360	mfar(w/med)	mfar	NC
Kono 2004[57]	RCT	2000	JPN	P,F	119	mfar	ac	NC
Kono 2012[58, 294-296]	RCT	2008	JPN	P	323	mfar	mfar	NC
Kukkonen-Harjula 2017[59, 297-301]	RCT	2014	FIN	P,F	300	ADL & ntr & exrc	ac	NC
Lambotte 2018[60, 302-309]	RCT	2017	BEL	P,F	871	mfar	ac	NC
Leung 2004[61, 310]	RCT	2000	HKG	all	260	mfar(w/med)	ac	?
Leveille 1998[62, 311, 312]	RCT	1995	USA	U	201	educ & exrc & mfar(w/med+slfm)	ac	NC
Lewin 2013[63, 313-315]	RCT	2005	AUS	F	750	hmcr & educ & mfar	hmcr	NC
Liddle 1996[64]	RCT	< 2006	AUS	U	105	aids & mfar	ac	NC
Liimatta 2019[65, 316-318]	RCT	2013	FIN	R,P	422	exrc & mfa-(w/med)	ac	NC
Loh 2015[66, 319, 320]	cRCT	2014	MYS	U	m:8; 256	ntr & exrc	ac	NC
Lood 2015[67]	RCT	2012	SWE	R,P	40	educ	ac	NC
Mann J 2021[68, 321-325]	cRCT	2018	AUS	all	m:14; 92	mfa-(w/med)	ac	NC
Mann WC 1999[69]	RCT	< 2006	USA	F	104	hmcr & aids	hmcr	NC
Markle-Reid 2006[70, 326, 327]	RCT	2001	CAN	F	288	hmcr & mfar(w/med+slfm)	hmcr & mfar	NC
Melis 2008[71, 328-334]	RCT	2003	NLD	F	155	mfar(w/med)	ac	NC

Study	Design	Enrolment began	Country	Population frailty	Enrolled	Experimental interventions	Control interventions	Funding
Meng 2005[72, 335-341]	RCT	1998	USA	F	1786	educ & vchr & mfar(w/med+slfm);<br>educ & mfar(w/med+slfm);<br>vchr	ac	NC
Messens 2014[73, 342, 343]	RCT	2011	EEE	P,F	208	aids & cgn & comm & mntr-mfa-	ac	NC
Metzelthin 2013[74, 150, 344-349]	cRCT	2009	NLD	F	m:12; 346	educ & mfar(w/med+slfm)	ac	NC
Moll van Charante 2016[75, 350-360]	cRCT	2006	NLD	all	m:116; 3526	educ & mfar(w/slfm)	ac	NC
Monteserin Nadal 2008[76, 361]	RCT	2004	ESP	all	620	educ & rsk-mfa-	ac	NC
Morey 2006[77, 362, 363]	RCT	< 2006	USA	all	179	exrc;<br>exrc	exrc	NC
Morey 2009[78, 364-367]	RCT	2004	USA	U	400	exrc	ac	NC
Morgan 2019[79, 368-370]	RCT	2014	GBR	P	51	exrc	ac	NC
Newbury 2001[80, 371]	RCT	1998	AUS	U	100	mfa-(w/med)	ac	NC
Newcomer 2004[81, 372, 373]	RCT	2001	USA	U	3079	educ & mfar(w/med)	ac	Mx
Ng 2015[82, 374, 375]	RCT	2009	SGP	P,F	246	cgn & ntr & exrc	ac	NC
Parsons J 2012[83, 376, 377]	cRCT	2007	NZL	P,F	m:?; 205	hmcr & mfar(w/slfm)	hmcr & mfa-	NC
Parsons M 2017[84, 378-381]	RCT	2003	NZL	F	113	hmcr & ADL & mfar(w/slfm)	hmcr & mfa-	NC
Parsons M 2012[85, 378-381]	cRCT	2003	NZL	F	m:55; 351	hmcr & mfar	hmcr & mfa-	NC
Pathy 1992[86]	RCT	< 2006	GBR	all	725	rsk-mfa-	ac	NC
Phelan 2007[87]	cRCT	2002	USA	all	m:31; 874	mfar(w/med+slfm)	ac	NC
Ploeg 2010[88, 382]	RCT	2004	CAN	P,F	719	educ & mfar(w/med)	ac	NC
Profener 2016[89, 383-385]	RCT	2007	DEU	F	553	educ & mfar	ac	NC
Rockwood 2000[90, 386]	RCT	< 2006	CAN	F	182	mfa-(w/med)	ac	NC
Romera-Liebana 2018[91, 387, 388]	RCT	2013	ESP	P,F	352	cgn & med & ntr & exrc	ac	NC
Rooijackers 2021[92, 389-393]	cRCT	2017	NLD	F	m:10; 264	hmcr & ADL & mfar(w/slfm)	hmcr	NC
Rubenstein 2007[93]	RCT	< 2006	USA	F	792	mfar(w/med)	ac	NC
Ryvicker 2011[94, 394]	cRCT	2005	USA	U	m:45; 3290	hmcr & mfar	hmcr & mfar	NC
Serra-Prat 2017[95, 395]	RCT	2013	ESP	P	172	ntr & exrc	ac	NC
Shapiro 2002[96]	RCT	1998	USA	F	108	hmcr & mfar	ac	NC
Sherman 2016[97, 396]	cRCT	2006	SWE	all	m:16; 583	mfa-(w/med)	ac	NC
Siemonsma 2018[98, 397, 398]	RCT	2009	NLD	F	155	ADL	mfa-	NC
Stewart 2005[99, 399, 400]	RCT	2000	GBR	P,F	321	mfa-	mfa-	NC
Stuck 1995[100, 401-405]	RCT	1988	USA	all	414	educ & mfar(w/med)	ac	NC
Stuck 2000[101, 406-409]	RCT	1993	CHE	all	791	mfar(w/med)	ac	NC
Stuck 2015[102, 264-267, 410]	RCT	2000	CHE	R,P	2284	educ & mfar(w/med+slfm)	ac	NC
Suijker 2016[103, 411-416]	cRCT	2010	NLD	F	m:24; 2283	mfar(w/med)	ac	NC
Szanton 2011[104, 417, 418]	RCT	2010	USA	P,F	40	ADL & aids & educ & exrc &<br>mfar(w/med+slfm)	ac	NC

Study	Design	Enrolment began	Country	Population frailty	Enrolled	Experimental interventions	Control interventions	Funding
Szanton 2019[105, 417, 419-428]	RCT	2012	USA	P,F	300	ADL & aids & educ & exrc & mfar(w/med+slfm)	ac	NC
Takahashi 2012[106, 429-435]	RCT	2009	USA	F	205	mntr-mfa-	ac	Mx
Teut 2013[107, 436]	cRCT	2009	DEU	F	m:8; 58	hmcr & hmnt & exrc	hmcr	Mx
Thiel 2019[108, 437, 438]	RCT	2017	DEU	F	?	exrc & mfar(w/med)	ac	NC
Thomas 2007[109]	RCT	2001	CAN	P,F	520	mfar(w/med); mfar(w/med)	ac	?
Tomita 2007[110]	RCT	< 2006	USA	F	124	aids	ac	NC
Tulloch 1979[111]	RCT	1972	GBR	all	339	mfar(w/med)	ac	?
Tuntland 2015[112, 439-441]	RCT	2012	NOR	U	61	hmcr & ADL & aids & mfa-(w/slfm)	hmcr & mfa-	NC
van der Pols-Vijlbrief 2017[113, 442]	RCT	2013	NLD	F	155	hmcr & ntr & mfar	hmcr	NC
van Dongen 2020[114, 443-446]	RCT	2016	NLD	all	168	ntr & exrc	ac	Mx
van Heuvelen 2005[115, 447]	RCT	2001	NLD	P,F	233	exrc & psyc	ac	NC
van Hout 2010[116, 448, 449]	RCT	2002	NLD	F	658	mfar(w/med)	ac	NC
van Leeuwen 2015[117, 450-454]	cRCT	2010	NLD	F	m:35; 1147	mfar(w/med+slfm)	ac	NC
van Lieshout 2018[118, 455]	RCT	2011	NLD	P,F	710	ADL & med & ntr & sst	ac	NC
van Rossum 1993[119, 456, 457]	RCT	1988	NLD	all	580	mfar	ac	NC
Vass 2005[120, 458-478]	cRCT	1999	DNK	all	m:34; 4060	mfar(w/med)	mfar	NC
Vetter 1984[121]	RCT	1980	GBR	all	1148	mfar	ac	NC
von Bonsdorff 2008[122, 479-484]	RCT	2003	FIN	R	632	exrc	ac	NC
Wallace 1998[123, 312]	RCT	< 2006	USA	all	100	exrc & mfar	ac	NC
Walters 2017[124, 485, 486]	RCT	2015	GBR	P	51	mfar(w/slfm)	ac	NC
Whitehead 2016[125, 487, 488]	RCT	2014	GBR	F	30	hmcr & ADL & aids & mfa-	hmcr & mfa-	NC
Williams 1992[126, 489]	RCT	< 2006	GBR	all	470	mfar	mfa-	NC
Wolter 2013[127, 490-492]	cRCT	2007	DEU	F	m:69; 920	hmcr & mfar(w/med)	hmcr	NC
Wong 2019[128, 493-496]	RCT	2016	HKG	all	540	mfar(w/slfm)	ac	NC
Yamada 2003[129]	RCT	1999	JPN	P,F	368	mfar(w/med)	ac	NC

Note that some reports provide information about multiple studies and are therefore cited more than once.

RCT: Randomised Controlled Trial; cRCT: cluster RCT.

Countries, territories, or areas of geographical interest, are indicated with ISO 3166-1 alpha-3 codes, except EEE to indicate a study in multiple European countries: AUS, Australia; BEL, Belgium; CAN, Canada; CHE, Switzerland; CHN, China; DEU, Germany; DNK, Denmark; ESP, Spain; FIN, Finland; FRA, France; GBR, United Kingdom; HKG, Hong Kong; ITA, Italy; JPN, Japan; MYS, Malaysia; NLD, Netherlands; NOR, Norway; NZL, New Zealand; SGP, Singapore; SWE, Sweden; THA, Thailand; USA, United States of America.

all: All frailty groups; R: Robust; P: Pre-frail; F: Frail, U, Unclassified.

m indicates the number of clusters assigned for cluster RCTs.

Intervention and control group abbreviations are a combination of the following:- ac: available care; ADL: activities of daily living training; aids: provision of aids and adaptions; cgn: cognitive training; comm: technology for communication and engagement; educ: health education; eng: engagement in meaningful activities; exrc: physical exercise; hmcr: formal homecare; hmnt: alternative

medicine; med: medication-review; mfa: multifactorial-action; mfar: multifactorial-action and follow-on routine review; mntr-mfa: monitoring, which may trigger multifactorial-action; ntr: nutritional support; psyc: psychological therapy; rsk-mfa: risk screening, which may trigger multifactorial-action; sst: social skills training; vchr: care voucher provision; wlfr: welfare rights advice; w/med: with medication-review; w/slfm: with self-management. See main paper for further information.

Funding:- C: Commercial; Mx: Mixed; NC: Non-Commercial; ? Unclear

References to included studies

1. Alegria M, Frontera W, Cruz-Gonzalez M, Markle SL, Trinh-Shevrin C, Wang Y*, et al.* Effectiveness of a Disability Preventive Intervention for Minority and Immigrant Elders: The Positive Minds-Strong Bodies Randomized Clinical Trial. *Am J Geriatr Psychiatry* 2019;**27**:1299–313. https://doi.org/10.1016/j.jagp.2019.08.008
2. Arthanat S. Promoting information communication technology adoption and acceptance for aging-in-place: A randomized controlled trial. *J Appl Gerontol* 2019;**40**:471–80. https://doi.org/10.1177/0733464819891045
3. Auvinen K, Voutilainen A, Jyrkkä J, Lönnroos E, Mäntyselkä P. Interprofessional medication assessment among home care patients: any impact on functioning? Results from a randomised controlled trial. *BMC Geriatr* 2020;**20**:390. https://doi.org/10.1186/s12877-020-01796-1
4. Balaban DJ, Goldfarb NI, Perkel RL, Carlson BL. Follow-up study of an urban family medicine home visit program. *J Fam Pract* 1988;**26**:307–12.
5. Barenfeld E, Dahlin-Ivanoff S, Wallin L, Gustafsson S. Promoting aging migrants' capabilities: A randomized controlled trial concerning activities of daily living and self-rated health. *AIMS Public Health* 2018;**5**:173–88. https://doi.org/10.3934/publichealth.2018.2.173
6. Bernabei R, Landi F, Gambassi G, Sgadari A, Zuccala G, Mor V*, et al.* Randomised trial of impact of model of integrated care and case management for older people living in the community. *BMJ* 1998;**316**:1348–51. https://doi.org/10.1136/bmj.316.7141.1348
7. Bleijenberg N, Drubbel I, Schuurmans MJ, Dam HT, Zuithoff NP, Numans ME*, et al.* Effectiveness of a proactive primary care program on preserving daily functioning of older people: a cluster randomized controlled trial. *J Am Geriatr Soc* 2016;**64**:1779–88. https://doi.org/10.1111/jgs.14325
8. Blom J, den Elzen W, van Houwelingen AH, Heijmans M, Stijnen T, Van den Hout W*, et al.* Effectiveness and cost-effectiveness of a proactive, goal-oriented, integrated care model in general practice for older people. A cluster randomised controlled trial: Integrated Systematic Care for older People--the ISCOPE study. *Age Ageing* 2016;**45**:30–41. https://doi.org/10.1093/ageing/afv174
9. Borrows A, Holland R. Independent living centre occupational therapy (OT) versus routine community OT. *Int J Ther Rehabil* 2013;**20**:187–94. https://doi.org/10.12968/ijtr.2013.20.4.187
10. Botjes E. *Methodebeschrijving EigenKrachtWijzer: Databank Effectieve sociale interventies* Report: Movisie; 2013. URL: https://www.movisie.nl/sites/movisie.nl/files/2018-03/Methodebeschrijving-eigenkrachtwijzer.pdf (Accessed 17 May 2020).
11. Bouman A, van Rossum E, Ambergen T, Kempen G, Knipschild P. Effects of a home visiting program for older people with poor health status: a randomized, clinical trial in The Netherlands. *J Am Geriatr Soc* 2008;**56**:397–404. https://doi.org/10.1111/j.1532-5415.2007.01565.x
12. Brettschneider C, Luck T, Fleischer S, Roling G, Beutner K, Luppa M*, et al.* Cost-utility analysis of a preventive home visit program for older adults in Germany. *BMC Health Serv Res* 2015;**15**:141. https://doi.org/10.1186/s12913-015-0817-0
13. Cameron ID, Fairhall N, Langron C, Lockwood K, Monaghan N, Aggar C*, et al.* A multifactorial interdisciplinary intervention reduces frailty in older people: randomized trial. *BMC Med* 2013;**11**:65. https://doi.org/10.1186/1741-7015-11-65
14. Carpenter GI, Demopoulos GR. Screening the elderly in the community: controlled trial of dependency surveillance using a questionnaire administered by volunteers. *BMJ* 1990;**300**:1253–6. https://doi.org/10.1136/bmj.300.6734.1253

15. Cesari M, Demougeot L, Boccalon H, Guyonnet S, Vellas B, Andrieu S. The Multidomain Intervention to preveNt disability in ElDers (MINDED) project: rationale and study design of a pilot study. *Contemp Clin Trials* 2014;**38**:145–54. https://doi.org/10.1016/j.cct.2014.04.006
16. Challis D, Clarkson P, Williamson J, Hughes J, Venables D, Burns A*, et al.* The value of specialist clinical assessment of older people prior to entry to care homes. *Age Ageing* 2004;**33**:25–34. https://doi.org/10.1093/ageing/afh007
17. Clark F, Azen SP, Zemke R, Jackson J, Carlson M, Mandel D*, et al.* Occupational therapy for independent-living older adults. A randomized controlled trial. *JAMA* 1997;**278**:1321–6. https://doi.org/10.1001/jama.1997.03550160041036
18. Clark F, Jackson J, Carlson M, Chou C-P, Cherry BJ, Jordan-Marsh M*, et al.* Effectiveness of a lifestyle intervention in promoting the well-being of independently living older people: results of the Well Elderly 2 Randomised Controlled Trial. [Erratum in: J Epidemiol Community Health 2012;66:1079-82]. *J Epidemiol Community Health* 2012;**66**:782–90. https://doi.org/10.1136/jech.2009.099754
19. Coleman EA, Grothaus LC, Sandhu N, Wagner EH. Chronic Care Clinics: A randomized controlled trial of a model of primary care for frail older adults. *J Am Geriatr Soc* 1999;**47**:775–83. https://doi.org/10.1111/j.1532-5415.1999.tb03832.x
20. Counsell SR, Callahan CM, Clark DO, Tu W, Buttar AB, Stump TE*, et al.* Geriatric care management for low-income seniors: a randomized controlled trial. *JAMA* 2007;**298**:2623–33. https://doi.org/10.1001/jama.298.22.2623
21. Cutchin MP, Coppola S, Talley V, Svihula J, Catellier D, Shank KH. Feasibility and effects of preventive home visits for at-risk older people: design of a randomized controlled trial. *BMC Geriatr* 2009;**9**:54. https://doi.org/10.1186/1471-2318-9-54
22. Dalby DM, Sellors JW, Fraser FD, Fraser C, van Ineveld C, Howard M. Effect of preventive home visits by a nurse on the outcomes of frail elderly people in the community: a randomized controlled trial. *CMAJ* 2000;**162**:497–500.
23. de Craen AJ, Gussekloo J, Blauw GJ, Willems CG, Westendorp RG. Randomised controlled trial of unsolicited occupational therapy in community-dwelling elderly people: the LOTIS trial. *PLoS Clin Trials* 2006;**1**:e2. https://doi.org/10.1371/journal.pctr.0010002
24. Dorresteijn TA, Zijlstra GA, Ambergen AW, Delbaere K, Vlaeyen JW, Kempen GI. Effectiveness of a home-based cognitive behavioral program to manage concerns about falls in community-dwelling, frail older people: results of a randomized controlled trial. *BMC Geriatr* 2016;**16**:2. https://doi.org/10.1186/s12877-015-0177-y
25. Dupuy L, Froger C, Consel C, Sauzeon H. Everyday functioning benefits from an assisted living platform amongst frail older adults and their caregivers. *Front Aging Neurosci* 2017;**9**:302. https://doi.org/10.3389/fnagi.2017.00302
26. Fabacher D, Josephson K, Pietruszka F, Linderborn K, Morley JE, Rubenstein LZ. An in-home preventive assessment program for independent older adults: a randomized controlled trial. *J Am Geriatr Soc* 1994;**42**:630–8. https://doi.org/10.1111/j.1532-5415.1994.tb06862.x
27. Fairhall N, Kurrle SE, Sherrington C, Lord SR, Lockwood K, John B*, et al.* Effectiveness of a multifactorial intervention on preventing development of frailty in pre-frail older people: study protocol for a randomised controlled trial. *BMJ Open* 2015;**5**:e007091. https://doi.org/10.1136/bmjopen-2014-007091
28. Faul AC, Yankeelov PA, Rowan NL, Gillette P, Nicholas LD, Borders KW*, et al.* Impact on geriatric assessment and self-management support on community-dwelling older adults with chronic illnesses. *J Gerontol Soc Work* 2009;**52**:230–49. https://doi.org/10.1080/01634370802609288
29. Fernandez-Barres S, Garcia-Barco M, Basora J, Martinez T, Pedret R, Arija V*, et al.* The efficacy of a nutrition education intervention to prevent risk of malnutrition for dependent

elderly patients receiving Home Care: A randomized controlled trial. *Int J Nurs Stud* 2017;**70**:131–41. https://doi.org/10.1016/j.ijnurstu.2017.02.020
30. Fischer G, Sandholzer H, Perschke-Hartmann C. *Final report of the scientific support of "Getting Healthy Elderly (GÄW)". A prevention project of the AOK Lower Saxony.)[German] (Abschlussbericht der wissenschaftlichen Begleitung von "Gesund Älter Werden (GÄW)").* unpublished: AOK Niedersachsen; 2009.
31. Ford AB, Katz S, Downs TD, Adams M. Results of long-term home nursing: the influence of disability. *J Chronic Dis* 1971;**24**:591–6. https://doi.org/10.1016/0021-9681(71)90047-6
32. Fox PJ, Breuer W, Wright JA. Effects of a health promotion program on sustaining health behaviors in older adults. *Am J Prev Med* 1997;**13**:257–64.
33. Fristedt S, Nystedt P, Skogar O. Mobile geriatric teams - a cost-effective way of improving patient safety and reducing traditional healthcare utilization among the frail elderly? A randomized controlled trial. *Clin Interv Aging* 2019;**14**:1911–24. https://doi.org/10.2147/CIA.S208388
34. Gene Huguet L, Navarro Gonzalez M, Kostov B, Ortega Carmona M, Colungo Francia C, Carpallo Nieto M*, et al.* Pre Frail 80: Multifactorial intervention to prevent progression of pre-frailty to frailty in the elderly. *J Nutr Health Aging* 2018;**22**:1266–74. https://doi.org/10.1007/s12603-018-1089-2
35. Gill TM, Baker DI, Gottschalk M, Peduzzi PN, Allore H, Byers A. A program to prevent functional decline in physically frail, elderly persons who live at home. *N Engl J Med* 2002;**347**:1068–74. https://doi.org/10.1056/NEJMoa020423
36. Giné-Garriga M, Sansano-Nadal O, Tully MA, Caserotti P, Coll-Planas L, Rothenbacher D*, et al.* Accelerometer-measured sedentary and physical activity time and their correlates in European older adults: The SITLESS study. *J Gerontol A Biol Sci Med Sci* 2020;**75**:1754–62. https://doi.org/10.1093/gerona/glaa016
37. Gitlin LN, Winter L, Dennis MP, Corcoran M, Schinfeld S, Hauck WW. A randomized trial of a multicomponent home intervention to reduce functional difficulties in older adults. *J Am Geriatr Soc* 2006;**54**:809–16. https://doi.org/10.1111/j.1532-5415.2006.00703.x
38. Grimmer K, Luker J, Beaton K, Kumar S, Crockett A, Price K. TRialing individualized interventions to prevent functional decline in at-risk older adults (TRIIFL): study protocol for a randomized controlled trial nested in a longitudinal observational study. *Trials* 2013;**14**:266. https://doi.org/10.1186/1745-6215-14-266
39. Gustafson DH, Kornfield R, Mares M-L, Johnston DC, Cody OJ, Yang EF*, et al.* Effect of an eHealth intervention on older adults' quality of life and health-related outcomes: a randomized clinical trial. *J Gen Intern Med* 2021;**37**:521–30. https://doi.org/10.1007/s11606-021-06888-1
40. Gustafsson S, Eklund K, Wilhelmson K, Edberg A-K, Johansson B, Kronlöf GH*, et al.* Long-Term Outcome for ADL Following the Health-Promoting RCT—Elderly Persons in the Risk Zone. *Gerontologist* 2013;**53**:654–63. https://doi.org/10.1093/geront/gns121
41. Hall N, De Beck P, Johnson D, Mackinnon K, Gutman G, Glick N. Randomized trial of a health promotion program for frail elders. *Can J Aging* 1992;**11**:72–91.
42. Harari D, Iliffe S, Kharicha K, Egger M, Gillmann G, von Renteln-Kruse W*, et al.* Promotion of health in older people: a randomised controlled trial of health risk appraisal in British general practice. *Age Ageing* 2008;**37**:565–71. https://doi.org/10.1093/ageing/afn150
43. Hattori S, Yoshida T, Okumura Y, Kondo K. Effects of reablement on the independence of community-dwelling older adults with mild disability: A randomized controlled trial. *Int J Environ Res Public Health* 2019;**16**:3954. https://doi.org/10.3390/ijerph16203954
44. Hay WI, van Ineveld C, Browne G, Roberts J, Bell B, Mills M*, et al.* Prospective care of elderly patients in family practice. Is screening effective? *Can Fam Physician* 1998;**44**:2677–87.

45. Hebert R, Robichaud L, Roy PM, Bravo G, Voyer L. Efficacy of a nurse-led multidimensional preventive programme for older people at risk of functional decline. A randomized controlled trial. *Age Ageing* 2001;**30**:147–53. https://doi.org/10.1093/ageing/30.2.147
46. Henderson MJ. *In-home preventive health assessment and telephone case management for over 75s living alone in independent living units: A cluster randomized controlled trial* [PhD thesis]. Queensland: Queensland University of Technology; 2005.
47. Hendriksen C, Lund E, Stromgard E. Consequences of assessment and intervention among elderly people: a three year randomised controlled trial. *Br Med J (Clin Res Ed)* 1984;**289**:1522–4. https://doi.org/10.1136/bmj.289.6457.1522
48. Hogg W, Lemelin J, Dahrouge S, Liddy C, Armstrong CD, Legault F*, et al.* Randomized controlled trial of anticipatory and preventive multidisciplinary team care: for complex patients in a community-based primary care setting. *Can Fam Physician* 2009;**55**:e76–85.
49. Holland SK, Greenberg J, Tidwell L, Malone J, Mullan J, Newcomer R. Community-based health coaching, exercise, and health service utilization. *J Aging Health* 2005;**17**:697–716. https://doi.org/10.1177/0898264305277959
50. Howel D, Moffatt S, Haighton C, Bryant A, Becker F, Steer M*, et al.* Does domiciliary welfare rights advice improve health-related quality of life in independent-living, socio-economically disadvantaged people aged ≥60 years? Randomised controlled trial, economic and process evaluations in the North East of England. *PLoS One* 2019;**14**:e0209560. https://doi.org/10.1371/journal.pone.0209560
51. Imhof L, Naef R, Wallhagen MI, Schwarz J, Mahrer-Imhof R. Effects of an advanced practice nurse in-home health consultation program for community-dwelling persons aged 80 and older. *J Am Geriatr Soc* 2012;**60**:2223–31. https://doi.org/10.1111/jgs.12026
52. Jing L, Jin Y, Zhang X, Wang F, Song Y, Xing F. The effect of Baduanjin qigong combined with CBT on physical fitness and psychological health of elderly housebound. *Medicine* 2018;**97**:e13654. https://doi.org/10.1097/MD.0000000000013654
53. Jitapunkul S. A randomised controlled trial of regular surveillance in Thai elderly using a simple questionnaire administered by non-professional personnel. *J Med Assoc Thai* 1998;**81**:352–6.
54. Kerse N, McLean C, Moyes SA, Peri K, Ng T, Wilkinson-Meyers L*, et al.* The cluster-randomized BRIGHT trial: Proactive case finding for community-dwelling older adults. *Ann Fam Med* 2014;**12**:514–24. https://doi.org/10.1370/afm.1696
55. King AII, Parsons M, Robinson E, Jorgensen D. Assessing the impact of a restorative home care service in New Zealand: A cluster randomised controlled trial. *Health Soc Care Community* 2012;**20**:365–74. https://doi.org/10.1111/j.1365-2524.2011.01039.x
56. Kono A, Izumi K, Yoshiyuki N, Kanaya Y, Rubenstein LZ. Effects of an updated preventive home visit program based on a systematic structured assessment of care needs for ambulatory frail older adults in Japan: A randomized controlled trial. *J Gerontol A Biol Sci Med Sci* 2016;**71**:1631–7. https://doi.org/10.1093/gerona/glw068
57. Kono A, Kai I, Sakato C, Harker JO, Rubenstein LZ. Effect of preventive home visits for ambulatory housebound elders in Japan: a pilot study. *Aging Clin Exp Res* 2004;**16**:293–9. https://doi.org/10.1007/BF03324554
58. Kono A, Kanaya Y, Fujita T, Tsumura C, Kondo T, Kushiyama K*, et al.* Effects of a preventive home visit program in ambulatory frail older people: a randomized controlled trial. *J Gerontol A Biol Sci Med Sci* 2012;**67**:302–9. https://doi.org/10.1093/gerona/glr176
59. Kukkonen-Harjula K, Karmeniemi P, Suikkanen S, Kaaria S, Sipila S, Pitkala K*, et al.* Long-term home-based physiotherapy for older people with signs of frailty-RCT (NCT02305433) [P-229]. *Eur Geriatr Med* 2017;**8**:S105. https://doi.org/10.1016/S1878-7649(17)30179-1
60. Lambotte D, De Donder L, De Roeck EE, Hoeyberghs LJ, van der Vorst A, Duppen D*, et al.* Randomized controlled trial to evaluate a prevention program for frail community-dwelling

older adults: a D-SCOPE protocol. *BMC Geriatr* 2018;**18**:194. https://doi.org/10.1186/s12877-018-0875-3

61. Leung AC-t, Liu C-p, Chow NW-s, Chi I. Cost-Benefit Analysis of a Case Management Project for the Community-Dwelling Frail Elderly in Hong Kong. *J Appl Gerontol* 2004;**23**:70–85. https://doi.org/10.1177/0733464804263088
62. Leveille SG, Wagner EH, Davis C, Grothaus L, Wallace J, LoGerfo M*, et al.* Preventing disability and managing chronic illness in frail older adults: a randomized trial of a community-based partnership with primary care. *J Am Geriatr Soc* 1998;**46**:1191–8. https://doi.org/10.1111/j.1532-5415.1998.tb04533.x
63. Lewin G, De San Miguel K, Knuiman M, Alan J, Boldy D, Hendrie D*, et al.* A randomised controlled trial of the Home Independence Program, an Australian restorative home-care programme for older adults. *Health Soc Care Community* 2013;**21**:69–78. https://doi.org/10.1111/j.1365-2524.2012.01088.x
64. Liddle J, March L, Carfrae B, Finnegan T, Druce J, Schwarz J*, et al.* Can occupational therapy intervention play a part in maintaining independence and quality of life in older people? A randomised controlled trial. *Aust N Z J Public Health* 1996;**20**:574–8. https://doi.org/10.1111/j.1467-842x.1996.tb01068.x
65. Liimatta H, Lampela P, Laitinen-Parkkonen P, Pitkala KH. Effects of preventive home visits on health-related quality-of-life and mortality in home-dwelling older adults. *Scand J Prim Health Care* 2019;**37**:90–7. https://doi.org/10.1080/02813432.2019.1569372
66. Loh DA, Hairi NN, Choo WY, Mohd Hairi F, Peramalah D, Kandiben S*, et al.* MultiComponent Exercise and theRApeutic lifeStyle (CERgAS) intervention to improve physical performance and maintain independent living among urban poor older people--a cluster randomised controlled trial. *BMC Geriatr* 2015;**15**:8. https://doi.org/10.1186/s12877-015-0002-7
67. Lood Q, Gustafsson S, Dahlin Ivanoff S. Bridging barriers to health promotion: a feasibility pilot study of the 'Promoting Aging Migrants' Capabilities study'. *J Eval Clin Pract* 2015;**21**:604–13. https://doi.org/10.1111/jep.12345
68. Mann J, Thompson F, McDermott R, Esterman A, Strivens E. Impact of an integrated community-based model of care for older people with complex conditions on hospital emergency presentations and admissions: a step-wedged cluster randomized trial. *BMC Health Serv Res* 2021;**21**:701. https://doi.org/10.1186/s12913-021-06668-x
69. Mann WC, Ottenbacher KJ, Fraas L, Tomita M, Granger CV. Effectiveness of assistive technology and environmental interventions in maintaining independence and reducing home care costs for the frail elderly. A randomized controlled trial. *Arch Fam Med* 1999;**8**:210–7. https://doi.org/10.1001/archfami.8.3.210
70. Markle-Reid M, Weir R, Browne G, Roberts J, Gafni A, Henderson S. Health promotion for frail older home care clients. *J Adv Nurs* 2006;**54**:381–95. https://doi.org/10.1111/j.1365-2648.2006.03817.x
71. Melis RJ, van Eijken MI, Teerenstra S, van Achterberg T, Parker SG, Borm GF*, et al.* A randomized study of a multidisciplinary program to intervene on geriatric syndromes in vulnerable older people who live at home (Dutch EASYcare Study). *J Gerontol A Biol Sci Med Sci* 2008;**63**:283–90. https://doi.org/10.1093/gerona/63.3.283
72. Meng H, Friedman B, Wamsley BR, Mukamel D, Eggert GM. Effect of a consumer-directed voucher and a disease-management-health-promotion nurse intervention on home care use. *Gerontologist* 2005;**45**:167–76. https://doi.org/10.1093/geront/45.2.167
73. Messens L, Quinn S, Saez I, Cuidad Mas MJ, Squillace P, Laura A-G. *Health monitoring and sOcial integration environMent for Supporting WidE ExTension of independent life at HOME (Home Sweet Home): Final Trial Evaluation Report* no. D7.5. Antwerp: Zorgbedrijf Antwerpen; 2014.

74. Metzelthin SF, Van Rossum E, De Witte LP, Ambergen AW, Hobma SO, Sipers W*, et al.* Effectiveness of interdisciplinary primary care approach to reduce disability in community dwelling frail older people: Cluster randomised controlled trial. *BMJ* 2013;**347**:f5264. https://doi.org/10.1136/bmj.f5264
75. Moll van Charante EP, Richard E, Eurelings LS, van Dalen JW, Ligthart SA, van Bussel EF*, et al.* Effectiveness of a 6-year multidomain vascular care intervention to prevent dementia (preDIVA): a cluster-randomised controlled trial. *Lancet* 2016;**388**:797–805. https://doi.org/10.1016/S0140-6736(16)30950-3
76. Monteserin Nadal R, Altimir Losada S, Brotons Cuixart C, Padros Selma J, Santaeugenia Gonzalez S, Moral Pelaez I*, et al.* Randomized clinical trial on the efficacy of global geriatric assessment in primary care. [Spanish]. *Rev Esp Geriatr Gerontol* 2008;**43**:5–12. https://doi.org/10.1016/s0211-139x(08)71144-2
77. Morey MC, Ekelund C, Pearson M, Crowley G, Peterson M, Sloane R*, et al.* Project LIFE: a partnership to increase physical activity in elders with multiple chronic illnesses. *J Aging Phys Act* 2006;**14**:324–43. https://doi.org/10.1123/japa.14.3.324
78. Morey MC, Peterson MJ, Pieper CF, Sloane R, Crowley GM, Cowper PA*, et al.* The Veterans Learning to Improve Fitness and Function in Elders Study: a randomized trial of primary care–based physical activity counseling for older men. *J Am Geriatr Soc* 2009;**57**:1166–74. https://doi.org/10.1111/j.1532-5415.2009.02301.x
79. Morgan GS, Haase AM, Campbell RM, Ben-Shlomo Y. A pilot randomised controlled trial of physical activity facilitation for older adults: feasibility study findings. *Pilot Feasibility Stud* 2019;**5**:40. https://doi.org/10.1186/s40814-019-0414-9
80. Newbury JW, Marley JE, Beilby JJ. A randomised controlled trial of the outcome of health assessment of people aged 75 years and over. *Med J Aust* 2001;**175**:104–7. https://doi.org/10.5694/j.1326-5377.2001.tb143541.x
81. Newcomer R, Maravilla V, Faculjak P, Graves MT. Outcomes of preventive case management among high-risk elderly in three medical groups: a randomized clinical trial. *Eval Health Prof* 2004;**27**:323–48. https://doi.org/10.1177/0163278704270011
82. Ng TP, Feng L, Nyunt MS, Feng L, Niti M, Tan BY*, et al.* Nutritional, physical, cognitive, and combination interventions and frailty reversal among older adults: A randomized controlled trial. *Am J Med* 2015;**128**:1225–36.e1. https://doi.org/10.1016/j.amjmed.2015.06.017
83. Parsons J, Rouse P, Robinson EM, Sheridan N, Connolly MJ. Goal setting as a feature of homecare services for older people: does it make a difference? *Age Ageing* 2012;**41**:24–9. https://doi.org/10.1093/ageing/afr118
84. Parsons M, Senior H, Kerse N, Chen MH, Jacobs S, Anderson C. Randomised trial of restorative home care for frail older people in New Zealand. *Nurs Older People* 2017;**29**:27–33. https://doi.org/10.7748/nop.2017.e897
85. Parsons M, Senior H, Kerse N, Chen MH, Jacobs S, Vanderhoorn S*, et al.* Should care managers for older adults be located in primary care? A randomized controlled trial. *J Am Geriatr Soc* 2012;**60**:86–92. https://doi.org/10.1111/j.1532-5415.2011.03763.x
86. Pathy MS, Bayer A, Harding K, Dibble A. Randomised trial of case finding and surveillance of elderly people at home. *Lancet* 1992;**340**:890–3. https://doi.org/10.1016/0140-6736(92)93294-W
87. Phelan EA, Balderson B, Levine M, Erro JH, Jordan L, Grothaus L*, et al.* Delivering effective primary care to older adults: a randomized, controlled trial of the senior resource team at group health cooperative. *J Am Geriatr Soc* 2007;**55**:1748–56. https://doi.org/10.1111/j.1532-5415.2007.01416.x
88. Ploeg J, Brazil K, Hutchison B, Kaczorowski J, Dalby DM, Goldsmith CH*, et al.* Effect of preventive primary care outreach on health related quality of life among older adults at risk

of functional decline: randomised controlled trial. *BMJ* 2010;**340**:c1480. https://doi.org/10.1136/bmj.c1480

89. Profener F, Anders J, Dapp U, Minder CE, Golgert S, von Renteln-Kruse W. Acceptance of preventive home visits among frail elderly persons : Participants an non-participants in a Follow-up after 2 and 4 years within the LUCAS longitudinal study. [German]. *Z Gerontol Geriatr* 2016;**49**:596–605. https://doi.org/10.1007/s00391-016-1127-9
90. Rockwood K, Stadnyk K, Carver D, MacPherson KM, Beanlands HE, Powell C*, et al.* A clinimetric evaluation of specialized geriatric care for rural dwelling, frail older people. *J Am Geriatr Soc* 2000;**48**:1080–5. https://doi.org/10.1111/j.1532-5415.2000.tb04783.x
91. Romera-Liebana L, Orfila F, Segura JM, Real J, Fabra ML, Möller M*, et al.* Effects of a primary care-based multifactorial intervention on physical and cognitive function in frail, elderly individuals: A randomized controlled trial. *J Gerontol A Biol Sci Med Sci* 2018;**73**:1688-74. https://doi.org/10.1093/gerona/glx259
92. Rooijackers TH, Kempen GIJM, Zijlstra GAR, van Rossum E, Koster A, Lima Passos V*, et al.* Effectiveness of a reablement training program for homecare staff on older adults' sedentary behavior: A cluster randomized controlled trial. *J Am Geriatr Soc* 2021;**69**:2566–78. https://doi.org/10.1111/jgs.17286
93. Rubenstein LZ, Alessi CA, Josephson KR, Trinidad Hoyl M, Harker JO, Pietruszka FM. A randomized trial of a screening, case finding, and referral system for older veterans in primary care. *J Am Geriatr Soc* 2007;**55**:166–74. https://doi.org/10.1111/j.1532-5415.2007.01044.x
94. Ryvicker M, Feldman PH, Rosati RJ, Sobolewski S, Maduro GA, Jr., Schwartz T. Improving functional outcomes in home care patients: impact and challenges of disseminating a quality improvement initiative. *J Healthc Qual* 2011;**33**:28–36. https://doi.org/10.1111/j.1945-1474.2011.00156.x
95. Serra-Prat M, Sist X, Domenich R, Jurado L, Saiz A, Roces A*, et al.* Effectiveness of an intervention to prevent frailty in pre-frail community-dwelling older people consulting in primary care: a randomised controlled trial. *Age Ageing* 2017;**46**:401-7. https://doi.org/10.1093/ageing/afw242
96. Shapiro A, Taylor M. Effects of a community-based early intervention program on the subjective well-being, institutionalization, and mortality of low-income elders. *Gerontologist* 2002;**42**:334–41. https://doi.org/10.1093/geront/42.3.334
97. Sherman H, Soderhielm-Blid S, Forsberg C, Karp A, Tornkvist L. Effects of preventive home visits by district nurses on self-reported health of 75-year-olds. *Prim Health Care Res Dev* 2016;**17**:56–71. https://doi.org/10.1017/S1463423614000565
98. Siemonsma PC, Blom JW, Hofstetter H, van Hespen ATH, Gussekloo J, Drewes YM*, et al.* The effectiveness of functional task exercise and physical therapy as prevention of functional decline in community dwelling older people with complex health problems. *BMC Geriatr* 2018;**18**:164. https://doi.org/10.1186/s12877-018-0859-3
99. Stewart S, Harvey I, Poland F, Lloyd-Smith W, Mugford M, Flood C. Are occupational therapists more effective than social workers when assessing frail older people? Results of CAMELOT, a randomised controlled trial. *Age Ageing* 2005;**34**:41–6. https://doi.org/10.1093/ageing/afh230
100. Stuck AE, Aronow HU, Steiner A, Alessi CA, Bula CJ, Gold MN*, et al.* A trial of annual in-home comprehensive geriatric assessments for elderly people living in the community. *N Engl J Med* 1995;**333**:1184–9. https://doi.org/10.1056/NEJM199511023331805
101. Stuck AE, Minder CE, Peter-Wuest I, Gillmann G, Egli C, Kesselring A*, et al.* A randomized trial of in-home visits for disability prevention in community-dwelling older people at low and high risk for nursing home admission. *Arch Intern Med* 2000;**160**:977–86. https://doi.org/10.1001/archinte.160.7.977

102. Stuck AE, Moser A, Morf U, Wirz U, Wyser J, Gillmann G*, et al.* Effect of health risk assessment and counselling on health behaviour and survival in older people: a pragmatic randomised trial. *PLoS Med* 2015;**12**:e1001889. https://doi.org/10.1371/journal.pmed.1001889
103. Suijker JJ, van Rijn M, Buurman BM, Ter Riet G, Moll van Charante EP, de Rooij SE. Effects of nurse-led multifactorial care to prevent disability in community-living older people: Cluster randomized trial. *PLoS One* 2016;**11**:e0158714. https://doi.org/10.1371/journal.pone.0158714
104. Szanton SL, Thorpe RJ, Boyd C, Tanner EK, Leff B, Agree E*, et al.* Community aging in place, advancing better living for elders: a bio-behavioral-environmental intervention to improve function and health-related quality of life in disabled older adults. *J Am Geriatr Soc* 2011;**59**:2314–20. https://doi.org/10.1111/j.1532-5415.2011.03698.x
105. Szanton SL, Xue QL, Leff B, Guralnik J, Wolff JL, Tanner EK*, et al.* Effect of a biobehavioral environmental approach on disability among low-income older adults: A randomized clinical trial. *JAMA Intern Med* 2019;**179**:204–11. https://doi.org/10.1001/jamainternmed.2018.6026
106. Takahashi PY, Pecina JL, Upatising B, Chaudhry R, Shah ND, Van Houten H*, et al.* A randomized controlled trial of telemonitoring in older adults with multiple health issues to prevent hospitalizations and emergency department visits. *Arch Intern Med* 2012;**172**:773–9. https://doi.org/10.1001/archinternmed.2012.256
107. Teut M, Schnabel K, Baur R, Kerckhoff A, Reese F, Pilgram N*, et al.* Effects and feasibility of an Integrative Medicine program for geriatric patients-a cluster-randomized pilot study. *Clin Interv Aging* 2013;**8**:953-61. https://doi.org/10.2147/CIA.S45242
108. Thiel C, Braun T, Grüneberg C. Physical training as core component of multimodal treatment of older frail people-study protocol of a randomized controlled pilot study. *Z Gerontol Geriatr* 2019;**52**:45-60. https://doi.org/10.1007/s00391-018-1443-3
109. Thomas R, Worrall G, Elgar F, Knight J. Can they keep going on their own? A four-year randomized trial of functional assessments of community residents. *Can J Aging* 2007;**26**:379–90. https://doi.org/10.3138/cja.26.4.379
110. Tomita MR, Mann WC, Stanton K, Tomita AD, Sundar V. Use of currently available smart home technology by frail elders: process and outcomes. *Top Geriatr Rehabil* 2007;**23**:24–34. https://doi.org/10.1097/00013614-200701000-00005
111. Tulloch AJ, Moore V. A randomized controlled trial of geriatric screening and surveillance in general practice. *J R Coll Gen Pract* 1979;**29**:733–40.
112. Tuntland H, Aaslund MK, Espehaug B, Førland O, Kjeken I. Reablement in community-dwelling older adults: a randomised controlled trial. *BMC Geriatr* 2015;**15**:1–11. https://doi.org/10.1186/s12877-015-0142-9
113. van der Pols-Vijlbrief R, Wijnhoven HAH, Bosmans JE, Twisk JWR, Visser M. Targeting the underlying causes of undernutrition. Cost-effectiveness of a multifactorial personalized intervention in community-dwelling older adults: A randomized controlled trial. *Clin Nutr* 2017;**36**:1498–508. https://doi.org/10.1016/j.clnu.2016.09.030
114. van Dongen EJ, Haveman-Nies A, Doets EL, Dorhout BG, de Groot LC. Effectiveness of a diet and resistance exercise intervention on muscle health in older adults: ProMuscle in Practice. *J Am Med Dir Assoc* 2020;**21**:1065–72. https://doi.org/10.1016/j.jamda.2019.11.026
115. van Heuvelen MJ, Hochstenbach JB, Brouwer WH, de Greef MH, Zijlstra GA, van Jaarsveld E*, et al.* Differences between participants and non-participants in an RCT on physical activity and psychological interventions for older persons. *Aging Clin Exp Res* 2005;**17**:236-45. https://doi.org/10.1007/BF03324603
116. van Hout HP, Jansen AP, van Marwijk HW, Pronk M, Frijters DF, Nijpels G. Prevention of adverse health trajectories in a vulnerable elderly population through nurse home visits: a

randomized controlled trial [ISRCTN05358495]. *J Gerontol A Biol Sci Med Sci* 2010;**65**:734–42. https://doi.org/10.1093/gerona/glq037
117. van Leeuwen KM, Bosmans JE, Jansen AP, Hoogendijk EO, Muntinga ME, van Hout HP*, et al.* Cost-effectiveness of a chronic care model for frail older adults in primary care: Economic evaluation alongside a stepped-wedge cluster-randomized trial. *J Am Geriatr Soc* 2015;**63**:2494–504. https://doi.org/10.1111/jgs.13834
118. van Lieshout MRJ, Bleijenberg N, Schuurmans MJ, de Wit NJ. The effectiveness of a PRoactive multicomponent intervention program on disability in independently living older people: A randomized controlled trial. *J Nutr Health Aging* 2018;**22**:1051–9. https://doi.org/10.1007/s12603-018-1101-x
119. van Rossum E, Frederiks CM, Philipsen H, Portengen K, Wiskerke J, Knipschild P. Effects of preventive home visits to elderly people. *BMJ* 1993;**307**:27–32. https://doi.org/10.1136/bmj.307.6895.27
120. Vass M, Avlund K, Lauridsen J, Hendriksen C. Feasible model for prevention of functional decline in older people: municipality-randomized, controlled trial. *J Am Geriatr Soc* 2005;**53**:563–8. https://doi.org/10.1111/j.1532-5415.2005.53201.x
121. Vetter NJ, Jones DA, Victor CR. Effect of health visitors working with elderly patients in general practice: a randomised controlled trial. *Br Med J (Clin Res Ed)* 1984;**288**:369–72. https://doi.org/10.1136/bmj.288.6414.369
122. von Bonsdorff MB, Leinonen R, Kujala UM, Heikkinen E, Törmäkangas T, Hirvensalo M*, et al.* Effect of physical activity counseling on disability in older people: A 2-year randomized controlled trial. *J Am Geriatr Soc* 2008;**56**:2188–94. https://doi.org/10.1111/j.1532-5415.2008.02000.x
123. Wallace JI, Buchner DM, Grothaus L, Leveille S, Tyll L, LaCroix AZ*, et al.* Implementation and effectiveness of a community-based health promotion program for older adults. *J Gerontol A Biol Sci Med Sci* 1998;**53**:M301–6. https://doi.org/10.1093/gerona/53a.4.m301
124. Walters K, Frost R, Kharicha K, Avgerinou C, Gardner B, Ricciardi F*, et al.* Home-based health promotion for older people with mild frailty: the HomeHealth intervention development and feasibility RCT. *Health Technol Assess* 2017;**21**:1–128. https://doi.org/10.3310/hta21730
125. Whitehead PJ, Walker MF, Parry RH, Latif Z, McGeorge ID, Drummond AE. Occupational Therapy in HomEcare Re-ablement Services (OTHERS): results of a feasibility randomised controlled trial. *BMJ Open* 2016;**6**:e011868. https://doi.org/10.1136/bmjopen-2016-011868
126. Williams EI, Greenwell J, Groom LM. The care of people over 75 years old after discharge from hospital: an evaluation of timetabled visiting by Health Visitor Assistants. *J Public Health Med* 1992;**14**:138–44. https://doi.org/10.1093/oxfordjournals.pubmed.a042711
127. Wolter A, Stolle C, Roth G, Rothgang H. Does the resident care assessment instrument improve long-term home care? - results of a nation-wide study in Germany. [German]. *Gesundheitswesen* 2013;**75**:29–32. https://doi.org/10.1055/s-0032-1309013
128. Wong AKC, Wong FKY, Chang K. Effectiveness of a community-based self-care promoting program for community-dwelling older adults: A randomized controlled trial. *Age Ageing* 2019;**48**:852–8. https://doi.org/10.1093/ageing/afz095
129. Yamada Y, Ikegami N. Preventive home visits for community- dwelling frail elderly people based on minimum data set-home care: randomized controlled trial. *Geriatr Gerontol Int* 2003;**3**:236–42. https://doi.org/10.1111/j.1444-1586.2003.00103.x
130. NCT02317432. *Building Community Capacity for Disability Prevention for Minority Elders (Positive Minds - Strong Bodies)*. US National Library of Medicine: ClinicalTrials.gov; 2014. URL: https://clinicaltrials.gov/ct2/show/NCT02317432 (Accessed 19 May 2020).
131. Porteny T, Alegría M, del Cueto P, Fuentes L, Markle SL, NeMoyer A*, et al.* Barriers and strategies for implementing community-based interventions with minority elders: positive minds-strong bodies. *Implement Sci Commun* 2020;**1**:1–13.

132. Arthanat S, Vroman KG, Lysack C. A home-based individualized information communication technology training program for older adults: a demonstration of effectiveness and value. *Disabil Rehabil Assist Technol* 2016;**11**:316–24.
133. Arthanat S, Vroman KG, Lysack C, Grizzetti J. Multi-stakeholder perspectives on information communication technology training for older adults: implications for teaching and learning. *Disabil Rehabil Assist Technol* 2019;**14**:453–61.
134. Auvinen K, Raisanen J, Merikoski M, Mantyla A, Kumpusalo-Vauhkonen A, Enlund H*, et al.* The Finnish Interprofessional Medication Assessment (FIMA): baseline findings from home care setting. *Aging Clin Exp Res* 2019;**31**:1471–9.
135. Merikoski M, Auvinen K, Kumpusalo-Vauhkonen A, Liukkonen T, Lämsä E, Lönroos E*, et al.* *Iäkkäiden Lääkehoidon Moniammatillinen Arviointi (ILMA)*. Helsinki: Sosiaali-ja Terveysministeriö; 2017. URL: http://urn.fi/URN:ISBN:978-952-00-3884-7 (Accessed 1 December 2022).
136. NCT02398812. *The Interprofessional Medication Assessment for Older Patients*. US National Library of Medicine: ClinicalTrials.gov; 2015. URL: https://clinicaltrials.gov/ct2/show/NCT02398812 (Accessed 28 April 2020).
137. Gustafsson S, Berglund H, Faronbi J, Barenfeld E, Ottenvall Hammar I. Minor positive effects of health-promoting senior meetings for older community-dwelling persons on loneliness, social network, and social support. *Clin Interv Aging* 2017;**12**:1867–77.
138. Arola A, Dahlin-Ivanoff S, Haggblom-Kronlof G. Impact of a person-centred group intervention on life satisfaction and engagement in activities among persons aging in the context of migration. *Scand J Occup Ther* 2019:1–11.
139. Gustafsson S, Lood Q, Wilhelmson K, Haggblom-Kronlof G, Landahl S, Dahlin-Ivanoff S. A person-centred approach to health promotion for persons 70+ who have migrated to Sweden: promoting aging migrants' capabilities implementation and RCT study protocol. *BMC Geriatr* 2015;**15**:10.
140. NCT01841853. *RCT of Health-promoting Intervention for Older Foreign-born Adults*. US National Library of Medicine: ClinicalTrials.gov; 2013. URL: https://clinicaltrials.gov/ct2/show/NCT01841853 (Accessed 28 April 2020).
141. Coleston-Shields DM. Integration of medical and social services for elderly people in the community reduced costs and use of health services. *Evidence Based Mental Health* 1998;**1**:106. https://doi.org/10.1136/ebmh.1.4.106
142. Britian O. Integration of services for elderly people reduced costs and use of health services [commentary on Bernabei R, Landi F, Gambassi G et al. Randomized trial of impact of model of integrated care and case management for older people living in the community. BR MED J 1998;316(7141):1348-51]. *Evidence Based Nursing* 1999:20–.
143. Bleijenberg N, Drubbel I, Ten Dam VH, Numans ME, Schuurmans MJ, de Wit NJ. Proactive and integrated primary care for frail older people: design and methodological challenges of the Utrecht primary care PROactive frailty intervention trial (U-PROFIT). *BMC Geriatr* 2012;**12**:16.
144. Bleijenberg N, ten Dam VH, Drubbel I, Numans ME, de Wit NJ, Schuurmans MJ. Development of a proactive care program (U-CARE) to preserve physical functioning of frail older people in primary care. *J Nurs Scholarsh* 2013;**45**:230–7.
145. Bleijenberg N, ten Dam VH, Drubbel I, Numans ME, Wit NJ, Schuurmans MJ. Treatment fidelity of an evidence-based nurse-led intervention in a proactive primary care program for older people. *Worldviews Evid Based Nurs* 2016;**13**:75–84.
146. Bleijenberg N, Ten Dam VH, Steunenberg B, Drubbel I, Numans ME, De Wit NJ*, et al.* Exploring the expectations, needs and experiences of general practitioners and nurses towards a proactive and structured care programme for frail older patients: a mixed-methods study. *J Adv Nurs* 2013;**69**:2262–73.

147. Laan W, Zuithoff NP, Drubbel I, Bleijenberg N, Numans ME, de Wit NJ, *et al.* Validity and reliability of the Katz-15 scale to measure unfavorable health outcomes in community-dwelling older people. *J Nutr Health Aging* 2014;**18**:848–54.
148. NTR2288. *Central Utrecht Elderly Care Project 'Om U': "Somebody who knows what's going on and can see things from my side."*. World Health Organization: International Clinical Trials Registry Platform; 2010. URL: https://trialsearch.who.int/Trial2.aspx?TrialID=NTR2288 (Accessed 11 May 2020).
149. ten Dam VH, Bleijenberg N, Numans ME, Drubbel I, Schuurmans MJ, de Wit NJ. [Proactive and structured care for the elderly in primary care]. [Dutch]. *Tijdschr Gerontol Geriatr* 2013;**44**:81–9.
150. Metzelthin SF, Bleijenberg N, Blom JW, Imhof L. Primary care strategies to maintain independence of frail older people: looking for evidence across borders. *Eur Geriatr Med* 2014;**5**:S31-S2.
151. Bleijenberg N, Imhof L, Mahrer-Imhof R, Wallhagen MI, de Wit NJ, Schuurmans MJ. Patient Characteristics Associated With a Successful Response to Nurse-Led Care Programs Targeting the Oldest-Old: A Comparison of Two RCTs. *Worldviews Evid Based Nurs* 2017;**14**:210–22.
152. NL1836. *Integrated Systematic Care for Older PEople (ISCOPE)*. Netherlands National Trial Register; 2009. URL: https://www.trialregister.nl/trial/1836 (Accessed 28 April 2020).
153. van Blijswijk SCE, Blom JW, de Craen AJM, den Elzen WPJ, Gussekloo J. Prediction of functional decline in community-dwelling older persons in general practice: a cohort study. *BMC Geriatr* 2018;**18**:1–9.
154. ISRCTN66679751. *Implementation of an internet-based instrument, the 'Eigen Kracht Wijzer' in older people*. BioMed Central: ISRCTN Registry; 2013. URL: https://doi.org/10.1186/ISRCTN66679751 (Accessed 11 May 2020).
155. Botjes E. *EigenKrachtWijzer*. Netherland: Movisie; 2013. URL: https://www.movisie.nl/interventie/eigenkrachtwijzer (Accessed 17 May 2020).
156. Nicolaides-Bouman A, van Rossum E, Kempen GI, Knipschild P. Effects of home visits by home nurses to elderly people with health problems: design of a randomised clinical trial in the Netherlands [ISRCTN92017183]. *BMC Health Serv Res* 2004;**4**:35.
157. Nicolaides-Bouman A, van Rossum E, Habets H, Kempen GI, Knipschild P. Home visiting programme for older people with health problems: process evaluation. *J Adv Nurs* 2007;**58**:425–35.
158. ISRCTN92017183. *Effects of home visits by home nurses to elderly people with health problems*. BioMed Central: ISRCTN Registry; 2004. URL: http://www.isrctn.com/ISRCTN92017183 (Accessed 28 April 2020).
159. Bouman AIE, Van Rossum E, Ambergen TW, Kempen GIJM, Knipschild PG. House calls to elderly with health problems: Effects of a random experiment. [Dutch]. *Ned Tijdschr Geneeskd* 2009;**153**:644–50.
160. Bouman A, van Rossum E, Evers S, Ambergen T, Kempen G, Knipschild P. Effects on health care use and associated cost of a home visiting program for older people with poor health status: a randomized clinical trial in the Netherlands. *J Gerontol A Biol Sci Med Sci* 2008;**63**:291–7.
161. Brettschneider C, Luck T, Fleischer S, Roling G, Beutner K, Sesselmann Y, *et al.* Cost-utility analysis of preventive home visits in older adults. *Value Health* 2014;**17**:A511.
162. Fleischer S, Roling G, Beutner K, Hanns S, Behrens J, Luck T, *et al.* Growing old at home - A randomized controlled trial to investigate the effectiveness and cost-effectiveness of preventive home visits to reduce nursing home admissions: Study protocol [NCT00644826]. *BMC Public Health* 2008;**8**:185. https://doi.org/10.1186/1471-2458-8-185

163. Luck T, Roling G, Heinrich S, Luppa M, Matschinger H, Fleischer S. Altern zu Hause-Unterstützung durch präventive Hausbesuche-Eine randomisierte kontrollierte Interventionsstudie. *Hallesche Beitr Gesundh Pflegewissenschaften* 2011;**10**.
164. NCT00644826. *Growing Old at Home*. US National Library of Medicine: ClinicalTrials.gov; 2008. URL: https://clinicaltrials.gov/ct2/show/NCT00644826 (Accessed 28 April 2020).
165. ACTRN12608000250336. *Frailty Intervention Trial*. Australian New Zealand Clinical Trials Registry; 2008. URL: https://www.anzctr.org.au/Trial/Registration/TrialReview.aspx?ACTRN=12608000250336 (Accessed 8 May 2020).
166. ACTRN12608000507381. *Increasing participation. A sub-study of the Frailty Intervention Trial (FIT)*. Australian New Zealand Clinical Trials Registry; 2008. URL: http://www.anzctr.org.au/ACTRN12608000507381.aspx (Accessed 11 May 2020).
167. ACTRN12608000565347. *The impact of a community based multidisciplinary frailty intervention for older people on informal carers' experience of caregiving.* Australian New Zealand Clinical Trials Registry; 2008. URL: https://www.anzctr.org.au/Trial/Registration/TrialReview.aspx?id=83207 (Accessed 12 May 2020).
168. Aggar C, Ronaldson S, Cameron ID. Reactions to caregiving during an intervention targeting frailty in community living older people. *BMC Geriatr* 2012;**12**:66.
169. Fairhall N, Aggar C, Kurrle SE, Sherrington C, Lord S, Lockwood K*, et al.* Frailty Intervention Trial (FIT). *BMC Geriatr* 2008;**8**:27.
170. Fairhall N, Sherrington C, Cameron ID, Kurrle SE, Lord SR, Lockwood K*, et al.* A multifactorial intervention for frail older people is more than twice as effective among those who are compliant: complier average causal effect analysis of a randomised trial. *J Physiother* 2017;**63**:40–4.
171. Fairhall N, Sherrington C, Kurrle SE, Lord SR, Lockwood K, Cameron ID. Effect of a multifactorial interdisciplinary intervention on mobility-related disability in frail older people: randomised controlled trial. *BMC Med* 2012;**10**:120.
172. Fairhall N, Sherrington C, Kurrle SE, Lord SR, Lockwood K, Howard K*, et al.* Economic evaluation of a multifactorial, interdisciplinary intervention versus usual care to reduce frailty in frail older people. *J Am Med Dir Assoc* 2015;**16**:41-8.
173. Fairhall N, Sherrington C, Lord S, Susan K, Cameron ID. A multifactorial interdisciplinary intervention reduces frailty, increases function and is cost-effective in older adults who are frail: randomised controlled trial. *Physiotherapy* 2015;**101**:eS371-eS2. https://doi.org/10.1016/j.physio.2015.03.588
174. Fairhall N, Sherrington C, Lord SR, Kurrle SE, Langron C, Lockwood K*, et al.* Effect of a multifactorial, interdisciplinary intervention on risk factors for falls and fall rate in frail older people: a randomised controlled trial. *Age Ageing* 2013;**43**:616–22.
175. Cesari M, Demougeot L, Vellas B. Multidomain intervention to prevent disability in elders-The MINDED study. *Eur Geriatr Med* 2013;**1**:S84–S5.
176. Cesari MM. *Multidomain Intervention to preveNt Disability in ElDers – MINDED*. Agence Nationale de la Recherche; n.d. URL: https://anr.fr/Project-ANR-11-CHEX-0008 (Accessed 6 November 2020).
177. Fougère B, Aubertin-Leheudre M, Vellas B, Andrieu S, Demougeot L, Cluzan C*, et al.* Clinical research for older adults in rural areas: the MINDED study experience. *Age (Dordr)* 2016;**38**:30.
178. Fougère B, Vellas B, Andrieu S, Demougeot L, Cluzan C, Cesari M. Difficulties encountered and solutions provided in the implementation of a multidisciplinary intervention for the prevention of dependency in the older population in rural areas: the MINDED study. *Geriatr Psychol Neuropsychiatr Vieil* 2015;**13**:259–64.

179. NCT02082171. *Multidomain Intervention to Prevent Disability in Elders*. US National Library of Medicine: ClinicalTrials.gov; 2013. URL: https://ClinicalTrials.gov/show/NCT02082171 (Accessed 11 May 2020).
180. Zengarini E, Ruggiero C, Mecocci P, Vellas B, Cesari M. Fatigue as a clinical sign of biological aging: exploratory analyses from the MINDED project. *Geriatr Gerontol Int* 2016;**16**:533.
181. Clarkson P, Venables D, Hughes J, Burns A, Challis D. Integrated specialist assessment of older people and predictors of care-home admission. *Psychol Med* 2006;**36**:1011–21.
182. Azen SP, Palmer JM, Carlson M, Mandel D, Cherry BJ, Fanchiang SP*, et al.* Psychometric properties of a Chinese translation of the SF-36 health survey questionnaire in the Well Elderly Study. *J Aging Health* 1999;**11**:240–51.
183. Clark F, Azen SP, Carlson M, Mandel D, LaBree L, Hay J*, et al.* Embedding health-promoting changes into the daily lives of independent-living older adults: long-term follow-up of occupational therapy intervention. *J Gerontol B Psychol Sci Soc Sci* 2001;**56**:P60–3.
184. Clark FA, Carlson M, Jackson J, Mandel D. Lifestyle redesign improves health and is cost-effective. *OT Practice* 2003;**8**:9–13.
185. Hay J, LaBree L, Luo R, Clark F, Carlson M, Mandel D*, et al.* Cost-effectiveness of preventive occupational therapy for independent-living older adults. *J Am Geriatr Soc* 2002;**50**:1381–8.
186. Jackson J, Carlson M, Mandel D, Zemke R, Clark F. Occupation in lifestyle redesign: the Well Elderly Study Occupational Therapy Program. *Am J Occup Ther* 1998;**52**:326–36.
187. Carlson M, Jackson J, Mandel D, Blanchard J, Holguin J, Lai M-Y*, et al.* Predictors of retention among African American and Hispanic older adult research participants in the Well Elderly 2 randomized controlled trial. *J Appl Gerontol* 2014;**33**:357–82.
188. Jackson J, Mandel D, Blanchard J, Carlson M, Cherry B, Azen S*, et al.* Confronting challenges in intervention research with ethnically diverse older adults: The USC Well Elderly II Trial. *Clin Trials* 2009;**6**:90–101.
189. NCT00786344. *USC Well Elderly Study 2*. US National Library of Medicine: ClinicalTrials.gov; 2008. URL: https://clinicaltrials.gov/ct2/show/NCT00786344 (Accessed 21 August 2020).
190. Schelly D, Ohl A, Nadres R. Dosage and Efficacy in Behavioral Interventions With Community Dwelling Older Adults: Lifestyle Redesign Revisited. *J Appl Gerontol* 2021;**40**:1087–95. https://doi.org/10.1177/0733464820911335
191. Seidle JS. The Impact of Response to Stressful Events on Participation in Meaningful Activity: A Secondary Data Analysis Using The Well Elderly 2 Study. *Arch Phys Med Rehabil* 2020;**101 (12)**:e126. https://doi.org/10.1016/j.apmr.2020.10.004
192. Sonnenfeld M, Karmarkar A. Cortisol as a biomarker in rehabilitation services for community dwelling older adults. *Arch Phys Med Rehabil* 2019;**100**:e13.
193. Bielaszka-DuVernay C. Innovation profile: The 'GRACE' model: In-home assessments lead to better care for dual eligibles. *Health Affairs* 2011;**30**:431–4.
194. Counsell SR, Callahan CM, Buttar AB, Clark DO, Frank KI. Geriatric Resources for Assessment and Care of Elders (GRACE): a new model of primary care for low-income seniors. *J Am Geriatr Soc* 2006;**54**:1136–41.
195. Counsell SR, Callahan CM, Tu W, Stump TE, Arling GW. Cost analysis of the Geriatric Resources for Assessment and Care of Elders care management intervention. *J Am Geriatr Soc* 2009;**57**:1420–6.
196. Iloabuchi TC, Mi D, Tu W, Counsell SR. Risk Factors for Early Hospital Readmission in Low-Income Elderly Adults. *J Am Geriatr Soc* 2014;**62**:489–94. https://doi.org/10.1111/jgs.12688
197. NCT00182962. *GRACE: Geriatric Resources for Assessment and Care of Elders*. US National Library of Medicine: ClinicalTrials.gov; 2005. URL: https://clinicaltrials.gov/ct2/show/NCT00182962 (Accessed 21 August 2020).

198. NCT00985283. *Feasibility and Effects of Preventive Home Visits for Older Adults*. US National Library of Medicine: ClinicalTrials.gov; 2009. URL: https://clinicaltrials.gov/ct2/show/NCT00985283 (Accessed 4 August 2020).
199. Dalby DM, Sellors JW, Fraser FD, Fraser C, van Ineveld CH, Pickard L*, et al.* Screening seniors for risk of functional decline: results of a survey in family practice. *Can J Public Health* 1999;**90**:133–7. https://doi.org/10.1007/BF03404117
200. Claus E, Willen A, de Craen A, Knops H, Willems C. Protocol development for individual occupational therapy in the oldest old [article in Dutch]. *Ned Tijdschr Ergotherapie* 2003;**31**:83–6.
201. De Craen AJM, Westendorp RGJ, Willems CG, Buskens ICM, Gussekloo J. Assistive devices and community-based services among 85-year-old community-dwelling elderly in The Netherlands: Ownership, use,and need for intervention. *Disabil Rehabil Assist Technol* 2006;**1**:199–203. https://doi.org/10.1080/17483100612331392835
202. NCT00278096. *Randomised Controlled Trial of Unsolicited Occupational Therapy in Community-Dwelling Elderly*. US National Library of Medicine: ClinicalTrials.gov; 2006. URL: https://clinicaltrials.gov/show/NCT00278096 (Accessed 11 May 2020).
203. NCT01358032. *Evaluating an in-home multicomponent program to manage concerns about falling in frail community dwelling older people*. https://ClinicalTrials.gov/show/; 2009. URL: https://clinicaltrials.gov/ct2/show/NCT01358032 (Accessed 11 May 2020).
204. Dorresteijn TA, Zijlstra G, Van Haastregt JC, Vlaeyen JW, Kempen GI. Feasibility of a nurse-led in-home cognitive behavioral program to manage concerns about falls in frail older people: A process evaluation. *Res Nurs Health* 2013;**36**:257–70.
205. Evers S, Dorresteijn TAC, Wijnen BFM, van Haastregt JCM, Kempen G, Zijlstra GAR. Economic evaluation of a home-based programme to reduce concerns about falls in frail, independently-living older people. *Expert Rev Pharmacoecon Outcomes Res* 2020;**20**:641–51. https://doi.org/10.1080/14737167.2019.1666714
206. Dorresteijn TA, Zijlstra GA, Delbaere K, van Rossum E, Vlaeyen JW, Kempen GI. Evaluating an in-home multicomponent cognitive behavioural programme to manage concerns about falls and associated activity avoidance in frail community-dwelling older people: Design of a randomised control trial [NCT01358032]. *BMC Health Serv Res* 2011;**11**:228.
207. Dupuy L, Consel C, Sauzéon H. Self determination-based design to achieve acceptance of assisted living technologies for older adults. *Comput Human Behav* 2016;**65**:508–21. https://doi.org/10.1016/j.chb.2016.07.042
208. ACTRN12613000043730. *Pre-FIT: a multifactorial interdisciplinary treatment program for older people who are pre-frail*. Australian New Zealand Clinical Trials Registry; 2013. URL: https://www.anzctr.org.au/Trial/Registration/TrialReview.aspx?ACTRN=12613000043730 (Accessed 16 November 2020).
209. Cameron I, Fairhall N, John B, Lockwood K, Monaghan N, Sherrington C*, et al.* A multifactorial interdisciplinary intervention in pre-frail older people: randomised trial. *Journal of Innovation in Aging* 2017;**1**:196.
210. Rowan NL, Gillette PD, Faul AC, Yankeelov PA, Borders KW, Deck S*, et al.* Innovative Interdisciplinary Training in and Delivery of Evidence-Based Geriatric Services: Creating a Bridge With Social Work and Physical Therapy. *Gerontol Geriatr Educ* 2009;**30**:187–204. https://doi.org/10.1080/02701960903133448
211. Arija V, Martín N, Canela T, Anguera C, Castelao AI, García-Barco M*, et al.* Nutrition education intervention for dependent patients: protocol of a randomized controlled trial. *BMC Public Health* 2012;**12**:373. https://doi.org/10.1186/1471-2458-12-373
212. NCT01360775. *Nutritional Education for Dependant Patients (atdom_nut)*. US National Library of Medicine: ClinicalTrials.gov; 2011. URL: https://clinicaltrials.gov/ct2/show/NCT01360775 (Accessed 28 April 2020).

213. AOKN. *Preventive home visits to seniors The prevention program of the AOK Lower Saxony: Getting healthy older Qualitative experience report [German] (Präventive Hausbesuche bei Senioren Das Präventionsprogramm der AOK Niedersachsen: Gesund Älter Werden Qualitativer Erfahrungsbericht)*. Hannover: AOK – Institut für Gesundheitsconsulting; 2010.
214. Katz S, Ford AB, Downs TD, Adams M, Rusby DI. *The effects of continued care: A study of chronic illness in the home*. Rockville, MA: United States Department of Health, Education, and Welfare; 1972.
215. NCT03662945. *Mobile Geriatric Teams: Patient Safety and Healthcare Utilization (MGT)*. US National Library of Medicine: ClinicalTrials.gov; 2018. URL: https://clinicaltrials.gov/ct2/show/NCT03662945 (Accessed 6 November 2020).
216. Gill TM, Baker DI, Gottschalk M, Gahbauer EA, Charpentier PA, de Regt PT*, et al.* A prehabilitation program for physically frail community-living older persons. *Arch Phys Med Rehabil* 2003;**84**:394–404.
217. Gill TM, Baker DI, Gottschalk M, Peduzzi PN, Allore H, Van Ness PH. A prehabilitation program for the prevention of functional decline: effect on higher-level physical function. *Arch Phys Med Rehabil* 2004;**85**:1043–9.
218. Gill TM, McGloin JM, Gahbauer EA, Shepard DM, Bianco LM. Two Recruitment Strategies for a Clinical Trial of Physically Frail Community-Living Older Persons. *J Am Geriatr Soc* 2001;**49**:1039–45. https://doi.org/10.1046/j.1532-5415.2001.49206.x
219. Peduzzi P, Guo Z, Marottoli RA, Gill TM, Araujo K, Allore HG. Improved self-confidence was a mechanism of action in two geriatric trials evaluating physical interventions. *J Clin Epidemiol* 2007;**60**:94–102.
220. Blackburn NE, Skjodt M, Tully MA, Mc Mullan I, Giné-Garriga M, Caserotti P*, et al.* Older Adults' Experiences of a Physical Activity and Sedentary Behaviour Intervention: A Nested Qualitative Study in the SITLESS Multi-Country Randomised Clinical Trial. *Int J Environ Res Public Health* 2021;**18**:4730. https://doi.org/10.3390/ijerph18094730
221. Coll-Planas L, Blancafort Alias S, Tully M, Caserotti P, Giné-Garriga M, Blackburn N*, et al.* Exercise referral schemes enhanced by self-management strategies to reduce sedentary behaviour and increase physical activity among community-dwelling older adults from four European countries: protocol for the process evaluation of the SITLESS randomised controlled trial. *BMJ Open* 2019;**9**:e027073. https://doi.org/10.1136/bmjopen-2018-027073
222. SITLESS Consortium. *SITLESS*. 2021. URL: https://sitless.eu/ (Accessed 7 May 2021).
223. Deidda M, Coll-Planas L, Gine-Garriga M, Guerra-Balic M, Roque IFM, Tully MA*, et al.* Cost-effectiveness of exercise referral schemes enhanced by self-management strategies to battle sedentary behaviour in older adults: protocol for an economic evaluation alongside the SITLESS three-armed pragmatic randomised controlled trial. *BMJ Open* 2018;**8**:e022266.
224. Giné-Garriga M, Coll-Planas L, Guerra M, Domingo À, Roqué M, Caserotti P*, et al.* The SITLESS project: exercise referral schemes enhanced by self-management strategies to battle sedentary behaviour in older adults: study protocol for a randomised controlled trial. *Trials* 2017;**18**:221. https://doi.org/10.1186/s13063-017-1956-x
225. NCT02629666. *Exercise Referral Schemes Enhanced by Self-Management Strategies to Battle Sedentary Behaviour (SitLESS)*. US National Library of Medicine: ClinicalTrials.gov; 2015. URL: https://clinicaltrials.gov/ct2/show/NCT02629666 (Accessed 14 May 2020).
226. Tully MA, McMullan I, Blackburn NE, Wilson JJ, Bunting B, Smith L*, et al.* Sedentary behavior, physical activity, and mental health in older adults: An isotemporal substitution model. *Scand J Med Sci Sports* 2020;**30**:1957–65. https://doi.org/10.1111/sms.13762
227. Tully MA, McMullan I, Blackburn NE, Wilson JJ, Coll-Planas L, Deidda M*, et al.* Is Sedentary Behavior or Physical Activity Associated With Loneliness in Older Adults? Results of the European-Wide SITLESS Study. *J Aging Phys Act* 2020;**28**:549–55. https://doi.org/10.1123/japa.2019-0311

228. Wilson JJ, Blackburn NE, O'Reilly R, Kee F, Caserotti P, Tully MA. Association of objective sedentary behaviour and self-rated health in English older adults. *BMC Res Notes* 2019;**12**:12. https://doi.org/10.1186/s13104-019-4050-5
229. Wilson JJ, McMullan I, Blackburn NE, Skjødt M, Caserotti P, Giné-Garriga M*, et al.* Associations of sedentary behavior bouts with community-dwelling older adults' physical function. *Scand J Med Sci Sports* 2021;**31**:153–62. https://doi.org/10.1111/sms.13827
230. Wilson JJ, Skjødt M, McMullan I, Blackburn NE, Giné-Garriga M, Sansano-Nadal O*, et al.* Consequences of Choosing Different Settings When Processing Hip-Based Accelerometry Data From Older Adults: A Practical Approach Using Baseline Data From the SITLESS Study. *J Meas Phys Behav* 2020;**3**:89–99. https://doi.org/10.1123/jmpb.2019-0037 10.1123/jmpb.2019-0037
231. Gitlin LN. Enhancing Quality of Life in Functionally Vulnerable Older Adults: From Randomized Trial to Standard Care: Individuals at any age can learn new strategies to engage in valued activities. *Generations (San Francisco, Calif)* 2010;**34**:84–7.
232. Gitlin LN, Hauck WW, Dennis MP, Schulz R. Depressive Symptoms in Older African-American and White Adults with Functional Difficulties: The Role of Control Strategies. *J Am Geriatr Soc* 2007;**55**:1023–30. https://doi.org/10.1111/j.1532-5415.2007.01224.x
233. Gitlin LN, Hauck WW, Dennis MP, Winter L, Hodgson N, Schinfeld S. Long-term effect on mortality of a home intervention that reduces functional difficulties in older adults: results from a randomized trial. *J Am Geriatr Soc* 2009;**57**:476–81.
234. Gitlin LN, Hauck WW, Winter L, Dennis MP, Schulz R. Effect of an in-home occupational and physical therapy intervention on reducing mortality in functionally vulnerable older people: preliminary findings. *J Am Geriatr Soc* 2006;**54**:950–5.
235. Gitlin LN, Parisi J, Huang J, Winter L, Roth DL. Attachment to Life: Psychometric Analyses of the Valuation of Life Scale and Differences Among Older Adults. *Gerontologist* 2016;**56**:e21–31.
236. Gitlin LN, Winter L, Dennis MP, Hauck WW. Variation in response to a home intervention to support daily function by age, race, sex, and education. *J Gerontol A Biol Sci Med Sci* 2008;**63**:745–50.
237. Gitlin LN, Winter L, Stanley IH. Compensatory Strategies: Prevalence of Use and Relationship to Physical Function and Well-Being. *J Appl Gerontol* 2017;**36**:647–66. https://doi.org/10.1177/0733464815581479
238. Jutkowitz E, Gitlin L, Pizzi LT, Lee EH, Dennis M. Cost-effectiveness of able a functional program to decrease mortality in community-dwelling older adults. *Value Health* 2011;**14**:A108.
239. Jutkowitz E, Gitlin LN, Pizzi LT, Lee E, Dennis MP. Cost effectiveness of a home-based intervention that helps functionally vulnerable older adults age in place at home. *J Aging Res* 2012;**2012**:680265.
240. NCT00249925. *Project ABLE: Advancing Better Living for Elders*. US National Library of Medicine: ClinicalTrials.gov; 2005. URL: https://clinicaltrials.gov/ct2/show/NCT00249925 (Accessed 28 April 2020).
241. Rose KC, Gitlin LN, Dennis MP. Readiness to use compensatory strategies among older adults with functional difficulties. *Int Psychogeriatr* 2010;**22**:1225–39.
242. ACTRN12613000234718. *TRialing Individualised Interventions to prevent FunctionaL decline in at-risk older adults (TRIIFL): A nested randomized controlled trial*. Australian New Zealand Clinical Trials Registry; 2013. URL: http://www.anzctr.org.au/ACTRN12613000234718.aspx (Accessed 11 May 2020).
243. Gustafson DH, Sr., McTavish F, Gustafson DH, Jr., Mahoney JE, Johnson RA, Lee JD*, et al.* The effect of an information and communication technology (ICT) on older adults' quality of life: study protocol for a randomized control trial. *Trials* 2015;**16**:191.

244. NCT02128789. *Bring Communities and Technology Together for Healthy Aging (ElderTree)*. US National Library of Medicine: ClinicalTrials.gov; 2014. URL: https://clinicaltrials.gov/ct2/show/NCT02128789 (Accessed 8 June 2020).
245. Behm L, Eklund K, Wilhelmson K, Ziden L, Gustafsson S, Falk K*, et al.* Health Promotion Can Postpone Frailty: Results from the RCT Elderly Persons in the Risk Zone. *Public Health Nurs* 2016;**33**:303–15.
246. Behm L, Wilhelmson K, Falk K, Eklund K, Ziden L, Dahlin-Ivanoff S. Positive health outcomes following health-promoting and disease-preventive interventions for independent very old persons: long-term results of the three-armed RCT Elderly Persons in the Risk Zone. *Arch Gerontol Geriatr* 2014;**58**:376–83.
247. Dahlin-Ivanoff S, Eklund K, Wilhelmson K, Behm L, Häggblom-Kronlöf G, Zidén L*, et al.* For whom is a health-promoting intervention effective? Predictive factors for performing activities of daily living independently. *BMC Geriatr* 2016;**17**:171–.
248. Dahlin-Ivanoff S, Gosman-Hedstrom G, Edberg AK, Wilhelmson K, Eklund K, Duner A*, et al.* Elderly persons in the risk zone. Design of a multidimensional, health-promoting, randomised three-armed controlled trial for "prefrail" people of 80+ years living at home. *BMC Geriatr* 2010;**10**:27.
249. Gustafsson S, Wilhelmson K, Eklund K, Gosman-Hedstrom G, Ziden L, Kronlof GH*, et al.* Health-promoting interventions for persons aged 80 and older are successful in the short term-results from the randomized and three-armed elderly persons in the risk zone study. *J Am Geriatr Soc* 2012;**60**:447–54.
250. NCT00877058. *Support for frail elderly persons - from prevention to palliation*. US National Library of Medicine: ClinicalTrials.gov; 2008. URL: https://ClinicalTrials.gov/show/NCT00877058 (Accessed 11 May 2020).
251. Wilhelmson K, Eklund K. Positive Effects on Life Satisfaction Following Health-Promoting Interventions for Frail Older Adults: A Randomized Controlled Study. *Health Psychol Res* 2013;**1**:e12.
252. Ziden L, Haggblom-Kronlof G, Gustafsson S, Lundin-Olsson L, Dahlin-Ivanoff S. Physical function and fear of falling 2 years after the health-promoting randomized controlled trial: elderly persons in the risk zone. *Gerontologist* 2014;**54**:387–97.
253. Biddulph JP, Iliffe S, Kharicha K, Harari D, Swift C, Gillmann G*, et al.* Risk factors for depressed mood amongst a community dwelling older age population in England: cross-sectional survey data from the PRO-AGE study. *BMC Geriatr* 2014;**14**:5. https://doi.org/10.1186/1471-2318-14-5
254. Carmaciu C, Iliffe S, Kharicha K, Harari D, Swift C, Gillmann G*, et al.* Health risk appraisal in older people 3: prevalence, impact, and context of pain and their implications for GPs. *Br J Gen Pract* 2007;**57**:630-5.
255. Harari D, Iliffe S, Kharicha K, Stuck AE, Swift CG. Multi-domain health promotion for older people: randomised controlled study of health risk appraisal in primary...British Geriatrics Society: Abstracts of papers presented at the Spring Scientific Meeting, 6-7 April 2006. *Age Ageing* 2006;**35**:i67.
256. Iliffe S, Kharicha K, Carmaciu C, Harari D, Swift C, Gillman G*, et al.* The relationship between pain intensity and severity and depression in older people: exploratory study. *BMC Fam Pract* 2009;**10**:54.
257. Iliffe S, Kharicha K, Harari D, Swift C, Gillmann G, Stuck AE. Health risk appraisal in older people 2: the implications for clinicians and commissioners of social isolation risk in older people. *Br J Gen Pract* 2007;**57**:277–82.
258. Iliffe S, Kharicha K, Harari D, Swift C, Stuck AE. Health risk appraisal for older people in general practice using an expert system: a pilot study. *Health Soc Care Community* 2005;**13**:21–9.

259. Iliffe S, Swift C, Harari D, Kharicha K, Goodman C, Manthorpe J. Health promotion in later life: public and professional perspectives on an expert system for health risk appraisal. *Prim Health Care Res Dev* 2010;**11**:187–96. https://doi.org/10.1017/S1463423609990442
260. Kharicha K, Iliffe S, Harari D, Swift C, Gillmann G, Stuck AE. Health risk appraisal in older people 1: are older people living alone an "at-risk" group? *Br J Gen Pract* 2007;**57**:271–6.
261. Kharicha K, Iliffe S, Harari D, Swift CG, Goodman C, Manthorpe J*, et al.* Feasibility of repeated use of the Health Risk Appraisal for Older people system as a health promotion tool in community-dwelling older people: retrospective cohort study 2001–05. *Age Ageing* 2011;**41**:128–31. https://doi.org/10.1093/ageing/afr126
262. Raymond M, Iliffe S, Kharicha K, Harari D, Swift C, Gillmann G*, et al.* Health risk appraisal for older people 5: self-efficacy in patient-doctor interactions. *Prim Health Care Res Dev* 2011;**12**:348–56.
263. Tsakos G, Sheiham A, Iliffe S, Kharicha K, Harari D, Swift CG*, et al.* The impact of educational level on oral health-related quality of life in older people in London. *Eur J Oral Sci* 2009;**117**:286-92.
264. Blozik E, Wagner JT, Gillmann G, Iliffe S, von Renteln-Kruse W, Lubben J*, et al.* Social network assessment in community-dwelling older persons: results from a study of three European populations. *Aging Clin Exp Res* 2009;**21**:150–7.
265. Stuck AE, Elkuch P, Dapp U, Anders J, Iliffe S, Swift CG. Feasibility and yield of a self-administered questionnaire for health risk appraisal in older people in three European countries. *Age Ageing* 2002;**31**:463–7. https://doi.org/10.1093/ageing/31.6.463
266. Stuck AE, Kharicha K, Dapp U, Anders J, Von Renteln-Kruse W, Meier-Baumgartner H*, et al.* Development, feasibility and performance of a health risk appraisal questionnaire for older persons. *BMC Med Res Methodol* 2007;**7**:1. https://doi.org/10.1186/1471-2288-7-1
267. Stuck AE, Kharicha K, Dapp U, Anders J, Von Renteln-Kruse W, Meier-Baumgartner HP*, et al.* The PRO-AGE study: An international randomised controlled study of health risk appraisal for older persons based in general practice. *BMC Med Res Methodol* 2007;**7**:2. https://doi.org/10.1186/1471-2288-7-2
268. UMIN000031329. *Evaluation of effectiveness of preventive rehabilitation programs for the frail elderly by physical therapist*. University hospital Medical Information Network Center Clinical Trials Registry (UMIN-CTR); 2018. URL: https://upload.umin.ac.jp/cgi-open-bin/ctr/ctr.cgi?function=brows&action=brows&recptno=R000035759&type=summary&language=E (Accessed 4 May 2020).
269. Hay WI, Browne G, Roberts J, Jamieson E. Prospective care of elderly patients in family practice. Part 3: Prevalence of unrecognized treatable health concerns. *Can Fam Physician* 1995;**41**:1695.
270. ACTRN12605000134628. *In-home preventive health assessment and telephone case management for over 75s living alone in independent living units: a cluster randomised controlled trial*. World Health Organization: International Clinical Trials Registry Platform; 2005. URL: https://trialsearch.who.int/Trial2.aspx?TrialID=ACTRN12605000134628 (Accessed 11 May 2020).
271. Hendriksen C. An intervention study among elderly people. Methodological and practical experiences. *Scand J Prim Health Care* 1986;**4**:39–42.
272. Hendriksen C, Lund E, Strømgård E. Use of Social and Health Services by Elderly People during the Terminal 18 Months of Life. *Scand J Soc Med* 1987;**15**:169–74. https://doi.org/10.1177/140349488701500308
273. Hendriksen C, Lund E, Stromgard E. Hospitalization of elderly people. A 3-year controlled trial. *J Am Geriatr Soc* 1989;**37**:117–22.

274. NCT00238836. *Anticipatory & Preventive Team Care (APTCare): At Risk Patients of Family Health Networks*. US National Library of Medicine: ClinicalTrials.gov; 2005. URL: https://clinicaltrials.gov/ct2/show/NCT00238836 (Accessed 28 April 2020).
275. Humbert J, Legault F, Dahrouge S, Halabisky B, Boyce G, Hogg W*, et al.* Integration of nurse practitioners into a family health network. *Can Nurse* 2007;**103**:30–4.
276. Gray D, Armstrong CD, Dahrouge S, Hogg W, Zhang W. Cost-effectiveness of Anticipatory and Preventive multidisciplinary Team Care for complex patients: evidence from a randomized controlled trial. *Can Fam Physician* 2010;**56**:e20–9.
277. Fletcher J, Hogg W, Farrell B, Woodend K, Dahrouge S, Lemelin J*, et al.* Effect of nurse practitioner and pharmacist counseling on inappropriate medication use in family practice. *Can Fam Physician* 2012;**58**:862–8.
278. Dahrouge S, Hogg W, Lemelin J, Liddy C, Legault F. Methods for a study of Anticipatory and Preventive multidisciplinary Team Care in a family practice. *Can Fam Physician* 2010;**56**:e73–83.
279. Holland SK, Greenberg J, Tidwell L, Newcomer R. Preventing disability through community-based health coaching. *J Am Geriatr Soc* 2003;**51**:265–9.
280. Tidwell L, Holland SK, Greenberg J, Malone J, Mullan J, Newcomer R. Community-Based Nurse Health Coaching and Its Effect on Fitness Participation. *Lipincotts Case Manag* 2004;**9**:267-79. https://doi.org/10.1097/00129234-200411000-00006
281. Haighton C, Moffatt S, Howel D, McColl E, Milne E, Deverill M*, et al.* The Do-Well study: protocol for a randomised controlled trial, economic and qualitative process evaluations of domiciliary welfare rights advice for socio-economically disadvantaged older people recruited via primary health care. *BMC Public Health* 2012;**12**:382.
282. Haighton C, Moffatt S, Howel D, Steer M, Becker F, Bryant A*, et al.* Randomised controlled trial with economic and process evaluations of domiciliary welfare rights advice for socioeconomically disadvantaged older people recruited via primary health care (the Do-Well study). *Public Health Res* 2019;**7**:3. https://doi.org/10.3310/phr07030
283. ISRCTN37380518. *Advice on welfare rights for disadvantaged older people recruited via primary health care*. BioMed Central: ISRCTN Registry; 2012. URL: http://isrctn.com/ISRCTN37380518 (Accessed 11 May 2020).
284. Lin SY, Kerse N, McLean C, Moyes SA. Validation of quality of life and functional measures for older people for telephone administration. *J Prim Health Care* 2010;**2**:35–42.
285. Wilkinson-Meyers L, Brown P, McLean C, Kerse N. Met and unmet need for personal assistance among community-dwelling New Zealanders 75 years and over. *Health Soc Care Community* 2014;**22**:317–27.
286. Wham CA, McLean C, Teh R, Moyes S, Peri K, Kerse N. The BRIGHT Trial: what are the factors associated with nutrition risk? *J Nutr Health Aging* 2014;**18**:692-7.
287. Schäfers A, Martini N, Moyes S, Hayman K, Zolezzi M, McLean C*, et al.* Medication use in community-dwelling older people: pharmacoepidemiology of psychotropic utilisation. *J Prim Health Care* 2014;**6**:267–78.
288. McLean C, Kerse N, Moyes SA, Ng T, Lin SY, Peri K. Recruiting older people for research through general practice: the Brief Risk Identification Geriatric Health Tool trial. *Australas J Ageing* 2014;**33**:257–63.
289. ACTRN12606000256572. *A cluster randomised control trial to compare the effects of a restorative home based model versus usual care to improve wellbeing for community dwelling older people*. Australian New Zealand Clinical Trials Registry; 2006. URL: https://www.anzctr.org.au/Trial/Registration/TrialReview.aspx?id=1417 (Accessed 21 August 2020).
290. King AII. *Creating sustainable home care services for older people* [PhD thesis]. Auckland: The University of Auckland; 2010.

291. King AII, Parsons M, Robinson E. A restorative home care intervention in New Zealand: Perceptions of paid caregivers. *Health Soc Care Community* 2012;**20**:70–9.
292. JPRN-UMIN000006463. *Development and effects of preventive home visit program among ambulatory frail older people living at home*. World Health Organization: International Clinical Trials Registry Platform; 2011. URL: https://trialsearch.who.int/Trial2.aspx?TrialID=JPRN-UMIN000006463 (Accessed 11 May 2020).
293. Kono A, Izumi K, Kanaya Y, Tsumura C, Rubenstein LZ. Assessing the quality and effectiveness of an updated preventive home visit programme for ambulatory frail older Japanese people: research protocol for a randomized controlled trial. *J Adv Nurs* 2014;**70**:2363–72.
294. JPRN-UMIN000001113. *Effects of preventive home visits for ambulatory frail elders: a randomized clinical trial in Osaka, Japan*. World Health Organization: International Clinical Trials Registry Platform; 2008. URL: https://trialsearch.who.int/Trial2.aspx?TrialID=JPRN-UMIN000001113 (Accessed 11 May 2020).
295. Kono A, Fujita T, Tsumura C, Kondo T, Kushiyama K, Rubenstein LZ. Preventive home visit model targeted to specific care needs of ambulatory frail elders: preliminary report of a randomized trial design. *Aging Clin Exp Res* 2009;**21**:167–73.
296. Kono A, Kanaya Y, Tsumura C, Rubenstein LZ. Effects of preventive home visits on health care costs for ambulatory frail elders: a randomized controlled trial. *Aging Clin Exp Res* 2013;**25**:575–81.
297. Kukkonen-Harjula K, Karmeniemi P, Suikkanen S, Sipil, Pitkala K, Hupli M. Long-term home-based physiotherapy for older people with signs of frailty or consequent to a hip fracture operation-Design of RCT (NCT02305433). *Eur Geriatr Med* 2016;**7**:S149-.
298. NCT02305433. *Effects of Long-term Intensive Home-based Physiotherapy on Older People With an Operated Hip Fracture or Frailty (RCT)*. US National Library of Medicine: ClinicalTrials.gov; 2014. URL: https://clinicaltrials.gov/show/NCT02305433 (Accessed 11 May 2020).
299. Soukkio P, Suikkanen S, Kaaria S, Kautiainen H, Sipila S, Kukkonen-Harjula K*, et al.* Effects of 12-month home-based physiotherapy on duration of living at home and functional capacity among older persons with signs of frailty or with a recent hip fracture - protocol of a randomized controlled trial (HIPFRA study). *BMC Geriatr* 2018;**18**:232.
300. Suikkanen S, Soukkio P, Pitkala K, Kaaria S, Kautiainen H, Sipila S*, et al.* Older persons with signs of frailty in a home-based physical exercise intervention: baseline characteristics of an RCT. *Aging Clin Exp Res* 2019;**31**:1419–27.
301. Suikkanen SA, Soukkio PK, Aartolahti EM, Kautiainen H, Kaaria SM, Hupli MT*, et al.* Effects of Home-Based Physical Exercise on Days at Home and Cost-Effectiveness in Pre-Frail and Frail Persons: Randomized Controlled Trial. *J Am Med Dir Assoc* 2020;**18**:18. https://doi.org/10.1016/j.jamda.2020.06.005
302. Domènech-Abella J, Switsers L, Mundó J, Dierckx E, Dury S, De Donder L. The association between perceived social and physical environment and mental health among older adults: mediating effects of loneliness. *Aging Ment Health* 2021;**25**:962-8. https://doi.org/10.1080/13607863.2020.1727853
303. Duppen D, Rossi G, Dierckx E, Hoeyberghs L, De Donder L. Focusing on positive outcomes in frailty research: development of a short well-being instrument for older adults (SWIO). *Int Psychogeriatr* 2019;**31**:767–77. https://doi.org/10.1017/S1041610219000401
304. Dury S, Dierckx E, van der Vorst A, Van der Elst M, Fret B, Duppen D*, et al.* Detecting frail, older adults and identifying their strengths: results of a mixed-methods study. *BMC Public Health* 2018;**18**:191. https://doi.org/10.1186/s12889-018-5088-3
305. NCT03168204. *Evaluating the Efficacy of a Detection and Prevention Program for Frail Community-dwelling Older Adults (D-SCOPE)*. US National Library of Medicine:

ClinicalTrials.gov; 2017. URL: https://clinicaltrials.gov/ct2/show/NCT03168204 (Accessed 21 August 2020).

306. Smetcoren A-S, Dury S, den Donder L, Dierckx E. D-SCOPE: towards a positive view on prevention in frail elderly [D-SCOPE: naar een positieve kijk op preventie bij kwetsbare ouderen]. *Geron* 2017;**19**:35–8. https://doi.org/10.1007/s40718-017-0010-0
307. Switsers L, Dierckx E, Domènech-Abella J, De Donder L, Dury S. Negative old-age life events and well-being in later life: the moderating and mediating role of loneliness. *Int Psychogeriatr* 2021;**33**:1265-76. https://doi.org/10.1017/S1041610220004196
308. Van der Elst MCJ, Schoenmakers B, Op het Veld LPM, De Roeck EE, Van der Vorst A, Kempen GIJM*, et al.* Concordances and differences between a unidimensional and multidimensional assessment of frailty: a cross-sectional study. *BMC Geriatr* 2019;**19**:346. https://doi.org/10.1186/s12877-019-1369-7
309. van der Vorst A, Zijlstra GAR, De Witte N, Vogel RGM, Schols JMGA, Kempen GIJM*, et al.* Explaining discrepancies in self-reported quality of life in frail older people: a mixed-methods study. *BMC Geriatr* 2017;**17**:251. https://doi.org/10.1186/s12877-017-0641-y
310. Leung AC, Liu CP, Tsui LL, Li SY, Tang GW, Yau DC*, et al.* The use of the Minimum Data Set. Home Care in a case management project in Hong Kong. *Care Manag J* 2001;**3**:8–13.
311. Phelan EA, Williams B, Penninx BWJH, LoGerfo JP, Leveille SG. Activities of daily living function and disability in older adults in a randomized trial of the Health Enhancement Program. *J Gerontol A Biol Sci Med Sci* 2004;**59**:838–43.
312. Phelan EA, Cheadle A, Schwartz SJ, Snyder S, Williams B, Wagner EH*, et al.* Promoting health and preventing disability in older adults: lessons from intervention studies carried out through an academic-community partnership. *Fam Community Health* 2003;**26**:214–20.
313. Lewin G, Allan J, Patterson C, Knuiman M, Boldy D, Hendrie D. A comparison of the home-care and healthcare service use and costs of older Australians randomised to receive a restorative or a conventional home-care service. *Health Soc Care Community* 2014;**22**:328–36.
314. Lewin G, Concanen K, Youens D. The home independence program with non-health professionals as care managers: An evaluation. *Clin Interv Aging* 2016;**11**:807–17.
315. Lewin G, Vandermeulen S. A non-randomised controlled trial of the Home Independence Program (HIP): an Australian restorative programme for older home-care clients. *Health Soc Care Community* 2010;**18**:91–9.
316. ACTRN12616001411437. *Evaluation of preventive home visits in elderly over 75 years*. Australian New Zealand Clinical Trials Registry; 2016. URL: https://anzctr.org.au/Trial/Registration/TrialReview.aspx?ACTRN=12616001411437 (Accessed 11 May 2020).
317. Liimatta H, Lampela P, Kautiainen H, Laitinen-Parkkonen P, Pitkala KH. The Effects of Preventive Home Visits on Older People's Use of Health Care and Social Services and Related Costs. *J Gerontol A Biol Sci Med Sci* 2019;**29**:29. https://doi.org/10.1093/gerona/glz139
318. Liimatta H, Lampela P, Laitinen-Parkkonen P, Pitkala KH. Preventive home visits to promote the health-related quality of life of home-dwelling older people: baseline findings and feasibility of a randomized, controlled trial. *Eur Geriatr Med* 2017;**8**:440-5.
319. ISRCTN22749696. *CERgAS: multiComponent Exercise and theRApeutic lifeStyle intervention to improve physical function and maintain independent living among urban poor older people*. BioMed Central: ISRCTN Registry; 2014. URL: http://www.isrctn.com/ISRCTN22749696 (Accessed 5 November 2020).
320. Rosli R, Loh D, Choo W, MohdHairi F, Peramalah D, Kandiben S*, et al.* Effects of multicomponent exercise and therapeutic lifestyle (CERgAS) intervention on cognitive function in lower income elderly population: A cluster randomised controlled trial. *Age Ageing* 2017;**46**:ii7. https://doi.org/10.1093/ageing/afx118.26

321. ACTRN12617000198325. *Efficacy and cost-effectiveness of a community based model of care for older patients with complex needs: a study protocol for a multicentre randomized controlled trial using a stepped wedge cluster design*. Australian New Zealand Clinical Trials Registry; 2017. URL: https://www.anzctr.org.au/Trial/Registration/TrialReview.aspx?id=372235 (Accessed 11 May 2020).
322. Kinchin I, Jacups S, Mann J, Quigley R, Harvey D, Doran CM*, et al.* Efficacy and cost-effectiveness of a community-based model of care for older patients with complex needs: a study protocol for a multicentre randomised controlled trial using a stepped wedge cluster design. *Trials* 2018;**19**:668.
323. Mann J, Quigley R, Harvey D, Tait M, Williams G, Strivens E. OPEN ARCH: integrated care at the primary–secondary interface for the community-dwelling older person with complex needs. *Aust J Prim Health* 2020;**26**:104–8. https://doi.org/10.1071/PY19184
324. Mann J, Thompson F, Quigley R, McDermott R, Devine S, Strivens E. Beyond multimorbidity: primary care and the older person with complex needs. *Aust J Prim Health* 2021;**27**:194–201. https://doi.org/10.1071/PY20125
325. Quigley R, Russell S, Harvey D, Mann J. OPEN ARCH integrated care model: experiences of older Australians and their carers. *Aust J Prim Health* 2021;**27**:236–42. https://doi.org/10.1071/py20203
326. Markle-Reid M. *Frail elderly home care clients: the effects and expense of adding nursing health promotion and preventative care to personal support services* [PhD thesis]. Hamilton, Ontario: McMaster University; 2002.
327. Markle-Reid M, Weir R, Browne G, Henderson S, Roberts J, Gafni A. *Frail elderly homecare clients: The costs and effects of adding nursing health promotion and preventive care to personal support services*. Ottawa, Ontario: Canadian Health Services Research Foundation; 2003. URL: https://www.researchgate.net/publication/228552727 (Accessed 21 August 2020).
328. Houles M. Cost-effectiveness analysis of a multidisciplinary intervention for vulnerable elderly individuals living at home. [French]. *Cah de l'Annee Gerontol* 2010;**2**:169–72.
329. Houles M. Randomised study of a multi-disciplinary programme focusing on geriatric syndromes in vulnerable, elderly individuals living at home (Dutch EASYcare Study). [French]. *Cah de l'Annee Gerontol* 2010;**2**:166–8.
330. Melis RJ, Adang E, Teerenstra S, van Eijken MI, Wimo A, van Achterberg T*, et al.* Cost-effectiveness of a multidisciplinary intervention model for community-dwelling frail older people. *J Gerontol A Biol Sci Med Sci* 2008;**63**:275–82.
331. Melis RJ, Teerenstra S, Rikkert MG, Borm GF. Pseudo cluster randomization performed well when used in practice. *J Clin Epidemiol* 2008;**61**:1169–75.
332. Melis RJ, van Eijken MI, Boon ME, Olde Rikkert MG, van Achterberg T. Process evaluation of a trial evaluating a multidisciplinary nurse-led home visiting programme for vulnerable older people. *Disabil Rehabil* 2010;**32**:937–46.
333. Melis RJ, van Eijken MI, Borm GF, Wensing M, Adang E, van de Lisdonk EH*, et al.* The design of the Dutch EASYcare study: a randomised controlled trial on the effectiveness of a problem-based community intervention model for frail elderly people [NCT00105378]. *BMC Health Serv Res* 2005;**5**:65.
334. NCT00105378. *Dutch EASYcare Study*. US National Library of Medicine: ClinicalTrials.gov; 2005. URL: https://clinicaltrials.gov/ct2/show/NCT00105378 (Accessed 28 April 2020).
335. Friedman B, Li Y, Liebel DV, Powers BA. Effects of a home visiting nurse intervention versus care as usual on individual activities of daily living: a secondary analysis of a randomized controlled trial. *BMC Geriatr* 2014;**14**:24.

336. Friedman B, Wamsley BR, Liebel DV, Saad ZB, Eggert GM. Patient satisfaction, empowerment, and health and disability status effects of a disease management–health promotion nurse intervention among Medicare beneficiaries with disabilities. *Gerontol* 2009;**49**:778–92.
337. Li Y, Liebel DV, Friedman B. An investigation into which individual instrumental activities of daily living are affected by a home visiting nurse intervention. *Age Ageing* 2013;**42**:27–33.
338. Liebel DV. Process evaluation of a nurse home visiting intervention that postpones disability worsening in older adults. *Diss Abst Int Pt B - Sci & Eng* 2008;**68**:5860.
339. Liebel DV, Powers BA, Friedman B, Watson NM. Barriers and facilitators to optimize function and prevent disability worsening: A content analysis of a nurse home visit intervention. *J Adv Nurs* 2012;**68**:80–93.
340. Meng H, Friedman B, Dick AW, Liebel D, Wamsley BR, Eggert GM*, et al.* Impact of a disease management-health promotion nurse intervention on personal assistance use and expenditures. *Home Health Care Serv Q* 2009;**28**:113–29.
341. Meng H, Friedman B, Dick AW, Wamsley BR, Eggert GM, Mukamel D. Effect of a voucher benefit on the demand for paid personal assistance. *Gerontologist* 2006;**46**:183–92.
342. Keijser W, Bond R, Saez I, Vandewoude M. *Health monitoring and sOcial integration environMent for Supporting WidE ExTension of independent life at HOME (Home Sweet Home): Trial Protocol Version 1.0* no. D1.2. Antwerp: Zorgbedrijf Antwerpen; 2010.
343. NCT01218373. *The Clinical Evaluation of Continuous Assistance Offered to Older People Living Independently*. US National Library of Medicine: ClinicalTrials.gov; 2010. URL: https://ClinicalTrials.gov/show/NCT01218373 (Accessed 11 May 2020).
344. Daniels R, van Rossum E, Metzelthin S, Sipers W, Habets H, Hobma S*, et al.* A disability prevention programme for community-dwelling frail older persons. *Clin Rehabil* 2011;**25**:963–74.
345. ISRCTN31954692. *The reduction of disability in community-dwelling frail elderly*. BioMed Central: ISRCTN Registry; 2009. URL: http://isrctn.com/ISRCTN31954692 (Accessed 11 May 2020).
346. Metzelthin SF, Daniëls R, van Rossum E, Cox K, Habets H, de Witte LP*, et al.* A nurse-led interdisciplinary primary care approach to prevent disability among community-dwelling frail older people: A large-scale process evaluation. *Int J Nurs Stud* 2013;**50**:1184–96. https://doi.org/10.1016/j.ijnurstu.2012.12.016
347. Metzelthin SF, Van Rossum E, De Witte LP, Ambergen A, Hobma S, Sipers W*, et al.* Frail elderly people living at home; effects of an interdisciplinary primary care programme. [Dutch]. *Ned Tijdschr Geneeskd* 2014;**158**:A7355.
348. Metzelthin SF, van Rossum E, de Witte LP, Hendriks MR, Kempen GI, Metzelthin SF*, et al.* The reduction of disability in community-dwelling frail older people: design of a two-arm cluster randomized controlled trial. *BMC Public Health* 2010;**10**:511–.
349. Metzelthin SF, van Rossum E, Hendriks MR, De Witte LP, Hobma SO, Sipers W*, et al.* Reducing disability in community-dwelling frail older people: cost-effectiveness study alongside a cluster randomised controlled trial. *Age Ageing* 2015;**44**:390–6.
350. Beishuizen CRL, Coley N, Moll van Charante EP, van Gool WA, Richard E, Andrieu S. Determinants of Dropout and Nonadherence in a Dementia Prevention Randomized Controlled Trial: The Prevention of Dementia by Intensive Vascular Care Trial. *J Am Geriatr Soc* 2017;**65**:1505–13.
351. Bussel EF, Richard E, Busschers WB, Steyerberg EW, Gool WA, Moll van Charante EP*, et al.* A cardiovascular risk prediction model for older people: Development and validation in a primary care population. *J Clin Hypertens* 2019;**21**:1145–52.

352. ISRCTN29711771. *Prevention of dementia by intensive vascular care*. BioMed Central: ISRCTN Registry; 2006. URL: http://www.isrctn.com/ISRCTN29711771 (Accessed 28 April 2020).
353. Ligthart SA, Richard E, van Gool WA, Moll van Charante EP. Cardiovascular risk management in community-dwelling elderly: opportunities for prevention. *European Journal of Preventive Cardiology* 2012;**19**:1365–72.
354. Ligthart SA, van den Eerenbeemt KD, Pols J, van Bussel EF, Richard E, Moll van Charante EP. Perspectives of older people engaging in nurse-led cardiovascular prevention programmes: a qualitative study in primary care in the Netherlands. *Br J Gen Pract* 2015;**65**:e41–8.
355. Richard E, Ligthart S, van Charante EM, Van Gool W. Methodological issues in a cluster-randomized trial to prevent dementia by intensive vascular care. *J Nutr Health Aging* 2010;**14**:315–7.
356. Richard E, Van den Heuvel E, van Charante EPM, Achthoven L, Vermeulen M, Bindels PJ*, et al.* Prevention of dementia by intensive vascular care (PreDIVA): a cluster-randomized trial in progress. *Alzheimer Dis Assoc Disord* 2009;**23**:198–204.
357. van Bussel EF, Hoevenaar-Blom MP, Busschers WB, Richard E, Peters RJG, van Gool WA*, et al.* Effects of Primary Cardiovascular Prevention on Vascular Risk in Older Adults. *Am J Prev Med* 2018;**55**:368–75.
358. van Dalen JW, Moll van Charante EP, Caan MWA, Scheltens P, Majoie C, Nederveen AJ*, et al.* Effect of Long-Term Vascular Care on Progression of Cerebrovascular Lesions: Magnetic Resonance Imaging Substudy of the PreDIVA Trial (Prevention of Dementia by Intensive Vascular Care). *Stroke* 2017;**48**:1842–8.
359. van Dalen JW, Moll van Charante EP, van Gool WA, Richard E. Discontinuation of Antihypertensive Medication, Cognitive Complaints, and Incident Dementia. *J Am Med Dir Assoc* 2019;**20**:1091–7. https://doi.org/10.1016/j.jamda.2018.12.006
360. van Dalen JW, Van Wanrooij LL, van Charante EPM, Richard E, van Gool WA, Moll van Charante EP. Apathy is associated with incident dementia in community-dwelling older people. *Neurology* 2018;**90**:e82–e9.
361. Monteserin R, Brotons C, Moral I, Altimir S, San Jose A, Santaeugenia S*, et al.* Effectiveness of a geriatric intervention in primary care: a randomized clinical trial. *Fam Pract* 2010;**27**:239–45.
362. Morey MC, Sloane R, Pieper CF, Peterson MJ, Pearson MP, Ekelund CC*, et al.* Effect of Physical Activity Guidelines on Physical Function in Older Adults. *J Am Geriatr Soc* 2008;**56**:1873–8. https://doi.org/10.1111/j.1532-5415.2008.01937.x
363. Peterson MJ, Sloane R, Cohen HJ, Crowley GM, Pieper CF, Morey MC*, et al.* Effect of telephone exercise counseling on frailty in older veterans: Project LIFE. *Am J Mens Health* 2007;**1**:326–34.
364. Hall KS, Sloane R, Pieper CF, Peterson MJ, Crowley GM, Cowper PA*, et al.* Long-term changes in physical activity following a one-year home-based physical activity counseling program in older adults with multiple morbidities. *J Aging Res* 2011;**2011**:308407. https://doi.org/10.4061/2011/308407
365. Huffman KM, Sloane R, Peterson MJ, Bosworth HB, Ekelund C, Pearson M*, et al.* The impact of self-reported arthritis and diabetes on response to a home-based physical activity counselling intervention. *Scand J Rheumatol* 2010;**39**:233-9.
366. Morey MC, Peterson MJ, Pieper CF, Sloane R, Crowley GM, Cowper P*, et al.* Project LIFE--Learning to Improve Fitness and Function in Elders: methods, design, and baseline characteristics of randomized trial. *J Rehabil Res Dev* 2008;**45**:31–42.
367. NCT00435188. *Life 2: Improving Fitness and Function in Elders (Project LIFE)*. US National Library of Medicine: ClinicalTrials.gov; 2007. URL: https://clinicaltrials.gov/ct2/show/NCT00435188 (Accessed 20 June 2020).

368. Haase AM, Taylor AH, Fox KR, Thorp H, Lewis G. Rationale and development of the physical activity counselling intervention for a pragmatic TRial of Exercise and Depression in the UK (TREAD-UK). *Ment Health Phys Act* 2010;**3**:85–91. https://doi.org/10.1016/j.mhpa.2010.09.004
369. ISRCTN80470273. *The PACE Study: physical activity facilitation for older adults*. BioMed Central: ISRCTN Registry; 2013. URL: http://www.isrctn.com/ISRCTN80470273 (Accessed 20 June 2020).
370. Morgan GS, Haase AM, Campbell R, Ben-Shlomo Y. Physical ACtivity facilitation for Elders (PACE): study protocol for a randomised controlled trial. *Trials* 2015;**16**:91.
371. Newbury JW. *75+ Health assessments: a randomised controlled trial* [MD thesis]. Adelaide: Adelaide University; 2001.
372. Graves MT, Slater MA, Maravilla V, Reissler L, Faculjak P, Newcomer RJ. Implementing an early intervention case management program in three medical groups. *Case Manag* 2003;**14**:48–52. https://doi.org/10.1016/S1061-9259(03)00212-1
373. Maravilla V, Graves MT, Newcomer R. Development of a standardized language for case management among high-risk elderly. *Lippincotts Case Manag* 2005;**10**:3–13. https://doi.org/10.1097/00129234-200501000-00002
374. NCT00973258. *Randomized Controlled Trial of Community-based Nutritional, Physical and Cognitive Training Intervention Programmes for At Risk Frail Elderly (FIT)*. US National Library of Medicine: ClinicalTrials.gov; 2009. URL: https://clinicaltrials.gov/ct2/show/NCT00973258 (Accessed 28 April 2020).
375. Ng TP, Ling LHA, Feng L, Nyunt MSZ, Feng L, Niti M*, et al.* Cognitive Effects of Multi-Domain Interventions Among Pre-Frail and Frail Community-Living Older Persons: Randomized Controlled Trial. *J Gerontol A Biol Sci Med Sci* 2018;**73**:806–12.
376. ACTRN12608000027314. *Examination of the effect of a designated client centred goal facilitation tool on service provision, quality of life and independence among older people*. Australian New Zealand Clinical Trials Registry; 2008. URL: https://www.anzctr.org.au/Trial/Registration/TrialReview.aspx?id=82511&isReview=true (Accessed 8 June 2020).
377. Parsons JG, Sheridan N, Rouse P, Robinson E, Connolly M. A randomized controlled trial to determine the effect of a model of restorative home care on physical function and social support among older people. *Arch Phys Med Rehabil* 2013;**94**:1015–22.
378. Parsons M, Senior HE, Kerse N, Chen MH, Jacobs S, Vanderhoorn S*, et al.* The Assessment of Services Promoting Independence and Recovery in Elders Trial (ASPIRE): a pre-planned meta-analysis of three independent randomised controlled trial evaluations of ageing in place initiatives in New Zealand. *Age Ageing* 2012;**41**:722–8.
379. Parsons M, Anderson C, Senior H, Chen X, Kerse N, Jorgensen D*, et al. ASPIRE: Assessment of Services Promoting Independence and Recovery in Elders*: New Zealand Ministry of Health; 2006. URL: https://www.health.govt.nz/system/files/documents/pages/aspire-research-report.pdf (Accessed 29 May 2020).
380. Auckland UniServices Limited. *An economic evaluation of the Assessment of Service Promoting Independence and Recovery in Elders (ASPIRE) - final report*: New Zealand Ministry of Health; 2006. URL: https://www.health.govt.nz/system/files/documents/pages/aspire-economic-evaluation-report.pdf (Accessed 29 May 2020).
381. ACTRN12605000140651. *Assessment of Services Promoting Independence and Recovery in Elders*. Australian New Zealand Clinical Trials Registry; 2005. URL: http://www.anzctr.org.au/ACTRN12605000140651.aspx (Accessed 11 May 2020).

382. NCT00134836. *Preventive Primary Care Outreach for High Risk Older Persons*. US National Library of Medicine: ClinicalTrials.gov; 2004. URL: https://ClinicalTrials.gov/show/NCT00134836 (Accessed 11 May 2020).
383. Dapp U, Anders J, von Renteln-Kruse W, Golgert S, Meier-Baumgartner HP, Minder CE. The longitudinal urban cohort ageing study (LUCAS): study protocol and participation in the first decade. *BMC Geriatr* 2012;**12**:35. https://doi.org/10.1186/1471-2318-12-35
384. Dapp U, Anders JA, von Renteln-Kruse W, Minder CE, Meier-Baumgartner HP, Swift CG*, et al.* A randomized trial of effects of health risk appraisal combined with group sessions or home visits on preventive behaviors in older adults. *J Gerontol A Biol Sci Med Sci* 2011;**66**:591–8. https://doi.org/10.1093/gerona/glr021
385. von Renteln-Kruse W, Anders J, Dapp U, Meier-Baumgartner HP. Präventive Hausbesuche durch einespeziell fortgebildete Pflegefachkraft bei 60-jährigen undälteren Personen in Hamburg. *Z Gerontol Geriatr* 2003;**36**:378–91. https://doi.org/10.1007/s00391-003-0179-9
386. Rockwood K, Howlett S, Stadnyk K, Carver D, Powell C, Stolee P. Responsiveness of goal attainment scaling in a randomized controlled trial of comprehensive geriatric assessment. *J Clin Epidemiol* 2003;**56**:736–43.
387. NCT01969526. *Effectiveness of a Multifactorial Intervention on Frailty*. US National Library of Medicine: ClinicalTrials.gov; 2013. URL: https://clinicaltrials.gov/ct2/show/NCT01969526 (Accessed 27 August 2021).
388. Romera L, Orfila F, Segura JM, Ramirez A, Moller M, Fabra ML*, et al.* Effectiveness of a primary care based multifactorial intervention to improve frailty parameters in the elderly: a randomised clinical trial: rationale and study design. *BMC Geriatr* 2014;**14**:125.
389. Metzelthin SF, Rooijackers TH, Zijlstra GAR, van Rossum E, Veenstra MY, Koster A*, et al.* Effects, costs and feasibility of the 'Stay Active at Home' Reablement training programme for home care professionals: study protocol of a cluster randomised controlled trial. *BMC Geriatr* 2018;**18**:276.
390. Metzelthin SF, Zijlstra GA, van Rossum E, de Man-van Ginkel JM, Resnick B, Lewin G*, et al.* 'Doing with ...' rather than 'doing for ...' older adults: Rationale and content of the 'Stay Active at Home' programme. *Clin Rehabil* 2017;**31**:1419–30.
391. NCT03293303. *The Feasibility, Effects and Costs of the 'Stay Active at Home Programme'*. US National Library of Medicine: ClinicalTrials.gov; 2017. URL: https://clinicaltrials.gov/ct2/show/NCT03293303 (Accessed 28 April 2020).
392. Rooijackers TH, Zijlstra GAR, van Rossum E, Vogel RGM, Veenstra MY, Kempen GIJM*, et al.* Process evaluation of a reablement training program for homecare staff to encourage independence in community-dwelling older adults. *BMC Geriatr* 2021;**21**:5. https://doi.org/10.1186/s12877-020-01936-7
393. Smeets RGM, Kempen GIJM, Zijlstra GAR, van Rossum E, de Man-van Ginkel JM, Hanssen WAG*, et al.* Experiences of home-care workers with the 'Stay Active at Home' programme targeting reablement of community-living older adults: An exploratory study. *Health Soc Care Community* 2020;**28**:291-9. https://doi.org/10.1111/hsc.12863
394. Ryvicker M, Marren J, Sobolewski S, Acampora T, Flannery M, Buff E*, et al.* Spreading improvement strategies within a large home healthcare organization. *J Healthc Qual* 2008;**30**:48–58.
395. Serra-Prat M. *Clinical and economic assessment of a pre-frail screening program. NCT02138968*. ClinicalTrials.gov: US National Institutes of Health; 2014. URL: https://ClinicalTrials.gov/show/NCT02138968 (Accessed 7 February, 2020).
396. Sherman H. *Preventive home visits for 75 years old persons by the district nurse* [thesis]. Stockholm: Karolinska Institutet; 2012.

397. Fleuren MAH, Vrijkotte S, Jans MP, Pin R, van Hespen A, van Meeteren NLU*, et al.* The implementation of the functional task exercise programme for elderly people living at home. *BMC Musculoskelet Disord* 2012;**13**:1–9.
398. NTR2407. *Training of the ability to live independently 75+.* Netherlands National Trial Register; 2010. URL: https://www.trialregister.nl/trial/2280 (Accessed 8 May 2020).
399. Stewart S, Lloyd-Smith W, Poland F. Running a community clinical trial: lessons from the CAMELOT project. *Br J Ther Rehabil* 2003;**10**:443–8. https://doi.org/10.12968/bjtr.2003.10.10.13475
400. Flood C, Mugford M, Stewart S, Harvey I, Poland F, Lloyd-Smith W. Occupational therapy compared with social work assessment for older people. An economic evaluation alongside the CAMELOT randomised controlled trial. *Age Ageing* 2005;**34**:47–52.
401. Alessi CA, Stuck AE, Aronow HU, Yuhas KE, Bula CJ, Madison R*, et al.* The process of care in preventive in-home comprehensive geriatric assessment. *J Am Geriatr Soc* 1997;**45**:1044–50.
402. Büla CJ, Alessi CA, Aronow HU, Yubas K, Gold M, Nisenbaum R*, et al.* Community physicians' cooperation with a program of in-home comprehensive geriatric assessment. *J Am Geriatr Soc* 1995;**43**:1016-20.
403. Büla CJ, Bérod AC, Stuck AE, Alessi CA, Aronow HU, Santos-Eggimann B*, et al.* Effectiveness of preventive in-home geriatric assessment in well functioning, community-dwelling older people: secondary analysis of a randomized trial. *J Am Geriatr Soc* 1999;**47**:389-95.
404. Cho CY, Alessi CA, Cho M, Aronow HU, Stuck AE, Rubenstein LZ*, et al.* The association between chronic illness and functional change among participants in a comprehensive geriatric assessment program. *J Am Geriatr Soc* 1998;**46**:677–82.
405. Rubenstein LZ, Aronow HU, Schloe M, Steiner A, Alessi CA, Yuhas KE*, et al.* A home-based geriatric assessment, follow-up and health promotion program: design, methods, and baseline findings from a 3-year randomized clinical trial. *Aging Clin Exp Res* 1994;**6**:105–20.
406. Stuck AE, Gafner Zwahlen H, Neuenschwander BE, Meyer Schweizer RA, Bauen G, Beck JC. Erratum for: Methodologic challenges of randomized controlled studies on in-home comprehensive geriatric assessment: The EIGER project. Aging Clin Exp Res 1995;7:218-23. *Aging Clin Exp Res* 1995;**7**:237.
407. Stuck AE, Gafner Zwahlen H, Neuenschwander BE, Meyer Schweizer RA, Bauen G, Beck JC. Erratum for: Methodologic challenges of randomized controlled studies on in-home comprehensive geriatric assessment: The EIGER project. Aging Clin Exp Res 1995;7:218-23. *Aging Clin Exp Res* 1998;**10**:348.
408. Stuck AE, Stuckelberger A, Gafner Zwahlen H, Minder CE, Beck JC. A randomised trial of in-home preventive visits with annual comprehensive geriatric assessment: Base-line findings of the EIGER project. [French]. *Med Hyg (Geneve)* 1995;**53**:2385–97.
409. Stuck AE, Gafner Zwahlen H, Neuenschwander BE, Meyer Schweizer RA, Bauen G, Beck JC. Methodologic challenges of randomized controlled studies on in-home comprehensive geriatric assessment: The EIGER project. *Aging Clin Exp Res* 1995;**7**:218–23.
410. ISRCTN28458424. *Disability prevention in the older population: use of information technology for health risk appraisal and prevention of functional decline*. BioMed Central: ISRCTN Registry; 2005. URL: http://www.isrctn.com/ISRCTN28458424 (Accessed 20 May 2020).
411. NL2535. *FIT-study: Maintaining Functionality in Transition*. Netherlands National Trial Register; 2010. URL: https://www.trialregister.nl/trial/2535 (Accessed 28 April 2020).
412. Suijker J, MacNeil-Vroomen J, Van Rijn M, Buurman B, Ter Riet G, De Rooij S*, et al.* Economic evaluation of nurse-led multifactorial care to prevent or postpone new disabilities in community-living older people: results of a cluster randomized trial. *Eur Geriatr Med* 2016;**7**:S103-.

413. Suijker JJ, Buurman BM, ter Riet G, van Rijn M, de Haan RJ, de Rooij SE*, et al.* Comprehensive geriatric assessment, multifactorial interventions and nurse-led care coordination to prevent functional decline in community-dwelling older persons: protocol of a cluster randomized trial. *BMC Health Serv Res* 2012;**12**:85.
414. Suijker JJ, MacNeil-Vroomen JL, van Rijn M, Buurman BM, de Rooij SE, Moll van Charante EP*, et al.* Cost-effectiveness of nurse-led multifactorial care to prevent or postpone new disabilities in community-living older people: Results of a cluster randomized trial. *PLoS One* 2017;**12**:e0175272.
415. Suijker JJ, van Rijn M, Ter Riet G, Moll van Charante EP, de Rooij SE, Buurman BM. Minimal Important Change and Minimal Detectable Change in Activities of Daily Living in Community-Living Older People. *J Nutr Health Aging* 2017;**21**:165–72.
416. Van Rijn M, Hoogteijling N, Suijker J, De Rooij S, Buurman B, Van Charante EM. Preventive home-visits and nurse-led care coordination: a qualitative study on the experiences, needs and preferences of community dwelling older people. *Eur Geriatr Med* 2016;**7**:S103-S4.
417. Szanton SL, Leff B, Li Q, Breysse J, Spoelstra S, Kell J*, et al.* CAPABLE program improves disability in multiple randomized trials. *J Am Geriatr Soc* 2021;**69**:3631-40. https://doi.org/10.1111/jgs.17383
418. Szanton SL, Tanner EK, Thorpe RJ, Agree E, Seplaki C, Boyd C*, et al.* A multi-component pilot to enhance aging-in-place capacity for low-income older adults. *Clin Transl Sci* 2010;**3**:S20.
419. LaFave SE, Granbom M, Cudjoe TKM, Gottsch A, Shorb G, Szanton SL. Attention control group activities and perceived benefit in a trial of a behavioral intervention for older adults. *Res Nurs Health* 2019;**42**:476–82.
420. NCT01576133. *Reducing Disability Via a Bundled Bio-Behavioral-Environmental Approach (CAPABLE)*. US National Library of Medicine: ClinicalTrials.gov; 2012. URL: https://clinicaltrials.gov/ct2/show/study/NCT01576133 (Accessed 28 April 2020).
421. NCT01743495. *CAPABLE for Frail Dually Eligible Older Adults (CAPABLE500)*. US National Library of Medicine: ClinicalTrials.gov; 2012. URL: https://clinicaltrials.gov/ct2/show/NCT01743495 (Accessed 28 April 2020).
422. Nkimbeng M, Roberts L, Thorpe Jr RJ, Gitlin LN, Delaney A, Tanner EK*, et al.* Recruiting older adults with functional difficulties into a community-based research study: Approaches and costs. *J Appl Gerontol* 2020;**39**:644–50. https://doi.org/10.1177/0733464818786612
423. Patadia P, Roberts L, Szanton S. Are difficulties with daily activities related to neighborhood? A geospatial analysis of CAPABLE baseline data. *J Am Geriatr Soc* 2018;**66**:S289.
424. Smith PD, Becker K, Roberts L, Walker J, Szanton SL. Associations among pain, depression, and functional limitation in low-income, home-dwelling older adults: An analysis of baseline data from CAPABLE. *Geriatr Nurs* 2016;**37**:348–52.
425. Szanton SL, Alfonso YN, Leff B, Guralnik J, Wolff JL, Stockwell I*, et al.* Medicaid cost savings of a preventive home visit program for disabled older adults. *J Am Geriatr Soc* 2018;**66**:614–20.
426. Szanton SL, Wolff JL, Leff B, Roberts L, Thorpe RJ, Tanner EK*, et al.* Preliminary data from Community Aging in Place, Advancing Better Living for Elders, a patient-directed, team-based intervention to improve physical function and decrease nursing home utilization: the first 100 individuals to complete a Centers for Medicare and Medicaid Services innovation project. *J Am Geriatr Soc* 2015;**63**:371–4. https://doi.org/10.1111/jgs.13245
427. Szanton SL, Wolff JW, Leff B, Thorpe RJ, Tanner EK, Boyd C*, et al.* CAPABLE trial: a randomized controlled trial of nurse, occupational therapist and handyman to reduce disability among older adults: rationale and design. *Contemp Clin Trials* 2014;**38**:102–12.
428. Waldersen BW, Wolff JL, Roberts L, Bridges AE, Gitlin LN, Szanton SL. Functional goals and predictors of their attainment in low-income community-dwelling older adults. *Arch Phys Med Rehabil* 2017;**98**:896–903.

429. NCT01056640. *Telemonitoring Versus Usual Care*. US National Library of Medicine: ClinicalTrials.gov; 2010. URL: https://clinicaltrials.gov/ct2/show/study/NCT01056640 (Accessed 18 May 2021).
430. Pecina JL, Hanson GJ, Van Houten H, Takahashi PY. Impact of telemonitoring on older adults health-related quality of life: the Tele-ERA study. *Qual Life Res* 2013;**22**:2315–21. https://doi.org/10.1007/s11136-013-0361-5
431. Pecina JL, Vickers KS, Finnie DM, Hathaway JC, Hanson GJ, Takahashi PY. Telemonitoring Increases Patient Awareness of Health and Prompts Health-Related Action: Initial Evaluation of the TELE-ERA Study. *Telemed E Health* 2011;**17**:461–6. https://doi.org/10.1089/tmj.2010.0213
432. Takahashi PY, Hanson GJ, Pecina JL, Stroebel RJ, Chaudhry R, Shah ND*, et al.* A randomized controlled trial of telemonitoring in older adults with multiple chronic conditions: the Tele-ERA study. *BMC Health Serv Res* 2010;**10**:255.
433. Takahashi PY, Hanson GJ, Thorsteinsdottir B, Van Houten HK, Shah ND, Naessens JM*, et al.* The impact of telemonitoring upon hospice referral in the community: a randomized controlled trial. *Clin Interv Aging* 2012;**7**:445–51. https://doi.org/10.2147/CIA.S36461
434. Upatising B, Hanson GJ, Kim YL, Cha SS, Yih Y, Takahashi PY. Effects of home telemonitoring on transitions between frailty states and death for older adults: a randomized controlled trial. *Int J Gen Med* 2013;**6**:145–51. https://doi.org/10.2147/IJGM.S40576
435. Upatising B, Wood DL, Kremers WK, Christ SL, Yih Y, Hanson GJ*, et al.* Cost comparison between home telemonitoring and usual care of older adults: a randomized trial (Tele-ERA). *Telemed E Health* 2015;**21**:3–8.
436. NCT00974506. *Pilot Study: Complementary Therapies in Geriatric Patients*. US National Library of Medicine: ClinicalTrials.gov; 2009. URL: https://clinicaltrials.gov/ct2/show/NCT00974506 (Accessed 21 August 2020).
437. DRKS00011831. *A multimodal intervention program for older people with frailty – a randomized controlled pilot trial*. World Health Organization: International Clinical Trials Registry Platform; 2017. URL: https://trialsearch.who.int/Trial2.aspx?TrialID=DRKS00011831 (Accessed 11 May 2020).
438. Ziller C, Braun T, Thiel C. Frailty phenotype prevalence in community-dwelling older adults according to physical activity assessment method. *Clin Interv Aging* 2020;**15**:343–55. https://doi.org/10.2147/CIA.S238204
439. Kjerstad E, Tuntland HK. Reablement in community-dwelling older adults: a cost-effectiveness analysis alongside a randomized controlled trial. *Health Econ Rev* 2016;**6**:15.
440. NCT02043262. *The Effectiveness of Reablement in Home Dwelling Older Adults. A Randomized Controlled Trial*. US National Library of Medicine: ClinicalTrials.gov; 2012. URL: https://clinicaltrials.gov/ct2/show/NCT02043262 (Accessed 28 April 2020).
441. Tuntland H, Espehaug B, Forland O, Hole AD, Kjerstad E, Kjeken I. Reablement in community-dwelling adults: study protocol for a randomised controlled trial. *BMC Geriatr* 2014;**14**:139.
442. NTR5184. *PROTO-study: Prevention of undernutrition in community dwelling older adults*. Netherlands National Trial Register; 2015. URL: https://www.trialregister.nl/trial/5045 (Accessed 21 August 2020).
443. Dorhout BG, Haveman-Nies A, van Dongen EJI, Wezenbeek NLW, Doets EL, Bulten A*, et al.* Cost-effectiveness of a Diet and Resistance Exercise Intervention in Community-Dwelling Older Adults: ProMuscle in Practice. *J Am Med Dir Assoc* 2021;**22**:792–. https://doi.org/10.1016/j.jamda.2020.12.036
444. NTR6038. *ProMuscle in Practice: Effectiveness of a combined resistance exercise and nutrition intervention to promote maintenance of physical functioning of community-dwelling elderly in a real-life setting*. Netherlands National Trial Register; 2016. URL: https://www.trialregister.nl/trial/5858 (Accessed 16 May 2020).

445. van Dongen EJI, Doets EL, de Groot LCPGM, Dorhout BG, Haveman-Nies A. Process Evaluation of a Combined Lifestyle Intervention for Community-Dwelling Older Adults: ProMuscle in Practice. *Gerontol* 2020;**60**:1538–54. https://doi.org/10.1093/geront/gnaa027
446. van Dongen EJI, Haveman-Nies A, Wezenbeek NLW, Dorhout BG, Doets EL, de Groot L. Effect, process, and economic evaluation of a combined resistance exercise and diet intervention (ProMuscle in Practice) for community-dwelling older adults: design and methods of a randomised controlled trial. *BMC Public Health* 2018;**18**:877.
447. University of Groningen. *Appendix in the final report of the project: "The effects of physical and cognitive interventions on disability in older persons"* no. 014-90-027. The Netherlands: University of Groningen; 2005.
448. Van Hout H. *The cost-effectiveness of systematic home visits by nurses of frail elderly primary care patients and caregivers of demented patients*. BioMed Central: ISRCTN Registry; 2005. URL: http://www.isrctn.com/ISRCTN05358495 (Accessed.
449. van Hout HP, Nijpels G, van Marwijk HW, Jansen AP, Van't Veer PJ, Tybout W*, et al.* Design and pilot results of a single blind randomized controlled trial of systematic demand-led home visits by nurses to frail elderly persons in primary care [ISRCTN05358495]. *BMC Geriatr* 2005;**5**:11.
450. Hoogendijk EO, van der Horst HE, van de Ven PM, Twisk JW, Deeg DJ, Frijters DH*, et al.* Effectiveness of a Geriatric Care Model for frail older adults in primary care: Results from a stepped wedge cluster randomized trial. *Eur J Intern Med* 2016;**28**:43–51.
451. Muntinga ME, Hoogendijk EO, van Leeuwen KM, van Hout HP, Twisk JW, van der Horst HE*, et al.* Implementing the chronic care model for frail older adults in the Netherlands: study protocol of ACT (frail older adults: care in transition). *BMC Geriatr* 2012;**12**:19.
452. Muntinga ME, Mokkink LB, Knol DL, Nijpels G, Jansen APD. Measurement properties of the Client-centered Care Questionnaire (CCCQ): factor structure, reliability and validity of a questionnaire to assess self-reported client-centeredness of home care services in a population of frail, older people. *Qual Life Res* 2014;**23**:2063–72.
453. Muntinga ME, Van Leeuwen KM, Schellevis FG, Nijpels G, Jansen AP. From concept to content: assessing the implementation fidelity of a chronic care model for frail, older people who live at home. *BMC Health Serv Res* 2015;**15**:18.
454. NL2043. *The frail elderly person at the centre of cohesive care*. Netherlands National Trial Register; 2010. URL: https://trialregister.nl/trial/2043 (Accessed 11 May 2020).
455. NTR3980. *KWIEK. Investigating the effectiveness on self-management and quality of life of an integrated multidimensional lifestyle program in older adults.* Netherlands National Trial Register; 2013. URL: https://www.trialregister.nl/trial/3814 (Accessed 22 May 2020).
456. van Rossum E, Frederiks C, Philipsen H, Kil-van Lierop J, Mantel A, Portengen J*, et al.* Design of a Dutch study to test preventive home visits to the elderly. *Nurs Res* 1991;**40**:185–8.
457. van Rossum HJL. *Effects of preventive home visits to the elderly* [thesis]. Maastricht: Rijksuniversiteit Limburg; 1993.
458. Avlund K, Vass M, Hendriksen C. Education of preventive home visitors: The effects on change in tiredness in daily activities. *Eur J Ageing* 2007;**4**:125–31.
459. Avlund K, Vass M, Kvist K, Hendriksen C, Keiding N. Educational intervention toward preventive home visitors reduced functional decline in community-living older women. *J Clin Epidemiol* 2007;**60**:954–62.
460. Avlund K, Vass M, Lund R, Yamada Y, Hendriksen C. Influence of psychological characteristics and social relations on receiving preventive home visits in older men and women. *Eur J Ageing* 2008;**5**:191–201. https://doi.org/10.1007/s10433-008-0086-4
461. Ekmann A, Vass M, Avlund K. Preventive home visits to older home-dwelling people in Denmark: are invitational procedures of importance? *Health Soc Care Community* 2010;**18**:563–71. https://doi.org/10.1111/j.1365-2524.2010.00941.x

462. Elkjaer E, Poulsen T, Avlund K. Stability and change in physical activity in old age: the role of changes in disability. *Eur J Ageing* 2006;**3**:89–97. https://doi.org/10.1007/s10433-006-0025-1
463. Hendriksen C, Vass M. Preventive home visits to elderly people in Denmark. *Z Gerontol Geriatr* 2005;**38**:I/31–I/3.
464. Jørgensen TSH, Lund R, Siersma VD, Nilsson CJ. Interplay between financial assets and social relations on decline in physical function and mortality among older people. *Eur J Ageing* 2018;**15**:133–42. https://doi.org/10.1007/s10433-017-0437-0
465. Kronborg C, Vass M, Lauridsen J, Avlund K. Cost effectiveness of preventive home visits to the elderly: economic evaluation alongside randomized controlled study. *Eur J Health Econ* 2006;**7**:238–46.
466. Lund R, Nilsson CJ, Avlund K. Can the higher risk of disability onset among older people who live alone be alleviated by strong social relations? A longitudinal study of non-disabled men and women. *Age Ageing* 2010;**39**:319–26. https://doi.org/10.1093/ageing/afq020
467. Nilsson CJ, Avlund K, Lund R. Social Inequality in Onset of Mobility Disability Among Older Danes: The Mediation Effect of Social Relations. *J Aging Health* 2010;**22**:522–41. https://doi.org/10.1177/0898264309359684
468. Nilsson CJ, Lund R, Avlund K. Cohabitation Status and Onset of Disability Among Older Danes: Is Social Participation a Possible Mediator? *J Aging Health* 2007;**20**:235–53. https://doi.org/10.1177/0898264307310474
469. Nilsson CJ, Siersma V, Mänty M, Avlund K, Vass M, Lund R. Mobility decline in old age: the combined effect of mobility-related fatigue and socioeconomic position. *J Epidemiol Community Health* 2014;**68**:510. https://doi.org/10.1136/jech-2013-203060
470. Poulsen T, Elkjaer E, Vass M, Hendriksen C, Avlund K. Promoting physical activity in older adults by education of home visitors. *Eur J Ageing* 2007;**4**:115–24. https://doi.org/10.1007/s10433-007-0057-1
471. Poulsen T, Siersma VD, Lund R, Christensen U, Vass M, Avlund K. Educational intervention and functional decline among older people: The modifying effects of social capital. *Scand J Public Health* 2014;**42**:295–303. https://doi.org/10.1177/1403494813520353
472. Vass M, Avlund K, Hendriksen C. Randomized intervention trial on preventive home visits to older people: baseline and follow-up characteristics of participants and non-participants. *Scand J Public Health* 2007;**35**:410–7.
473. Vass M, Avlund K, Hendriksen C, Andersen CK, Keiding N. Preventive home visits to older people in Denmark: methodology of a randomized controlled study. *Aging Clin Exp Res* 2002;**14**:509–15.
474. Vass M, Avlund K, Kvist K, Hendriksen C, Andersen CK, Keiding N. Structured home visits to older people. Are they only of benefit for women? A randomised controlled trial. *Scand J Prim Health Care* 2004;**22**:106–11.
475. Vass M, Avlund K, Parner ET, Hendriksen C. Preventive home visits to older home-dwelling people and different functional decline patterns. *Eur J Ageing* 2007;**4**:107–13. https://doi.org/10.1007/s10433-007-0059-z
476. Vass M, Avlund K, Siersma V, Hendriksen C. A feasible model for prevention of functional decline in older home-dwelling people - The GP role. A municipality-randomized intervention trial. *Fam Pract* 2009;**26**:56–64.
477. Vass M, Hendriksen C, Thomsen JL, Parner ET, Avlund K. Preventive home visits to home-dwelling older people and hospital admissions: a municipality-randomised intervention trial. *Eur J Ageing* 2008;**5**:67–76.
478. Vass M, Holmberg R, Fiil-Nielsen H, Lauridsen J, Avlund K, Hendriksen C. Preventive home visitation programmes for older people: The role of municipality organisation. *Eur J Ageing* 2007;**4**:133–40.

479. ISRCTN07330512. *Screening and Counseling for physical Activity and Mobility in Older people*. BioMed Central: ISRCTN Registry; 2005. URL: https://doi.org/10.1186/ISRCTN07330512 (Accessed 21 August 2020).
480. Leinonen R, Heikkinen E, Hirvensalo M, Lintunen T, Rasinaho M, Sakari-Rantala R*, et al.* Customer-oriented counseling for physical activity in older people: study protocol and selected baseline results of a randomized-controlled trial (ISRCTN 07330512). *Scand J Med Sci Sports* 2007;**17**:156–64. https://doi.org/10.1111/j.1600-0838.2006.00536.x
481. Mänty M, Heinonen A, Leinonen R, Törmäkangas T, Hirvensalo M, Kallinen M*, et al.* Long-term Effect of Physical Activity Counseling on Mobility Limitation Among Older People: A Randomized Controlled Study. *J Gerontol A Biol Sci Med Sci* 2009;**64A**:83–9. https://doi.org/10.1093/gerona/gln029
482. Rasinaho M, Hirvensalo M, Törmäkangas T, Leinonen R, Lintunen T, Rantanen T. Effect of physical activity counseling on physical activity of older people in Finland (ISRCTN 07330512). *Health Promot Int* 2011;**27**:463–74. https://doi.org/10.1093/heapro/dar057
483. Sallinen J, Mänty M, Leinonen R, Kallinen M, Törmäkangas T, Heikkinen E*, et al.* Factors associated with maximal walking speed among older community-living adults. *Aging Clin Exp Res* 2011;**23**:273–8. https://doi.org/10.1007/BF03337753
484. von Bonsdorff MB, Leinonen R, Kujala UM, Heikkinen E, Tormakangas T, Hirvensalo M*, et al.* Effect of physical activity counseling on home care use in older people: Letters to the editor. *J Am Geriatr Soc* 2009;**57**:571–3.
485. Avgerinou C, Gardner B, Kharicha K, Frost R, Liljas A, Elaswarapu R*, et al.* Health promotion for mild frailty based on behaviour change: Perceptions of older people and service providers. *Health Soc Care Community* 2019;**27**:1333–43. https://doi.org/10.1111/hsc.12781
486. ISRCTN11986672. *The development and feasibility of a new service to promote health and well-being in older people who are starting to become frailer: the HomeHealth study*. World Health Organization: International Clinical Trials Registry Platform; 2015. URL: https://trialsearch.who.int/Trial2.aspx?TrialID=ISRCTN11986672 (Accessed 11 May 2020).
487. ISRCTN21710246. *Occupational therapy in homecare re-ablement services*. BioMed Central: ISRCTN Registry; 2014. URL: http://www.isrctn.com/ISRCTN21710246 (Accessed 28 April 2020).
488. Whitehead PJ, Drummond AE, Walker MF, Parry RH, McGeorge ID, Latif Z*, et al.* Occupational Therapy in HomEcare Re-ablement Services (OTHERS): study protocol for a randomized controlled trial. *Trials* 2014;**15**:447–.
489. Williams EI, Greenwell J, Groom LM. Characteristics of patients aged 75 years and over who are discharged from hospital without district nursing support. *J Public Health Med* 1992;**14**:321–7. https://doi.org/10.1093/oxfordjournals.pubmed.a042749
490. Roth G, Wolter A, Stolle C, Rothgang H. The long and bumpy road to outcome-oriented management of long-term care in Germany: implementation of the Resident Assessment Instrument in home-care services. *Int J Health Plann Manag* 2014;**29**:316–29. https://doi.org/10.1002/hpm.2186
491. Stolle C, Wolter A, Roth G, Rothgang H. Effects of the Resident Assessment Instrument in home care settings: results of a cluster randomized controlled trial. *Z Gerontol Geriatr* 2012;**45**:315–22.
492. Stolle C, Wolter A, Roth G, Rothgang H. Improving health status and reduction of institutionalization in long-term care-Effects of the Resident Assessment Instrument- Home Care by degree of implementation. *Int J Nurs Pract* 2015;**21**:612–21.
493. NCT02286375. *Effects of Health-social Partnership Programme*. US National Library of Medicine: ClinicalTrials.gov; 2014. URL: https://clinicaltrials.gov/show/NCT02286375 (Accessed 11 May 2020).

494. Wong AKC, Wong FKY. The psychological impact of a nurse-led proactive self-care program on independent, non-frail community-dwelling older adults: A randomized controlled trial. *Int J Nurs Stud* 2020;**110**:103724. https://doi.org/10.1016/j.ijnurstu.2020.103724
495. Wong AKC, Wong FKY, So C. Cost-effectiveness of a preventive self-care health management program for community-dwelling older adults: a randomised controlled trial. *Age Ageing* 2020;**07**:07. https://doi.org/10.1093/ageing/afaa127
496. Wong KC, Wong FK, Chang KK. Health-social partnership intervention programme for community-dwelling older adults: a research protocol for a randomized controlled trial. *J Adv Nurs* 2015;**71**:2673–85.

Appendix 5. Example intervention description for each of the TIDieR items

The example given is for Whitehead 2016 [1] Home care reablement plus Occupational Therapy (Homecare, ADL, aids and multifactorial-action)

1. Brief name	Home care reablement plus Occupational Therapy. A targeted ADL programme, delivered by an occupational therapist incorporating goal setting, teaching/practising techniques, equipment/adaptations and provision of advice/support. This was in addition to home care reablement.
2. Why	The aims:<br><br>- to maximize independence in ADL activities, including: washing, dressing, bathing and showering, feeding, indoor mobility, transfers, stair mobility, toileting, meal preparation and kitchen activities, outdoor mobility and community access.<br><br>- to deliver cost-saving for social care services.<br><br>Rationale:<br><br>- The Care Act 2014 has placed a statutory duty on local authorities in England to provide services that prevent or delay the need for other health and social care services, which may involve maximising independent living. Reablement is identified within The Care Act statutory guidance as an example of prevention and has been identified as one of the 'top-ten' prevention services for older adults.<br><br>- Occupational therapists are argued to have a particularly important role to play in delivering successful reablement outcomes as services aim to support individuals to manage daily living tasks independently; this is a core aspect of occupational therapy practice. Furthermore, occupational therapists are the only allied health profession to be employed within social care services in significant numbers and thus are already working as social care professionals.
3. What (materials)	1. Provision of community equipment and/or minor adaptations (such as grab rails, half-steps or threshold removal or replacements)
4. What (procedures)	1. Received standard home care provided by paid reablement care workers homecare reablement services.<br><br>2. Enhanced programme targeted at ADLs, delivered by an occupational therapist. Tailored programme to participants needs.<br><br>3. Goal setting using the TARGET (practising activities, and/or a graded process of re-learning and building the skills to manage ADL independently).

	4. Equipment provision and environmental or activity modification. (Provision of community equipment and/or minor adaptations)<br><br>5. case management approach involving a minimum of weekly reviews and the coordination of the reablement episode and other services; and advice and information will also be provided to family members or carers.<br><br>6. If required, length of homecare and OT services can be extended after 6 weeks (available usual care).
5. Who provided	Number of intervention providers : 4+<br><br>1. Reablement workers (social / paid care workers)<br><br>2. Reablement care team leader (social care team manager)<br><br>3. Occupational therapist<br><br>4. Possible but unclear: Community Equipment Service staff for fitting equipment.<br><br>5. After 6-week reablement service, if continuous homecare is required: home care agency/ paid home care staff.
6. How	The intervention was provided in the home. Face to face home visits.
6b. How organised	1. A case management approach will be adopted by the occupational therapist involving a minimum of weekly reviews and the coordination of the reablement episode and other services.<br><br>2. The OTs are part of the social care services.<br><br>3. Home care reablement is provided by reablement social/paid care workers, under the direction of a reablement care team leader.<br><br>4. Services required after the enhanced home care reablement programme is provided by care agency.
7. Where	United Kingdom<br><br>The setting was a local authority homecare reablement service in England.<br><br>The study will be conducted within one city council homecare re-ablement service in England.<br><br>The service is divided into six geographical sub-teams. This RCT will be conducted within one subteam, which currently does not have routine input from an occupational therapist.
8. When and how much	Started following referral for home care reablement.<br><br>Enhanced programme and home care reablement provided concurrently up to six weeks.

	Home care can be extended and/or OT referrals for necessary period afterwards.
9. Tailoring	1. A program will then be agreed with the participant, which will be tailored to the needs of each individual, but will include: practicing activities, and/or a graded process of re-learning and building the skills to manage ADL independently; equipment provision and environmental or activity modification.<br><br>2. Length of home care reablement services, referrals to homecare are tailored.
10. Modifications	An unanticipated issue which affected the recruitment rate was the introduction of new occupational therapists into the reablement service during the course of the study. Midway through the trial recruitment period, additional occupational therapists were employed to work within the service.<br><br>However, the new occupational therapists had insufficient capacity to work with every service user and were allocated to particular geographical subteams within the authority. Therefore, the study continued within two geographical subteams where the additional occupational therapists were not employed (this was later reduced to one).
11. How well (planned)	1. Cost analysis<br><br>2. As part of the cost evaluation, a record will be kept of the number of times the occupational therapist visited each service user in the intervention group, the amount of time spent per visit and a log of what was carried out on each visit (in the form of a coded checklist). In addition, a record will be kept of the cost of equipment and minor adaptation services provided. Participants in both groups will report their use of health and social care services during the intervention and follow-up period [2].
12. How well (actual)	An unanticipated issue which affected the recruitment rate was the introduction of new occupational therapists into the reablement service during the course of the study. Midway through the trial recruitment period, additional occupational therapists were employed to work within the service. However, the new occupational therapists had insufficient capacity to work with every service user and were allocated to particular geographical subteams within the authority. Therefore, the study continued within two geographical subteams where the additional occupational therapists were not employed (this was later reduced to one).

References (appendix 5)

1. Whitehead PJ, Walker MF, Parry RH, Latif Z, McGeorge ID, Drummond AE; Occupational Therapy in HomEcare Re-ablement Services (OTHERS): results of a feasibility randomised controlled trial. *BMJ Open* 2016;**6**(8):e011868. doi: 10.1136/bmjopen-2016-011868.
2. Whitehead PJ, Drummond AE, Walker MF, *et al.*; Occupational Therapy in HomEcare Re-ablement Services (OTHERS): study protocol for a randomized controlled trial. *Trials* 2014;**15**(1):447–447.

Appendix 6: Plain language descriptions of the components and aspects of components used to determine intervention groups, organised by topic

Topic	Brief name (abbreviation)	Public-facing name	Plain language description
Activities	ADL (ADL)	Practise **day-to-day activities**	The person is offered support to practise carrying out day-to-day activities, for example dressing or taking the bus. The person may also be offered recommendations on how to carry out day-to-day activities safely or better. For example, this may include using appropriate footwear, removing loose rugs, cords, and clutter in walking paths or improvement of lighting. The person may receive an assessment to create a tailored day-to-day activities plan.
Activities	Aids (aids)	Get **equipment and technology** to support day-to-day activities	The person is offered equipment or technology to aid in day-to-day activities. This may include ramps, walking frames, grab rails, or a system of sensors that turn on the lights when the person gets up from the bed, for example. The person may receive an assessment to choose specific equipment or technology.
Activities	Meaningful activities (eng)	Identify and engage in **meaningful activities**	The person is offered support to identify and participate in activities that they find meaningful. Examples may include leisure activities, crafts, volunteering, but the focus is on the activities being ones that the person finds meaningful. The activities may be organized for the person, be done by the person alone, or be community activities that were already in place, for example.
Brain training	Cognitive training (cgn)	Do **brain training**	The person is offered training in thinking tasks such as memorising, paying attention or planning, among others. The training includes practical exercises and information about strategies to help thinking tasks.

Topic	Brief name (abbreviation)	Public-facing name	Plain language description
Diet/nutrition	Nutrition (ntr)	Get **dietary advice and support**	The person is offered recommendations about diet and/or food supplements in group sessions or one-to-one. This is different from receiving information about nutrition as part of "Find out more information about health" because there is a greater focus on providing specialized nutrition/dietary advice and related activities. For example, the person may also participate in writing a food diary, cooking certain types of meals, and weight monitoring. They may be provided with particular foods or supplements. The person may receive an assessment to create a tailored nutrition plan.
Financial support	Care voucher (vchr)	Get a health and care **voucher**	The person is offered a voucher to pay for health and personal care services and support on how to use the voucher.
Financial support	Welfare (wlfr)	Get advice about **welfare services** with follow-up	The person is offered tailored advice about the welfare services and benefits they can access. This is based on an assessment. Afterwards, the person is offered support in putting the plan in practice and accessing the services and benefits they are entitled to.
General health information	Education (educ)	Find out more **information about health**	The person is offered information about a set of health topics. The topics may include many areas, for example, oral health, nutrition, physical activity. The information may also focus on areas that are more important for the person. The way the information is provided is more structured than the particular advice someone may receive as part of a clinical consultation with a health professional. The person may be offered information in group sessions or on one-to-one contact.
Homecare	Homecare (hmcr)	Receive formal **home care**	The person is offered support services at home by health or care professionals. The services include, for example, nursing care or support with household tasks.

Topic	Brief name (abbreviation)	Public-facing name	Plain language description
Individualised care	Medication review (med)	**Optimise my medication**	The person is offered recommendations to change medication. For example, someone may be on too many medicines and be recommended to stop some. The changes to the medication can be provided on their own or as part of a more complete assessment and recommendations (see "Take part in individualised care planning based on an assessment" for more details).
Individualised care	Monitoring (mntr)	Get care planning from health **monitoring** (including providing equipment)	If a health need is identified from monitoring, the person is offered an individualised care plan (see "Take part in individualised care planning" for more details). To check for needs, the person participates in screening and monitoring of their bodily function, for example blood pressure, and heart rate. This happens at least weekly. The person is offered equipment to record their bodily function.
Individualised care	Multifactorial action (mfa)	Take part in **individualised care planning**	The person is offered an individualised care plan that includes recommendations for future action. The care plan is based on an assessment of the person's needs and preferences and may include a variety of actions (related with physical exercise, diet, mood, etc.). The assessment structure may be set in advance or guided by the experience of a clinician. The person may receive support to carry out actions, for example, with referrals to certain services. The person may also receive support from a care coordinator, who helps to deal with different services and/or professionals.
Individualised care	Review [in relation to multifactorial action] (mfar)	Have **regular follow ups** [after individualised care planning]	The person is regularly followed up after receiving an individualised care plan based on an assessment. The follow up may include encouraging the person to carry out previous recommendations. The person may also be offered a new assessment of their needs and other relevant changes, and an updated individualised care plan.

Topic	Brief name (abbreviation)	Public-facing name	Plain language description
Individualised care	Risk screening (rsk)	Get care planning following **screening** for possible health problems	A tool to indicate possible health problems is used routinely and, if indicated, the person is offered an individualised care plan (see “Take part in individualised care planning” for more details). The tool and the results that indicate problems are standardised, such as a questionnaire score or analysis of electronic health records.
Individualised care	Self-management [in multidomain assessment and care planning] (slfm)	Do activities to motivate taking good care of myself [when taking part in individualised care planning]	The person is engaged in conversations or activities designed to motivate them to care for themself. The person may also be offered guided practice in some techniques, for example, to help them set up personal goals and solve problems.
Alternative medicine	Alternative medicine (hmnt)	Get **alternative medicine**	The person is offered alternative medicine such as homeopathic or naturopathic consultation and treatment.
Physical exercise	Exercise (exrc)	Do physical **exercise**	The person is offered support to carry out physical exercise. The exercise may be on their own or in training sessions. This is different from receiving information about physical activity as part of “Find out more information about health” because there is a greater focus on providing specialised physical exercise advice and related activities. Physical exercises are activities done by a person to build up or maintain physical fitness (such as strength, balance, among others). The person may also receive an assessment to create a tailored exercise plan.
Social communication	Social skills (sst)	Practise **social interaction**	The person is offered information and support to improve their ability to relate with other people. This may include practising or discussing different ways of communicating.
Social communication	Telecoms (comm)	Get **technology for communication and engagement**	The person is offered technology to enable communication with friends, family, neighbours, or the community. For example, a mobile phone, or tablet, as well as applications such as email or social media. The person will usually receive support in using the applications.

Topic	Brief name (abbreviation)	Public-facing name	Plain language description
Wellbeing	Psychology (psyc)	Get **wellbeing advice and support**	The person is offered support for their wellbeing in areas like feeling low and dealing with worries. The support includes information about how we usually think and feel, and information and activities to deal with what we think and feel, such as noticing and learning how to overcome unhelpful thoughts.

Appendix 7: Intervention group summaries where group contains more than one intervention

Group: ADL

There are two interventions in this group: Dorresteijn 2016 [1], and Siemonsma 2018 [2].

TIDieR item	Description
1. Brief name	
2. Why	Goal: Both interventions had a focus on encouraging and enhancing independent living for older people. One was focused upon those who had a fear of falls which reduced and restricted their activity levels. This intervention also aimed to reduce burden on the healthcare services. The other intervention was focused upon increasing physical activity to prevent decline in a sustainable way.<br><br>Rationale: One intervention was based upon previous programme effectiveness. Both interventions had grounding in cognitive theories related to self-efficacy and control. The sustainability of the intervention was rationalised as likely due to embedding exercises within routine activity in one intervention. The other saw provision at home as beneficial to sustainability.
3. What (materials)	One intervention was vague in describing intervention material referring only to training materials for the providers of the intervention. The other intervention listed DVD's with case studies of challenges and solutions, printed materials including educational leaflets, checklists and worksheets, action planning documentation, standardised assessments and an evaluation questionnaire for participants.
4. What (procedures)	The descriptions of the processes for carrying out the interventions were varied. Both interventions mentioned an aspect of cognitive restructuring, motivational interviewing or confidence building. Training was provided in both interventions in a targeted and supervised way. One mentioned how this training could be monitored and adapted over time and was to focus on daily tasks. One was focused on reducing fear of falling. One intervention mentioned the input of caregivers. One also mentioned accessibility to usual care by a multidisciplinary team.
5. Who provided	Both interventions were delivered by healthcare professionals with specialised intervention training. One was delivered by community/geriatric nurses. The other by physiotherapists. Usual care was provided by relevant professionals.
6. How	Provision was face-to-face and to individuals or with a significant other present. One intervention included input over the telephone; the other had home based contact only.
6b. How organised	One intervention did not mention organisation, the other places organisation on the facilitator and the participants' significant other to undertake activities.
7. Where	Both interventions were implemented in The Netherlands, and in the participants' home.

8. When and how much	Eligibility for the interventions varied. One intervention was accessible on referral. The other was available to people over 70 living in their own homes, identified by a postal screening questionnaire as having a fear of falling and fair to poor self-perceived health with a level of frailty.<br><br>The nature, duration and frequency of delivery varied. One intervention comprised seven sessions, three of which were face-to-face around 60-75 minutes in duration and four of which were over the telephone about 35 minutes in duration. The other intervention was delivered over a maximum of 18 sessions. One intervention duration was ten weeks, the other three months.
9. Tailoring	Both interventions were tailored to the needs, abilities and preferences of the individuals. One intervention aimed to provide tailored training on a feared activity of the participants' choice. The other intervention was tailored to the participants' home environment and was monitored and adapted throughout the programme.
10. Modifications	Neither study described modifications to the intervention.
11. How well (planned)	One study did not mention any plans for adherence or fidelity assessment. The other study conducted an evaluation of acceptability and feasibility by the participant. They also aimed to collect information on adherence to the intervention protocol, the time spent on delivery of the intervention and identify any barriers to implementation.
12. How well (actual)	One study did not undertake adherence or fidelity assessment. The other found that the intervention was perceived as feasible and acceptable by deliverers and participants. The intervention protocol was broadly adhered to. Action planning decreased over the duration of the intervention from over 70% to just above 50%. It was noted that training on a feared activity was problematic as this feared activity was often hard to identify.

Group: ADL, aids, education, exercise, multifactorial-action and review with medication review and self-management

There are two interventions in this group: Szanton 2011 [3], and Szanton 2019 [4].

TIDieR item	Description
1. Brief name	
2. Why	Goal: Both interventions were targeted at both intrinsic (personal) and extrinsic (environmental) factors which contribute to disability in older people. In addition both interventions were targeted at those who were living on a low income. Both interventions had a focus on function, either by improvement in function or reducing functional difficulties. One intervention also mentioned reducing disability and the use of person centred goal setting to improve overall health, wellbeing and quality of life.<br><br>Rationale: Both studies mentioned the value of person centred approaches to care provision. One intervention refers to the need to address multiple factors which contribute to the decline of older people with a multi-component

	intervention, with consideration of the idea that such factors often interact to increase the impact on disability. This intervention also noted the need to reduce healthcare costs. The other intervention attributed the higher level of disability in lower income adults to a range of factors including environmental ones. This intervention was theoretically grounded and based on the success of piloting work.
3. What (materials)	Both interventions used a similar material base. A client/clinical assessment protocol, home modifications and assistive devices, letters and/or referrals from nurses to primary care providers. Additionally a DVD of Tai Chi exercises was provided in both interventions. One intervention also mentioned a health passport. The other intervention included the provision of a medication calendar and a Community Aging in Place - Advancing Better Living for Elders (CAPABLE) notebook to participants. In addition to the above one intervention described training materials for the providers, including a manual, audio tapes to record the sessions, a checklist to review the sessions and reminders regarding upcoming sessions.
4. What (procedures)	Both interventions included a multi-domain assessment with subsequent planning and arrangement of care based upon this. This assessment focused upon a range of domains including function, depression, pain, strength, medication and environmental factors among others. Physical exercise training and health related education provision on various topics including medication management, falls risk and self-management strategies were part of both interventions. The provision, fitting and relevant training on the use of adaptations to the environment was part of both interventions. Both interventions included access to relevant additional support such as Tai Chi training and mental health support for depression. Both interventions included routine reviewing and refinement of planning as well as access to usual care. One intervention also described the training and supervision of providers.
5. Who provided	Both interventions were delivered by nurses and occupational therapists. The adaptations were made by handymen. One intervention described input from a primary care provider and the other from relevant professionals of a multidisciplinary team as required.
6. How	The intervention was provided at home to individuals face-to-face. One intervention mentioned the collaborative development of care planning between providers and the inclusion of motivational interviewing to participants.
6b. How organised	In both interventions the care plans were designed to be delivered by a multidisciplinary team; occupational therapists and home modification co-ordinators organised and facilitated the housing adaptations. Both interventions involved planning documentation and appropriate letters and referrals to be sent by nurses. One intervention mentioned the staggering of intervention delivery to give participants time to engage with components. The other intervention used a secure share-site for the ease of sharing documentation across providers.
7. Where	Both interventions were undertaken in the USA, and were delivered at home.
8. When and how much	Both interventions targeted those who were from low income circumstances, with at least one limitation to activities of daily living and two limitations to instrumental activities of daily living. One intervention was location specific

	and participants were recruited from a waiting list for home based services. The participants were contacted by post.<br><br>The nature, duration and frequency of intervention delivery was similar across interventions, both involving around six visits from occupational therapists, four visits from nurses, each of around 60 to 90 minutes in duration. One intervention lasted six months, the other four months. Both interventions described home visits to provide adaptations over as many visits as required.
9. Tailoring	Both interventions were tailored to the participant's goals, preferences, and risk level. This included the number and nature of visits as well as the development of strategies. One intervention described the tailoring of the training, the adaptations at home and the behavioural plan. The other intervention included a medical alert if polypharmacy was a significant concern.
10. Modifications	No modifications were mentioned in either study.
11. How well (planned)	One study did not mention the intention to measure fidelity or evaluate the intervention. The other included staff training, reminders for participants, supervised learning of exercises and the supervision of providers to improve adherence.
12. How well (actual)	One study did not mention fidelity or evaluate the intervention effectiveness. The other intervention noted that 92.8% of participants received at least eight sessions, less than 4% received less than three sessions, which had been defined as a minimum threshold for treatment. There was a mean of 9.1 visits per participant.

Group: Aids

There are two interventions in this group: Borrows 2013 [5], and Tomita 2007 [6].

TIDieR item	Description
1. Brief name	
2. Why	Goal: One intervention had one clear goal, to reduce disability; whilst the other goal and rationale was focused upon decreasing dependence to sustain living at home, and to enable informed decision making by older people on equipment and products to maintain living at home.<br><br>Rationale: One study did not distinguish between the goal and rationale; however the implication was that independent living centres provide an opportunity to support informed decision making and safe use of aids and adaptations to maintain living at home. The other intervention was based on previous studies showing the benefit of assistive technology in sustaining living at home, additionally the technology of choice was based upon evidence due to ease of installation and use.
3. What (materials)	A range of materials were provided to participants in these interventions. One intervention was focused upon assistive technology, providing X10 Active Home kits including the necessary software, other standalone products, activity monitoring software and a computer and internet access as required. The other intervention was orientated to physical supportive equipment such as toileting and bathing equipment, medical equipment was also available on

	loan. Additionally this intervention provided information and advice on the safe use of equipment to maintain independence at home.
**4. What (procedures)**	Both interventions involved an assessment of needs; one specifies this as an assessment of both the individual and their home setting. One intervention included the installation of equipment, training on the safe use of this and ongoing support. The other intervention used the assessment to identify appropriate equipment which the participant was required to fit, although they could try out demonstration equipment at the independent living centre, which they received transportation to. Advice on other supportive service options was also identified during this assessment.
**5. Who provided**	One intervention was provided by an Occupational Therapist or nurse, with a geriatric nurse providing support. Equipment was fitted by a computer engineer. This study explicitly mentioned intervention specific training. The other intervention was provided by an Occupational Therapist assistant.
**6. How**	Both interventions were provided individually and face-to-face, however one was at home and provided additional support by telephone. The other intervention was provided in the independent living centre.
**6b. How organised**	Organisation was not always entirely clear in one intervention but stated that there was a cost limit of $400. The other intervention was organised by the British Red Cross.
**7. Where**	One intervention was undertaken in the USA and in the participant's home and the other in the independent living centre(s) in the UK.
**8. When and how much**	One intervention had clear eligibility criteria, participants had to be 60 years of age, living alone, have impairments to activities of daily living or instrumental activities of daily living, to be interested in technology and have no cognitive impairments. The other intervention simply mentioned access to be two weeks after randomisation.<br><br>The assessments for the intervention varied in length, one involved a 90 minute assessment, the other a 150 minute assessment. The technology intervention allowed for three to nine hours for engineer to install the equipment at the participant's home, with this intervention support was given as required.
**9. Tailoring**	Both studies described the interventions being tailored to the needs, preferences, and also the safe capacity of the participant. One intervention mentioned follow up support being as required, the other mentioned training on the equipment being tailored.
**10. Modifications**	Neither study mentioned modification to the intervention.
**11. How well (planned)**	One study did not mention work to assess fidelity or adherence. The other intervention mentioned that fidelity to the intervention was promoted. Additionally this study collected data on the type of technology which was provided, as well as problems encountered and solutions to those problems.
**12. How well (actual)**	One study did not report on fidelity or adherence. The other reported that 100% of participants received software, although there was variation in what support items they accessed. Two years later 65% of participants were still using one or multiple pieces of assistive technology. Lack of use of the equipment was usually related to a failure of the equipment, either meaning

	functional failure, the equipment not meeting the needs of the participants or the participant's inability to use it.

Group: Available care

There are 98 interventions in this group: Alegria 2019 [7], Arthanat 2019 [8], Balaban 1988 [9], Barenfeld 2018 [10], Bleijenberg 2016 [11], Blom 2016 [12], Botjes 2013 [13], Bouman 2008 [14], Brettschneider 2015 [15], Cameron 2013 [16], Carpenter 1990 [17], Cesari 2014 [18], Clark 1997 (2 interventions) [19], Clark 2012 [20], Coleman 1999 [21], Counsell 2007 [22], Cutchin 2009 [23], Dalby 2000 [24], de Craen 2006 [25], Dorresteijn 2016 [1], Fabacher 1994 [26], Fairhall 2015 [27], Fischer 2009 [28], Ford 1971 [29], Gene Huguet 2018 [30], Gill 2002 [31], Giné-Garriga 2020 [32], Gitlin 2006 [33], Grimmer 2013 [34], Gustafson 2021 [35], Gustafsson 2013 [36], Harari 2008 [37], Hay 1998 (2 interventions) [38], Hebert 2001 [39], Henderson 2005 [40], Hendriksen 1984 [41], Hogg 2009 [42], Holland 2005 [43], Howel 2019 [44], Imhof 2012 [45], Jitapunkul 1998 [46], Kerse 2014 [47], Kono 2004 [48], Kukkonen-Harjula 2017 [49], Lambotte 2018 [50], Leung 2004 [51], Leveille 1998 [52], Liddle 1996 [53], Liimatta 2019 [54], Loh 2015 [55], Lood 2015 [56], Mann J 2021 [57], Melis 2008 [58], Meng 2005 [59], Messens 2014 [60], Metzelthin 2013 [61], Moll van Charante 2016 [62], Monteserin Nadal 2008 [63], Morey 2009 [64], Morgan 2019 [65], Newbury 2001 [66], Newcomer 2004 [67], Ng 2015 [68], Pathy 1992 [69], Phelan 2007 [70], Ploeg 2010 [71], Profener 2016 [72], Rockwood 2000 [73], Romera-Liebana 2018 [74], Rubenstein 2007 [75], Serra-Prat 2017 [76], Shapiro 2002 [77], Sherman 2016 [78], Stuck 1995 [79], Stuck 2000 [80], Stuck 2015 [81], Suijker 2016 [82], Szanton 2011 [3], Szanton 2019 [4], Takahashi 2012 [83], Thiel 2019 [84], Thomas 2007 [85], Tomita 2007 [6], Tulloch 1979 [86], van Dongen 2020 [87], van Heuvelen 2005 [88], van Hout 2010 [89], van Leeuwen 2015 [90], van Lieshout 2018 [91], van Rossum 1993 [92], Vetter 1984 [93], von Bonsdorff 2008 [94], Wallace 1998 [95], Walters 2017 [96], Wong 2019 [97], and Yamada 2003 [98].

TIDieR item	Description
1. Brief name	
2. Why	Around 22 studies included some rationalisation and goals in their description of the intervention. Four referred to an ageing or frail population living with unmet needs or in some stage of functional decline. Three others described current care, including the need to limit costs, provide quality care and to compare standard primary care with the specialist care of geriatricians. Two studies indicated a need to promote independence in the older population. Five studies mentioned standardised care, whilst eight described access to actions that were not intended or anticipated to affect an individual's independence such as attention control, placebo, and assessments or social interaction.
3. What (materials)	Twenty-seven studies made some mention of materials required. Ten studies used various assessments, some of which were standardised. Eight described written materials provided to participants, a further study provided intervention materials to control participants at the end of the research process and another provided participants with placebo nutritional supplements. Four studies mentioned access to usual care equipment and services. At least ten studies described the sharing of information gleaned during assessment with other healthcare professionals through referrals etc. for ethical purposes. Materials for provider training

	and the assessment of fidelity were also mentioned in a small number of studies.
4. What (procedures)	A large majority of studies had some description of the procedure for the intervention. In 82 cases this included reference to usual available care, which a participant would access of their own accord. Ten studies described the assessment of participants, six mentioned social contact with the research team and referred to this as increased attention. Seven studies explained that identification of emergency needs required this information to be shared with other professionals as an ethical or moral obligation. Some studies provided non-active components to participants, in one case this was a placebo nutritional supplement, in five others this was written materials and eight interventions included peer contact, such as workshops or educational lectures.
5. Who provided	Almost half, around 43 studies, did not mention the providers. However, thirty-nine did refer to the provision of usual care by the expected professionals, while 15 mentioned the participants own GP or physician. It was not always clear if this was related to an aspect of the intervention which would be beyond usual care or not. Five studies explicitly referred to input from the research team. Additional providers mentioned were nurses, social workers, occupational therapists, non-trained or non-medical personnel, health educators and students, these last were usually when an intervention involved some non-active components such as placebo social interaction.
6. How	Sixty-four studies did not describe how 'available care' was delivered. At least thirteen of the remaining number referred to usual care being provided in the most appropriate way, for example in clinics and at home, through distanced or face-to-face methods. Some studies are less clear though a small percentage had face-to-face and individual contact for assessments, three used the postal service to provide information or collect assessments from participants, five conducted telephone calls and three had workshop or group sessions as part of a placebo, non-active component of the control.
6b. How organised	The majority, over 60, studies did not describe organisation. However around one third made some reference to organisation for funding. This was usually the nationally recognised approach to care funding in which the study was practising, be that state funded care or through varied insurance plans. GPs and primary care physicians were mentioned as involved in organisation in at least 14 studies. This was often in a gatekeeper role, recommending care and referring on to other services. Two studies mentioned explicit input in organisation of study including a nurse and a research assistant.
7. Where	All studies gave some indication of the location in which the studies were undertaken, though the country of one of these was unclear. Ninety-four studies were carried out in one country alone, whilst two were multi-site studies in four different countries. One study was carried out in Denmark, Northern Ireland, Germany and Spain: the other in Belgium, Spain, Ireland and Italy. Of the remaining studies the majority were also European, including 16 in The Netherlands, eight in the UK, four each in Germany, Spain and Finland. Three were carried out in Sweden, three in Switzerland and one each in France, Denmark and Belgium. A significant amount were also Northern American, including 26 in the USA and eight in Canada.

	Seven were caried out in Australia and one in New Zealand. Seven were undertaken in Asian countries, two in Japan, two in Hong Kong, one each in Thailand, Malaysia and Singapore.
8. When and how much	Most of the studies had set inclusion and exclusion criteria, although ten were not described at all. Fifty-nine studies involved those with identified specific needs, be that level of frailty, a diagnosis of a specific chronic condition or limitations to activities of daily living. Another common inclusion criterion was a minimum age limit. Fifty-three studies used age limits, usually just a minimum age, the lowest being 50 years and over, the highest being 85 years and over. Other common inclusion criteria were involvement in a service, which was mentioned in 26 studies, specific socio-economic factors, mentioned in at least nine studies, recent hospital attendance and involvement in a research cohort. Multiple studies excluded participants based on cognition and end of life status.<br><br>Few studies mentioned frequency of input given the nature of available care, however five did mention the contact of those administering assessments and ten described to some extent the nature of non-active components such as social telephone calls or workshop sessions.
9. Tailoring	Only ten of the 98 studies detailed any tailoring. Five mentioned that tailoring would be enacted by the participant themself in line with their own care needs. Three studies explicitly described processes to access emergency services should the need be identified through the research process. Four studies had non-active or control components which involved tailoring, such as a social activity tailored to the participants preferences.
10. Modifications	Only one study mentioned modifications which related to reformation of service provision during the project.
11. How well (planned)	Very few studies detailed any steps to ensure fidelity to the intervention. Three of these were related to the recording and supervision of contacts participants had with providers to ensure delivery was as per protocol. Two studies also mentioned training of providers to ensure experimental intervention components were not administered to control participants. At least two studies described steps taken to limit control participant access to the components of the experimental intervention, a further study detailed that any cross-contamination was measured. Two studies detailed steps to ensure participant compliance with attention control or placebo.
12. How well (actual)	Very few studies detailed success of delivery. Two studies noted that a substantial proportion of participants allocated to available care accessed components of the experimental intervention privately, while two others noted that participants accessed at least some aspects of the experimental intervention by some means. One reported on high levels of attrition, one explained that some participants had been referred to care due to initial assessments revealing emergency need. Three studies with placebo or attention-control components found compliance with these to be reasonably good. One study noted that the intervention was delivered as intended.

Group: Education

There are three interventions in this group: Barenfeld 2018 [10], Gustafsson 2013 [36], and Lood 2015 [56].

TIDieR item	Description
1. Brief name	
2. Why	Goal: All three interventions had very similar goals, focused around the prevention or delay of deterioration in health and quality of life of older people. Two were focused upon the prevention of frailty and morbidity, one of these was also to support ageing in place. Two interventions were aiming to reduce the consumption of care. Two interventions were also targeting minority groups with language barriers.<br>Rationale: Person-centred care approaches were the core rationale for two of the interventions, as was the premise that peer learning would prove beneficial. All three interventions were based upon previous research, including RCT's of group education. In one intervention it was put forth that a multidisciplinary team was well placed to provide health education and benefit the health outcomes of older people.
3. What (materials)	All three interventions provided health advice information in a written format. One also provided the information in audio format. Two interventions provided information in different languages as well as the native language. One intervention also mentioned documentation materials and referrals as required. One other intervention described how usual care needs were to be met with regards to equipment provision.
4. What (procedures)	All three interventions worked with a group session format, where health and social care professionals delivered a specific session. Sessions provided education and a forum for peer discussion which was relevant to participants and required their input and exchange of experiences. All interventions provided group sessions then a follow up of one individual session at home. All interventions enabled access to usual care, including home care and medical services. One intervention also described how providers were supported, and one other described the input of interpreters.
5. Who provided	All three interventions were provided by a multidisciplinary team including an Occupational Therapist, a Nurse, a Physiotherapist and a Social Worker. Usual care was provided by a range of staff as required. One intervention also required the input of supportive staff such as translators.
6. How	All three interventions were delivered face-to-face in group sessions of four to six participants and then with one session delivered individually.
6b. How organised	Organisation for the intervention was described in varying detail. All three described the input of the four key professionals and the participant. One intervention described the training of providers and some of the auditing processes, this intervention and one other also mentioned funding from the state and the other described input from the university. One intervention stated the importance of provider continuity.
7. Where	All three interventions were undertaken in Sweden, individual sessions were delivered at the home of the participant, however the location of the group session delivery was unclear in the reports.

8. When and how much	All three interventions required participants to not have existing support to carry out activities of daily living. Two interventions had a minimum age requirement of 70 years and to be a migrant to Sweden. The other intervention required participants to be classified as prefrail. It appeared that the interventions were location specific.<br><br>All three interventions were delivered through four weekly sessions, lasting between one and half and two hours. The follow up individual sessions were delivered about two to three weeks after the final group session.
9. Tailoring	All three interventions described tailoring to the needs of the participant. In the group sessions this involved pertinent discussion for the group and follow up was tailored to the individual's needs. The two interventions aimed at supporting migrants could tailor language as required.
10. Modifications	None of the reports described modifications to the interventions.
11. How well (planned)	All three studies described steps taken to improve adherence and monitor fidelity. All three developed their intervention with input from stakeholders including representatives of the participant group. One intervention also described training for providers and a priori approval of deviations from protocol. One other intervention implicated the use of consistent providers for continuity. The other intervention improved attendance by goal setting at registration and predefining minimum participation levels of 50% of meetings to be attended by participants.
12. How well (actual)	Intervention attendance was monitored and reported for all interventions. One intervention had 73% attendance at 3 or more sessions, one other had 99% attendance at 3 or more sessions while the other had 100% attendance at 3 or more sessions.

Group: Education, exercise, multifactorial-action and review with medication review and self-management strategies

There are two interventions in this group: Faul 2009 [99], and Leveille 1998 [52].

TIDieR item	Description
1. Brief name	
2. Why	Goal: To reduce the risk of frailty, disability and dependence by enhancing existing care models with the promotion of self-management strategies.<br><br>Rationale: Both interventions were theoretically driven and evidence based. Drawing upon previous work implicating the benefits of such programmes with older people of reducing risk of decline by empowering and informing older people.
3. What (materials)	Both interventions developed care planning based on an assessment of the participant and advice sheets were provided to the participant. One intervention provided a self-management workbook and referrals to services. The other used standardised assessments, accessed existing care notes and also provided an exercise software programme. This intervention also used scripting for their telephone contact.

**4. What (procedures)**	Both interventions required a comprehensive assessment, although the content of the data collected was only described for one, which focused on function, mobility, mental health, medication and the home environment. A tailored care plan was developed in both interventions, according to the needs and preferences of the participant. This included a tailored exercise plan. Both interventions provided information on health behaviour. One intervention also explicitly mentioned referrals on to mental health and substance misuse services. Both interventions described follow up input and telephone contact. One intervention used peer support mentoring, for which training was undertaken.
**5. Who provided**	One intervention was provided by an interdisciplinary team, led by a physical therapist and working with a physical therapist student and a social work student. The other was overseen by a geriatric nurse practitioner and required the input from health mentors, lay leaders, primary care physicians, dietitians and social workers as required.
**6. How**	Both interventions had individual provision, one also used group sessions. Provision was face-to-face, both interventions were conducted within the home and used telephone contact, one also appeared to have been in senior centres.
**6b. How organised**	Organisation varied, one intervention was overseen by the interdisciplinary team, students were supervised, aims were to forge community links. Reports were shared with participant's primary care physicians. The other intervention depended upon a number of large health providers for the development and implementation of the intervention as well as access to participants.
**7. Where**	Both interventions were provided at home, one was also provided in a senior centre. Both interventions were run in the USA.
**8. When and how much**	Inclusion and exclusion criteria varied between interventions. One had a minimum age of 65, requirement for participants to have a permanent address, to be literate and have a primary care physician. The other intervention required referral based on one or more chronic condition. One study excluded those living in long-term care, those with acute needs or recent serious health events, or those in receipt of home care. The other study excluded those living with dementia or with terminal conditions.<br><br>The interventions varied in number of visits: one conducted three, while the other conducted between one and eight. The duration of visits was mentioned in one study as 1-2 hours. One study had a requirement of eight phone calls, the other had between one and 22 calls. The duration and frequency of group sessions was noted for one study.
**9. Tailoring**	Both interventions required the tailoring of care and exercise routine planning based on needs and preference of the participant. One study had tailored referrals
**10. Modifications**	This was not mentioned in the studies.
**11. How well (planned)**	One study described the use of training and supervision to intervention providers to ensure fidelity. The other intervention promoted home exercise sessions to improve compliance with this aspect of the intervention.
**12. How well (actual)**	This was not reported in detail for either study. One study mentioned that generic issues rather than self-management strategies were more commonly discussed in contact sessions. The other intervention found that participants

	were reasonably willing to attend sessions but participation at exercise classes was lower than anticipated.

Group: Education, multifactorial-action and review with medication review

There are three interventions in this group: Newcomer 2004 [67], Ploeg 2010 [71], and Stuck 1995 [79].

TIDieR item	Description
1. Brief name	
2. Why	Goal: All three interventions had a goal of reducing health resource use and thus lowering health care costs. Two interventions aimed to provide timely and comprehensive care and improve patient health. One of these and the remaining intervention aimed to prevent decline by reducing risk factors, increasing quality life years and improving health and wellbeing.<br><br>Rationale: Two interventions were based on previous research findings. Proactive and preventative approaches and appropriate use of health services and beneficial relationships with health care professionals were mentioned by these two interventions also. One of these interventions believed improved access and awareness of preventative health planning would be advantageous, whilst the other suggested that home-based care provision would be of benefit. The third intervention simply stated that the intervention would improve quality of life and reduce mortality compared to usual care.
3. What (materials)	All three interventions utilised referrals based upon need. Two used standardised screening measures. All three used types of recording and documentation, in care planning including one which described electronic records. Two interventions described the provision of information, one of these was about local community resources. One intervention provided aids and equipment as and when needed.
4. What (procedures)	All three interventions involved a multidomain assessment, referrals from this, as well as some form of care planning process following the assessment. Additionally, all three interventions provided educational materials in some form. Two studies described the monitoring process. Three studies described interaction to promote empowerment of participants, including coaching and encouragement from the providers. One intervention included communication from the participant to the primary care provider, one mentioned involvement of the family physician and one described the review process.
5. Who provided	All three interventions were primarily provided by Nurses, though each was described differently, one as a Nurse Case Manager, one as a Home Care Nurse and one as a Geriatric Nurse. Two studies mentioned the input of the Family Physician or Primary Care Physician, other input on these two interventions came from health care professionals as needed. Research Assistant input was required for one intervention.
6. How	Two interventions involved the initial assessment being undertaken face-to-face at the home of the participant. Follow up contact could be by

	telephone. In the other intervention initial screening was undertaken by post, with the option for telephone or face-to-face assessments if required.
**6b. How organised**	All three interventions described the bulk of organisation by the nurse provider and some input organisationally by various health care professionals as needed following referrals. Medication reviews were part of all interventions and two mentioned specialist input. One intervention required input from project geriatricians, another mentioned the participants taking an active role. Two mentioned input from the family doctor.
**7. Where**	Two interventions were undertaken in the USA and one in Canada.<br><br>Two interventions were undertaken at home, one predominantly involved self-assessment. Follow up care was provided in a variety of locations one was specifically at home, while one other described community care settings.
**8. When and how much**	Only one study described detailed inclusion criteria, while one invited participants from the voter registry. The one which described inclusion criteria included those who were enrolled on a specific health insurance programme for a minimum of a year, had a high risk of adverse health outcomes and were aged 80 and over or 65 and over with one chronic health condition.<br><br>The nature, duration and frequency of contact was very variable across the interventions. One involved the potential for daily contact for a period of time while others only had three required contacts. All three studies mentioned that contact was as per requirement but in addition to routine reviews.
**9. Tailoring**	All three interventions mentioned some tailoring to the need of the participant. One varied the mode of assessment (postal, telephone or face-to-face) to need. The frequency and nature of contact was tailored to need as were the referrals to services in all three interventions.
**10. Modifications**	Only one study described modification, this was to care protocols during the research project.
**11. How well (planned)**	One study did not describe any steps taken to measure fidelity or promote adherence. Two interventions described the documentation of adherence to treatment or appointment by participants and reasons for not adhering. The process of care was described as collected in one study as was physician co-operation in adherence.
**12. How well (actual)**	One intervention described how 42 participants were contacted to establish reasons for non-adherence. Another inferred that the bulk of participants received the minimum required visits (n=3), as three was the mean average number of visits received. In the other study detailed descriptions were made of adherence by both participants and the professionals involved. Five thousand six hundred and ninety-four recommendations were made across all participants with an average of 28.8 per participant, over half of these were not fully complied with although adherence was stable across the duration of the study. Major problems were more likely to be identified in the first year of involvement, while therapeutic and preventative recommendations were similar over time. Adherence was better from physicians than referrals to other professionals or community services or those requiring self-care.

Group: Education, multifactorial-action and review with medication review and self-management strategies

There are five interventions in this group: Coleman 1999 [21], Counsell 2007 [22], Meng 2005 [59], Metzelthin 2013 [61], and Stuck 2015 [81].

TIDieR item	Description
1. Brief name	
2. Why	Goal: The goals of the five interventions were closely aligned, though differently described. Three interventions were clearly seeking to improve the health, function and quality of life of older people, two of these sought to do this by identifying risk factors for decline, two of these three also sought to promote self-management of health. Of the other two interventions one sought to change how primary care was delivered, increasing ancillary support to manage unmet needs in the chronically ill. The other was focused on improving geriatric care, driving down costs and reducing long-term care admissions. Reducing care costs was mentioned by three studies in all.<br><br>Rationale: All studies were rationalised through previous research, three through existing study findings and four through reviews. At least three had also got a theoretical grounding, often in behaviour change theory. One had used intervention mapping from existing findings. One study was heavily grounded in the idea of person-centred care and the need to manage frequently undiagnosed geriatric syndromes. One study was also informed by policy recommendations.
3. What (materials)	A range of materials were used in the five interventions. Four studies described training materials and protocols for providers. Two of these and the remaining other used treatment strategies for specific conditions. Two interventions utilised existing health records while one described their use of validated assessments. At least two studies described referrals and communication with professionals. One mentioned case management and care planning materials. Two interventions explained the input of the participant, one of these provided self-management videos to study participants, another used motivational interview materials.
4. What (procedures)	All five interventions were based around an initial assessment of needs, four of these were described as multidomain, one of which was formulated from electronic software designed to draw information from pre-existing patient records. Information from assessments was discussed by a multidisciplinary team in four interventions, three of which explicitly described planning from this assessment. Four interventions explained the review process and timing which varied. Three of these and the remaining other described the provision of self-management advice, though again the approach to delivering this varied. One intervention mentioned the process by which pharmacist input was implemented, though all assessments incorporated an aspect of medication review. One study described the provision of assistive devices, and one detailed the input of family members in care planning and post intervention care planning. Four studies detail usual care access.

**5. Who provided**	All five interventions had major input from nurses, four of these had GP or primary care physician input as well. Other professionals were involved to varying degrees including Physiotherapists, Occupational Therapists, Pharmacists and Social Workers. Multidisciplinary input was described to varying degrees as being when it was required. Three studies explicitly refer to the provision of specialist training on delivery of the intervention.
**6. How**	For one intervention, provision was not reported. For the remaining four, all were provided face-to-face and at home with some provision over the telephone in all instances. Three interventions were provided individually, one of these with a family care giver if one was available.
**6b. How organised**	Organisation of the five interventions varied, although all five required organisational input from nurses to some degree. Three of these also required organisational input from GPs or primary care physicians, these same three place responsibility on the multidisciplinary team for organisation of sessions and ongoing care needs. Two studies described case conference sessions while one detailed the organisation of clinics.
**7. Where**	Three interventions were undertaken in the USA, one in The Netherlands and one in Switzerland. Four interventions took place in the participant's home, while the other was carried out in clinics and practice rooms at the health care facility undertaking the study.
**8. When and how much**	A variety of inclusion and exclusion criteria were listed for the studies. Three had minimum age limits, two of these being 65 years of age and over and one being 70 and over. Three studies required the participant to be identified as at risk of increase care needs, two included frailty status as part of their inclusion criteria. One included those with a reduced income level, while two others were limited to specific primary care practices. One study excluded those with limitations on basic activities of daily living, cognitive impairments or terminal diagnosis, while two others included those with some limitations on (instrumental) activities of daily living.
**9. Tailoring**	All five studies described how the intervention was tailored to the participants needs in line with their assessment. Three also incorporated the preferences of the participant. Four interventions provided contact levels ad hoc so these were also varied. The intervention with group sessions tailored discussion to the needs of the group.
**10. Modifications**	Only one intervention described modifications made during delivery. This was necessary due to changes to funding and reassignment of services. Steps were taken to ensure that delivery was as close to randomisation as possible.
**11. How well (planned)**	All five studies described steps taken to improve adherence and measure efficacy and fidelity to the intervention, although some to a minimal degree. Four studies detailed the use of trained providers and two of these also used ongoing supervision to improve fidelity. Two also used existing record keeping processes to improve adherence. Two interventions included a process evaluation, one of which had been supported by feasibility work. One other intervention had also undertaken feasibility studies.
**12. How well (actual)**	Studies had varied success with their intervention implementation. One study found that although no benefit could be shown in results, participants expressed satisfaction with the intervention. One other study reported high levels of adherence to meeting requirements and care planning, suggesting that non-adherence to care planning was often

	related to participant reluctance. A third study found that most aspects of the intervention were well complied with, and home visits were well received, however some aspects were not complied with by providers or participants. This was similarly seen in the fourth study, where implementing the full protocol was problematic, assessments were conducted but care planning not always successful. Though participants were most often considered as committed to the plans. For the final study, which was undertaken through self-assessment, over 85% of assessments were returned and almost 60% of participants remained in the project for a full two years, although some aspects of the intervention were not as successfully adhered to as others.

Group: Education, multifactorial-action and review with self-management strategies.

There are two interventions in this group: Hattori 2019 [100], and Moll van Charante 2016 [62].

TIDieR item	Description
1. Brief name	
2. Why	Goal: One case was focussed on improving independence by encouraging self-management skills whilst the other case was focussed on reducing the incidence of dementia and cardiovascular disease, and the burden of functional disability in the elderly.<br><br>Rationale: Both cases were based on previous research. One case was based on the association between vascular and lifestyle risk of dementia and the potential to prevent dementia if risk factors are agreed. The other case on the other hand was based on effectiveness of multicomponent interventions
3. What (materials)	Varied devices used in both cases. Both cases used equipment to measure care goal activities. One case used original assessment for comprehensive clinical assessment, assessment sheet for self-management and booklet for preventing long-term care needs. The other case used detailed protocol which guided recommendations and referrals.
4. What (procedures)	Both cases started with comprehensive clinical assessments and a joint discussion of care goals and planning. Both cases involved training of staff during intervention provision.
5. Who provided	One case had intervention provided by a rehabilitation specialist such as an OT or physiotherapist with training by a care manager. The other case involved a practice nurse with supervision of a GP. Both cases had other professionals like dietitians, dental hygienists and other specialised health professionals also participating when required.
6. How	One case was delivered individually but the other case was presumed to be individually delivered.
6b. How organised	In all cases, mechanisms were in place to facilitate care coordination including meetings to discuss patients' goals with at least one case

	conference and a practice nurse under supervision of a GP who coordinated the intervention.
7. Where	One case was in The Netherlands in general practices organised in health centres and the other case was in Neyagawa, Osaka, Japan in a long-term care insurance system for people with mild to severe disability. One case was delivered face-to-face and the other case was presumed to be delivered face-to-face.
8. When and how much	In one case, the intervention was for five months which included one home-visit, up to 12 modules weekly lasting two to three hours and one review module. The other case was a nurse-led intervention every four months for six years and a total of 18 visits to the GP.
9. Tailoring	The care plan for both cases were tailored based on the participant's needs assessment and goals.
10. Modifications	Not mentioned in both cases.
11. How well (planned)	In both cases, measures were taken to promote fidelity through supervision and monitoring of the interventions. One case monitored the intervention through regular visits to the practice nurses. The other case did not state specifically who did the monitoring and supervision.
12. How well (actual)	One case had a high attendance rate with 76% attending at least one module and 66% attended at least seven modules. The other case had a relatively high drop-out rate with 544 participants receiving less than two visits per year before the end of study.

Group: Exercise

There are seven interventions in this group: Giné-Garriga 2020 [32], Morey 2006 (3 interventions) [101], Morey 2009 [64], Morgan 2019 [65], and von Bonsdorff 2008 [94].

TIDieR item	Description
1. Brief name	
2. Why	Goal: Two studies did not discuss the goal of their intervention. The remaining five all stated that a primary goal was to increase physical activity, one also mentioned the reduction of sedentary behaviour. Further aims included the improvement of health, function, quality of life and retention of independence, the reduction in disability and need for supportive services are also mentioned. One intervention explicitly refers to long-term behaviour change.<br>Rationale: Six studies refer to a theoretical basis for behaviour change and motivational techniques. Two studies refer to reviews undertaken to ground their intervention, while three discuss evidence-based effectiveness. Two studies were based upon feasibility or previous interventions. The benefit of motivational support was highlighted in three studies and at least three used recommendations and guidelines for healthy physical activity to ground development of their intervention.
3. What (materials)	A variety of materials were used across interventions. A core component was the provision of written materials to the participant, in five

	interventions these were based on physical activity promotion, either exercise tips or advice about physical activity services in the area. Two interventions provided materials unrelated to physical activity such as general health promotion. Four interventions mentioned activity planning in their materials used. Access to care notes to include activity planning or to gather information as required in five interventions. Three studies provided pedometers to participants, two provided training equipment. Documentation to track activity was provided to participants in two studies. Progress reporting was mentioned in two interventions. Referrals were mentioned in one study. Three studies mentioned manuals and scripting or fidelity assessment in their reports.
**4. What (procedures)**	All seven interventions involve the development of a tailored plan to promote physical activity, five studies explicitly mentioned input from participants on the development of this. Two studies also describe a focus on strength and walking or balance training. A pedometer was provided in two interventions. Support to set and maintain goals was mentioned in five studies. Follow up support and review were also mentioned by at least five studies, although how this was provided (either by post, phone or face-to-face) was not always clear. The provision of health-related information was mentioned by three studies, three studies explicitly refer to behaviour change techniques. Usual care was mentioned as available in three interventions. One study mentioned using referrals as part of the intervention.
**5. Who provided**	The intervention was provider by a range of individuals. Six studies detail the need for providers to be trained in the specifics of the intervention. Five interventions had input from primary care physicians or GPs and four from health counsellors. Two studies involved nurses or healthcare workers. Others involved in delivery included a qualified fitness instructor and a physiotherapist.
**6. How**	All seven interventions were provided individually and face-to-face. Five interventions also use telephone contact and two used the postal system. Group contact was mentioned in two interventions and one explicitly refers to the follow up process. One intervention preferred participants to have the support of a family member or loved one. Motivational interviewing and strategies for motivation, problem solving, goal setting and self-management are mentioned as key in five studies.
**6b. How organised**	One study did not mention how the intervention was organised. The six other interventions involve primary care providers or GP input, counsellors had an organisational role in three interventions and participant input was required in four interventions. A university, trained facilitators, the physiotherapist, and the local health and social services had input in one intervention. It was noted in three studies that the intervention was underway during a time of change in health promotion services.
**7. Where**	Interventions were delivered in a range of locations; although it was not always clear what was undertaken in each place. Veterans' health clinics are mentioned in fours interventions, primary care centres are mentioned in three, additionally, GP surgeries and leisure centres are identified locations in one intervention each.<br><br>Four interventions were delivered in the USA. The remaining three were in various European locations, one in the UK, one in Finland, and one was

	delivered in four countries including Denmark, Northern Ireland, Germany and Spain.
8. When and how much	The criteria for inclusion in the intervention were varied. Five interventions carried a minimum age, one was 65 years and over, the remaining four were 70 years and over. Four involved veterans only, one recruited old volunteers. Three studies assessed physical ability to safely take part and three required participants to have high levels of sedentary behaviour. Two studies required participants to not have dementia or at least be cognitively intact. Fours studies excluded those with high physical activity levels. Four excluded those with terminal diagnoses or specific health conditions.<br>The nature, duration and frequency of input varied over the interventions. One intervention provided 32 face-to-face exercise sessions, twice weekly for 16 weeks. However, the majority (four) only mentioned one face-to-face contact. Telephone contact varied between three and 13 calls. One intervention mentioned mailed updates. Intervention duration ranged from 16 weeks to two years.
9. Tailoring	All seven studies detail some level of tailoring in line with the participant's ability and capacity. One described tailoring following progress by the participant. One suggested the involvement of friends or family was optional.
10. Modifications	Not mentioned for any of the studies.
11. How well (planned)	One study did not mention how they were promoting adherence or measuring implementation fidelity. Adherence to the intervention was promoted through the telephone contacts in at least two interventions. Involvement of the primary care provider was also seen as beneficial to adherence in one study. Implementation and fidelity to the intervention was measured and analysed in at least two interventions. One study explicitly mentioned the use of qualitative approaches such as interviews to evaluate the intervention.
12. How well (actual)	One study did not mention how well the intervention was implemented. Four studies mentioned positive endorsement by the service provider. Three interventions report on flexibility with phone call delivery. Two interventions report that all participants received baseline input. One described minimum dosage input being received by all participants. One study stated that the anticipated duration of delivery was as intended. Call delivery was above 90% in one intervention, another reported that at least 302 of 318 participants received a minimum of four calls.

Group: Exercise and psychology

There are three interventions in this group: Alegria 2019 [7], Jing 2018 [102], and van Heuvelen 2005 [88].

TIDieR item	Description
Brief name	
Why	Goal: to improve physical and psychological health / reduce physical and mental disability.

	Rationale: Previous demonstration of effectiveness of each component, including psychological benefits of physical exercise. The combination was expected to provide further additional benefits.
**What: materials**	Some provided equipment and instructions for the exercises and materials to support the psychological tasks.
**What: procedures**	All the interventions provided exercise sessions and psychological training.<br>One intervention additionally provided encouragement calls to continue practising.<br>One intervention additionally provided regular mood screening.<br>Staff were provided with training prior to intervention in two studies, and regular supervision in one.
**Who**	In some cases, specialists in exercise or psychological training provide the relevant component. In others the provider was a community health worker or nursing student.
**How**	Both physical exercise and psychological training were provided individually and in group sessions in different combinations.<br>The interventions included face-to-face contact as well as telephone calls. In one intervention, the two components were provided as parts of one session.
**How organised**	Few details of organisation provided.
**Where**	China, The Netherlands and USA.<br>The intervention took place in community facilities and at the participant's home.
**When and how much**	Started in different circumstances:<br>(a) participants did not have cognitive impairment and were not very active;<br>(b) participants were housebound;<br>(c) participants had low mood and mild-moderate disability.<br>Physical exercise session frequency was between three times per week and once every two weeks for approximately 3 months. Sessions continued at greater or lesser frequency or not at all after this for an additional 6 weeks to 3 months.<br>Psychological training occurred for 18 weeks to 6 months, at a frequency of every 2 to 2.5 weeks for at least 3 months, with step-down to monthly training for the last 3 months in one.
**Tailoring**	The psychological training was tailored to individuals' problems in two interventions. Optional remote delivery was available in one intervention depending on participants' circumstances.
**Modifications**	Not mentioned
**How well (planned)**	One provided feedback on delivery, which was recorded. One encouraged participation by offering transport and sending newsletters. Encouragement calls or personal attention were also detailed in two interventions.

How well (actual)	Most participants did not attend all sessions in the two studies that reported details.

Group: Homecare

There are 12 interventions in this group: Auvinen 2020 [103], Bernabei 1998 [104], Dupuy 2017 [105], Fernandez-Barres 2017 [106], Fristedt 2019 [107], King 2012 [108], Lewin 2013 [109], Mann WC 1999 [110], Rooijackers 2021 [111], Teut 2013 [112], van der Pols-Vijlbrief 2017 [113], and Wolter 2013 [114].

TIDieR item	Description
1. Brief name	
2. Why	Goal: Only three of 12 interventions mentioned an explicit Goal, where the goal was to provide care and support to older people enabling them to stay at home. One added the goal of continuity of care for older people.<br><br>Rationale: Ten studies made no mention of the rationale for the intervention, one mentioned the importance of responding to the needs of older people and the other identified that caregivers were an important resource for the care of older people.
3. What (materials)	Eight studies did not mention the materials used for the intervention. One intervention mentioned the use of fake sensors as this was a control group, another mentioned care plans to identify support needs, one mentioned likely equipment for carrying out usual care and one other provided participants with a healthy diet brochure.
4. What (procedures)	In all interventions there was some reference to the provision of usual care, including home care services. Two studies mentioned assessments being carried out as part of usual care practice and one of these interventions developed care plans from this assessment.
5. Who provided	Intervention provision was by a range of practitioners. In four interventions this was by nurses, one of these was supported by a doctor. Four other interventions were provided by paid care support workers. External co-ordination was mentioned in two other interventions. Five interventions mentioned the input of a range of health and social care professionals to carry out care as required.
6. How	Delivery was not always described. Eight interventions were delivered individually and ten face-to-face. One intervention mentioned that some group input may be part of some intervention input.
6b. How organised	In one study the organisation was not described. Of the remaining 11, four were organised by home care providers, two had external coordination, two had state input mentioned in reference to organisation. Other individuals mentioned include nurses, home care staff, care providers and nurses.
7. Where	One study did not state a location of provision. Of the remaining 11, eight were undertake in European locations, including Germany, Spain, The Netherlands, Finland, Sweden, Italy and France. One intervention was based in the USA, one in New Zealand and one in Australia. Whilst it was not always explicitly mentioned the nature of provision suggests that the intervention was provided in the participant's home.

8. When and how much	There were various inclusion and exclusion criteria for eligibility to the intervention. Ten studies mentioned that participants needed to be in receipt of usual care. Additionally age limits of age 65 and over or age 75 and over were prerequisites for eligibility in three instances. Additional requirements for inclusion were that no previous assessments were undertaken on the participant, that they had high level need or polypharmacy, that they were house bound, that they had a frailty level which indicated decline over the preceding six months, that they resided within a certain housing community or that they were undernourished. Only three interventions specified exclusion criteria, one excluded those with the highest level of need, two excluded those with cognitive impairments, one of these also excluded those who were terminally ill or bedbound.<br><br>The nature and frequency of contact was rarely described, for four studies it was and then it was assumed that this would vary according to need.
9. Tailoring	Four interventions mentioned that provision would be tailored according to the need of participants. The remaining eight did not describe tailoring.
10. Modifications	No studies mentioned modification to their intervention.
11. How well (planned)	No studies mentioned steps taken to improve implementation or adherence to the intervention.
12. How well (actual)	Not mentioned for many studies though one found that home care staff promoted reablement principles that were not part of the intervention.

Group: Homecare and multifactorial-action

There are five interventions in this group: Parsons J 2012 [115], Parsons M 2012 [116], Parsons M 2017 [117], Tuntland 2015 [118], and Whitehead 2016 [119].

TIDieR item	Description
1. Brief name	
2. Why	Goal: Although the goals of the interventions were differently described the overall focus was to enable older people who were identified as requiring support to live at home, to maintain home living. Identification of the appropriate level of care appeared to be a key aim across interventions. The promotion of health-related quality of life, independence and social connectedness was mentioned as an explicit aim of one intervention. Rehabilitation as set at an appropriate level was mentioned by another. One other mentioned reducing care costs was an aim.<br><br>Rationale: Two studies did not explicitly state a rationale. One was somewhat ambiguous stating that there was a need for appropriate home care service provision. One intervention was based on evidence that older people often lose function when in hospital and those who do lose function often fail to regain it. This intervention suggests that home care has the potential to improve this situation. One intervention was based on Care Act guidelines around care provision and that a key component of this was empowerment and reablement.

**3. What (materials)**	Materials were minimally described in the studies. Three of the interventions described using standardised assessments. One mentioned the development of care planning with client input. Two others described accessing services or equipment through referral systems as required.
**4. What (procedures)**	The process of the intervention was not always clearly described. Four interventions described an assessment process; one of these was at a six-week time point to identify continuing needs. Care planning following from the assessment was stated by one intervention. Four interventions explicitly refer to access to standard home care service provision and other healthcare services remaining in place. One intervention described little other than to indicate that the care package was designed to include input from family and community services.
**5. Who provided**	Assessments were conducted by needs assessors and processed by healthcare co-ordinators for two interventions. One of these also mentioned the input of the research team. Home care aide input was mentioned explicitly by two interventions. The role of those undertaking assessments in not clearly specified in three interventions. One intervention mentioned reablement workers and social care managers. Input from additional healthcare professionals as required was mentioned in the delivery of all five interventions.
**6. How**	It was not always clear how assessments were undertaken, two interventions mentioned this being an individual assessment but with no indication that it was face-to-face. Three interventions mentioned the provision of care being face-to-face and individual in nature. One other clearly stated that provision of care was face-to-face and at home.
**6b. How organised**	A range of organisations and individuals were involved in the organisation of the interventions, three interventions utilised an assessment agency with needs assessors to undertake assessments. One of these interventions also had organisational input from the research team, home care co-ordinators and home care aides. Two interventions had healthcare organisation input, one of these studies mentioned funding by the health district board. One intervention mentioned organisation by the relevant healthcare professional providing care. Another was organised by reablement workers for an initial six weeks, then an Occupational Therapist and home care service should continued care be required.
**7. Where**	Three interventions were implemented in New Zealand, one in Norway and one in the United Kingdom.<br><br>Four interventions mentioned delivery of the intervention at home.
**8. When and how much**	Eligibility to all five interventions was after referral to home care services. One of these required this to be on hospital discharge.<br><br>The nature, duration and frequency of intervention input was not always mentioned. Two interventions note that input frequency and duration was varied. One stated that there was no time limit to input. However one other conducted six weeks of reablement followed by homecare as required after this time point.
**9. Tailoring**	All five interventions were tailored in line with the needs of the individual. One mentioned consideration of the preferences of the individual, whilst another was tailored to the effort given by the individual. Three mentioned flexibility over the duration of input.

**10. Modifications**	Four studies did not mention any modifications. One intervention required modifications to be made following changes to the recruitment approach of Occupational Therapists in the service.
**11. How well (planned)**	Three studies did not mention any plans for adherence or fidelity assessment. One study described the collection and analysis of the care planning documentation. The other conducted a cost analysis identifying the number of contacts, the provision of equipment and individually reported additional service use.
**12. How well (actual)**	Three studies did not undertake adherence or fidelity assessments. One intervention found that 15% of care plans documented individualised activity related to functional improvement. The other intervention remarked on changes to the intervention due to changes in the recruitment of staff.

Group: Home care, ADL, multifactorial-action from care-planning and review with self-management strategies

There are three interventions in this group: King 2012 [108], Parsons M 2017 [117], and Rooijackers 2021 [111].

TIDieR item	Description
**1. Brief name**	
**2. Why**	Goal: The three interventions all aimed to improve, restore, promote or maintain independence or function. One intervention also had a goal of improving wellbeing of participants. All three interventions desired improved service provision. One intervention aimed to reduce admission to long-term care.<br><br>Rationale: All three interventions were based on previous studies or existing models of working which showed benefit to older people. Studies mentioned a theoretical basis in the evidence for restorative approaches and social theories.
**3. What (materials)**	All three interventions used a range of assessments over a number of domains, one explicitly referred to psychological, social and physical components. Two studies detailed training materials for staff and one intervention mentioned goal setting documentation, action planning documentation and exercise booklets for participants.
**4. What (procedures)**	All three interventions required a multidomain assessment to be undertaken, all three were also co-ordinated by a nurse. Goal setting, care planning and tailored exercise planning was also part of all three interventions. Regular review, referrals and staff training are each mentioned as part of one intervention. Usual care was available across all interventions.
**5. Who provided**	Registered nurses and support workers are involved in the provision of all three interventions. One intervention involved multidisciplinary input as well. Training was important for providers of all three interventions.
**6. How**	All three interventions were provided face-to-face, three mentioned this being on an individual basis. One intervention mentioned telephone contact as well.

**6b. How organised**	All three interventions appear to have nurse co-ordinators as a core organisational feature. Support worker input was key across all three interventions as well. Hospital staff were involved in one intervention, a physiotherapist or occupational therapist in another. Funding was through healthcare insurance for one intervention while one other mentioned input from charitable services.
**7. Where**	All three interventions were delivered at home. Two interventions mentioned involvement of one key healthcare provider.<br><br>Two studies were undertaken in New Zealand, one in The Netherlands.
**8. When and how much**	All three interventions required participants to be in receipt of home care. One had an age limit of 65 years and over. One recruited those with high levels of need placing them at risk of long-term care admission. One study placed a language restriction on participants. Only one study excluded those with serious or terminal illness or cognitive impairment.<br><br>The duration and frequency of contact varied across the three interventions. Contact ranged from multiple daily contacts to a minimum of once a fortnight. One mentioned four-six months of input, and one mentioned 12 months duration. Two interventions mentioned reassessment, one at 12 months one at six.
**9. Tailoring**	All three interventions included tailored care planning according to the assessment of participants. Two mentioned this being in conjunction with the participant. One described the adaptation of visits according to need.
**10. Modifications**	This was not mentioned by any of the studies.
**11. How well (planned)**	One study did not mention any attempt to assess implementation or fidelity. The two other studies both delivered training and support to intervention providers. One study undertook feasibility work. One promoted adherence through prompts to providers. One study detailed a process evaluation through the collection of documents about records and qualitative methods.
**12. How well (actual)**	One study did not mention any attempt to assess implementation or fidelity. A variety of findings were reported by the remaining two studies. One was delivered during the expected timeframe, follow up calls were received between 70-89% of the time, although over 50% of initial assessments did not identify tasks. The other intervention described barriers to implementation such as low staffing and resistance from clients, whilst additional funding and digital care planning facilitated implementation. Compliance measured as 73%-86% for attendance at over half of the meetings, over 50% of assignments were completed by team members. Staff were noted as perceiving change as positive due to the intervention.

Group: Homecare, multifactorial-action and review

There are six interventions in this group: Hall 1992 [120], Markle-Reid 2006 [121], Parsons M 2012 [116], Ryvicker 2011 [122], Ryvicker 2011 [122], and Shapiro 2002 [77].

TIDieR item	Description

1. Brief name	
2. Why	Goal: The six interventions had a number of goals, four of which promoted the improvement of function in older people with some level of dependency through home care to enable independent living for as long as possible. Two studies indicated that reducing service duplication and integration of services by promoting better across service communication as additional aims. Improving the home care service by optimising the role and enhancing retention was an aim of one other intervention. One other intervention indicated the early input of interventions to promote proactive care as a goal.<br><br>Rationale: one study did not describe the rationale for their intervention. Two interventions suggested that integrated care approaches appear beneficial to support the holistic care neds of older people. Two interventions were based on previous research. One intervention suggested that that are barriers to providing successful home care and this impacts on home care worker retention and the outcomes of those using the service. One other study identified the potential that home care provision has to improve the wellbeing of older people.
3. What (materials)	Two studies did not describe the materials used in their intervention. The remaining four all described a variety of assessments for older people, some of these were routinely undertaken in usual care, others were specifically developed for the intervention. One intervention also accessed medical records to complete assessments. One intervention developed guidance called 'Five Promises' to aid with communication between older people and the staff providing care. One intervention also described documentation used in the supervision of staff who delivered the intervention.
4. What (procedures)	All six interventions used an assessment to identify needs, one of these specifically involved patients' preferences. Care-planning was explicitly developed from this assessment in four interventions, one involving family caregivers in this process. A further four described the review process, with one again including patients' preferences in this. Services were arranged as part of three interventions. Training and supervision are described in the reports of two interventions. Additionally, access to usual care was noted for four interventions.
5. Who provided	A range of professionals were involved in the provision of the interventions; many by multiple individuals and roles. Nurses were involved in three interventions and case workers in three as well. Access to a multidisciplinary team was mentioned in four interventions, though this was sometimes in conducting assessments and at other times in carrying out care. A personal support worker was mentioned in one intervention. Another mentioned the need to ensure providers were trained in intervention delivery.
6. How	One study did not describe how the intervention was delivered. Four interventions were delivered face-to-face, four at home and four individually (although these were not always the same four). One intervention also used telephone contact and another required input from a caregiver.
6b. How organised	Organisation was variable. One intervention required little planning and organisation. Two interventions relied upon case managers to organise the intervention; clinicians were involved in care planning for three interventions. Nurse input was described as key in three interventions as was the home care team in two of these. The family caregiver had some input in one intervention.

7. Where	Five of the six interventions were undertaken in North America; two in Canada, three in the USA. The remaining intervention was carried out in New Zealand. Four of the six interventions were delivered at home, one mentioned specific care settings, whilst the other did not state the location of intervention delivery.
8. When and how much	All six interventions commenced following on from assessment indicating that the older person required home care. Two of the interventions required evidence on the assessment for capacity for improvement, whilst one other required a specific level of ill-being. One intervention had a minimum age requirement of 75 years of age.<br><br>The nature, frequency and duration of the interventions was not well described, four studies described an initial assessment and then all six refer to review of this assessment, although for some this was at specific time points and others it was ongoing or as required. Only one study described duration, and this was 18 months long.
9. Tailoring	All six interventions were designed to be tailored to the needs and capacity of the participant identified at assessment. Two interventions tailored according to participant's wishes also. One intervention was tailored to the caregiver as well; this intervention also tailored the supportive contact according to need.
10. Modifications	Four studies did not mention any modifications. One mentioned that there were changes to the criteria to assess support changed during implementation, impacting on the service provision. One study mentioned that there were changes made to the hours provided by home care over the duration of the intervention.
11. How well (planned)	Only two studies described steps taken to assess fidelity and adherence, the remaining four did not. The two that carried out a survey on the intervention, used both standardised intervention materials and training materials for providers. They both also conducted interviews with delivery team managers and carried out observations of meetings.
12. How well (actual)	Four interventions were not assessed for fidelity. One study stated that components of the intervention were widely accepted, although some components were not considered feasible the 'Five Promises' guidance was seen to show benefit. Training was seen to be inconsistent and Clinician and commitment to support the intervention was variable. The other study described how there was little capacity to implement the intervention.

Group: Homecare, multifactorial-action from care-planning and review with medication review

There are three interventions in this group: Bernabei 1998 [104], Fristedt 2019 [107], and Wolter 2013 [114].

TIDieR item	Description
1. Brief name	
2. Why	Goal: All three interventions had a goal of reducing institutional admissions be that to hospital or long-term care, one also sought to reduce the cost of providing care to older people. Two studies mentioned the aim of improving function, one study implied this would improve quality of life for older

	people. One study described the need to improve communication between services.<br><br>Rationale: One study mentioned the need to integrate social and medical services to provide clarity over their purpose, this intervention was also grounded in policy recommendations and the wishes of older people themselves. The two other interventions were developed following on from previous research showing the benefit of such interventions. One of these was part of a government policy to improve quality of care, the other was to identify deficits in current care processes.
3. What (materials)	All three interventions included assessments of need, though these varied in type they were designed to cover multiple domains of need, with a requirement for sufficient data to develop a care plan. Two interventions required access to existing medical records, one of which also required agreement for care planning with the participants GP. Two interventions mentioned equipment required by staff such as transportation and a laptop in one intervention, and a protocol for conducting assessments in another.
4. What (procedures)	All three interventions conducted multidomain assessments. Two explicitly refer to care planning from this. One intervention described the monitoring process following assessment in detail while the other two mentioned reviewing. Two interventions mentioned the staff training process and one described the need for agreement of care plans with the participants care providers. All interventions included access to usual care services, although one did replace some existing service provision with the intervention multidisciplinary team.
5. Who provided	One intervention was provided by a case manager and the participants GP as well as a specialist trained multidisciplinary team. A multidisciplinary team provides one other intervention, while the third was provided by home care nurses and staff. All studies mentioned the input of multidisciplinary team members as per usual care needs.
6. How	All interventions were provided individually and face-to-face. One intervention mentioned that some of the services accessed may be in different locations and in group contexts as was relevant. Another intervention required collaboration with participants and/or their relative(s).
6b. How organised	The interventions were organised differently. Although two interventions relied upon some level of state involvement. One of these involved input from existing services, case managers and the GP and used weekly sessions to discuss the intervention implementation. The other of these the geriatrician for the intervention took over the primary care responsibilities for the participants. The third intervention was organised by the home care service and nurses.
7. Where	All three interventions were undertaken in Europe, one in Germany, one in Sweden and one in Italy. All three were implemented at home. Two studies mentioned the system of care in which they were operating, this was varied, and integration of services was mixed.
8. When and how much	Two interventions were accessible to participants upon receipt of home care services, although one of these specified no previous assessment and planning to be undertaken. The other interventions criteria were

	participants being over 75 years or age and having a level frailty which indicated decline over the previous six months.<br>The nature, duration and frequency of input was not described in detail by the studies. One intervention lasted for 12 months with input over alternate months; another intervention had an assessment visit then varied amounts of contact over the following 15 weeks. The other study did not mention input explicitly, although reassessment was mentioned.
**9. Tailoring**	All interventions could be tailored to the needs of the participant, one was also tailored to the needs of the relatives. Two interventions also mentioned the provision of support from providers being tailored to needs and wishes of participants.
**10. Modifications**	There were no mentions of modifications to the interventions in any studies.
**11. How well (planned)**	Two studies did not mention any steps taken to improve fidelity or measure adherence. However, one explicitly mentioned that staff providing the intervention received training and advice, and support was on hand to improve fidelity.
**12. How well (actual)**	Two studies did not describe fidelity or implementation of the intervention. The other study however described how the intervention saw an increase in the level of care planning and in keeping care plans up to date. Although, implementation of the intervention varied between providers, some being able to implement well or rapidly (optimal) and others unable to implement even over a longer period of time (sub-optimal). Nurses feedback indicated that a year was needed to implement. Further analysis indicated that there were certain factors associated with improved implementation including services with higher levels of qualified staff, staff having lower workloads, and smaller services were more likely to implement well.

Group: Homecare, multifactorial-action and review with self-management strategies

There are two interventions in this group: Hall 1992 [120], and Parsons J 2012 [115].

TIDieR item	Description
**1. Brief name**	
**2. Why**	Goals: The goals of the two interventions were broadly aligned. One intervention stated assisting frail older people to live at home for longer and sustain their total wellbeing as a goal, the other focused on the restoration and maintenance of function as well as engagement with community services. Both interventions focused on the empowerment of older people to take control of their own lives, one intervention also wished to change the philosophy of home care provision from increasing dependence to promoting independence.<br>Rationale: one study was based on previous work which showed a gap in existing home care interventions. The other intervention was also based on existing evidence, suggesting that hospitalised older people often lose

	function and then do not regain this once back at home, identifying home care as having potential to improve this situation.
**3. What (materials)**	One intervention used a protocol to guide care planning formulation and then referrals to services. The other intervention developed and used a specific tool called Towards Achieving Realistic Goals in Elders tool (TARGET), standardised assessments care planning and client reviewing are also mentioned as materials for this intervention.
**4. What (procedures)**	Both interventions use multidomain assessments the planning and arrangement/organisation of care and a regular review component. One intervention stated that review was monthly. Both interventions also used supported goal-setting and had access to usual care, and home care specific to their needs based on standard existing assessments. One intervention also mentioned the training of deliverers ahead of providing the intervention.
**5. Who provided**	The interventions were provided by different teams. One used nurses to conduct the assessment and carry out care. The other used trained needs assessors, home care coordinators, and home care aides as well as the research team in the delivery of their intervention. Both studies mentioned access to healthcare professionals as needed.
**6. How**	Both interventions are provided to individuals, however only one stated that this was face-to-face, the nature of the assessment for the other intervention was unclear.
**6b. How organised**	Organisation of the intervention also differed. One was organised by a nurse who provides referrals based on their assessment then community services arrange relevant services. The other intervention required the assessment agency to conduct needs assessments, the home care agency to coordinate this, and the coordinators to plan and review the relevant care to be provided by home care aides. The research team are also mentioned as having organisational input in one intervention.
**7. Where**	One intervention was conducted in Canada, the other in New Zealand
**8. When and how much**	Eligibility for both interventions was on enrolment or referral for personalised care at home. In both cases this was based on standardised assessments identifying this need, visits and support were according to need. One intervention mentioned specific review at three and 12+ months.
**9. Tailoring**	In both cases care planning was tailored to need and with preferences identified by the participant based on their multidomain assessment. One intervention also mentioned that usual care was also based on the need of the client.
**10. Modifications**	Modifications were not mentioned for either study.
**11. How well (planned)**	One intervention did not mention any plans for adherence or fidelity assessment. In the other intervention support plans and details of services accessed were collected and analysed, the number of reviews undertaken by home care coordinators was also gathered.

**12. How well (actual)**	One intervention did not report on adherence or fidelity. The other intervention identified almost 2/3rds of planning included activity targeting functional improvement. However, the review process was not increased by use of TARGET. 85% of participants engaged in goal setting and 10 referrals to allied health professionals were made.

Group: Meaningful activities and education

There are two interventions in this group: Clark 1997 [19], and Clark 2012 [20].

TIDieR item	Description
1. Brief name	
2. Why	Goal: The Interventions were both designed to benefit the physical, psychological and functional health of older people. One also mentioned attending to cognitive health as well. Both interventions had the aim of reducing decline; this was to be targeted through engaging people in meaningful activities. One study mentioned education to inform better health practice in older people. The other intervention mentioned that they targeted an ethnically diverse population, and a desire to embed the intervention in everyday routine.<br><br>Rationale: Both interventions were developed based on previous study, additionally both interventions mentioned occupation specifically as part of successful ageing. One of the interventions rationalised development through an occupational science theoretical basis, acknowledging that occupation is socially generative and productive. Previous study had been used to select components of this intervention for evidence-based benefit. The other intervention indicated that activity and lifestyle are modifiable factors for targeting change.
3. What (materials)	One study did not specify the materials they provided although it was noted that they were culturally adapted for the population. The other intervention provided educational materials including ‘25 ways to stay healthy’ which was developed by participants, a life redesign journal and an instructional video on crime prevention.
4. What (procedures)	Both interventions consisted of the same procedures. This was to provide educational sessions on various topics to groups of older people. Additionally individual education sessions were provided which could be tailored to the participant. Interventions provided opportunities to take part in activity sessions. Functional training was available to enable easier engagement with activities and usual care was also noted as available to participants.
5. Who provided	Both interventions were provided by occupational therapists, particularly trained in supporting older people. These were able to speak appropriate languages as required. One intervention also mentioned a session being delivered by a police officer.
6. How	Both interventions were provided face-to-face and using both group and individual settings. Interventions aimed to facilitate peer interaction and were using psychological approaches to ensure intervention efficacy.
6b. How organised	One intervention report mentioned the funding source as the National Institute of Health and the American Occupational Therapy Foundation. The

	other intervention suggested that continuity in provision was key, and that money was available to compensate participants for taking part in activities.
7. Where	Both interventions were undertaken in the USA. The individual components of the interventions were provided at home. The group sessions were provided at community-based sites for one intervention although the location of the group sessions in the other intervention was unspecified.
8. When and how much	One intervention was available to older people living in specific locations following a health assessment by a physician. The other intervention was undertaken with people over 60 years of age, recruited through various approaches within a specific location, for example targeting senior housing and community centres. Both interventions were aiming to reach culturally and ethnically diverse populations where there was an assumed health disparity.<br><br>Both interventions provided two hourly group sessions once a week, one provided hourly individual sessions once a month, the other made up to ten hours of individual sessions available, this intervention lasted six months the other nine.
9. Tailoring	Both interventions were tailored according to the needs and activity preferences of the individuals. These activities could be adapted over the intervention period. Both interventions could be tailored to the language of the participant.
10. Modifications	Neither study detailed modifications made to the intervention.
11. How well (planned)	Both studies detail efforts to maintain fidelity and assess adherence to the intervention. Both studies described training to the providers. One intervention also asked participants to refrain from speaking to each other about their activity involvement to avoid contamination across activities. One intervention took steps to ensure that providers were continuous across the intervention delivery. In addition reminders were sent about activities taking place and contamination across activity provision was measured.
12. How well (actual)	For one intervention 65% of the participants attended at least half of the sessions. For the other intervention on average, participants attended 56% of the scheduled sessions. Whilst 17% of individuals did not attend any intervention sessions. There was some cultural variation in attendance, conflict was seen across participants, but this was well managed by intervention providers.

Group: Multifactorial-action

There are nine interventions in this group: Borrows 2013 [5], Botjes 2013 [13], de Craen 2006 [25], Grimmer 2013 [34], Hay 1998 [38], Siemonsma 2018 [2], Stewart 2005 (2 interventions) [123], and Williams 1992 [124].

TIDieR item	Description
1. Brief name	
2. Why	Goal: One study did not mention an explicit goal of their intervention, the remaining eight had a variable focus around promoting independence or preventing and/or delaying dependence or functional decline in older

	people. For three interventions this was also to maintain living at home, and for another this was to ensure that older people could continue to contribute to society. Three interventions had an additional aim of reducing the cost of care provision. One desired the promotion of health and wellbeing for older people.<br><br>Rationale: There were mixed rationales for the interventions, one study did not state a rationale. Four interventions were based on previous research or evidence in the literature. One of these was also theoretically driven by the perceived benefit of patient involvement in care decision making. Three other interventions were based on the perceived benefit of their approach, be that by utilising specific staff expertise or by implementing specific ways of working. Further rationalisations were evident including the need for working proactively, which was mentioned twice, adherence to policy recommendations or that certain patient groups were specifically at risk.
3. What (materials)	One study did not mention the materials that were used in their intervention. For six interventions assessment documentation, be that electronic or paper based, was described. Three interventions mentioned the provision of appropriate therapy, equipment or adaptations. Two interventions described communication with health care professionals and participants in their materials. One study required access to patient clinical records and referrals. The provision of information to participants was mentioned for one intervention; one other described the use of a protocol to guide care. As one intervention was based on the internet, a computer and internet access were required for this.
4. What (procedures)	All nine interventions described an assessment of need, for four interventions this was described in more detail as being multidomain. Six interventions described the implementation of appropriate recommendations, referrals, therapy or adaptations in line with this assessment. One of these six and one other mentioned care or action planning following assessment. Three interventions required the participants to have input on the solutions to their care needs. Three also mentioned access to usual care. Two studies mentioned processes designed to sustain the programme by developing community partnerships and improved communication between different support services.
5. Who provided	Eight of the nine interventions were provided by professionals. Three of these were occupational therapist led, one of these may have been an occupational therapist assistant at times. One intervention was physiotherapist led while another was provided by physiotherapists and occupational therapists. One was led by a research nurse, one by a health visitor and one by a social worker. The remaining intervention was conducted online however there was a volunteer on hand to provide support if required, although their background was not specified.
6. How	Not all studies clearly described how the intervention was delivered. In six studies it was evident that provision was face-to-face, and in five this was individually provided. One intervention was provided both face-to-face and over the telephone. Another intervention was less clear in detailing how it was provided, although the setting appeared to be clinically based. One intervention was conducted online with the option of support for those struggling to complete the assessment questionnaire.

**6b. How organised**	For one intervention organisation was not mentioned, for the remaining eight interventions there was varied input. State or local authority input was mentioned for three studies. The relevant provider such as the occupational therapist, social worker, research nurse or health visitor was responsible for organisation in most instances. Established care providers and GPs were also involved in three interventions. Additionally, the older person was seen to have some responsibility for organisation in two interventions.
**7. Where**	Seven interventions were undertaken in Europe, four in the UK, three in The Netherlands. One intervention was implemented in Australia and one in Canada. Six interventions were carried out at the participant's home, one mentioned attending clinic and one a location befitting the participants therapy requirements.
**8. When and how much**	The eligibility requirements for inclusion were varied, though four had a minimum age requirement, this ranged from 65 and over to 85 and over. Five specified that participants had to have an evident need or referral to services. Two were following hospital discharge, although one was upon discharge from emergency services and the other from inpatient care. One intervention required participants to have a level of frailty, although the assessment was unspecified, one recruited through a pre-existing cohort. Another specified that participants had mental capacity to be included.<br><br>The nature, frequency of input and duration of the interventions was rarely described in any detail. Contacts ranged from one to 18 occasions. The duration of the intervention was only mentioned for three interventions ranging from three weeks to six months.
**9. Tailoring**	All interventions involved tailoring to participants need. Two also mentioned tailoring of support level according to need and two others mentioned attending to the preferences of the participant.
**10. Modifications**	None of the studies described modifications to their interventions.
**11. How well (planned)**	Five interventions did not report taking steps to measure or promote fidelity or adherence. One intervention conducted a process evaluation of the experience of the intervention by participants. One documented compliance with referrals. Whilst two others promoted adherence and interest with regular contact and meetings.
**12. How well (actual)**	Four studies did not describe the success of intervention implementation. One intervention found that 60/109 participants were able to take part, and of these over 90% received a care plan. Non-compliance was explored and found to be related to lack of support or access to computer equipment. Although the experience was seen to be beneficial at times some of the suggestions were not welcomed by participants. In one other intervention 66/147 showed need, of which approximately 50% accepted support offered, of those who did not the proposed solutions was not seen as likely to help by participants. Another intervention identified that compliance for first appointments was quite good although uptake dropped off after this, however healthcare professionals rated compliance with the intervention as high for those who did attend appointments. For the two interventions which promoted adherence and interest with regular contact and meetings these were poorly attended.

Group: Multifactorial-action and review

There are 15 interventions in this group: Challis 2004 [125], Cutchin 2009 [23], Hattori 2019 [100], Henderson 2005 [40], Hendriksen 1984 [41], Imhof 2012 [45], Kono 2004 [48], Kono 2012 (2 interventions) [126], Kono 2016 [127], Lambotte 2018 [50], van Rossum 1993 [92], Vass 2005 [128], Vetter 1984 [93], and Williams 1992 [124].

TIDieR item	Description
1. Brief name	
2. Why	Goal: Not all studies identified a goal of their intervention, however the majority did. Goals most often focused upon older people, improvement of their health and function being a goal of seven interventions, quality of life and wellbeing a part of three of these and an aim of a further two. Supporting independent living was an aim of two, promoting self-care a focus of one other. The identification of needs was a goal of five interventions, one simply stating that older people had needs, two others identifying that these were often unmet and of a medical and social nature, two others suggested early identification of these needs was key. Other primary goals were to reduce health resource use in six interventions, in particular for long-term admissions in two studies. Four interventions also identified that accessing care and support was vital for older people.<br><br>Rationale: Not all studies were rationalised, and one was unclear in rationale. Eight interventions were based on previous research which showed benefit, four in particular focusing on proactive and preventative approaches to care. Evidence that unmet needs lead to acute care admissions was the foundation for two interventions. Two interventions had grounding in theory. One intervention was policy informed, one suggested that social care could manage many needs of older people better than primary care providers. One of the studies highlighted that they had developed their intervention collaboratively.
3. What (materials)	Four interventions did not describe any materials they provided. Seven described the assessment documentation they used, five used referrals and communications to other services. Four others mentioned documentation relating to summaries of the assessments, such as care plans or feedback to participants and families. Four interventions described the use of protocols, instructions, or manuals by providers. One intervention developed and used a coding system to aid with carrying out the intervention. One also described the loan of assistive equipment to participants.
4. What (procedures)	All interventions consisted of some kind of assessment, some described these in some detail, others used specific validated assessments, but all 15 were defined as covering multiple domains, such as physical, social, psychological and cognitive aspects. Six of these assessments resulted in the production of care plans in collaboration with participants, one of these also included family in care planning. Seven interventions provided information and advice to participants, five also provided referrals on to other services. Thirteen studies described the review process in some detail. Twelve interventions explicitly mentioned that access to usual care would be sustained for participants.
5. Who provided	The interventions were provided by a range of individuals. Eight interventions were provided by more than one person, the remaining seven

	appeared to be unidisciplinary. Ten interventions had some Nurse input although the specialisms of the Nurses varied, including Community Psychiatric Nurses, Advanced Practise Nurses, Public Health Nurses and Community Care or District Nurses. Social services personnel were involved in four interventions and Health Visitors involved in two. Occupational Therapists involved in two others, one of these also involved a Physiotherapist. Other professional input came from a medical student, a geriatric specialist, a Care Manager and a GP. Four studies also described the specific training given to intervention providers.
**6. How**	Although provision was not always described, face-to-face delivery was implied for all interventions. At least 11 interventions were provided to the participant at home, and 14 appeared to have had some individual provision. One intervention also notes that some group delivery may have occurred depending on the nature of the recommendations made to the participant. Six interventions also used telephone calls to contact participants, however one of these interventions required the telephone call to be initiated by the participant or their family member. One intervention also posted out recommendations and required the participant to act on those recommendations. One intervention explicitly stated that providers were continuous for each participant, to aid in building a rapport.
**6b. How organised**	Organisation of the intervention was by a range of individuals, however in at least 12 instances this was the professional providing the intervention, be that a Nurse, Occupational Therapist, Physiotherapist, Health Visitor or other professional. Funding was described for four studies; this came from the state and in one case was supported by research funds. Although many interventions described input of multiple professionals including GPs, four explain that decision making was to be unidisciplinary. Four interventions mentioned that the participant had to take responsibility for organising care in line with recommendations.
**7. Where**	Thirteen interventions were described as being provided in the participant's home.<br><br>Eight interventions were undertaken in Europe, three in the UK, two in Denmark, one in Switzerland, one in Belgium and one in The Netherlands. One intervention was undertaken in Australia, another in the USA and five others were carried out in Japan.
**8. When and how much**	For one intervention it was not stated when input commenced. For the other studies, inclusion and exclusion criteria were wide ranging. Five interventions were to start following assessment which indicated a specific level of need. Four were specific to location but that the participants own accommodation, their GP surgery or upon their discharge from hospital. One required their participants to be registered with the welfare authority. Seven had minimum age requirements, these ranged from 60 and over to 80 and over. One required a specific level of frailty in their participants and four required a level of ADL limitation but with capacity to ambulate. Participants were excluded if they had a severe cognitive impairment or dementia in two studies and were at the end of their life in of these. One other study excluded those who had used welfare services in the preceding three months in three studies.<br><br>The nature, frequency and duration of input was varied across interventions and was not always clear. The longest visits were up to two hours in length. The longest intervention duration was three years, while the shortest stated

	was nine months. Input over this duration was varied, 12 visits was the most contacts a participant could expect to have though most interventions ranged between quarterly and bi-annually contacts. Other contacts included scheduled and ad hoc telephone calls. One intervention appeared to have also included ad hoc visits as required and upon request.
9. Tailoring	One study did not mention any tailoring of the intervention, the remaining 14 all indicated tailoring of provision was in response to the participants needs assessment. Five interventions considered the preferences and wishes of the participant, one of these also considered family input. Four interventions tailored additional contact to need. One study mentioned that participants had the right to decline recommendations.
10. Modifications	None of the studies described any modifications made to the intervention.
11. How well (planned)	Seven studies did not describe if or how they took steps to improve or measure adherence to, or the efficacy of, their intervention. Three studies described supervision, training and monitoring of providers. At least three described the role of a detailed protocol and system of working in consistent delivery by different providers. One study ensured that providers could raise questions and queries to enhance their practice and one other carried out quality assessments on the data collection process. At least two documented contacts and actions made during planning. One intervention was piloted, and one other conducted qualitative investigation alongside the trial.
12. How well (actual)	Seven studies did not describe how well their intervention was implemented. Four interventions examined participant compliance with visits, all four suggested this was at least 60%, one was as high as 98%, although one other study found that the number of visits per participant varied widely in their intervention. Actions and recommendations from their assessment were measured in three studies, compliance was over 50% in two studies, however for the other intervention, almost 80% of the time no recorded action was made in a visit. Consistency in provision was considered good in one study, in another a provider left and had to be replaced and in a third the intervention delivery was very varied across the two providers.

Group: Multifactorial-action and review with medication review

There are 24 interventions in this group: Bouman 2008 [14], Brettschneider 2015 [15], Cesari 2014 [18], Challis 2004 [125], Dalby 2000 [24], Fabacher 1994 [26], Fairhall 2015 [27], Ford 1971 [29], Fox 1997 [129], Harari 2008 [37], Hebert 2001 [39], Hogg 2009 [42], Kono 2016 [127], Leung 2004 [51], Melis 2008 [58], Rubenstein 2007 [75], Stuck 2000 [80], Suijker 2016 [82], Thomas 2007 (2 interventions) [85], Tulloch 1979 [86], van Hout 2010 [89], Vass 2005 [128], and Yamada 2003 [98].

TIDieR item	Description
1. Brief name	
2. Why	Goal: Most interventions had the goal of maintaining and improving the function of older people, additional aims included improving quality of life and reducing negative outcomes. Reducing the costs associated with health

	resource use and long-term care admissions were also identified goals of half of the interventions. Six interventions explicitly described the importance of identification of health needs over a number of physical, psychological and social domains. Behaviour change and self-management promotion was mentioned by four studies. Other goals of interventions included reducing care-giver burden, promoting appropriate health care access and increasing human interaction within care provision.<br><br>Rationale: An ageing population and current complexity with the identification of risk, as well as the prevalence of unmet need and treatable conditions. Furthermore, variations in screening and appropriate service provision were seen as important to address by interventions. The majority of interventions claimed to be based upon previous evidence regarding the benefits of multidisciplinary screening, multidisciplinary or specialist input on outcomes for older people. More than half of the studies were based on previous study findings and/or pilot work. Home visits were believed to be key to success in at least 11 interventions. The importance of the nurse role was mentioned by two studies. Other rationalisation included the perceived benefits of behaviour change techniques and care-giver support.
3. What (materials)	One study did not report on the materials used. Over 20 interventions described the use of assessment tools which covered a range of domains. The delivery of these appears to have been by a healthcare professional in most cases, although one was posted to participants and their caregivers to complete. Care note access was required in nine interventions. Over half of interventions mentioned access to referrals. Eight interventions communicated recommendations to GPs, five to participants. Four interventions listed training for staff and five provided guidelines. Three interventions refer to the provision of resource information. Participants were provided with equipment for the monitoring of health conditions in one intervention. One intervention explicitly refers to equipment used to assess a participant for use by a healthcare professional. Recording documentation which was used by health care professionals, the research team and the participant was mentioned in six studies.
4. What (procedures)	All interventions involved multi-domain assessments although one was carried out as self-assessment by post; the majority were undertaken by trained healthcare staff. A range of domains were incorporated including, among others, physical health, cognition, mental health, medication and social aspects. All interventions develop some sort of care planning; nine interventions explicitly refer to consultation and agreement on this planning with the participant. Reviewing of the planning was mentioned across the interventions, however the way in which this took place was varied, sometimes with face-to-face contact at home while others placed telephone calls. Actions from the assessment and planning were often related to referrals on to other services, and/or the provision of the information and advice, be that to the participant or caregiver or other healthcare staff. Five interventions explicitly refer to the need for participants to take actions themselves. Nine described support from others to sustain the recommendations and actions. Multidisciplinary discussion was mentioned by at least three interventions. Access to usual care was described as maintained in at least 13 interventions.
5. Who provided	Nurses, including those with more general and specific skill sets were the main implementers of the intervention in 17 descriptions. Geriatrician input was part of eight interventions; GPs were significant contributors to five

	interventions. Other professionals defined as involved included social workers and physicians' assistants. Multidisciplinary input was often described in the interventions as accessed when required; this would be from dietitians, physiotherapists, pharmacists and health visitors among others.
6. How	Intervention provision was primarily to the participant on an individual basis. Twenty-one interventions described face-to face contact, 16 are explicitly at the participant's own home. Nine explicitly described telephone contact with participants at some point in the process, be that initial assessment or follow-up. Two studies were conducted in clinics and two others were primarily utilising routine care note data. One was conducted through self-assessment by post. Three interventions described the need for family or caregiver involvement.
6b. How organised	Organisation of the intervention was not always clear or explicitly stated. The intervention was organised by a range of individuals most frequently, in at least 14 cases, this was by a nurse. Having the input of a range of individuals was mentioned in eight studies; GP input was mentioned in twelve studies, although at times this was suggested as not required. The participant or their caregiver was expected to co-ordinate their response to the assessment in at least four interventions. Geriatricians took a lead role in organisation for three interventions.
7. Where	At least some of the intervention was provided to the participants at their home in 21 described studies. Health care settings including rehabilitation centres and clinics were also delivery sites in at least seven studies.<br><br>Eleven interventions were provided in European countries, including The Netherlands, the UK, Germany, France, Denmark and Switzerland. The USA and Canada were the location of a further nine interventions. Three interventions were provided in Asian locations, Hong Kong and Japan. Australia was the site of one intervention.
8. When and how much	Not all studies described the inclusion and exclusion criteria for involvement. Studies varied significantly on how they recruited and involved participants. Some used age as a limitation, however this varied from 50 years and over to 80 years and over. Some studies included those who had been recently discharged from hospital or were awaiting other service input. Some studies used assessment of frailty level or disability as an inclusion criterion. Some studies excluded individuals who were severely ill or living with dementia or severe cognitive impairments. Other exclusions were based on the intervention being supported by the GP or geographical limitations.<br><br>The frequency, duration and nature of input across interventions was highly variable. In some studies this was unclear. Some interventions provided a minimum of one contact at assessment only, whilst others provided a range of contacts based on need. Length of involvement in the intervention was also varied, from a minimum of seven weeks to four years; most interventions were around 1-2 years. Frequency of contact ranged from bi-weekly to annual input. Visit length was described in at least nine studies, the duration of visits being between 20 minutes and two hours. The nature of the follow up interaction was less formally described and often appeared to be tailored.
9. Tailoring	All intervention reports described some level of tailoring. Twenty-three interventions reflected tailoring to the needs identified for the participant during their assessment. Most of this included the number and duration of contacts. Nine interventions described collaboration with the participant,

	whilst four had a preference for input from caregivers or family as well. Four interventions mentioned contribution from GP's or Pharmacists as and when required. Flexibility about the location of the intervention delivery was also mentioned in two studies.
10. Modifications	Only one intervention described a required modification, this was due to a lack of equipment and the need to adjust the aim of the intervention.
11. How well (planned)	Approaches to measuring how well the intervention worked were described for 16 studies, the remaining eight did not have descriptions of this. To promote adherence and fidelity seven studies described training and supervision of providers, three studies implemented follow up contact, three had used piloting work to improve the feasibility of the intervention, two used other pre-existing groups to enhance the intervention, two used family input to promote compliance, one used a small team of nurses to promote good relationships, one used goal setting approaches and a postal questionnaire included stamped addressed envelopes to promote questionnaire return. Various approaches to measuring adherence were described while around ten studies just described this generally, five mentioned specific documentation on assessment or follow-up visits and discussions with participants, three described analysis of the recommendations, and three referred to the collection of barriers and facilitators. Only one study described evaluation of the intervention by the participant.
12. How well (actual)	Eight studies did not report on how well the interventions actually worked. This information was compiled in variable ways including a measurement of compliance. Full compliance was reported for seven studies, varying between 13% and 90%. Partial compliance was reported for eight studies, varying between 42% and 97%. Three studies reported on the number of problems identified. Two collected information on the time spent by nurses at visits, or the number of visits undertaken. One study reported on sustainability over time. A number of other studies described barriers to their intervention including resistance from other clinicians in three studies, a lack of motivation to change or disagreement from participants was mentioned by two studies, logistic issues in one study, feasibility perception in one study, lack of financial resources for participants to act on recommendations in one study, and variability in the provider working style in one study. Three studies described variation in adherence to the recommendations, for example medication change had a higher adherence rate than changing smoking/alcohol use behaviours.

Group: Multifactorial-action and review with medication review and self-management strategies

There are three interventions in this group: Fox 1997 [129], Phelan 2007 [70], and van Leeuwen 2015 [90].

TIDieR item	Description
1. Brief name	
2. Why	Goal: The goals of the three interventions were broadly aligned and similar in nature. All three sought to improve health and reduce disability and poor health outcomes. One intervention was aiming to increase adherence to

	healthy behaviour advice. While the two others wanted to improve the quality of care and reduce hospitalisations. One of these interventions was also seeking to improve quality of life and reduce carer burden.<br><br>Rationale: Three interventions were rationalised on the premise that provision of health information may promote better self-care. Two interventions were based on evidence of success from similar approaches, one of these and one other had a behaviour change theory to ground the intervention development. One intervention was also based on policy recommendations, one on the idea that professionals trained in geriatric care were best placed to advise on supporting older people. The third suggested that there were benefits to early intervention and integrated care between various professionals and their patients.
**3. What (materials)**	There were a number of materials used in delivery of the interventions. All three interventions described care planning documentation. One also mentioned documentation to collect health history, a food and fluid diary, equipment to collect physiological data, various health advice materials and referrals. This and one other described materials used to record meeting information. Two interventions described heath assessments in some detail relating to validation and standardisation. One of these also ensured that the patient and GP received documentation of care planning.
**4. What (procedures)**	All three interventions utilised a multidomain assessment. All three also had a follow up or review procedure, although how this was conducted varied. Two interventions provided individualised health information and advice, one of these also described risk identification, referrals and behaviour change or motivational sessions. Two interventions created action or care plans, one described how specialist input from geriatricians and geropharmacists was enacted, including family caregiver involvement. Two studies detailed access to usual care.
**5. Who provided**	All three interventions had input from nurses, though these came from various specialisms including public health and geriatrics. Two interventions involved various gerontological specialists including geriatricians, and one also involved a geropharmacist. One intervention included a primary care practitioner.
**6. How**	All three interventions were provided face-to-face and individually with additional telephone contact.
**6b. How organised**	All three interventions were organised by team members, generally led by the nurses. Patient input was required in organisation of the intervention as well. The geriatric team described were required to support with organisation for two interventions. Two studies mentioned funding, one was by the state and the other was by a large health organisation.
**7. Where**	Two interventions took place in the USA, and one in The Netherlands. Two interventions were undertaken in clinics or community hubs. The other took place at the participant's home.
**8. When and how much**	Two interventions had minimum age limits; one was aged 60 years and over, this intervention was targeted at those with lower wealth and utilising the public health service for the first time. The other intervention limited by age was open to those aged 75 and over and using a particular health organisation. The third study targeted those who were identified as frail coexistent with polypharmacy.

	The nature and duration of input was somewhat varied across interventions, though all included an initial assessment. The review period was not always stated, however for one intervention this was every six months. One intervention required referrals to be enacted within three months.
9. Tailoring	All three interventions tailored planning in line with the needs and preferences of the participant. The frequency of follow up visits or review processes was also tailored in two interventions, specifically with an additional visit at three months on one study should this be required.
10. Modifications	No studies described modifications to the interventions.
11. How well (planned)	All three studies took some steps to increase adherence and fidelity. One intervention documented the recommendations made, and the implementation of these. One other engaged with supportive measures for providers to support with troubleshooting. The third intervention standardised processes to improve adherence, and measured implementation at the participant, provider and organisational level. In addition to this the third intervention also undertook qualitative work to identify barriers and facilitators to implementation.
12. How well (actual)	All three studies described how well their intervention was delivered to some extent. One found that around ¾ of participants were at least moderately adherent to the intervention recommendations, economic limits were identified as a barrier to adherence. Another intervention found almost ¾ of those invited received a visit, on average participants received two visits and six phone calls. The third intervention found that adherence for some components increased over time, while others decreased. Additionally, there was some variation in delivery between different providers. Of the providers who received the training, the motivational interview training was seen to be beneficial to practice, however the training on the assessment was not.

Group: Multifactorial-action and review with self-management strategies

There are two interventions in this group: Walters 2017 [96], and Wong 2019 [97].

TIDieR item	Description
1. Brief name	
2. Why	Goal: Both interventions aimed to support older people to live independently through addressing health and social problems proactively.<br>Rationale: Evidence bases such as literature reviews and stakeholder opinions identified that multifaceted strategies would optimise self-management change. Additionally, a range of theories and approaches exist for promoting successful ageing, self-efficacy, care management and behaviour change among others.
3. What (materials)	A range of materials were required for the interventions. One was manualised and used a range of health educational materials, equipment for exercise and planning documentation. The other used a structured assessment, and

	health educational materials, promotion of self-management through identification with older celebrities, and referral systems.
**4. What (procedures)**	The interventions both used care planning to identify relevant services and referrals. Self-efficacy and behaviour change techniques were used to promote monitoring and self-care. Additionally, regular routine follow up and review as well as access to all standardised care were features of both procedures. One intervention explicitly emphasised exercise, education and environmental change (i.e., home adaptation) as part of the assessment process, but this was not provided to all participants.
**5. Who provided**	One intervention was provided by a non-specialist support worker with training in behaviour change techniques. The other intervention used intervention-trained nurse case managers and community workers under the supervision of the nurse case managers.
**6. How**	Both interventions were focused on face-to-face interaction at assessment. One intervention explicitly involved a family carer in this. Other contacts could be undertaken by remote methods such as telephone or video calling. Techniques to promote self-care were focused on self-efficacy and behaviour change approaches.
**6b. How organised**	The interventions involved the nurse or support worker organising care in conjunction with the participant, in relation to the care planning. With one intervention this was explicitly reviewed and modified as required.
**7. Where**	One intervention was undertaken in a district of Hong Kong, the other in two regions of the UK.<br><br>The intervention was carried out at home.
**8. When and how much**	With one intervention, participants were eligible for involvement if they were 60 years or more and not engaged in other health or social programmes. The other intervention recruited those who were 65 years or more and classified as mildly frail.<br><br>The number, frequency and duration of visits differed between the interventions. Face-to-face contacts ranged from 30-120 minutes, with an expectation of a minimum of six contacts. Telephone contacts were mentioned as being 6-12 minutes long by one intervention.
**9. Tailoring**	The interventions were tailored based on the co-developed care plan which identified the participants' needs, goals and wishes. One intervention also tailored the behaviour change technique to the participant.
**10. Modifications**	Not mentioned
**11. How well (planned)**	For both interventions, fidelity and adherence were promoted through training providers in intervention delivery and recording and documenting the contact sessions with participants. One intervention also included case conference meetings, the other involved consultation with stakeholders to facilitate intervention delivery.
**12. How well (actual)**	Only one study reported on actual adherence. For this intervention, delivery was largely as intended with coverage of a range of domains and tailored goals identified. 96% of participants identified at least one goal, fidelity to the intervention at appointments was assessed at 72.1%, attendance at appointments was 91.3%.

Group: Multifactorial-action with medication review

There are five interventions in this group: Balaban 1988 [9], Mann J 2021 [57], Newbury 2001 [66], Rockwood 2000 [73], and Sherman 2016 [78].

TIDieR item	Description
1. Brief name	
2. Why	Goal: Although goals were varied common aims were to identify those who were at risk of having unmet needs, often including social needs, could benefit from additional care and support with a goal of improving wellbeing, reducing admissions to hospital and retaining functional independent living in the community. Some interventions utilised goal setting and tailoring approaches to improve the likelihood to success.<br><br>Rationale: Evidence indicates there is unmet need in the older population which may lead to higher level resource use. Identification of those at risk and person-centred planning may be an appropriate preventative measure in improving health outcomes for older people as well as reducing admissions to hospital.
3. What (materials)	Materials required were not mentioned in one study. However, for the remaining four a range of approaches were used to undertake assessments. Some assessments were completed using routinely collected data, all involved Nurse or clinician visits to carry out a physical assessment and questionnaires. The process was usually documented in participants' patient records and relevant prescriptions and referrals to services were made. One intervention used goal setting as part of the process.
4. What (procedures)	Procedures were different across the interventions although all carried out an assessment of needs, usually this was explicitly undertaken at the participant's home, this was primarily focused upon medical and social needs, however a psychosocial and functional approach was taken with one assessment. Assessments were usually undertaken by Nurses, sometimes with multidisciplinary input as well. Medication checks were included in all five assessments. One intervention took a person-centred approach and explicitly incorporated the wishes of the participant, another intervention also undertook goal setting at assessment. The provision of the recommended care was sometimes the role of the participants own GP, other times this was provided as part of the intervention. In one intervention it was unclear who would act on recommendations made. Follows were mentioned as part of two intervention procedures.
5. Who provided	Primarily interventions were provided by nurses, some of whom were specialised in geriatric care. Two interventions involved geriatricians in the assessment phase. One used a programme physician.<br><br>One intervention explicitly referred to the involvement of physiotherapists, Occupational Therapists, Social Workers, Dietitians, Audiologists and Speech and Language Therapists as part of the care carried out following assessment. Other interventions relied on GPs to enact required care.
6. How	Only one study mentioned how participants were initially contact this was by letter and telephone. All studies refer to contact with clinicians, for most

	interventions this was at the participant's home and presumably therefore was face-to-face.
6b. How organised	In four interventions there was significant nurse input. Although with one study it was not clear who was in charge of the care planning process, this was usually undertaken by a nurse, with support from physicians or specialist geriatricians in two studies. The recommendations were at times carried out as part of the intervention and other times were sent to the participant's GP.
7. Where	A range of international locations were involved, including Australia, Sweden and Canada.<br>Interaction with participants was usually at home or in a primary care facility.
8. When and how much	Identification of participants varied. Three studies involved people based on age, those 75 and over for two of these and the other involved those aged 70 plus, or 50 plus who appeared at risk due to physical and/or social needs limiting their access to services or increasing risk of ill health. The two remaining interventions were access based on their risk of decline related to health or social illbeing.<br>All studies had a minimum of one visit, the remaining contact was based on needs identified.
9. Tailoring	All studies mentioned some elements of tailoring, given that assessments were aiming to identify specific needs. The need for follow up care and recommendations were mentioned as tailored in four of the interventions. The timing and location of assessment (and if necessary, the follow up) was also mentioned as flexible for three studies.
10. Modifications	Only one intervention mentioned modifications - the nature of these was not specified.
11. How well (planned)	Three studies explicitly refer to training to enhance fidelity, additionally two of these also used reliability checks on the assessments made. One intervention had also been part of a feasibility pilot.
12. How well (actual)	Studies varied in reporting how well the intervention worked. Two made no reference to implementation effectiveness. One stated that on average participants who were able received on average 2.0-3.8 visits. Another reported on inter-rater reliability of assessments being between 0.79-0.94 across assessors. One other intervention was reported as carried out as planned and the process was straightforward.

Group: Nutrition and exercise

There are three interventions in this group: Loh 2015 [55], Serra-Prat 2017 [76], and van Dongen 2020 [87].

TIDieR item	Description
1. Brief name	
2. Why	Goal: The long-term aim of the three interventions was to improve frailty status, physical functioning and/or reduce loss of independence. For one intervention good oral care was implicated.

	Rationale: Previous research implicates muscle wastage as a contributing factor to frailty; insufficient or poor diet also contributes to this health state. Evidence supports the use of multicomponent nutritional and exercise programme in enhancing physical functioning.
3. What (materials)	The interventions primarily used a combination of leaflets and educational information such as DVDs describing or promoting physical exercise and providing nutritional advice. In addition to this some interventions provided referrals to nutritional units, training sessions and checklists relating to physical exercise and nutritional exercise undertaken, and oral care advice. One intervention provided cash rewards for involvement in sessions.
4. What (procedures)	A range of processes were seen across the interventions. Screening and identification of particular risk was seen in one of the interventions. All interventions involved an exercise session with provision of an exercise programme to be undertaken at home, one of the interventions provided a more tailored programme. Nutritional advice provision was more varied, involving screening and referral, workshops and a tailored diet provision or group advice sessions. Interventions provided a range of other activities designed to promote adherence including phone calls, training and support for healthcare professionals, goal setting and peer engagement.
5. Who provided	Who provided the intervention was not always clear. When stated, a range of healthcare professionals were seen to be involved. Nutritional advice was provided by dietitians or nutritionists, physical activity training was provided by physiotherapists or trained fitness instructors. Other professionals, including health promotion employees were involved to facilitate involvement.
6. How	Although not always clear in the reporting, physical training and nutritional exercise appeared to have been provided face-to-face. Some of these sessions were group or workshop based. Some sessions had motivational techniques built in. Additional educational supplements were supplied. Telephone calls were provided to enhance adherence and for additional consultation purposes.
6b. How organised	This was either not mentioned or somewhat unclear in reporting for two interventions, suggesting involvement by various disciplines in executing relevant aspects such as the nutritional assessment, overseen by the research nurse. One intervention was partially coordinated by care sport collaborators who connected primary care services and the sports sector.
7. Where	The interventions were carried out in Spain, The Netherlands and Malaysia. In some studies there was little detail about the locations of the intervention, it was suggested that primary care centres were used. Two of the interventions detailed either the use of local sports settings and/or community facilities.
8. When and how much	Only two interventions provide details relating to eligibility, one intervention was aimed at those 60 and over, the other stipulated 70 and over with prefrailty.<br><br>There was variation in the number frequency, duration and nature of contact across the interventions. The exercise component varied in input from one session with recommendation to follow an exercise plan at home, to 24 weeks of sessions which decreased from hourly bi-weekly sessions to weekly sessions. The nutritional component varied from input only upon referral to 6, 30-minute sessions. The intervention which provided oral care advice included 2 sessions.

9. Tailoring	This was not always reported upon. One intervention provided referral to dietary services if their nutritional assessment showed a risk. One intervention provided tailoring to all components including tailored exercise programmes and dietary advice. Additionally, participants could choose to attend additional activities that were offered.
10. Modifications	Not mentioned for any of the studies.
11. How well (planned)	The reporting of this varied across interventions. From a planned process evaluation to detail attendance, satisfaction, enablers and barriers to involvement, to attendance records for physical activity and dietary intake and/or telephone contact to monitor compliance.
12. How well (actual)	This was not reported for all interventions. For one study 47.5% were considered to have adhered at 12 month follow up. One intervention found that attendance was high at intensive support sessions (first 12 weeks), between 98.8% and 83.6%, but lower at (later) moderate support sessions, between 59.8% and 56.1%. Protein intake improved from baseline to 12 weeks and still remained higher than baseline at 24 weeks follow up.

Group: Risk-screening

There are six interventions in this group: Bleijenberg 2016 (2 interventions) [11], Carpenter 1990 [17], Jitapunkul 1998 [46], Kerse 2014 [47], and Pathy 1992 [69].

TIDieR item	Description
1. Brief name	
2. Why	Goal: to preserve daily functioning and enhance their quality of life and maintain community living. Two interventions clearly mentioned the identification of those at risk of decline or with unmet needs.<br><br>Rationale: based on evidence that there are older people living with unmet needs and identification of those at risk and with unmet needs through appropriate screening targeted action planning can be achieved in other similar programmes and pilot work.
3. What (materials)	A range of screening assessments or electronic patient records were used in the identification of risk and unmet needs including frailty measures, at least one of these assessments was explicitly by postal self-report.<br><br>Guidelines on the appropriate prescription of aids, medication or referral to health and social services following assessment varied across the interventions.
4. What (procedures)	Identification through the screening of patient records or using questionnaires and assessments either delivered by a range of individuals, from volunteers to trained health care professionals, for at least one intervention this was undertaken through self-assessment by the older person.<br><br>Identification of those deemed at risk or with unmet need resulted in a protocol to be enacted for accessing appropriate care. This was usually through needs based tailored referrals to health and social care services, prescriptions and access to aids.

5. Who provided	Screening assessments were undertaken by a range of individuals from volunteers to nurses, non-professionals and trained staff. One study mentioned interpretation of the screening assessment by a trained nurse. All but one intervention detailed that the reports generated were to be acted upon by the participant's GP or a geriatrician. Other health and social care professionals were to be involved with enactment of care as was relevant.
6. How	For one intervention this was not described. For at least three interventions the initial assessment was done at distance, usually by post. One described an at home face-to-face assessment. The follow up of any required care based on the screening assessments was explicitly to be undertaken individually and at home by relevant clinicians in two interventions. Location and type of follow-up care was less clear in two further interventions, and not mentioned in the remaining two.
6b. How organised	Organisation of the screening process was by a range of individuals or was unstated for some of the interventions. Organisation of the subsequent care was often undertaken by the participants GP or members of the GP practice such as health visitors or nurses. The interventions varied as to whether the care was unidisciplinary or multidisciplinary. One intervention explicitly refers to organisation by the research team and facilitation by the district health board.
7. Where	Four of the interventions were based in Europe, two in the UK, two in The Netherlands. One was in New Zealand and one in Thailand.<br><br>Two interventions did not specify a location for carrying out the intervention, one stated that some assessment will be carried out at home, three others mentioned that the intervention was based in the participant's home.
8. When and how much	Eligibility for intervention involvement varied across studies, one did not mention a minimum age, two recruited at 60 years and over with an indication of multimorbidity, polypharmacy or lack of contact with services, one recruited at 65 and over, one at 75 and over, whilst another recruited at varying ages depending on ethnicity.<br><br>Repetition of the screening process was mentioned as being annual in one study or every three years in another.<br><br>Input from services according to need varied, four interventions explicitly mentioned follow up support being needs based, ranging from a minimum of one visit to quarterly visits for three years to as required.
9. Tailoring	One intervention did not mention any tailoring. The remaining five mentioned tailoring based on the assessments undertaken. Additional tailoring to the specific needs and required input by service for individuals including the nature and frequency of follow up visits and contact was also mentioned.
10. Modifications	Only one study described modifications which were required due to reforms to geriatric services
11. How well (planned)	Only two studies described approaches to maintain fidelity. One refers to use of manualised training of the staff involved, the other refers to use of manualised training and supervision of staff, collection of information on barriers and facilitators of the intervention and the undertaking of a 6-week pilot study.
12. How well (actual)	This was not mentioned by two of the studies. The feasibility of the interventions was variable and information relating to this differs. One

	intervention, while perceived as feasible by staff, only managed to deliver follow up care to a third of those assessed as in need or at risk. Referral rates in the study group exceeded the control group until the final year of the study for one intervention. One study saw assessment completion and return rates of 88%. Another study reported that 40% of those screened were not in need of visits.

References (appendix 7)

1. Dorresteijn TA, Zijlstra GA, Ambergen AW, Delbaere K, Vlaeyen JW, Kempen GI; Effectiveness of a home-based cognitive behavioral program to manage concerns about falls in community-dwelling, frail older people: results of a randomized controlled trial. *BMC Geriatr* 2016;**16**:2. doi: 10.1186/s12877-015-0177-y.
2. Siemonsma PC, Blom JW, Hofstetter H*, et al.*; The effectiveness of functional task exercise and physical therapy as prevention of functional decline in community dwelling older people with complex health problems. *BMC Geriatr* 2018;**18**(1):164. doi: 10.1186/s12877-018-0859-3.
3. Szanton SL, Thorpe RJ, Boyd C*, et al.*; Community aging in place, advancing better living for elders: a bio-behavioral-environmental intervention to improve function and health-related quality of life in disabled older adults. *J Am Geriatr Soc* 2011;**59**(12):2314–20. doi: 10.1111/j.1532-5415.2011.03698.x.
4. Szanton SL, Xue QL, Leff B*, et al.*; Effect of a biobehavioral environmental approach on disability among low-income older adults: A randomized clinical trial. *JAMA Intern Med* 2019;**179**(2):204–211. doi: 10.1001/jamainternmed.2018.6026.
5. Borrows A, Holland R; Independent living centre occupational therapy (OT) versus routine community OT. *Int J Ther Rehabil* 2013;**20**(4):187–194. doi: 10.12968/ijtr.2013.20.4.187.
6. Tomita MR, Mann WC, Stanton K, Tomita AD, Sundar V; Use of currently available smart home technology by frail elders: process and outcomes. *Top Geriatr Rehabil* 2007;**23**(1):24–34. doi: 10.1097/00013614-200701000-00005.
7. Alegria M, Frontera W, Cruz-Gonzalez M*, et al.*; Effectiveness of a Disability Preventive Intervention for Minority and Immigrant Elders: The Positive Minds-Strong Bodies Randomized Clinical Trial. *Am J Geriatr Psychiatry* 2019;**27**(12):1299–1313. doi: 10.1016/j.jagp.2019.08.008.
8. Arthanat S; Promoting information communication technology adoption and acceptance for aging-in-place: A randomized controlled trial. *J Appl Gerontol* 2019;**40**(5):471–480. doi: 10.1177/0733464819891045.
9. Balaban DJ, Goldfarb NI, Perkel RL, Carlson BL; Follow-up study of an urban family medicine home visit program. *J Fam Pract* 1988;**26**(3):307–12.
10. Barenfeld E, Dahlin-Ivanoff S, Wallin L, Gustafsson S; Promoting aging migrants' capabilities: A randomized controlled trial concerning activities of daily living and self-rated health. *AIMS Public Health* 2018;**5**(2):173–188. doi: 10.3934/publichealth.2018.2.173.
11. Bleijenberg N, Drubbel I, Schuurmans MJ*, et al.*; Effectiveness of a proactive primary care program on preserving daily functioning of older people: a cluster randomized controlled trial. *J Am Geriatr Soc* 2016;**64**(9):1779–88. doi: 10.1111/jgs.14325.
12. Blom J, den Elzen W, van Houwelingen AH*, et al.*; Effectiveness and cost-effectiveness of a proactive, goal-oriented, integrated care model in general practice for older people. A cluster randomised controlled trial: Integrated Systematic Care for older People--the ISCOPE study. *Age Ageing* 2016;**45**(1):30–41. doi: 10.1093/ageing/afv174.
13. Botjes E; Methodebeschrijving EigenKrachtWijzer: Databank Effectieve sociale interventies Movisie, 2013.

14. Bouman A, van Rossum E, Ambergen T, Kempen G, Knipschild P; Effects of a home visiting program for older people with poor health status: a randomized, clinical trial in The Netherlands. *J Am Geriatr Soc* 2008;**56**(3):397–404. doi: 10.1111/j.1532-5415.2007.01565.x.
15. Brettschneider C, Luck T, Fleischer S*, et al.*; Cost-utility analysis of a preventive home visit program for older adults in Germany. *BMC Health Serv Res* 2015;**15**:141. doi: 10.1186/s12913-015-0817-0.
16. Cameron ID, Fairhall N, Langron C*, et al.*; A multifactorial interdisciplinary intervention reduces frailty in older people: randomized trial. *BMC Med* 2013;**11**:65. doi: 10.1186/1741-7015-11-65.
17. Carpenter GI, Demopoulos GR; Screening the elderly in the community: controlled trial of dependency surveillance using a questionnaire administered by volunteers. *BMJ* 1990;**300**(6734):1253–6. doi: 10.1136/bmj.300.6734.1253.
18. Cesari M, Demougeot L, Boccalon H, Guyonnet S, Vellas B, Andrieu S; The Multidomain Intervention to preveNt disability in ElDers (MINDED) project: rationale and study design of a pilot study. *Contemp Clin Trials* 2014;**38**(1):145–54. doi: 10.1016/j.cct.2014.04.006.
19. Clark F, Azen SP, Zemke R*, et al.*; Occupational therapy for independent-living older adults. A randomized controlled trial. *JAMA* 1997;**278**(16):1321–6. doi: 10.1001/jama.1997.03550160041036.
20. Clark F, Jackson J, Carlson M*, et al.*; Effectiveness of a lifestyle intervention in promoting the well-being of independently living older people: results of the Well Elderly 2 Randomised Controlled Trial. [Erratum in: J Epidemiol Community Health 2012;66:1079-82]. *J Epidemiol Community Health* 2012;**66**(9):782–790. doi: 10.1136/jech.2009.099754.
21. Coleman EA, Grothaus LC, Sandhu N, Wagner EH; Chronic Care Clinics: A randomized controlled trial of a model of primary care for frail older adults. *J Am Geriatr Soc* 1999;**47**(7):775–783. doi: 10.1111/j.1532-5415.1999.tb03832.x.
22. Counsell SR, Callahan CM, Clark DO*, et al.*; Geriatric care management for low-income seniors: a randomized controlled trial. *JAMA* 2007;**298**(22):2623–33. doi: 10.1001/jama.298.22.2623.
23. Cutchin MP, Coppola S, Talley V, Svihula J, Catellier D, Shank KH; Feasibility and effects of preventive home visits for at-risk older people: design of a randomized controlled trial. *BMC Geriatr* 2009;**9**:54. doi: 10.1186/1471-2318-9-54.
24. Dalby DM, Sellors JW, Fraser FD, Fraser C, van Ineveld C, Howard M; Effect of preventive home visits by a nurse on the outcomes of frail elderly people in the community: a randomized controlled trial. *CMAJ* 2000;**162**(4):497–500.
25. de Craen AJ, Gussekloo J, Blauw GJ, Willems CG, Westendorp RG; Randomised controlled trial of unsolicited occupational therapy in community-dwelling elderly people: the LOTIS trial. *PLoS Clin Trials* 2006;**1**(1):e2. doi: 10.1371/journal.pctr.0010002.
26. Fabacher D, Josephson K, Pietruszka F, Linderborn K, Morley JE, Rubenstein LZ; An in-home preventive assessment program for independent older adults: a randomized controlled trial. *J Am Geriatr Soc* 1994;**42**(6):630–8. doi: 10.1111/j.1532-5415.1994.tb06862.x.
27. Fairhall N, Kurrle SE, Sherrington C*, et al.*; Effectiveness of a multifactorial intervention on preventing development of frailty in pre-frail older people: study protocol for a randomised controlled trial. *BMJ Open* 2015;**5**(2):e007091. doi: 10.1136/bmjopen-2014-007091.
28. Fischer G, Sandholzer H, Perschke-Hartmann C; Final report of the scientific support of “Getting Healthy Elderly (GÄW)”. A prevention project of the AOK Lower Saxony.)[German] (Abschlussbericht der wissenschaftlichen Begleitung von "Gesund Älter Werden (GÄW)“). *Ein Präventionsprojekt der AOK Niedersachsen. AOK Niedersachsen, unveröffentlichtes Dokument*: AOK Niedersachsen, 2009.
29. Ford AB, Katz S, Downs TD, Adams M; Results of long-term home nursing: the influence of disability. *J Chronic Dis* 1971;**24**(9):591–596. doi: 10.1016/0021-9681(71)90047-6.

30. Gene Huguet L, Navarro Gonzalez M, Kostov B*, et al.*; Pre Frail 80: Multifactorial intervention to prevent progression of pre-frailty to frailty in the elderly. *J Nutr Health Aging* 2018;**22**(10):1266–1274. doi: 10.1007/s12603-018-1089-2.
31. Gill TM, Baker DI, Gottschalk M, Peduzzi PN, Allore H, Byers A; A program to prevent functional decline in physically frail, elderly persons who live at home. *N Engl J Med* 2002;**347**(14):1068–74. doi: 10.1056/NEJMoa020423.
32. Giné-Garriga M, Sansano-Nadal O, Tully MA*, et al.*; Accelerometer-measured sedentary and physical activity time and their correlates in European older adults: The SITLESS study. *J Gerontol A Biol Sci Med Sci* 2020;**75**(9):1754–1762. doi: 10.1093/gerona/glaa016.
33. Gitlin LN, Winter L, Dennis MP, Corcoran M, Schinfeld S, Hauck WW; A randomized trial of a multicomponent home intervention to reduce functional difficulties in older adults. *J Am Geriatr Soc* 2006;**54**(5):809–16. doi: 10.1111/j.1532-5415.2006.00703.x.
34. Grimmer K, Luker J, Beaton K, Kumar S, Crockett A, Price K; TRialing individualized interventions to prevent functional decline in at-risk older adults (TRIIFL): study protocol for a randomized controlled trial nested in a longitudinal observational study. *Trials* 2013;**14**:266. doi: 10.1186/1745-6215-14-266.
35. Gustafson DH, Kornfield R, Mares M-L*, et al.*; Effect of an eHealth intervention on older adults' quality of life and health-related outcomes: a randomized clinical trial. *J Gen Intern Med* 2021;**37**(3):521–530. doi: 10.1007/s11606-021-06888-1.
36. Gustafsson S, Eklund K, Wilhelmson K*, et al.*; Long-Term Outcome for ADL Following the Health-Promoting RCT—Elderly Persons in the Risk Zone. *Gerontologist* 2013;**53**(4):654–663. doi: 10.1093/geront/gns121.
37. Harari D, Iliffe S, Kharicha K*, et al.*; Promotion of health in older people: a randomised controlled trial of health risk appraisal in British general practice. *Age Ageing* 2008;**37**(5):565–71. doi: 10.1093/ageing/afn150.
38. Hay WI, van Ineveld C, Browne G*, et al.*; Prospective care of elderly patients in family practice. Is screening effective? *Can Fam Physician* 1998;**44**:2677–87.
39. Hebert R, Robichaud L, Roy PM, Bravo G, Voyer L; Efficacy of a nurse-led multidimensional preventive programme for older people at risk of functional decline. A randomized controlled trial. *Age Ageing* 2001;**30**(2):147–53. doi: 10.1093/ageing/30.2.147.
40. Henderson MJ; In-home preventive health assessment and telephone case management for over 75s living alone in independent living units: A cluster randomized controlled trial. *Faculty of Health, Center for Health Research - Nursing*. Queensland: Queensland University of Technology, 2005.
41. Hendriksen C, Lund E, Stromgard E; Consequences of assessment and intervention among elderly people: a three year randomised controlled trial. *Br Med J (Clin Res Ed)* 1984;**289**(6457):1522–4. doi: 10.1136/bmj.289.6457.1522.
42. Hogg W, Lemelin J, Dahrouge S*, et al.*; Randomized controlled trial of anticipatory and preventive multidisciplinary team care: for complex patients in a community-based primary care setting. *Can Fam Physician* 2009;**55**(12):e76–85.
43. Holland SK, Greenberg J, Tidwell L, Malone J, Mullan J, Newcomer R; Community-based health coaching, exercise, and health service utilization. *J Aging Health* 2005;**17**(6):697–716. doi: 10.1177/0898264305277959.
44. Howel D, Moffatt S, Haighton C*, et al.*; Does domiciliary welfare rights advice improve health-related quality of life in independent-living, socio-economically disadvantaged people aged ≥60 years? Randomised controlled trial, economic and process evaluations in the North East of England. *PLoS One* 2019;**14**(1):e0209560. doi: 10.1371/journal.pone.0209560.
45. Imhof L, Naef R, Wallhagen MI, Schwarz J, Mahrer-Imhof R; Effects of an advanced practice nurse in-home health consultation program for community-dwelling persons aged 80 and older. *J Am Geriatr Soc* 2012;**60**(12):2223–31. doi: 10.1111/jgs.12026.
46. Jitapunkul S; A randomised controlled trial of regular surveillance in Thai elderly using a simple questionnaire administered by non-professional personnel. *J Med Assoc Thai* 1998;**81**(5):352–6.

47. Kerse N, McLean C, Moyes SA, *et al.*; The cluster-randomized BRIGHT trial: Proactive case finding for community-dwelling older adults. *Ann Fam Med* 2014;**12**(6):514–24. doi: 10.1370/afm.1696.
48. Kono A, Kai I, Sakato C, Harker JO, Rubenstein LZ; Effect of preventive home visits for ambulatory housebound elders in Japan: a pilot study. *Aging Clin Exp Res* 2004;**16**(4):293–9. doi: 10.1007/BF03324554.
49. Kukkonen-Harjula K, Karmeniemi P, Suikkanen S, *et al.*; Long-term home-based physiotherapy for older people with signs of frailty-RCT (NCT02305433) [P-229]. *Eur Geriatr Med* 2017;**8**(S1):S105. doi: 10.1016/S1878-7649(17)30179-1.
50. Lambotte D, De Donder L, De Roeck EE, *et al.*; Randomized controlled trial to evaluate a prevention program for frail community-dwelling older adults: a D-SCOPE protocol. *BMC Geriatr* 2018;**18**(1):194. doi: 10.1186/s12877-018-0875-3.
51. Leung AC-t, Liu C-p, Chow NW-s, Chi I; Cost-Benefit Analysis of a Case Management Project for the Community-Dwelling Frail Elderly in Hong Kong. *J Appl Gerontol* 2004;**23**(1):70–85. doi: 10.1177/0733464804263088.
52. Leveille SG, Wagner EH, Davis C, *et al.*; Preventing disability and managing chronic illness in frail older adults: a randomized trial of a community-based partnership with primary care. *J Am Geriatr Soc* 1998;**46**(10):1191–8. doi: 10.1111/j.1532-5415.1998.tb04533.x.
53. Liddle J, March L, Carfrae B, *et al.*; Can occupational therapy intervention play a part in maintaining independence and quality of life in older people? A randomised controlled trial. *Aust N Z J Public Health* 1996;**20**(6):574–8. doi: 10.1111/j.1467-842x.1996.tb01068.x.
54. Liimatta H, Lampela P, Laitinen-Parkkonen P, Pitkala KH; Effects of preventive home visits on health-related quality-of-life and mortality in home-dwelling older adults. *Scand J Prim Health Care* 2019;**37**(1):90–97. doi: 10.1080/02813432.2019.1569372.
55. Loh DA, Hairi NN, Choo WY, *et al.*; MultiComponent Exercise and theRApeutic lifeStyle (CERgAS) intervention to improve physical performance and maintain independent living among urban poor older people--a cluster randomised controlled trial. *BMC Geriatr* 2015;**15**:8. doi: 10.1186/s12877-015-0002-7.
56. Lood Q, Gustafsson S, Dahlin Ivanoff S; Bridging barriers to health promotion: a feasibility pilot study of the 'Promoting Aging Migrants' Capabilities study'. *J Eval Clin Pract* 2015;**21**(4):604–613. doi: 10.1111/jep.12345.
57. Mann J, Thompson F, McDermott R, Esterman A, Strivens E; Impact of an integrated community-based model of care for older people with complex conditions on hospital emergency presentations and admissions: a step-wedged cluster randomized trial. *BMC Health Serv Res* 2021;**21**(1):701. doi: 10.1186/s12913-021-06668-x.
58. Melis RJ, van Eijken MI, Teerenstra S, *et al.*; A randomized study of a multidisciplinary program to intervene on geriatric syndromes in vulnerable older people who live at home (Dutch EASYcare Study). *J Gerontol A Biol Sci Med Sci* 2008;**63**(3):283–90. doi: 10.1093/gerona/63.3.283.
59. Meng H, Friedman B, Wamsley BR, Mukamel D, Eggert GM; Effect of a consumer-directed voucher and a disease-management-health-promotion nurse intervention on home care use. *Gerontologist* 2005;**45**(2):167–76. doi: 10.1093/geront/45.2.167.
60. Messens L, Quinn S, Saez I, Cuidad Mas MJ, Squillace P, Laura A-G; Health monitoring and sOcial integration environMent for Supporting WidE ExTension of independent life at HOME (Home Sweet Home): Final Trial Evaluation Report. *ICT PSP—Health, Ageing and Inclusion Programme*. Antwerp: Zorgbedrijf Antwerpen, 2014.
61. Metzelthin SF, Van Rossum E, De Witte LP, *et al.*; Effectiveness of interdisciplinary primary care approach to reduce disability in community dwelling frail older people: Cluster randomised controlled trial. *BMJ* 2013;**347**:f5264. doi: 10.1136/bmj.f5264.
62. Moll van Charante EP, Richard E, Eurelings LS, *et al.*; Effectiveness of a 6-year multidomain vascular care intervention to prevent dementia (preDIVA): a cluster-randomised controlled trial. *Lancet* 2016;**388**(10046):797–805. doi: 10.1016/S0140-6736(16)30950-3.

63. Monteserin Nadal R, Altimir Losada S, Brotons Cuixart C*, et al.*; Randomized clinical trial on the efficacy of global geriatric assessment in primary care. [Spanish]. *Rev Esp Geriatr Gerontol* 2008;**43**(1):5–12. doi: 10.1016/s0211-139x(08)71144-2.
64. Morey MC, Peterson MJ, Pieper CF*, et al.*; The Veterans Learning to Improve Fitness and Function in Elders Study: a randomized trial of primary care–based physical activity counseling for older men. *J Am Geriatr Soc* 2009;**57**(7):1166–1174. doi: 10.1111/j.1532-5415.2009.02301.x.
65. Morgan GS, Haase AM, Campbell RM, Ben-Shlomo Y; A pilot randomised controlled trial of physical activity facilitation for older adults: feasibility study findings. *Pilot Feasibility Stud* 2019;**5**:40. doi: 10.1186/s40814-019-0414-9.
66. Newbury JW, Marley JE, Beilby JJ; A randomised controlled trial of the outcome of health assessment of people aged 75 years and over. *Med J Aust* 2001;**175**(2):104–7. doi: 10.5694/j.1326-5377.2001.tb143541.x.
67. Newcomer R, Maravilla V, Faculjak P, Graves MT; Outcomes of preventive case management among high-risk elderly in three medical groups: a randomized clinical trial. *Eval Health Prof* 2004;**27**(4):323–48. doi: 10.1177/0163278704270011.
68. Ng TP, Feng L, Nyunt MS, *et al.*; Nutritional, physical, cognitive, and combination interventions and frailty reversal among older adults: A randomized controlled trial. *Am J Med* 2015;**128**(11):1225–1236.e1. doi: 10.1016/j.amjmed.2015.06.017.
69. Pathy MS, Bayer A, Harding K, Dibble A; Randomised trial of case finding and surveillance of elderly people at home. *Lancet* 1992;**340**(8824):890–3. doi: 10.1016/0140-6736(92)93294-W.
70. Phelan EA, Balderson B, Levine M*, et al.*; Delivering effective primary care to older adults: a randomized, controlled trial of the senior resource team at group health cooperative. *J Am Geriatr Soc* 2007;**55**(11):1748–56. doi: 10.1111/j.1532-5415.2007.01416.x.
71. Ploeg J, Brazil K, Hutchison B*, et al.*; Effect of preventive primary care outreach on health related quality of life among older adults at risk of functional decline: randomised controlled trial. *BMJ* 2010;**340**:c1480. doi: 10.1136/bmj.c1480.
72. Profener F, Anders J, Dapp U, Minder CE, Golgert S, von Renteln-Kruse W; Acceptance of preventive home visits among frail elderly persons : Participants an non-participants in a Follow-up after 2 and 4 years within the LUCAS longitudinal study. [German]. *Z Gerontol Geriatr* 2016;**49**(7):596–605. doi: 10.1007/s00391-016-1127-9.
73. Rockwood K, Stadnyk K, Carver D*, et al.*; A clinimetric evaluation of specialized geriatric care for rural dwelling, frail older people. *J Am Geriatr Soc* 2000;**48**(9):1080–5. doi: 10.1111/j.1532-5415.2000.tb04783.x.
74. Romera-Liebana L, Orfila F, Segura JM*, et al.*; Effects of a primary care-based multifactorial intervention on physical and cognitive function in frail, elderly individuals: A randomized controlled trial. *J Gerontol A Biol Sci Med Sci* 2018;**73**(12):1688-1674. doi: 10.1093/gerona/glx259.
75. Rubenstein LZ, Alessi CA, Josephson KR, Trinidad Hoyl M, Harker JO, Pietruszka FM; A randomized trial of a screening, case finding, and referral system for older veterans in primary care. *J Am Geriatr Soc* 2007;**55**(2):166–74. doi: 10.1111/j.1532-5415.2007.01044.x.
76. Serra-Prat M, Sist X, Domenich R*, et al.*; Effectiveness of an intervention to prevent frailty in pre-frail community-dwelling older people consulting in primary care: a randomised controlled trial. *Age Ageing* 2017;**46**(3):401-407. doi: 10.1093/ageing/afw242.
77. Shapiro A, Taylor M; Effects of a community-based early intervention program on the subjective well-being, institutionalization, and mortality of low-income elders. *Gerontologist* 2002;**42**(3):334–41. doi: 10.1093/geront/42.3.334.
78. Sherman H, Soderhielm-Blid S, Forsberg C, Karp A, Tornkvist L; Effects of preventive home visits by district nurses on self-reported health of 75-year-olds. *Prim Health Care Res Dev* 2016;**17**(1):56–71. doi: 10.1017/S1463423614000565.

79. Stuck AE, Aronow HU, Steiner A, *et al.*; A trial of annual in-home comprehensive geriatric assessments for elderly people living in the community. *N Engl J Med* 1995;**333**(18):1184–9. doi: 10.1056/NEJM199511023331805.
80. Stuck AE, Minder CE, Peter-Wuest I, *et al.*; A randomized trial of in-home visits for disability prevention in community-dwelling older people at low and high risk for nursing home admission. *Arch Intern Med* 2000;**160**(7):977–86. doi: 10.1001/archinte.160.7.977.
81. Stuck AE, Moser A, Morf U, *et al.*; Effect of health risk assessment and counselling on health behaviour and survival in older people: a pragmatic randomised trial. *PLoS Med* 2015;**12**(10):e1001889. doi: 10.1371/journal.pmed.1001889.
82. Suijker JJ, van Rijn M, Buurman BM, Ter Riet G, Moll van Charante EP, de Rooij SE; Effects of nurse-led multifactorial care to prevent disability in community-living older people: Cluster randomized trial. *PLoS One* 2016;**11**(7):e0158714. doi: 10.1371/journal.pone.0158714.
83. Takahashi PY, Pecina JL, Upatising B, *et al.*; A randomized controlled trial of telemonitoring in older adults with multiple health issues to prevent hospitalizations and emergency department visits. *Arch Intern Med* 2012;**172**(10):773–779. doi: 10.1001/archinternmed.2012.256.
84. Thiel C, Braun T, Grüneberg C; Physical training as core component of multimodal treatment of older frail people-study protocol of a randomized controlled pilot study. *Z Gerontol Geriatr* 2019;**52**(1):45-60. doi: 10.1007/s00391-018-1443-3.
85. Thomas R, Worrall G, Elgar F, Knight J; Can they keep going on their own? A four-year randomized trial of functional assessments of community residents. *Can J Aging* 2007;**26**(4):379–90. doi: 10.3138/cja.26.4.379.
86. Tulloch AJ, Moore V; A randomized controlled trial of geriatric screening and surveillance in general practice. *J R Coll Gen Pract* 1979;**29**(209):733–40.
87. van Dongen EJ, Haveman-Nies A, Doets EL, Dorhout BG, de Groot LC; Effectiveness of a diet and resistance exercise intervention on muscle health in older adults: ProMuscle in Practice. *J Am Med Dir Assoc* 2020;**21**(8):1065–1072. doi: 10.1016/j.jamda.2019.11.026.
88. van Heuvelen MJ, Hochstenbach JB, Brouwer WH, *et al.*; Differences between participants and non-participants in an RCT on physical activity and psychological interventions for older persons. *Aging Clin Exp Res* 2005;**17**(3):236-245. doi: 10.1007/BF03324603.
89. van Hout HP, Jansen AP, van Marwijk HW, Pronk M, Frijters DF, Nijpels G; Prevention of adverse health trajectories in a vulnerable elderly population through nurse home visits: a randomized controlled trial [ISRCTN05358495]. *J Gerontol A Biol Sci Med Sci* 2010;**65**(7):734–42. doi: 10.1093/gerona/glq037.
90. van Leeuwen KM, Bosmans JE, Jansen AP, *et al.*; Cost-effectiveness of a chronic care model for frail older adults in primary care: Economic evaluation alongside a stepped-wedge cluster-randomized trial. *J Am Geriatr Soc* 2015;**63**(12):2494–2504. doi: 10.1111/jgs.13834.
91. van Lieshout MRJ, Bleijenberg N, Schuurmans MJ, de Wit NJ; The effectiveness of a PRoactive multicomponent intervention program on disability in independently living older people: A randomized controlled trial. *J Nutr Health Aging* 2018;**22**(9):1051–1059. doi: 10.1007/s12603-018-1101-x.
92. van Rossum E, Frederiks CM, Philipsen H, Portengen K, Wiskerke J, Knipschild P; Effects of preventive home visits to elderly people. *BMJ* 1993;**307**(6895):27–32. doi: 10.1136/bmj.307.6895.27.
93. Vetter NJ, Jones DA, Victor CR; Effect of health visitors working with elderly patients in general practice: a randomised controlled trial. *Br Med J (Clin Res Ed)* 1984;**288**(6414):369–72. doi: 10.1136/bmj.288.6414.369.
94. von Bonsdorff MB, Leinonen R, Kujala UM, *et al.*; Effect of physical activity counseling on disability in older people: A 2-year randomized controlled trial. *J Am Geriatr Soc* 2008;**56**(12):2188–2194. doi: 10.1111/j.1532-5415.2008.02000.x.

95. Wallace JI, Buchner DM, Grothaus L*, et al.*; Implementation and effectiveness of a community-based health promotion program for older adults. *J Gerontol A Biol Sci Med Sci* 1998;**53**(4):M301–6. doi: 10.1093/gerona/53a.4.m301.
96. Walters K, Frost R, Kharicha K*, et al.*; Home-based health promotion for older people with mild frailty: the HomeHealth intervention development and feasibility RCT. *Health Technol Assess* 2017;**21**(73):1–128. doi: 10.3310/hta21730.
97. Wong AKC, Wong FKY, Chang K; Effectiveness of a community-based self-care promoting program for community-dwelling older adults: A randomized controlled trial. *Age Ageing* 2019;**48**(6):852–858. doi: 10.1093/ageing/afz095.
98. Yamada Y, Ikegami N; Preventive home visits for community- dwelling frail elderly people based on minimum data set-home care: randomized controlled trial. *Geriatr Gerontol Int* 2003;**3**:236–242. doi: 10.1111/j.1444-1586.2003.00103.x.
99. Faul AC, Yankeelov PA, Rowan NL*, et al.*; Impact on geriatric assessment and self-management support on community-dwelling older adults with chronic illnesses. *J Gerontol Soc Work* 2009;**52**(3):230–249. doi: 10.1080/01634370802609288.
100. Hattori S, Yoshida T, Okumura Y, Kondo K; Effects of reablement on the independence of community-dwelling older adults with mild disability: A randomized controlled trial. *Int J Environ Res Public Health* 2019;**16**(20):3954. doi: 10.3390/ijerph16203954.
101. Morey MC, Ekelund C, Pearson M*, et al.*; Project LIFE: a partnership to increase physical activity in elders with multiple chronic illnesses. *J Aging Phys Act* 2006;**14**(3):324–43. doi: 10.1123/japa.14.3.324.
102. Jing L, Jin Y, Zhang X, Wang F, Song Y, Xing F; The effect of Baduanjin qigong combined with CBT on physical fitness and psychological health of elderly housebound. *Medicine* 2018;**97**(51):e13654. doi: 10.1097/MD.0000000000013654.
103. Auvinen K, Voutilainen A, Jyrkkä J, Lönnroos E, Mäntyselkä P; Interprofessional medication assessment among home care patients: any impact on functioning? Results from a randomised controlled trial. *BMC Geriatr* 2020;**20**:390. doi: 10.1186/s12877-020-01796-1.
104. Bernabei R, Landi F, Gambassi G*, et al.*; Randomised trial of impact of model of integrated care and case management for older people living in the community. *BMJ* 1998;**316**(7141):1348–51. doi: 10.1136/bmj.316.7141.1348.
105. Dupuy L, Froger C, Consel C, Sauzeon H; Everyday functioning benefits from an assisted living platform amongst frail older adults and their caregivers. *Front Aging Neurosci* 2017;**9**:302. doi: 10.3389/fnagi.2017.00302.
106. Fernandez-Barres S, Garcia-Barco M, Basora J*, et al.*; The efficacy of a nutrition education intervention to prevent risk of malnutrition for dependent elderly patients receiving Home Care: A randomized controlled trial. *Int J Nurs Stud* 2017;**70**:131–141. doi: 10.1016/j.ijnurstu.2017.02.020.
107. Fristedt S, Nystedt P, Skogar O; Mobile geriatric teams - a cost-effective way of improving patient safety and reducing traditional healthcare utilization among the frail elderly? A randomized controlled trial. *Clin Interv Aging* 2019;**14**:1911–1924. doi: 10.2147/CIA.S208388.
108. King AII, Parsons M, Robinson E, Jorgensen D; Assessing the impact of a restorative home care service in New Zealand: A cluster randomised controlled trial. *Health Soc Care Community* 2012;**20**(4):365–374. doi: 10.1111/j.1365-2524.2011.01039.x.
109. Lewin G, De San Miguel K, Knuiman M*, et al.*; A randomised controlled trial of the Home Independence Program, an Australian restorative home-care programme for older adults. *Health Soc Care Community* 2013;**21**(1):69–78. doi: 10.1111/j.1365-2524.2012.01088.x.
110. Mann WC, Ottenbacher KJ, Fraas L, Tomita M, Granger CV; Effectiveness of assistive technology and environmental interventions in maintaining independence and reducing home care costs for the frail elderly. A randomized controlled trial. *Arch Fam Med* 1999;**8**(3):210–7. doi: 10.1001/archfami.8.3.210.

111. Rooijackers TH, Kempen GIJM, Zijlstra GAR, *et al.*; Effectiveness of a reablement training program for homecare staff on older adults' sedentary behavior: A cluster randomized controlled trial. *J Am Geriatr Soc* 2021;**69**(9):2566–2578. doi: 10.1111/jgs.17286.
112. Teut M, Schnabel K, Baur R, *et al.*; Effects and feasibility of an Integrative Medicine program for geriatric patients-a cluster-randomized pilot study. *Clin Interv Aging* 2013;**8**:953-961. doi: 10.2147/CIA.S45242.
113. van der Pols-Vijlbrief R, Wijnhoven HAH, Bosmans JE, Twisk JWR, Visser M; Targeting the underlying causes of undernutrition. Cost-effectiveness of a multifactorial personalized intervention in community-dwelling older adults: A randomized controlled trial. *Clin Nutr* 2017;**36**(6):1498–1508. doi: 10.1016/j.clnu.2016.09.030.
114. Wolter A, Stolle C, Roth G, Rothgang H; Does the resident care assessment instrument improve long-term home care? - results of a nation-wide study in Germany. [German]. *Gesundheitswesen* 2013;**75**(1):29–32. doi: 10.1055/s-0032-1309013.
115. Parsons J, Rouse P, Robinson EM, Sheridan N, Connolly MJ; Goal setting as a feature of homecare services for older people: does it make a difference? *Age Ageing* 2012;**41**(1):24–9. doi: 10.1093/ageing/afr118.
116. Parsons M, Senior H, Kerse N, *et al.*; Should care managers for older adults be located in primary care? A randomized controlled trial. *J Am Geriatr Soc* 2012;**60**(1):86–92. doi: 10.1111/j.1532-5415.2011.03763.x.
117. Parsons M, Senior H, Kerse N, Chen MH, Jacobs S, Anderson C; Randomised trial of restorative home care for frail older people in New Zealand. *Nurs Older People* 2017;**29**(7):27–33. doi: 10.7748/nop.2017.e897.
118. Tuntland H, Aaslund MK, Espehaug B, Førland O, Kjeken I; Reablement in community-dwelling older adults: a randomised controlled trial. *BMC Geriatr* 2015;**15**:1–11. doi: 10.1186/s12877-015-0142-9.
119. Whitehead PJ, Walker MF, Parry RH, Latif Z, McGeorge ID, Drummond AE; Occupational Therapy in HomEcare Re-ablement Services (OTHERS): results of a feasibility randomised controlled trial. *BMJ Open* 2016;**6**(8):e011868. doi: 10.1136/bmjopen-2016-011868.
120. Hall N, De Beck P, Johnson D, Mackinnon K, Gutman G, Glick N; Randomized trial of a health promotion program for frail elders. *Can J Aging* 1992;**11**(1):72–91.
121. Markle-Reid M, Weir R, Browne G, Roberts J, Gafni A, Henderson S; Health promotion for frail older home care clients. *J Adv Nurs* 2006;**54**(3):381–95. doi: 10.1111/j.1365-2648.2006.03817.x.
122. Ryvicker M, Feldman PH, Rosati RJ, Sobolewski S, Maduro GA, Jr., Schwartz T; Improving functional outcomes in home care patients: impact and challenges of disseminating a quality improvement initiative. *J Healthc Qual* 2011;**33**(5):28–36. doi: 10.1111/j.1945-1474.2011.00156.x.
123. Stewart S, Harvey I, Poland F, Lloyd-Smith W, Mugford M, Flood C; Are occupational therapists more effective than social workers when assessing frail older people? Results of CAMELOT, a randomised controlled trial. *Age Ageing* 2005;**34**(1):41–6. doi: 10.1093/ageing/afh230.
124. Williams EI, Greenwell J, Groom LM; The care of people over 75 years old after discharge from hospital: an evaluation of timetabled visiting by Health Visitor Assistants. *J Public Health Med* 1992;**14**(2):138–44. doi: 10.1093/oxfordjournals.pubmed.a042711.
125. Challis D, Clarkson P, Williamson J, *et al.*; The value of specialist clinical assessment of older people prior to entry to care homes. *Age Ageing* 2004;**33**(1):25–34. doi: 10.1093/ageing/afh007.
126. Kono A, Kanaya Y, Fujita T, *et al.*; Effects of a preventive home visit program in ambulatory frail older people: a randomized controlled trial. *J Gerontol A Biol Sci Med Sci* 2012;**67**(3):302–9. doi: 10.1093/gerona/glr176.
127. Kono A, Izumi K, Yoshiyuki N, Kanaya Y, Rubenstein LZ; Effects of an updated preventive home visit program based on a systematic structured assessment of care needs for ambulatory frail older adults in Japan: A randomized controlled trial. *J Gerontol A Biol Sci Med Sci* 2016;**71**(12):1631–1637. doi: 10.1093/gerona/glw068.

128. Vass M, Avlund K, Lauridsen J, Hendriksen C; Feasible model for prevention of functional decline in older people: municipality-randomized, controlled trial. *J Am Geriatr Soc* 2005;**53**(4):563–8. doi: 10.1111/j.1532-5415.2005.53201.x.
129. Fox PJ, Breuer W, Wright JA; Effects of a health promotion program on sustaining health behaviors in older adults. *Am J Prev Med* 1997;**13**(4):257–64.
